# The Myxobacterial Antibiotic Myxovalargin: Biosynthesis, Structural Revision, Total Synthesis, and Molecular Characterization of Ribosomal Inhibition

**DOI:** 10.1021/jacs.2c08816

**Published:** 2023-01-05

**Authors:** Timm O. Koller, Ullrich Scheid, Teresa Kösel, Jennifer Herrmann, Daniel Krug, Helena I. M. Boshoff, Bertrand Beckert, Joanna C. Evans, Jan Schlemmer, Becky Sloan, Danielle M. Weiner, Laura E. Via, Atica Moosa, Thomas R. Ioerger, Michael Graf, Boris Zinshteyn, Maha Abdelshahid, Fabian Nguyen, Stefan Arenz, Franziska Gille, Maik Siebke, Tim Seedorf, Oliver Plettenburg, Rachel Green, Anna-Luisa Warnke, Joachim Ullrich, Ralf Warrass, Clifton E. Barry, Digby F. Warner, Valerie Mizrahi, Andreas Kirschning, Daniel N. Wilson, Rolf Müller

**Affiliations:** † Institute for Biochemistry and Molecular Biology, University of Hamburg, Martin-Luther-King-Platz 6, 20146 Hamburg, Germany; ‡ Helmholtz Institute for Pharmaceutical Research Saarland (HIPS), Helmholtz Center for Infection Research (HZI), Saarland University Campus, 66123 Saarbrücken, Germany; § Department of Pharmacy, Saarland University, 66123 Saarbrücken, Germany; ∥ German Center for Infection Research (DZIF), partner site Hannover-Braunschweig, 38124 Braunschweig, Germany; ⊥ Leibniz Universität Hannover, Institute of Organic Chemistry and Center for Biomolecular Drug Research (BMWZ), Schneiderberg 1B, 30167 Hannover, Germany; # Tuberculosis Research Section, Laboratory of Clinical Immunology and Microbiology, National Institute of Allergy and Infectious Disease, National Institutes of Health, Bethesda, Maryland 20892, United States; ∇ SAMRC/NHLS/UCT Molecular Mycobacteriology Research Unit & DST/NRF Centre of Excellence for Biomedical TB Research, Institute of Infectious Disease and Molecular Medicine and Department of Pathology, University of Cape Town, Rondebosch 7700, South Africa; ○ Department of Computer Science and Engineering, Texas A&M University, College Station, Texas 77843, United States; ◆ Institute of Medicinal Chemistry, Helmholtz Zentrum München, German Research Center for Environmental Health (GmbH), Ingolstaedter Landstr. 1, 85764 Neuherberg, Germany; ¶ MSD Animal Health Innovation GmbH, Zur Propstei, 55270 Schwabenheim, Germany; & Department of Molecular Biology and Genetics, Johns Hopkins University, Baltimore, Maryland 21205, United States; Howard Hughes Medical Institute, Johns Hopkins University School of Medicine, Baltimore, Maryland 21205, United States

## Abstract

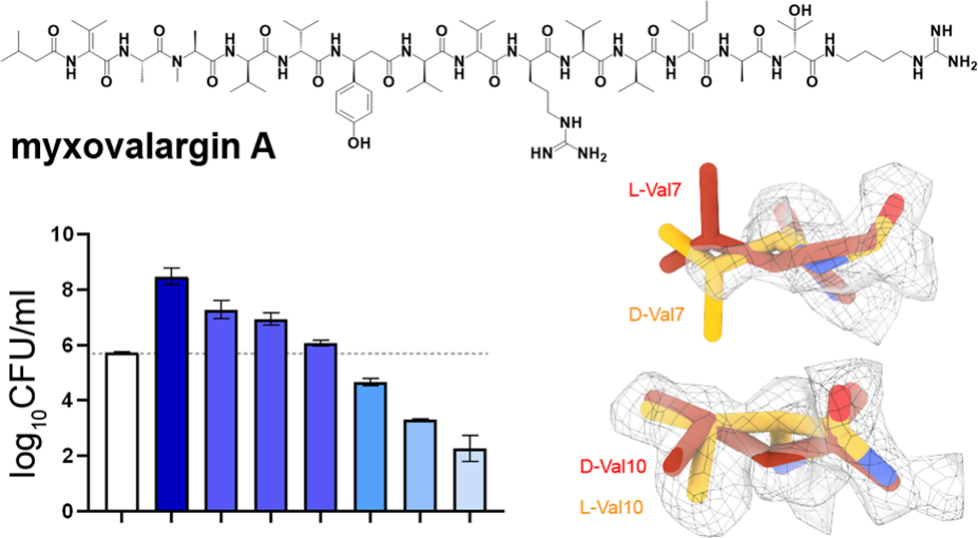

Resistance of bacterial pathogens against antibiotics is declared by WHO as a major global health threat. As novel antibacterial agents are urgently needed, we re-assessed the broad-spectrum myxobacterial antibiotic myxovalargin and found it to be extremely potent against *Mycobacterium tuberculosis*. To ensure compound supply for further development, we studied myxovalargin biosynthesis in detail enabling production via fermentation of a native producer. Feeding experiments as well as functional genomics analysis suggested a structural revision, which was eventually corroborated by the development of a concise total synthesis. The ribosome was identified as the molecular target based on resistant mutant sequencing, and a cryo-EM structure revealed that myxovalargin binds within and completely occludes the exit tunnel, consistent with a mode of action to arrest translation during a late stage of translation initiation. These studies open avenues for structure-based scaffold improvement toward development as an antibacterial agent.

## Introduction

Antimicrobial resistance (AMR) is on a constant rise and is significantly limiting treatment options against bacterial pathogens, especially those belonging to the WHO priority list including *Mycobacterium tuberculosis.*(1) Unfortunately, this trend is not matched by parallel developments of promising antibiotics due to a broken market for these life-saving drugs and numerous additional complications, which make the identification of promising antibiotic candidates exhibiting little or no cross-resistance with commonly used antibiotics extremely rare events.^2^

Microorganisms continue to be a major source for innovative antibiotics. Ubiquitously found but understudied soil-dwelling predatory myxobacteria have proven to be a rich source of bioactive compounds.^3−5^ Myxovalargin (Myx) was described in 1983 from *Myxococcus fulvus* strain Mx f65 as displaying activity against a broad panel of bacteria, exhibiting minimum inhibitory concentrations (MICs) between 0.3–5 μg/mL for Gram-positive and 6–100 μg/mL for Gram-negative bacteria, respectively.^6^ The myxovalargin structure was described in 1987 as comprising some congeners with Myxovalargin A (MyxA) as the predominant species.^7^ They were shown to most likely act on protein biosynthesis in bacteria and also exhibited an additional unspecific membrane effect at concentrations above 18 μg/mL, causing damage to eukaryotic cells including human erythrocytes, but did not inhibit eukaryotic translation at either dose.^8^ The efficacy in mouse models of infection with *Staphylococcus aureus,Streptococcus pyogenes*, and *Escherichia coli* could be shown at median efficacious doses (ED_50_) of 2.3, 2 × 0.4, and 2 × 10 mg/kg subcutaneously, respectively. However, compound development was stopped because of the limitations in compound availability and potential narrow therapeutic index, where the median lethal dose (LD_50_) in mice was determined at 10 mg/kg s.c. To our knowledge, neither the total synthesis nor a description of the biosynthetic pathway of Myx has been described.

In this study, we reassessed myxovalargin for its activity against *M. tuberculosis* and other pathogens and found the compound to exhibit potent antitubercular activity (MIC of 0.2 μg/mL) and a bactericidal mode-of-action, thus opening a potential therapeutic window for application. We identified the biosynthetic gene cluster, and biosynthetic reasoning followed by feeding experiments revealed that myxovalargins differ from the originally proposed structure, which was eventually corroborated by establishing a concise total synthesis of Myx. Next, the binding site of MyxA was visualized on the large ribosomal subunit using cryo-EM at between 2.1 and 2.5 Å resolution. The resolution enables a detailed description of the contacts that MyxA establishes with the ribosome, including water-mediated interactions, and provides a structural basis for the resistance mutations observed in *M. tuberculosis*. MyxA displayed good pharmacokinetic properties and showed no adverse effects after daily dosing of 2 mg/kg over 7 days. We demonstrated in vivo efficacy in a mouse model of *Pasteurella multocida* sepsis and then assessed myxovalargin in the more challenging mouse model of acute *M. tuberculosis* infection. Here, toxic effects after 1 week of treatment were observed, highlighting the necessity of further improving the safety margin of Myx. Understanding the structure–activity relationship is now feasible, and the cryo-EM structure opens avenues for the structure-based scaffold improvement of myxovalargin by using chemical and biotechnological production of analogues as a means to differentiate the specific inhibition of the ribosome from the unspecific toxic effect at higher doses.

## Results

### Bioactivity Profiling of Myxovalargin

**Figure 1:**
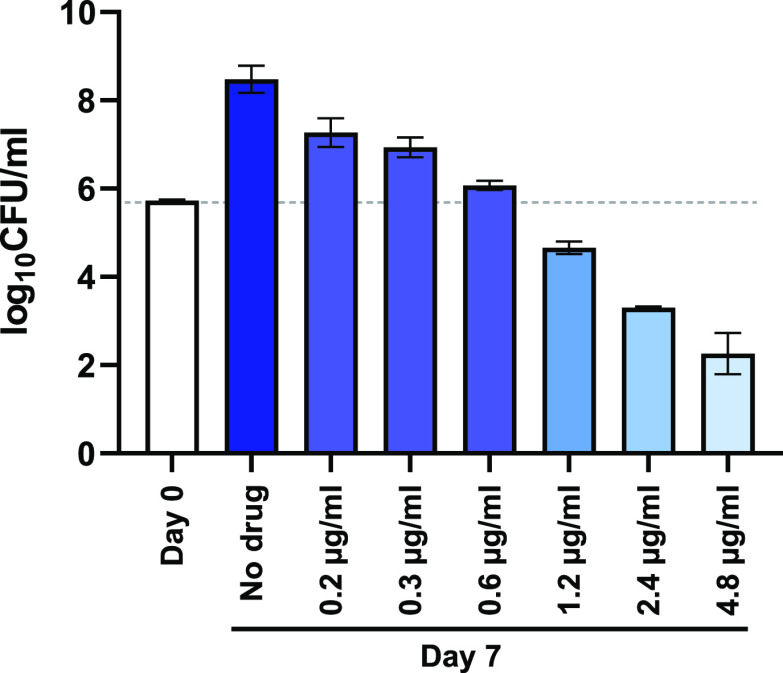
Dose-dependent bactericidal activity of MyxA. MyxA shows dose-dependent bactericidal activity against *M. tuberculosis* H37Rv in vitro. Limit of detection is 10 bacilli. Data are representative of the mean and SD of independent triplicates.

MyxA was identified as a potent inhibitor (MICs in sub- to low μg/mL range) from a screen of purified natural products of myxobacterial origin for growth inhibitory activity against *M. tuberculosis.* The natural product was bactericidal against *M. tuberculosis* in vitro (Figure 1) and bacteriostatic ex vivo (Figure S1–1). Spontaneous resistance mutants of *M. tuberculosis* were isolated at a frequency of ∼6 × 10^–7^ at 10-fold MIC. Whole-genome sequencing of mutant strains displaying MIC shifts of >40-fold (Table 1) revealed mutations in *rrl*, the 23S ribosomal RNA gene, identifying the ribosome as the likely antibacterial target of myxovalargins. The *rrl* mutations associated with *M. tuberculosis* resistance to MyxA showed limited overlap with those found in strains resistant to the oxazolidinone antibiotic linezolid, a key component of a new treatment-shortening regimen for extensively drug-resistant tuberculosis (XDR TB).^9−11^ In addition to the mutations detected in *rrl*, other SNPs were also identified in these three MyxA^R^ mutants (Table 1). While the SNP in *pks12* was synonymous, the other mutations map to the *ppsA* and *ppsE* genes, which are involved in the biosynthesis of phenolphthiocerol and phthiocerol dimycoserosate (PDIM). *M. tuberculosis* readily loses the ability to biosynthesize PDIM when propagated in vitro.^12^ We therefore considered it unlikely that these mutations contributed significantly to the MyxA^R^ phenotypes of any of the mutants. Besides characterizing the potent activity of MyxA against *M. tuberculosis*, we reinvestigated the spectrum of antimicrobial activity.^6^ The natural product efficiently kills Gram-positive bacteria and exerts moderate activity against Gram-negative pathogens, including an efflux-deficient strain of *E. coli* (Table S1-1). Encouragingly, MyxA also inhibited the growth of mastitis-causing pathogens *S. aureus* and *Streptococcus uberis* and Gram-negative bovine respiratory pathogens *Mannheimia haemolytica*, *P. multocida*, and *Histophilus somni* (Table S1-2) without causing any apparent cytotoxicity (HepG2 median cytotoxic dose CTD_50_ > 99 μM).

**Table 1:** Susceptibility of *M. tuberculosis* Strains to MyxA^a^

			MIC (μg/mL)		
strain	description	mutations	7H9/Glu/ADC/Tw medium	7H9/Glu/CAS/Tx medium	7H9/ADC/Twmedium
H37Rv	wildtype		4.9	0.2	0.2
MXV-SRM67	MyxA^R^ mutant	*rrl*: a2741g, Rv2931/ppsA: W1682*	>210	47	ND
MXV-SRM69	MyxA^R^ mutant	*rrl*: t2847c, *rrl*: t2849g, Rv2935/*ppsE*: G146*	>210	>210	ND
MXV-SRM70	MyxA^R^ mutant	*rrl*: t2847c, Rv2048c/*pks12*: A2643A	>210	8.4	ND

a MIC, minimal inhibitory concentration; 7H9, Difco Middlebrook 7H9 medium; Glu, glucose; ADC, Difco albumin–dextrose–catalase supplement; Tw, Tween 80; CAS, casitone; Tx, tyloxapol; media composition defined in SI; ND, not determined; *, stop codon.

Since the activity of MyxA was diminished in the presence of milk but not in the presence of serum (Table S1-2), subsequent profiling focused on respiratory pathogens. We determined the MIC_90_s (n ≥ 20) for *M. haemolytica* and *P. multocida* at 16 μg/mL and for *H. somniat* 8 μg/mL (Table S1-2 and Figure S1-2).

### In Vivo Pharmacokinetics and Pharmacodynamic Models with MyxA

**Figure 2:**
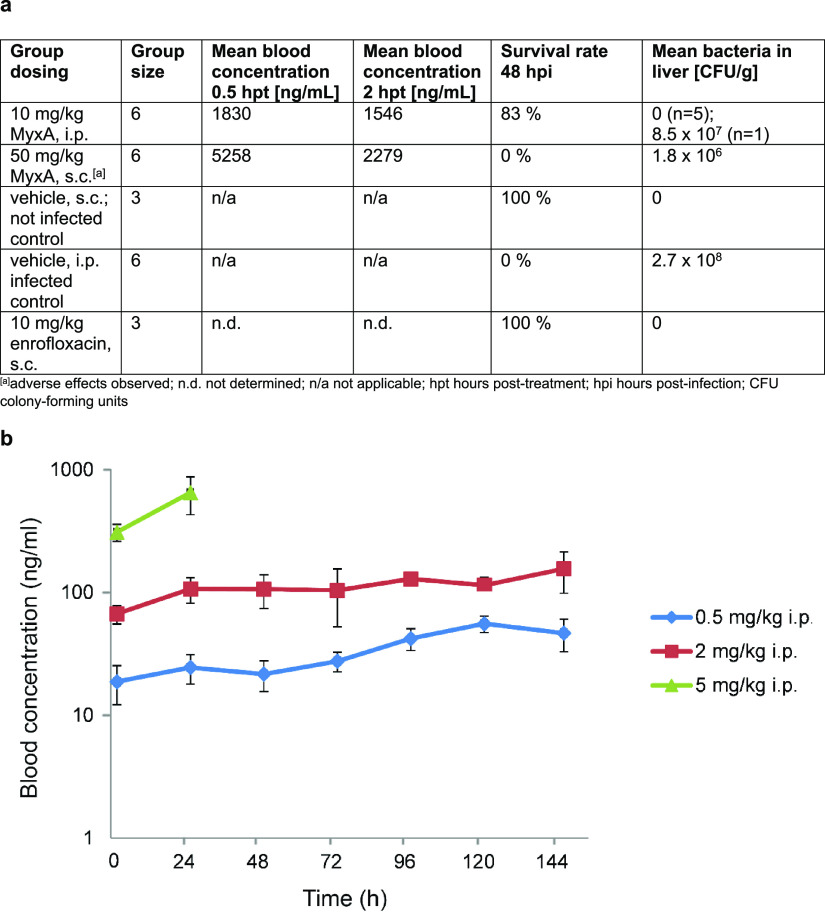
In vivo results with *P. multocida* and blood concentration time profile*.* (a), Summary of in vivo results with MyxA in *P. multocida* sepsis mouse model. (b) Blood concentration time profiles of MyxA in male C57Bl6 mice after intraperitoneal administration of 0.5, 2, and 5 mg/kg (mean ± SD, *n* = 3).

In vivo infection models vary significantly in their pathophysiology and severity. We initially demonstrated activity of MyxA in the mouse model of *P. multocida* sepsis. Mice were treated with single doses of 10 mg/kg (i.p.) or 50 mg/kg (s.c.) MyxA 1 h post-infection. Strikingly, although blood levels of MyxA at 30 min and 2 h after administration did not exceed in vitro MICs, the compound was highly effective in reducing the bacterial burden in liver and significantly increasing survival rates when administered i.p. (Figure 2a). Interestingly, although blood levels after s.c. administration of 50 mg/kg were higher than in the group receiving the 10 mg/kg i.p. dose, the latter treatment was much more successful and bacteriological cure was achieved for five out of six animals. In fact, only marginal effects on bacterial burden and no improvement in 48 h survival compared to the vehicle group were found in animals receiving 50 mg/kg (s.c.). Given that sufficiently high blood levels were obtained, this might point toward a significant contribution of MyxA toxicity at this dose to observed adverse effects and mortality.

Next, we investigated pharmacokinetics in mice receiving i.p. doses of 0.5, 2, and 5 mg/kg/day on 7 consecutive days in order to select a dosing scheme for the significantly more challenging 4 week model of *M. tuberculosis* infection. Although the single 10 mg/kg i.p. dose was well tolerated (no adverse effects according to clinical scoring) in the *P. multocida* sepsis model, we observed severe adverse effects in the highest dose group (5 mg/kg/day) on day 2. However, seven consecutive doses of 0.5 mg/kg/day and 2 mg/kg/day were well tolerated.

Based on the PK results (Figure 2b), it seems plausible that the toxic effect of multiple 5 mg/kg doses is caused by an accumulative effect due to the long half-life of MyxA.

To achieve first insights related to in vivo efficacy of MyxA for chemotherapy of tuberculosis during the acute stage of infection, C57Bl/6 mice were aerosol-infected with ∼300 *M. tuberculosis* bacilli and daily treatment at 2 mg/kg (i.p.) initiated after 10 days. Unfortunately, the MyxA-treated group became moribund after 1 week of daily treatment and the study had to be terminated. Based on organ burden enumeration in lung and spleen, no significant effect on bacterial burden was observed (Figure S1-3). The non-curing and toxic effects of MyxA in this mouse model can be possibly explained by two factors. First, although blood levels are in the range of in vitro MIC against *M. tuberculosis*, MyxA might not reach infected tissue sites at sufficient levels. Second, the low drug clearance and observed in vivo toxicity at doses >2 mg/kg/day in combination with the pathophysiology of *M. tuberculosis* infection might potentiate overall toxicity as observed after 1 week in the mouse model of tuberculosis. Nevertheless, both issues could be addressed by modification of the MyxA scaffold following the established total synthetic route with the overall goal to reduce off-target toxicity and improve (oral) bioavailability and lung exposure.

The in vitro results reignited our interest in the Myx scaffold. We therefore set out to ensure sufficient compound supply for follow-up studies by implementing a dual strategy comprising both the myxobacterial source and the development of a route to total synthesis.

### Biosynthesis, Biotechnological Production Optimization, and Structural Revision

**Figure 3:**
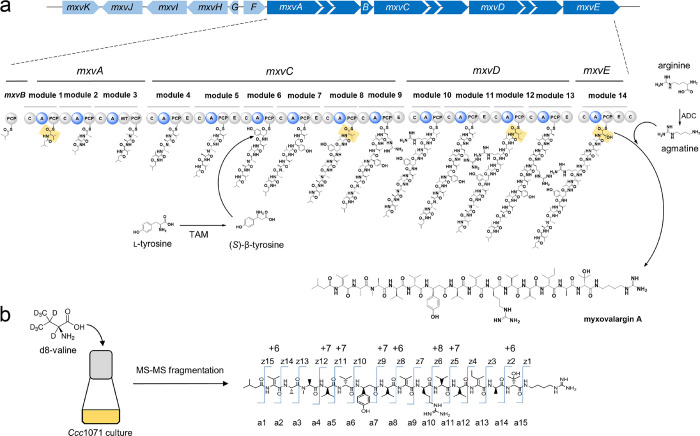
Myxovalargin biosynthesis and feeding study. (a) Architecture of the native MXV BGC from producer strain *Corallococcus coralloides* 1071 (GenBank accession number OQ092403) and model for MXV biosynthesis. The five NRPS genes *mxvABCDE* constitute most of the 66 kbp long MXV BGC. MxvB incorporates the starter unit (isovaleryl in case for MXV A). Module 6 incorporates (*S*)-β-tyrosine, which is derived from l-tyrosine by a tyrosine aminomutase (TAM/MxvJ). The α,β-dehydro-amino acids and the hydroxy valine (orange highlighted squares) are proposed to be formed by MxvH. The final agmatine residue is derived from arginine by an arginine decarboxylase (ADC) and hypothesized to be incorporated by the terminal C domain, through which the molecule gets released from the assembly line. PCP = peptidyl carrier protein; C = condensation domain; A = adenylation domain; MT = methyltransferase domain; E = epimerization domain. (b) Feeding experiment with subsequent MS/MS fragmentation analysis to investigate incorporation of building blocks into myxovalargins.

For the first approach, we identified accessible myxovalargin producers through a search across 2500 LC–MS datasets from a previous secondary metabolome study.^13^ Among the small number of candidates, strain MCy6431–by 16S rRNA sequence comparison likely a species of the genus *Pyxidicoccus*–showed more favorable production and growth characteristics when compared to the literature-reported producer Mx f65 and was also amenable to genetic manipulation. Strain MCy6431 was thus subjected to media optimization and up-scaling of fermentation to facilitate myxovalargin production (see the SI). Furthermore, its 13.2 Mbp genome sequence was determined to enable elucidation of the biosynthetic pathway for myxovalargin. The myxovalargin scaffold comprises mainly non-proteinogenic amino acid building blocks including several d-valine, d-alanine, and d-arginine as well as (*S*)-β-tyrosine, dehydro-valine, dehydro-isoleucine, and hydroxy-valine moieties and was therefore assumed as the product of a nonribosomal peptide synthetase (NRPS)-type biosynthesis route.^14^ Targeted gene inactivation connected the compound class to a large biosynthetic gene cluster (BGC) spanning some 70 kbps and comprising at least two operons, with core biosynthesis functions encoded by genes *mxvA*–*mxvE*. The number and type of domains encoded within the *mxv* biosynthetic locus as well as the biochemical characteristics of individual domains predicted through detailed bioinformatic analysis largely agree with a model for the NRPS-directed biosynthesis of myxovalargins as shown in Figure 3a (see also the SI). A tyrosine 2,3-aminomutase supplying the striking (*S*)-β-tyrosine building block is encoded by *mxvJ*, which is a homologue of the aminomutase-encoding gene characterized from *Myxococcus* in an earlier report,^15^ and its role in Myx biosynthesis was confirmed by targeted gene inactivation and complementation through supplementation of β-tyrosine (SI). Since the Myx assembly line does not employ a type-1 thioesterase (*mxvF* instead encodes a type-2 thioesterase with proofreading function only), Myx biosynthesis intriguingly terminates with a C-domain catalyzed incorporation of agmatine, as underpinned by our feeding study (see the SI). The finding and positioning of D-configured amino acids in Myxs agrees widely with the presence of integral epimerase (E) domains encoded on *mxvA*, *mxvC*, *mxvD*, and *mxvE* as well as with the respective downstream C-domain classification into LCL or DCL types,^16^ however, with two remarkable exceptions: Module 7 contains an E domain, which suggests d-valine at the respective position in the peptide, while module 10 lacks an E domain, which is contrary to the d-valine assigned to this position in the published structure of MyxA.^7^ This stereochemical discrepancy could not be rationalized in light of the assumed colinear NRPS-based architecture of Myx biosynthesis and therefore prompted us to first verify the genetic information, which was performed by resequencing and Southern blotting (see the SI). Our observations ultimately led us to challenge the original stereochemical assignment at these positions.

Consequently, we carried out an independent determination of Myx configuration using a precursor feeding approach coupled with mass spectrometry (see the SI). The outcome of our MS/MS-based analysis of labeled fragment ions (Figure 3b) unambiguously confirmed the altered stereochemical assignment of myxovalargin building blocks as d-valine incorporated by module 7 and l-valine for module 10. We thus show the revised configuration throughout this article, and the structural revision also informed the total synthesis approach.

### Total Synthesis and Verification of the Structural Revision

**Figure 4:**
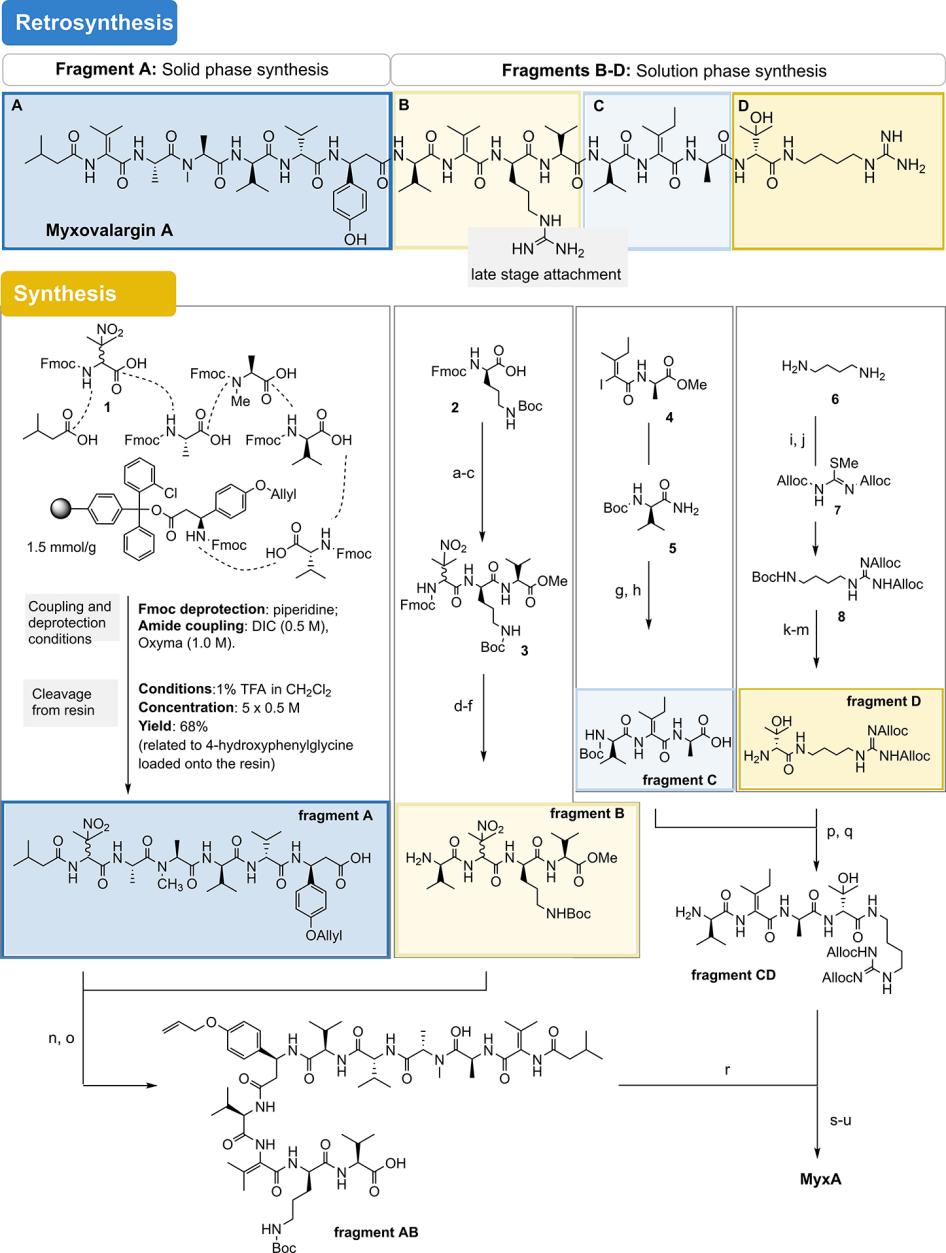
Retrosynthetic analysis of MyxA and principal methods employed for fragment synthesis. Boc = *tert*-butyloxycarbonyl, DIPEA = di*iso*propylethylamine, Fmoc = fluorenylmethoxycarbonyl, DIC = di*iso*propylcarbodiimide, Oxyma = ethyl cyanohydroxyiminoacetate, Alloc = allyloxycarbonyl, EDC = 1-ethyl-3-(3-dimethylaminopropyl)carbodiimide, HOAt = 1-hydroxy-7-azabenzotriazole, TBAF = tetra-*n*-butylammonium fluoride, TFA = trifluoroacetic acid; PyAOP = (7-azabenzotriazol-1-yloxy)tripyrrolidinophosphonium hexafluorophosphate, HATU = *O*-(7-Azabenzotriazol-1-yl)-*N*,*N*,*N*′,*N*′-tetramethyluronium-hexafluorphosphate. Reagents and conditions: (a) L-Val-OMe·HCl, EDC HCl, HOAt, NaHCO_3_, CH_2_Cl_2_/DMF (5:1), 0 °C to rt, 16 h (98%); (b) (H_2_NCH_2_CH_2_)_3_N, CH_2_Cl_2_, 0 °C to rt, 4 h (99%); (c) **1**, EDC HCl, HOAt, NaHCO_3_, CH_2_Cl_2_/DMF (5:1), 0 °C to rt, 18 h (86%, *d.r*. = 1.5:1); (d) Me_2_NH, DMF, rt, 6 h; (e) **S13**, EDC HCl, HOAt, DIPEA, CH_2_Cl_2_/DMF (5:1), 0 °C to rt, 18 h (79% o2s, *d.r.* = 3:1); (f) TBAF (1 M in THF), THF, rt, 15 h (quant.); (g) **5**, CuI, *N,N*-dimethylcyclohexane-1,2-diamine, K_2_CO_3_, 1,4-dioxane, 70 °C, 20 h (65%); (h) LiOH (1 M in H_2_O), THF, 0 °C to rt, 21 h (quant.); (i) Boc_2_O, 1,4-dioxane, rt, 20 h (89%); (j) **7**, Et_3_N, THF, rt, 72 h (93%); (k) Me_3_Si-I, CH_2_Cl_2_, rt, 5 min; (l) **S20**, EDC HCl, Oxyma, NaHCO_3_, CH_2_Cl_2_/DMF (6:1), 0 °C to rt., 20 h (43%); (m) TFA, 0 °C, 2 h (quant.); (n) HOAt, PyAOP, DIPEA, DMF, 0 °C to rt, 20 h (71%); (o) LiOH (1 M in H_2_O), THF, 0 °C to rt, 4 h (quant.); (p) fragments **C** and **D**, EDC HCl, HOAt, −15 °C, then NaHCO_3_, rt, MeCN, DMF, 20 h (60%); (q) Me_3_Si-I, CH_2_Cl_2_, rt, 10 min (quant.); (r) fragments **AB** and **CD**, HOAt, HATU, DIPEA, DMF, 0 °C to rt, 17 h (55%); (s) Me_3_Si-I, CH_2_Cl_2_, rt, 1 h; (t) **7**, Et_3_N, THF, rt, 16 h; (u) PhSiH_3_ (6 equiv), Pd(PPh_3_)_4_ (0.2 equiv), CH_2_Cl_2_, rt, 2 h (33% o3s).

To substantiate the chemical structure of MyxA, we have developed a total synthesis program for both proposed structures. Initial attempts to synthesize MyxA under solution phase conditions had to be terminated unsuccessfully because epimerization of individual positions of the peptide involved was frequently observed during amide bond formation in which valine was involved, as well as during deprotection steps. An approach to MyxA based solely on solid-phase synthesis was also not possible due to the presence of several dehydro-amino acids. Therefore, a hybrid strategy combining solid-phase chemistry with synthesis in solution was chosen. Our retrosynthesis gave four major fragments A–D. This and the forward syntheses exemplified for the revised structure of MyxA are shown in Figure 4 (for details, see the SI). Based on the experience mentioned above, fragment A should preferably be prepared by microwave-accelerated solid-phase synthesis since we observed epimerizations during its preparation in the solution phase. In contrast, fragments B–D can be prepared in large scale in solution, and the same is true for the final peptide coupling reactions and deprotection steps.

Two different strategies were used for the introduction of the olefinic double bonds. For the generation of dehydrovaline, base-promoted elimination of the nitro group in β-nitrovaline **1** was the method of choice,^17^ while dehydroisoleucine was introduced into the peptide backbone by a copper-mediated Goldberg reaction of acylamide **5** with vinyl iodide **4**.^18^ The synthesis of unusual amino acids such as 4-hydroxyphenyl-β-alanine **S7** and β-nitrovaline **1** as well as vinyl iodide **4** and amidinating agent **7** are described in the Supporting Information. Fragment B was obtained in 66% yield starting from protected d-ornithine **2**. The Goldberg coupling was used in the synthesis of fragment C as key reaction which was achieved in satisfactory yield. Finally, fragment D was prepared from 1,4-diamino-butane **6**, which was coupled with Fmoc-protected hydroxy-valine, and amidinated using the Alloc-protected methyl carbamimidothioate **7** reagent.

The total synthesis was completed by first coupling fragments A and B and fragments C and D. The resulting two larger peptide fragments were then joined, eventually yielding the complete peptide backbone. A notable step was the facile saponification of the methyl ester in peptide fragment AB as it was accompanied by the elimination of the nitro groups and the formation of the two dehydrovalines. Once the complete peptide chain was available, the synthesis was completed by mild removal of the Boc group, amidination with reagent **7**, and global removal of all allyl-bearing protecting groups. It should be noted that the final removals of the protecting groups again faced the hurdles already mentioned as it was again difficult to completely suppress partial epimerizations. This synthetic route was also used to prepare the originally reported structure of MyxA, which is described in the SI. LC–MS analyses of both synthetic products accompanied with NMR studies, as well as comparison with a natural sample of MyxA, confirmed that the structure proposed here is the correct one and that the published one must be revised. It can also be highlighted that the synthesis paves the way for the development of a medicinal chemistry program for myxovalargin.

### Binding Mode and Molecular Interactions of Myxovalargin with the Ribosome

**Figure 5:**
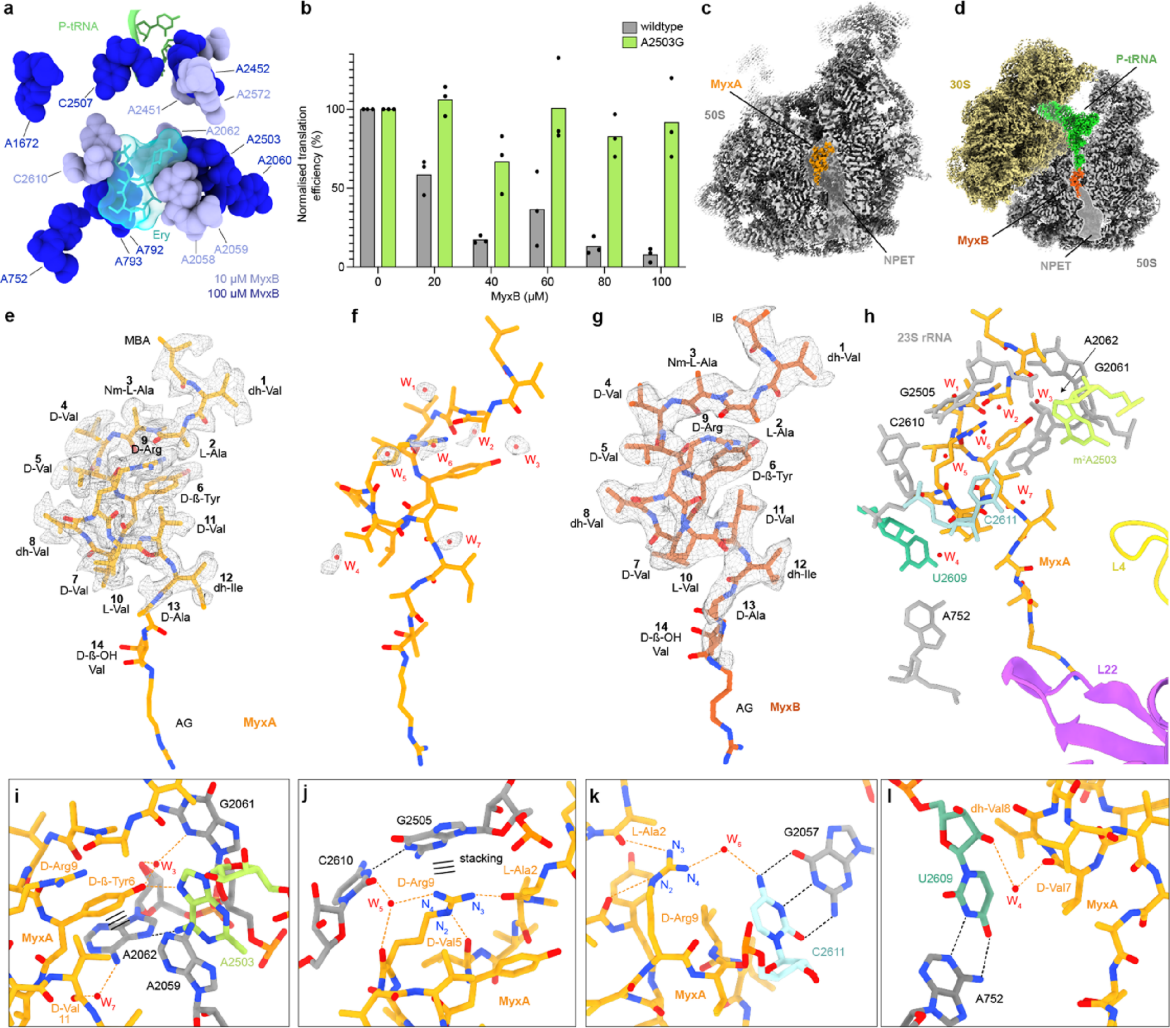
Interaction of Myxovalargin with the *E. coli* ribosome. (a) Relative position of P-site tRNA (green) and erythromycin (Ery, cyan) to 23S rRNA nucleotides protected from DMS by 10 μM (light blue) or 100 μM (dark blue) MyxB. (b) In vitro translation efficiency of wildtype or A2503G mutant ribosomes in the presence of increasing concentrations of MyxB. Values are normalized with 100% defined as the firefly luciferase luminescence observed in the absence of drug. (c, d) Transverse section of the cryo-EM density map of (c) MyxA (orange) bound to the large 50S subunit (gray), and (d) MyxB (red) within the MyxB-SRC, with the nascent peptide exit tunnel (NPET) indicated using shading. (e, f) Molecular model of MyxA (orange) with cryo-EM density map (mesh) for (e) MyxA, and (f) putative water molecules W_1_–W_7_ (red). (g) Molecular model of MyxB (red) with cryo-EM density map (mesh). (h) Overview of the MyxA binding site with MyxA (orange) surrounded by 23S rRNA nucleotides (gray), ribosomal proteins L4 (yellow) and L22 (purple), and putative water molecules W_1_–W_7_ (red). Identified *M. tuberculosis* MyxA^R^ mutations equivalent to *E. coli* positions U2609 (turquoise), C2611 (cyan), and A2503 (lime) are highlighted. (i–l) Interactions of MyxA (orange) with 23S rRNA (gray) and putative water molecules (red). Orange dashed lines indicated potential hydrogen bonds between MyxA and the ribosome, whereas back dashed lines indicate ribosomal intramolecular hydrogen bonding. In (i) and (j), stacking interactions are indicated with three horizontal lines.

The myxovalargin resistance mutations (Table 1) suggest that these peptides inhibit translation by binding to the large ribosomal subunit. We therefore employed DMS-MaPseq and could indeed show that MyxB protects multiple 23S rRNA nucleotides of the large ribosomal subunit from chemical modification by dimethyl sulfate (DMS) (Figure S7-1).^19,20^ The modification sites cluster around the erythromycin binding site within the nascent polypeptide exit tunnel (NPET) (Figure 5a) and include A2503, which is equivalent to the mutation site in *M. tuberculosis* (A2741G) that confers MyxA resistance (Table 1). To demonstrate that the single A2503G mutation in *E. coli* is sufficient to confer myxovalargin resistance, we employed an *E. coli* strain bearing the A2503G mutation.^21^ Because Gram-negative bacteria, such as *E. coli*, are naturally resistant to myxovalargin, it was necessary to isolate the mutated 70S ribosomes and monitor the translation activity of purified wildtype and mutant ribosomes in the absence and presence of myxovalargin in vitro. As shown in Figure 5b, in vitro translation of the firefly luciferase reporter by wildtype *E. coli* ribosomes was inhibited by MyxB in a dose-dependent fashion, consistent with previous reports.^8^ By contrast, *E. coli* ribosomes bearing the A2503G mutation were completely resistant to MyxB even at the highest concentration tested (100 μM). This supports the suggestion that 23S rRNA mutations, rather than the other SNPs, observed in the *M. tuberculosis* MyxA^R^ strains (Table 1) are likely to be responsible for resistance phenotype.

To visualize the interactions of myxovalargins with the ribosome, we determined cryo-EM structures of MyxA in complex with the vacant *E. coli* 50S subunit at 2.10 Å (Figure 5c, Figures S7-2 and S7-3 and Table S7-1) as well as a MyxB-stalled ribosome complex (MyxB-SRC), at 2.96 Å (Figure 5d, Figure S7-4 to S7-6 and Table S7-1). In both cases, additional density was observed within the NPET that could be unambiguously assigned to MyxA (Figure 5e,f and Figure S7-7) and MyxB, respectively (Figure 5g). While the cryo-EM density allowed the majority of MyxA to be modeled (Figure 5e and Figure S7-7a–f), the D-Ala13, D-ß-OH-Val14, and terminal AG moieties were less well-resolved and observed only at lower map thresholds (Figure S7-7i–k). We also observed density that we attributed to seven water molecules (W_1_–W_7_) (Figure 5f and Figure S7-7c–f). The density for MyxA was consistent with the stereochemistry of Val7 and Val10 having D- and L-configurations (Figure S7-7l), as determined here (Figure 3), rather than L- and D-configurations, as suggested previously.^6,8,18^ Despite the lower resolution, the cryo-EM density of MyxB-SRC was well-resolved and suggested an analogous mode of interaction with the ribosome as MyxA (Figure 5g), consistent with the high conservation in their chemical structures (Figure 3).

The overall conformation of myxovalargins on the ribosome is oriented with the MBA moiety positioned at the PTC and the AG extending down the NPET toward ribosomal protein L22 (Figure 5h). The central region of MyxA is highly compacted, which is likely driven by stacking interactions from the sidechains of D-ß-Tyr6 and D-Arg9 of MyxA with the nucleobases of A2062 and G2505, respectively, of the 23S rRNA (Figure 5i,j and Figure S7-8). The compacted conformation is further stabilized by intramolecular interactions between D-Arg9 and backbone carbonyls of L-Ala2 and D-Val5 of MyxA (Figure 5j). In addition, the sidechain OH group of D-ß-Tyr6 can hydrogen bond with the N7 of m^2^A2503 (Figure 5i). This latter interaction, together with a potential hydrogen bond from the backbone carboxyl of dh-Val1 to the N2 of G2061 (Figure S7-8), represent the only direct hydrogen bonds that can be formed between MyxA and the rRNA, whereas we predict at least nine additional hydrogen bonds between MyxA and rRNA that are mediated via the water molecules W_1_-W_7_ (Figure S7-8). Unsurprisingly, given the highly hydrophobic nature of most of the MyxA sidechains (Ala, Val, and Ile), the majority of the water-mediated interactions involve the backbone carbonyls of MyxA, with the exceptions including the polar sidechains of D-ß-Tyr6 (with W_3_) (Figure 5i) and D-Arg9 (with W_5_ and W_6_) (Figure 5j,k). Two waters (W_3_ and W_5_) are present on the ribosome in the absence of MyxA, whereas the rest become stabilized by MyxA binding (Figure S7-9a,b).^22^ The high structural conservation of the PTC and NPET between *M. tuberculosis* and *E. coli* 70S ribosomes (Figure S7-9c), suggests that MyxA is likely to bind to *M. tuberculosis* ribosomes in a similar fashion to that observed here for *E. coli.*(23) The lower activity of Myx against eukaryotic (wheat germ and rabbit) ribosomes reported previously^8^ may result from sequence as well as conformational differences in the rRNA (Figure S7-9d). Similarly, the MyxA resistance mutations identified within the rRNA of *M. tuberculosis* all map directly within the binding site of MyxA on the *E. coli* 50S subunit (Figure 4h). Mutations at position A2503G (MtuA2741G), U2609C (MtuU2847C), and C2611G (MtuU2849G) are predicted to cause conformational changes in the rRNA nucleotides that abolish direct and water-mediated interactions with MyxA (Figure 5i,k,l and Figure S7-10).

### Mechanism of Action of Myxovalargin to Inhibit Translation

**Figure 6:**
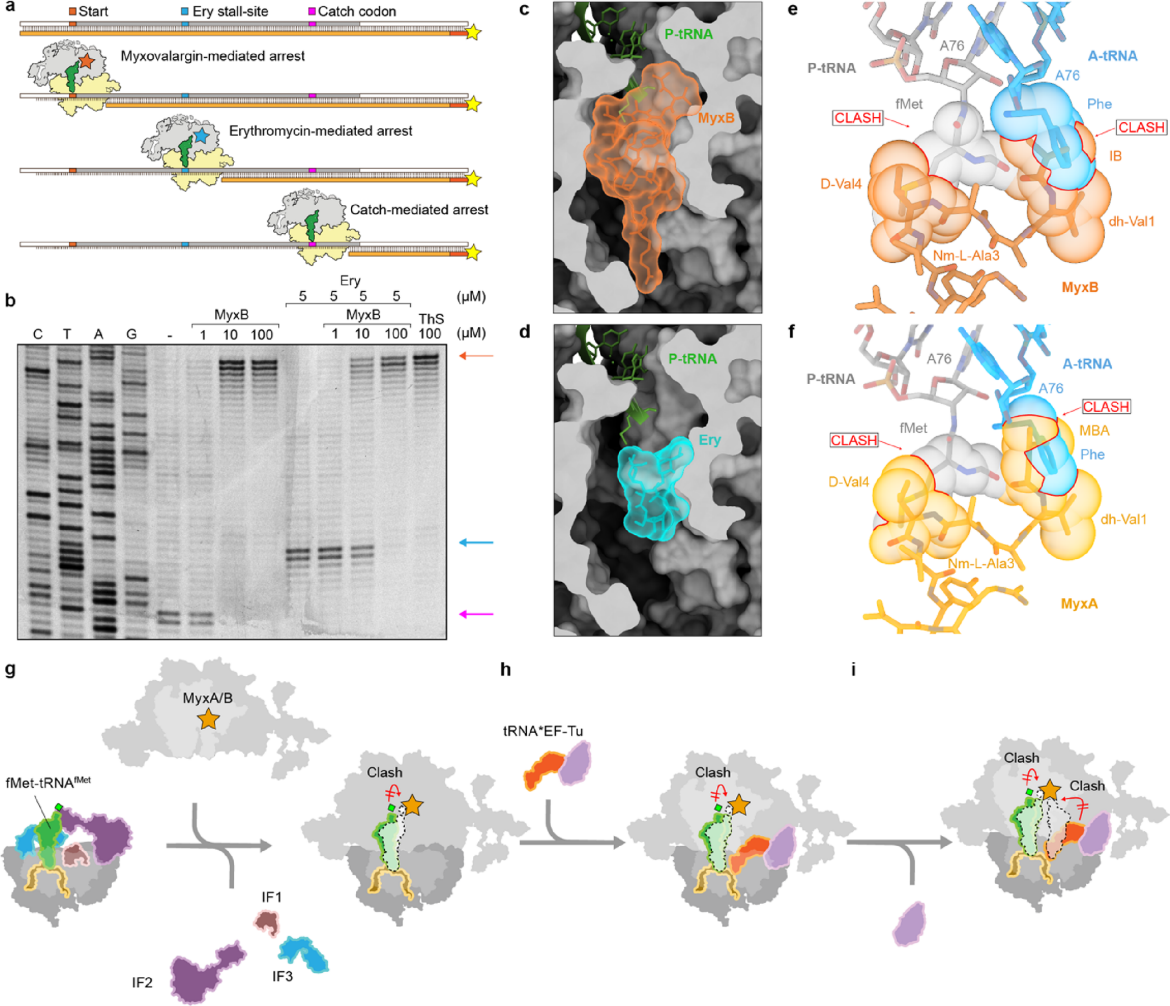
Mechanism of action of myxovalargin to inhibit protein synthesis. (a) Scheme to show the MyxB-mediated (red), Ery-mediated (cyan) and catch-codon mediated (pink) arrest and reverse transcriptase product (yellow). (b) Toeprinting assay performed using mRNA encoding ErmBL leader peptide in the absence (−) and presence of increasing MyxB concentrations (1, 10, and 100 μM) both with and without preincubation of 5 μM Ery and 100 μM ThS as comparison. Sequencing lanes C, T, A, G and arrows indicating the translational arrest of MyxB (red), Ery (cyan), and catch-codon (pink). (c, d) Transverse section of the NPET shown as surface (gray) with P-tRNA (green) and surface representations of (c) MyxB (red) or (d) erythromycin (Ery, cyan). (e, f) Superimposition of the binding site of (e) MyxB (red) or (f) MyxA (orange), with the CCA-ends of initiator fMet-tRNA^fMet^ in the P-site (gray)^28^ and Phe-tRNA^Phe^ in the A-site (cyan),^28^ with sterically clashing atoms indicated using the sphere representation. (g–i) Scheme illustrating the proposed mechanism of action of myxovalargins to inhibit translation. (g) 30S initiation complex, with bound initiation factors (IF) 1 (brown), IF 2 (purple), IF 3 (cyan), mRNA (yellow), and initiator fMet-tRNA^fMet^ (green), joining with large 50S ribosomal subunit, with MyxB (red) bound within the NPET through release of IF 1–IF 3. Positioning of MyxB in the NPET leads to misplacement of initiator tRNA. (h) Delivery of amino acylated-tRNA (orange) by EF-Tu (light purple) to the ribosomal A-site. (i) Accommodation of A-site tRNA in the PTC is blocked due to sterical hinderance of MyxB, arresting translation right after initiation.

To address the mechanism of action of myxovalargins, we employed the toeprinting assay, which uses reverse transcription to monitor the position of ribosomes on a mRNA (Figure 6a).^24^ The assay was performed in the absence of isoleucine (Ile) such that in the absence of antibiotics, ribosomes can initiate at the AUG start codon but become stuck after translating 15 amino acids when the Ile “catch-codon” enters the A-site (Figure 6a,b). By contrast, the presence of increasing concentrations (10–100 μM) of MyxB led to a loss of the ribosomes at the Ile catch codon, and instead, a new band appeared corresponding to ribosomes positioned with the AUG start codon in the P-site (Figure 6a,b), similar to that observed when the control antibiotic thiostrepton was used (Figure 6b). We also performed toeprinting reactions in the presence of 5 μM erythromycin (Ery), which allows ribosomes to translate the first 10 amino acids of the ErmBL mRNA but then stalls them with GAU (Asp) codon in the P-site (Figure 6a,b), as reported previously.^25−27^ After pre-incubation with Ery, we titrated reactions with increasing MyxB, which also led to a loss of the Ery-dependent ErmBL stalling band and appearance of ribosomes positioned on the start codon (Figure 6b), consistent with the overlap in ribosomal binding sites of MyxB and Ery (Figure 6c,d). Collectively, our toeprinting results are consistent with the previous finding that MyxB does not prevent binding of the fMet-tRNA at the P-site but rather interferes with binding of aminoacyl-tRNAs to the A-site.^8^ Indeed, in the MyxB-SRC, we observed the simultaneous presence of P-site tRNA and MyxB (Figure 5d) but no ribosomes with additional presence of A-site tRNA (Figure S7-5). However, careful examination of the MyxB-SRC revealed that while the acceptor arm of the P-site tRNA has accommodated on the large subunit, the terminal CCA-end is not fully accommodated at the PTC (Figure S7-6i). Instead, the P-site tRNA is shifted by 1.5 Å when compared to a fully accommodated pre-attack state P-site tRNA (Figure S7-6) and accommodation appears to be prevented due to direct steric clashes with MyxB (Figure 6e), specifically, between the fMet moiety of the initiator tRNA and the IB and D-Val4 of MyxB (Figure 6e).^28^ Indeed, the density for the P-site tRNA in the MyxB-SRC is consistent with an initiator fMet-tRNA; however, the CCA-end of the P-site tRNA is flexible, and thus, there is little to no density observed for the fMet moiety (Figure S7-6g,h). Even if the fMet-tRNA were able to fully accommodate at the P-site, the IB and D-Val1 moieties of MyxB encroach on the A-site and would prevent accommodation of most, if not all, aminoacyl-tRNAs at the PTC (Figure 6e). We note here that the only difference between MyxA and MyxB is the addition of two methyl groups on the IB moiety of MyxA (Figure 3), which we would predict occlude the A-site even further (Figure 6f).

## Discussion

Many antibiotics identified in the past were not further studied and developed for therapeutic use. Reasons include the underappreciation of the challenge of antimicrobial resistance for human therapy some decades ago as well as a lack of economic feasibility of access to natural product scaffolds often exhibiting challenging chemical structures. In addition, our understanding of the global importance of bacterial pathogens has shifted over time and led to the announcement of the WHO priority list of most important species.

In this study, we have re-evaluated the myxobacterial antibiotic myxovalargin and found it to be bactericidal on several important human and animal pathogens at much lower MICs than described and anticipated from previous studies including tuberculosis-causing bacilli. Through identification of an improved natural producer and its fermentation, compound availability was ensured to enable in vivo efficacy and early preclinical studies as reported here. Importantly, by studying the molecular details of Myx biosynthesis, two misassignments in the stereochemistry of the reported structure were identified, which then provided the basis for the development of a concise synthetic route, ultimately verifying the corrected structural assignment. Having the compound in hand also allowed the determination of the ribosome as the molecular target, which was found in a combination of genome sequencing of resistant mutants raised in *M. tuberculosis* and subsequent biochemical and structural analysis of myxovalargin binding to the bacterial ribosome. Although the binding site of myxovalargin on the ribosome overlaps significantly with other antimicrobial peptides, such as Api137,^29^ Bac7,^30,31^ klebsazolicin, and phazolicin,^32,33^ the conformation and interaction mode are quite distinct (Figure S7-11e–h). Unlike other tunnel binding antibiotics, such as Ery and TcmX, the compact conformation of MyxA completely occludes the NPET, in a manner reminiscent of the blockage caused by the synergistic streptogramin dalfopristin and quinupristin combination (Figure S7-11i–l).^34^ Our toeprinting assays and structure of the MyxB-SRC lead us to propose a model for the mechanism of action of myxovalargins, namely, that they trap ribosomes during a late stage of translation initiation by preventing full accommodation of the initiator tRNA at the peptidyl-transferase center on the large ribosomal subunit (Figure 6g–i). MyxA shows sufficient pharmacokinetic properties, and an in vivo efficacy proof-of-concept was provided in a mouse model of sepsis caused by the bovine pathogen *P. multocida.* However, toxicity observed when administering MyxA in a more challenging mouse model of tuberculosis is presumably caused by an unspecified “membrane effect” and currently prevents further development toward tuberculosis treatment. Overall, the observations in the performed mouse models revealed several hurdles that need to be overcome prior to progressing myxovalargins as antibiotic agents for in vivo application. These issues, in particular, reducing toxicity, can now be addressed by modification of the MyxA scaffold based on the molecular understanding of its mode of action following the established total synthesis route or via genetic engineering of biotechnological production.

## Experimental Section

### Mouse Model of *M. tuberculosis*

C57Bl6 female mice were housed in biocontainment racks and maintained in accordance with the Animal Welfare Act, the Guide for the Care and Use of Laboratory Animals, and all applicable regulations, standards, and policies in a fully AAALAC International accredited Animal Biosafety Level (ABSL) 3 vivarium suitable for housing *M. tuberculosis*-infected animals.^35^ All procedures were performed utilizing appropriate anesthetics when needed as listed in the NIAID DIR Animal Care and Use Committee-approved animal study proposal LCIM-4E. Euthanasia methods were consistent with AVMA Guidelines on euthanasia and endpoint criteria listed in the NIAID DIR Animal Care and Use Committee approved animal study proposal LCIM-4E. For additional method details, see the SI.

### Activity of MyxA in a Mouse Model of *P. multocida* sepsis

Mouse infection experiments were approved by the local animal welfare authorities of Rhineland-Palatinate, Germany (23 177-07/G15-4-054). Method details can be found in the SI.

### Pharmacokinetics of MyxA after Multiple Dosing

The pharmacokinetic study was done at a CRO (Pharmacelsus GmbH, Saarbrücken, Germany). Adult male C57Bl6 mice (7 weeks old at delivery) were purchased from Janvier Labs (France). The animals were housed in a separate temperature-controlled room (20–24 °C) and maintained in a 12 h light/12 h dark cycle. Food and water were available ad libitum throughout the duration of the study. All experimental procedures were approved by and conducted in accordance with the regulations of the local Animal Welfare authorities (Landesamt für Gesundheit und Verbraucherschutz, Abteilung Lebensmittel und Veterinärwesen, Saarbrücken, Germany; TV 2.4.2.2 14/2020). Additional method details can be found in the SI.

### Biochemical Analysis of Myxovalargin Interaction with the Ribosome

DMS-MapSeq was performed as previously described.^19,20^ Preparation of mutant ribosomes and analysis using in vitro translation and toeprinting assays were performed as before.^19,27^ MyxA-70S ribosome complexes were generated by incubating *E. coli* 70S ribosomes with 100 μM MyxA. MyxB-SRC were generated using the disome approach, as described previously.^25−27^ Additional method details can be found in the SI.

### Cryo-EM Analysis of the Myxovalargin Ribosome Complexes

*E. coli* MyxA-70S ribosomes and MyxB-SRC were applied to pre-coated Quantifoil holey carbon supported grids using a Vitrobot Mark IV (FEI). Data collection was performed on Titan Krios 300 V TEM equipped with a direct electron detector. Images of single ribosome particles were aligned using MotionCor^36,37^ within RELION^38^ and particles were picked using crYOLO^39^ with default settings and a general JANNI model (https://cryolo.readthedocs.io/en/stable/). Defocus values were determined using CTFFind 4.^40,41^ Images were processed with RELION 3.1.^38^ 3D refinement was done using a 70S *E. coli* ribosome as reference (PDB ID: 7NSO).^42^ After 3D classification, the combined 70S particles were CTF-refined, Bayesian-polished, and 3D-refined, and the average resolution was determined using the “gold-standard” criterion (FSC_0.143_).^38^ The final reconstructions were corrected for the modulation transfer function and sharpened by applying a negative B factor automatically estimated by RELION 3.1.^38^ A high-resolution *E. coli* ribosomal 50S subunit model (PDB ID: 7K00)^22^ was initially rigid-body-fitted using ChimeraX^43^ and manually adjusted in Coot.^44^ The molecular model for MyxA/B was generated using ChemDraw (PerkinElmer Informatics) with the 3D model calculated with restraints from AceDRG^45,46^ implemented in Coot Lidia.^44^ Model refinement was done in Phenix 1.19.2-4158 using metal and structural restraints calculated by Phenix eLBOW^47^ and validated by Phenix comprehensive Cryo-EM validation and MolProbity server^48^ (http://molprobity.biochem.duke.edu/). Additional method details can be found in the SI.

## Supporting Information Available

In-detail description of all utilized microbiological procedures, cultivation and extraction conditions, molecular biology including genetic manipulation protocols, analytical chemistry methods including HPLC and uHPLC–MS methods as well as the synthetic procedures including the solid phase protocol, bioactivity testing protocols, structural biology methods, and *in-silico* analyses on gene and protein level (PDF)
