# Supplementary material for: The Myxobacterial Antibiotic Myxovalargin: Biosynthesis, Structural Revision, Total Synthesis, and Molecular Characterization of Ribosomal Inhibition
Source: J Am Chem Soc. 2023 Jan 5;145(2):851–63. doi: 10.1021/jacs.2c08816 (PMC9853869; doi:10.1021/jacs.2c08816)
Supplement: Supplementary file 1 — ja2c08816_si_001.pdf [file ja2c08816_si_001.pdf]

Supporting Information

The myxobacterial antibiotic myxovalargin: Biosynthesis, structural revision, total synthesis and molecular characterization of ribosomal inhibition

1. **Bioactivity evaluation of myxovalargin A**
2. **Material and Methods used for the cultivation, genetic manipulation and characterization of myxovalargin producers**
3. **Myxovalargin biosynthetic gene cluster**
4. **On the stereochemical configuration of myxovalargin A**
5. **Total synthesis – Experimental data**
6. **Spectra of synthesis products**
7. **Cryo-EM structures**
8. **References**

1. Bioactivity evaluation of myxovalargin A

Methods

Mycobacterial strains, culture conditions and media. The wildtype laboratory strain of *M. tuberculosis* used in this study was H37RvMA[1] and is referred to herein as H37Rv. *M. tuberculosis* strains were cultured in various media, as previously described[2–5]. 7H9/ADC/Tw was prepared by supplementing Middlebrook 7H9 (Difco) with 10% albumin-dextrose-catalase (ADC) enrichment (Difco), 0.2% glycerol, and 0.05%Tween 80. 7H9/Glu/ADC/Tw medium was prepared by substituting 0.2% glycerol with 0.2% glucose. 7H9/Glu/CAS/Tx was prepared by supplementing 7H9 with 0.4% glucose, 0.03% casitone (CAS), 0.081% NaCl, and 0.05% Tyloxapol (Tx). Colony-forming units (CFUs) were enumerated by plating on Middlebrook 7H10 agar with a 7H10 agar base (Difco) supplemented with 10% oleic acid-albumin-dextrose-catalase (OADC) (Difco) and 0.5% glycerol.

Activity of MyxA in *M. tuberculosis in vitro* and *ex vivo*. MIC testing for *M. tuberculosis* strains by broth microdilution assay, and time-kill kinetics *in vitro*[3–5] or *ex vivo* (in THP-1-derived macrophages)[6], were performed as previously described. For the intracellular activity assay, mature macrophages were infected with logarithmic-phase *M. tuberculosis* resuspended in RPMI/FBS media at a MOI of 1:1 for 4 h at 37 °C in the presence of 5% CO_2. Infected macrophages were washed twice with pre-warmed PBS to remove any extracellular bacteria, and then incubated for 7 d in 100 µL RPMI/FBS media supplemented with or without varying concentrations of MyxA at 37°C in the presence of 5% CO_2. The incubation media was removed and replenished every 48 h. Infected macrophages were lysed at days 3 and 7 using 100 µL 0.05% SDS and the released, viable intracellular bacteria were enumerated by plating for CFUs. Suspensions were washed in Middlebrook 7H9 broth (Difco) supplemented with ADC (Difco) prior to resuspension and plating to ensure removal of any residual compound. Bacterial colonies were enumerated following 3-4 weeks incubation at 37°C.

Activity of myxovalargins against other bacterial species. All bacterial indicator strains were handled according to standard procedures and were purchased from the German Collection of Microorganisms and Cell Cultures (DSMZ), the American Type Culture Collection (ATCC), or were part of our internal collection. *E. faecium* was cultured in tryptic soy broth and *M. smegmatis* in ADC-supplemented Middlebrook 7H9 broth. All other strains were cultured in in cation-adjusted Müller-Hinton broth. The bacterial inoculum for testing myxovalargins in standard microbroth dilution according to CLSI guidelines was adjusted to approximately 10^4 colony-forming units (CFU)/mL. Serial dilutions of compounds (0.03 to 64 µg/mL) were prepared in sterile 96-well plates and the bacterial suspension was added. Growth inhibition was assessed after overnight incubation (24 h; 48 h for *M. smegmatis*) at either 30°C or 37°C, and the minimum inhibitory concentration (MIC) was determined as the highest dilution with no visible growth. For bovine isolates *Mannheima haemolytica, Pasteurella multocida, Histophilus somni, Staphylococcus aureus* and *Streptococcus uberis* MICs were determined according to CLSI document VET01-A4. For selected strains, 50% (v/v)

bovine serum was added to cation-adjusted Müller-Hinton broth (CaMHB) to assess serum-dependence of antibacterial activity. For *S. aureus* and *S. uberis* also UTH milk was used as test medium. After overnight incubation in milk, viability was determined visually by the addition of alamar Blue® for 2h prior to the end of the incubation period.

Determination of cytotoxic activity in HepG2. Cytotoxicity was assessed by the alamarBlue® assay (Invitrogen) with human liver HepG2 cells in a 384-well plate format. Cells were incubated with test compound concentrations (0.005 - 99.0 µM) for 24 h in supplemented RPMI1640 medium, then medium was removed and alamarBlue® was added followed by incubation for 4 h. Metabolic formation of the fluorescent resorufin was measured on a plate reader (excitation 550 nm, emission 595 nm). The fluorescence signal is proportional to metabolically active and viable cells. Finally, the cytotoxic dose at 50% viability (CTD_{50}) values were calculated reflecting the test compound concentration that reduced cell viability by 50%. The mean value of 4 replicates is shown.

Isolation and whole-genome sequencing of MyxA-resistant mutants of *M. tuberculosis*. Exponential-phase cultures of *M. tuberculosis* were plated on Middelbrook 7H10 agar (Difco) containing MyxA at 10-fold the MIC observed in 7H9/ADC/Tw. Of 14 colonies that were selected for screening, 9 were identified that demonstrated heritable resistance to MyxA. Four of these strains were analyzed by whole-genome sequencing, as previously described[4], and mutations were identified in three (main text **Table 1**).

Activity of MyxA in a mouse model of *P. multocida* sepsis. Mouse infection experiments were approved by the local animal welfare authorities of Rhineland-Palatinate, Germany (23 177-07/G15-4-054). Groups of 6 BALB/c mice were infected with approximately 400 CFU *Pasteurella multocida* per mouse in 200 µl phosphate-buffered saline (PBS, pH 7.4) via intraperitoneal application. Myxovalargin A was dissolved to 1 mg/ml (i.p. application) with 10% DMSO (w/w) in 25% (w/w) Captisol® in PBS pH 7.4, or 5 mg/ml (s.c. application) with 20% (w/w) DMSO in 10% (w/w) Captisol® in PBS pH 7.4. Doses were applied 1 h after infection at 10 mg/kg (i.p.) or 50 mg/kg (s.c.). Control mice received PBS pH 7.4 or Baytril® (Enrofloxacin) at a dose of 10 mg/kg. Mice were sacrificed upon occurrence of pain symptoms or at the end of the infection period of 48 h. Livers were isolated and assessed for bacterial colonization by homogenization in PBS pH 7.4 buffer and plating on Muller-Hinton agar plates containing 5% sheep blood followed by an over night incubation at 35 °C.

Pharmakokinetics of MyxA after multiple dosing. The pharmacokinetic study was done at a CRO (Pharmacelsus GmbH, Saarbrücken, Germany). Adult male C57Bl6 mice (7 weeks old at delivery) were purchased from Janvier Labs (France). The animals were housed in a separate temperature-controlled room (20-24°C) and maintained in a 12h light/12h dark cycle. Food and water were available ad libitum throughout the duration of the study. All experimental procedures were approved by and conducted in accordance with the regulations of the local Animal Welfare authorities (Landesamt für Gesundheit und Verbraucherschutz, Abteilung Lebensmittel- und Veterinärwesen, Saarbrücken, TV 2.4.2.2 14/2020). MyxA was prepared in 0.9% saline as a clear solution. Applications were conducted once daily on 7 consecutive days in the morning by intraperitoneal gavage at time point t= 0

minutes. The study comprised three groups (n = 5): 0.5, 2, and 5 mg/kg/d. Viability and mortality were observed twice daily. Blood samples were collected from all animals on treatment days 1 to 7 to determine the blood levels of the test items 2 hours post dose. At each of the designated time points 20 µL blood was collected from the tail vein into Li-heparin 20 µL capillaries (Minivette POCT, SARSTEDT). The blood samples were transferred into polypropylene tubes and frozen on dry ice within 1-2 min of sampling. All samples were stored at -20°C until LC-MS analysis. The HPLC system consisted of a U-HPLC pump (Accela) and an AS Open auto sampler (Thermo Fisher Scientific, USA). Mass spectrometry was performed on a Q Exactive (Orbitrap) accurate mass spectrometer equipped with a heated electrospray (H-ESI) interface (Thermo Fisher Scientific, USA) connected to a PC running the standard software Xcalibur 4.0.27.19 and Chromeleon 7.2. Acetonitrile-extracted analytes were separated on a Poroshell, 2.7 µm, 100x3 mm analytical column with a C6-Phenyl, 4x2.0 mm ID precolumn. The HPLC was performed in the gradient mode using acetonitrile + 0.1% FA as organic phase (A) and water + 0.1% FA as aqueous phase (B). For quantification, the ISTD method was applied and the system calibrated using a mathematical model for an accurate best-fit calibration. The concentration levels for calibration were chosen in the range of expected sample concentrations. The lower limit of quantification (LLOQ) was set to 1.2 ng/mL blood. The upper limit of calibration was 19200 ng/mL.

Mouse model of *M. tuberculosis*.

Mouse studies were carried out in accordance with the Guide for the Care and Use of Laboratory Animals of the National Institutes of Health under Animal study protocol number LCIM 4E. C57BL/6J mice were infected by the aerosol route as previously described[7]. After 10 days, groups of 10 mice were dosed daily with myxovalargin given intraperitoneally at 2 mg/kg in a total volume of 0.2 mL per mouse, vehicle (sterile saline) control dosed intraperitoneally at 0.2 mL per mouse or Rifampicin given by oral gavage at 10 mg/kg. The myxovalargin group became moribund within 1 week and the experiment was terminated by euthanizing those mice. Bacterial burden was determined by plating the appropriate dilutions of lung and spleen M7H9 homogenates on 7H11/OADC plates and counting the resulting bacteria colonies after at least 21 days of incubation at 37 C.

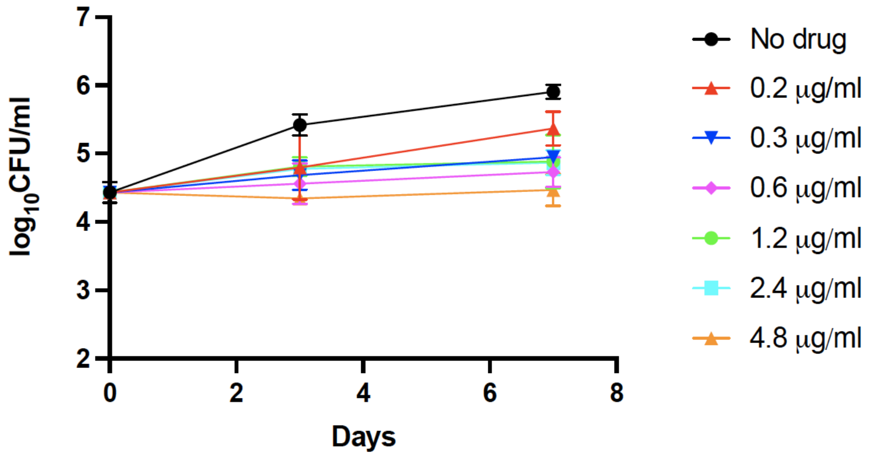

Figure S1-1. **MxyA shows bacteriostatic activity against intracellular *M. tuberculosis* H37Rv.** Limit of detection is 10 bacilli. Data are representative of the mean and SD of independent triplicates.

Table S1-1: Evaluation of *in vitro* activity of natural myxovalargins using a small panel of Gram-positive and Gram-negative bacterial strains.

**bacterial strain**	**MIC [µg/mL]**		
	Myx A	Myx B	Myx C
Chromobacterium violaceum DSM-30191	8	32	8
Enterococcus faecium DSM-20477	0.5	1	1
Escherichia coli DSM-1116	32	64	64
Escherichia coli K12 Δ*tolC*	16	32	32
Micrococcus luteus DSM-1790	1	2	2
Mycobacterium smegmatis mc^2155	4	8	4
Pseudomonas aeruginosa PAO1	32	64	64
Pseudomonas aeruginosa PA14	16	32	32
Staphylococcus aureus DSM-346	2	8	2

Table S1-2: **Minimum inhibitory concentrations (MIC) of MyxA against bovine pathogens.**

**bacterial strain**	**MIC [µg/mL]**
Mannheimia haemolytica MIC_{90} (n = 55)[1]	16 (MIC range: 4 - >16)
Mannheimia haemolytica (n = 2) + serum	20
Pasteurella multocida MIC_{90} (n = 25) [1]	16 (MIC range: 8 - >16)
Pasteurella multocida (n = 2) + serum	10
Histophilas somni MIC_{90} (n = 20) [1]	8 (MIC range: 2-8)
Staphylococcus aureus (n = 6)	1-2
Staphylococcus aureus (n = 6) + milk	> 64
Streptococcus uberis (n = 6)	0.25-2
Streptococcus uberis (n = 6) + milk	> 64

[1]MIC distributions are displayed in Figure S8-2.

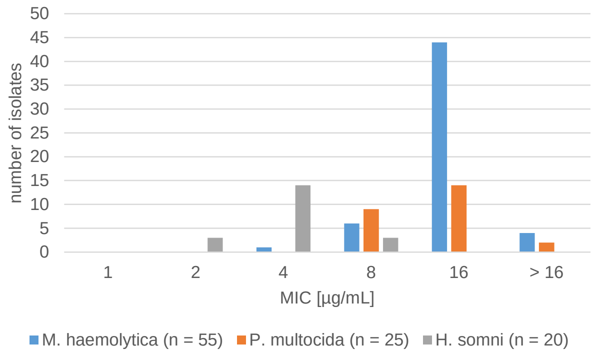

species	n	MIC range	MIC_{50}	MIC_{90}
M. haemolytica	55	4 - >16	16	16
P. multocida	25	8 - >16	16	16
H. somni	20	2-8	4	8

Figure S1-2. **MIC distribution of MyxA among bovine respiratory pathogens.**

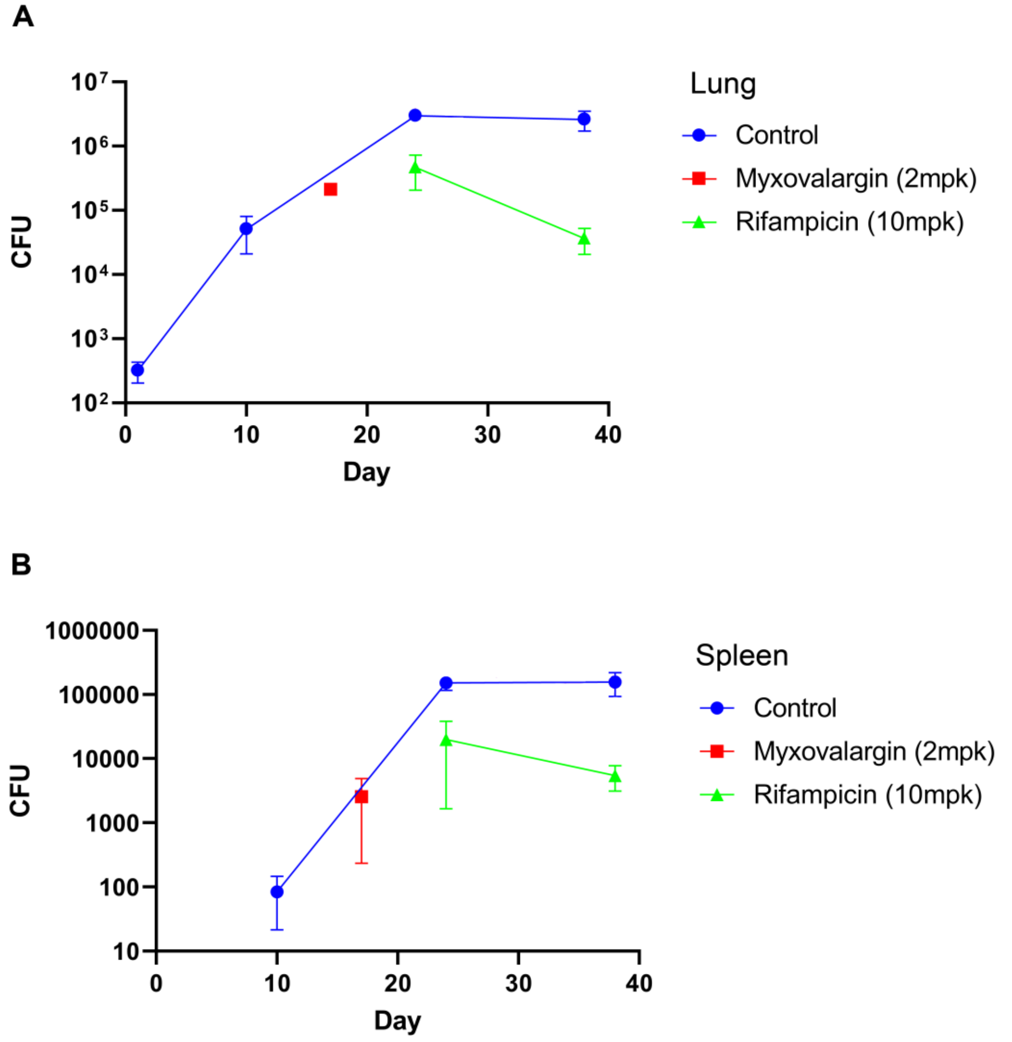

Figure S1-3. **Bacterial burden in lungs (A) and spleens (B) of *M. tuberculosis* infected mice.**

2. Material and methods used for the cultivation, genetic manipulation, and characterization of myxovalargin producers

Strains

Strain	**Short name**	source	purpose
Escherichia coli DH10B	**DH10B**	HIPS-MINS	cloning strain
Myxococcus fulvus 65	**MCy8286 (Mxf65)**	HIPS-MINS	myxovalargin producer
Corallococcus coralloides 1071	**MCy6431 (Ccc1071)**	HZI-MWIS	myxovalargin producer
Unclassified sp. 983 (*formerly Angiococcus* sp. 983)	**MCy5730 (Ang983)**	HZI-MWIS	myxovalargin producer

Isolation of genomic DNA from myxobacteria: Isolation of genomic DNA from myxobacteria for PCR was performed by using the “Gentra Puregene Yeast/ Bact. Kit” by the manufacturer Qiagen Sciences accordingly to the protocol “DNA Purification from Gram-negative Bacteria.

Genomic DNA for plasmid recovery and sequencing was purified by using the following protocol: 50 ml (100 ml) of fresh culture were spun down at 8.000 rpm for 10 min at room temperature. Supernatant was discarded and cells washed once with 15 ml TE buffer and then resuspended in a 15 ml Falcon tube by addition of 6 ml TE buffer. 30 µl Proteinase K solution (20 mg/ml in 50 mM Tris-HCl pH 8.0; 1 mM $CaCl_2$) were added, then inverted several times. With 300 µl 10% SDS the tube was inverted until the mixture became clear. After incubation in the hybridization oven for 2 h at slight rotation, 1 ml 5 M NaCl, then 800 µl of CTAB/NaCl were added and thoroughly mixed. The tube was incubated at 65 °C for 30 min in the hybridization oven.1 volume (= 8 ml) Phenol:Chloroform:Isoamylalcohol (25:24:1) was added and the tube swung for 1 h, then centrifuged at 8.000 rpm for 5 min at room temperature. The upper phase was transferred into a new tube by using an end-cut 1 ml-tip. This step was repeated 3 times. The supernatant was transferred into a new falcon tube by using an end-cut 1 ml-tip. After adding 5 ml Chloroform:Isoamylalcohol (24:1) the tube was swung for 1h, then spun for 10 min at 8000 rpm. 4 ml supernatant was carefully transferred into a new tube using end-cut 1 ml tip. 400 µl of 3 M Na-acetate pH 7.5 were added, then mixed very well by inverting the tube several times. 11 ml of 100% cold ethanol were

poured in and the tube inverted several times, until the appearance of cotton like DNA. The DNA was collected using a Pasteur-pipette by rolling the DNA on the end of the Pasteur-pipette. The pipette-end (with DNA) was immersed into a 2 ml tube containing 70% ethanol for DNA washing. The DNA pellet was dried on the pipette-end by carefully attaching it to the inner site of a fresh 2 ml tube to remove the ethanol drops and dried over night at room temperature. The genomic DNA was suspended in 0.5-1 ml 10 mM Tris-HCl, pH 8.0).

Plasmids and oligonucleotides

Plasmid	Origin	Resistance	Size (bp)	Use	Features
pCR®2.1-TOPO®	Invitrogen	KanR, AmpR	3931	Transformation	lacPromotor
pJet 1.2	Fermentas/ Thermo Scientific	AmpR	2974	Cloning	-
pMycoMarKan	-	KanR, TetR	5957	Transformation	Transposase

Isolation of plasmids: The isolation of plasmid DNA from myxobacteria and *E. coli* was conducted either by using the GeneJET Plasmid Miniprep Kit from Fermentas/ Thermo Scientific or by alkaline lysis according to Sambrook et al.

Primer name	Source	Sequence	Purpose
1071_Mxv8_fwd	Ccc1071	GACTTCGCCCGCAGGCACAAG	Plasmid recovery – single crossover
1071_Mxv8_rev	Ccc1071	CATGACGTACGCCAGCTGCTCC	Plasmid recovery – single crossover
1071_Mxv8_test_fwd	Ccc1071	GACTACCTCAAGTGGCTCTCC	Plasmid recovery – single crossover verification
1071_Mxv8_test_rev	Ccc1071	CGAAGCCCACGTAGTGCTG	Plasmid recovery – single crossover verification
1071_Mxv10_fwd	Ccc1071	GGTTCGGCAACGAGCTCCTTCC	Plasmid recovery – single crossover
1071_mxv10_rev	Ccc1071	CACCACGCCTTCCATGTCATCCAG	Plasmid recovery – single crossover
1071_Mxv10_test_fwd	Ccc1071	CAGGTGCAGGAGTGGAGC	Plasmid recovery – single crossover verification
1071_Mxv10_test_rev	Ccc1071	CCAACGGTGACCTCGGTG	Plasmid recovery – single crossover verification
Ccc_ABfwd	Ccc1071	GAGTCCGCCACCAGCAGGAC	Knockout *mxvH*
Ccc_ABrev	Ccc1071	CTGCGCAACCCGGAGATGCTG	Knockout *mxvH*
Ccc_ABtestFwd	Ccc1071	CGTAGAGCCAGGACACCGGAG	Verification *mxvH*-KO

Ccc_ABtestRev	Ccc1071	GTCCACTCCTGGTACGCGCAC	Verification *mxvH*-KO
ABC1KO_fwd	Ccc1071	GAATTCCTGCTGCACCTGACGCTCGCTC	Knockout *mxvI*
ABC1KO_rev	Ccc1071	AAGCTTGCAACGTCGTCGCCAAGTGGAG	Knockout *mxvI*
ABC1test_fwd	Ccc1071	CCTGGTCCAGCACGATGATGCG	Verification *mxvI*-KO
ABC1test_rev	Ccc1071	CTCGTGCGCATGCCCATCACC	Verification *mxvI*-KO
Ccc_Tam_TestF	Ccc1071	CAGCCCCATGCTGACGATGTGGATCCC	Knockout *mxvJ*
Ccc_Tam_TestR	Ccc1071	CTGCCTCATGAAGGGCTACTCC	Knockout *mxvJ*
CCC_TAMconF	Ccc1071	GTCAACACCGGCTTCGGCGAG	Verification *mxvJ*-KO
CCC_TAMconR	Ccc1071	CTGACGCAGCTTCTCGTGCAC	Verification *mxvJ*-KO
Ccc_Abc_TestF	Ccc1071	GGAGGTGCTGCTGCTGGACGAG	Knockout *mxvK*
Ccc_Abc_TestR	Ccc1071	CGAGCACCAGCAGCCACACGTG	Knockout *mxvK*
ABCconFwd	Ccc1071	CTCCTGTGCACCCAGTCCGTG	Verification *mxvK*-KO
ABCconRev	Ccc1071	ACGTGCCAGTGCGTCAGGGTG	Verification *mxvK*-KO
Adx_fwd	Ccc1071	CATGCGCGTGAAGCTGTCCACG	Knockout *adc*
Adx_rev	Ccc1071	GCAGGTCGCCCAGAATCTCCTG	Knockout *adc*
Adx_test_fwd	Ccc1071	CTCGACAACGAGGACGCGCTC	Verification *adc*-KO
Adx_test_rev	Ccc1071	GGTGATCTTCCCGTTGCGCAGC	Verification *adc*-KO
mxvAKOfwd	Ccc1071	CTGCTCCTGTCCTCACGGGAG	Knockout *mxvA*
mxvAKOrev	Ccc1071	GTCCTCCAGCATCAGCGCCAG	Knockout *mxvA*
mxvAKO1kb_test_fwd	Ccc1071	CAGCTACCGCGACTTCGTGCTG	Verification *mxvA*-KO
mxvAKO1kb_test_rev	Ccc1071	GTGGAGCCGGAGGTGTAGATGAG	Verification *mxvA*-KO
pJET1.2 - forward seq	pJET1.2	CGACTCACTATAGGGAGAGCGGC	Sequencing amplicons
pJET1.2 - reverse seq	pJET1.2	AAGAACATCGATTTTCCATGGCAG	Sequencing amplicons
pTOPOin	pTOPO 2.1	CCTCTAGATGCATGCTCGAGC	test primer for pTOPO Knock-out mutants
pTOPOout	pTOPO 2.1	TTGGTACCGAGCTCGGATCC	test primer for pTOPO Knock-out mutants

Polymerase chain reation (PCR): For amplification with Phusion® High Fidelity Polymerase kit (New England Biolabs) the denaturation temperature of 98°C was chosen and the elongation temperature of 72°C. The annealing temperature was chosen according to the primer sequence or evaluated by gradient PCR. The elongation time was calculated by using 20 sec. for approximately 1 kbp. For Taq polymerase (Fermentas) a denaturation temperature of 95°C was chosen and an elongation temperature of 72°C. The annealing temperature was chosen according to the primer sequence or evaluated by gradient PCR. The elongation time was calculated by using 30 sec. for approximately 1 kbp.

PCR with Phusion® polymerase

H_2O purified: 14 µl
HF buffer: 5 µl
dNTPs: 2 µl
DMSO: 1 µl
Primer fwd. 1 µl

Primer rev. 1 µl
Template 1 µl
Phusion® 0.2 µl

PCR with Taq polymerase
H2O purified:14 µl
MgCl2 buffer: 2.5 µl
Taq buffer + KCl 2.5 µl
dNTPs: 2 µl
DMSO: 1 µl
Primer fwd. 1 µl
Primer rev. 1 µl
Template 1 µl
Taq 0.2

Electrophoresis: Electrophoresis was performed with gels of 0.8% Agarose (Biolabs Agarose) and in gel staining with Roti®Safe. As DNA reference ladder GeneRuler™ 1kb DNA Ladder, GeneRuler™ 1kb Plus DNA Ladder and GeneRuler™ 100 bp by Thermo Scientific were used. Gels were run between 80 to 120 V depending on size of the gel, if not mentioned differently.

Pulsed field gel electrophoresis: The samples were run in gels of 1% Agarose in 0.25 % TBE buffer: 0.25 % TBE in a Biometra Rotaphor System 6.0. The chosen program "2 kb – 800 kb, 24 h" was set to the following parameter: duration: 24 h; temperature: 13°C; interval: 60 sec. to 1sec (log), angle 120°C to 110 °C (lin); voltage 180 V to 120 V (log).

Nucleic acid purification: Nucleic acids were purified by using the NucleoSpin® Gel and PCR cleanup Kit by Machery-Nagel GmbH Co KG, 52355 Düren, Germany.

Southern Blot: Southern Blot was performed by using 1 -10 µg genomic DNA purified according to the genomic DNA isolation protocol described above. The probe was labelled with DIG High prime (Roche) according to manufacturer procedure. Blotting, hybridization, and detection was conducted according to common protocols.

Preparation of competent *E. coli* cells: *E. coli* cells were grown in 500 ml LB medium to an OD_{600} = 0.6-0.8. Then, the cell suspension was centrifuged 12 min at 4°C with 6000 x g. The pellet was washed three times with 10% glycerin, first 100 ml, then twice with 50 ml. The cells were centrifuged each time for 5 min at 4°C with 6000 x g. After the final centrifugation step, cells were resuspended in 2.5 ml 10% glycerin, and finally pipetted as 50 µl aliquots in 1.5 ml Eppendorf tubes. Cells were directly shock-frosted in liquid nitrogen and stored at -80°C.

Transformation of *E. coli*: Competent *E. coli* cells were electroporated at 1250V, 200 Ω, 25 µF in a 1 mm glass cuvette. After electroporation, 250 µl LB medium were added and cells incubated at 37 °C for 1 h. Finally, they were plated on LB agar by supplementation of the appropriate selection marker.

Transformation of myxobacteria: Cells were grown to an OD_{600} = 0.6 – 1.0 in 50 ml medium. 2 ml of the culture were transferred to a 2 ml Eppendorf tube and centrifuged at 8000 rpm for 1 min. The supernatant was discarded, and the cells were once washed with 1 ml purified water and centrifuged again with 8000 rpm. The cells were resuspended in 50 µl purified water and 5 µl plasmid solution (200 – 600 ng/µl) was added. After mixing, the suspension was transferred into a 1 mm glass cuvette and electroporated at 650 V, 200 Ω, 25 µF. 1 ml medium was added and the suspension transferred into a 2 ml Eppendorf tube and incubated on a 30°C shaker. After 6 h regeneration time, the cells were mixed in 10 ml soft-agar and plated on an agar plate, both containing the same selection marker.

Media recipes

Name	Ingredients	amount	pH
AMB modified	Starch, soluble<br>Casitone<br>HEPES<br>$MgSO_4$ x 7 H_2O (Grüssing)<br>K_2HPO_4	5 g/l<br>2.5 g/l<br>1.19 g/l<br>0.5 g/l<br>0.25 g/l	pH 7.0 (KOH)
Soce377-4H	Starch, soluble<br>Yeast extract (BD)<br>$MgSO_4$ x 7 H_2O (Grüssing)<br>HEPES<br>Fe-EDTA<br>$CaCl_2$ x 2 H_2O<br>Glucose monohydrate (Applichem)<br>Fructose<br>K_2HPO_4	3 g/l<br>1.7 g/l<br>1.5 g/l<br>4 g/l<br>8 mg/l<br>0.1 %<br>0.1 %<br>0.1 %<br>0.1 %	pH 7.4
VY/2	Baker's yeast (50g/100mL)<br>$CaCl_2$ x 2 H_2O<br>HEPES<br>Vitamin B12 (sterilfiltered)	10 ml/l<br>1.0 g/l<br>50mM<br>0.5 mg/l	pH 7.2 (KOH)
LB medium	Tryptone<br>NaCl<br>Yeast extract	10 g/l<br>5 g/l<br>5 g/l	-
A-Medium	Glycerol (99,5g/l w/v)<br>Soy flour (degreased) HENSEL<br>Starch soluble (ROTH)<br>Yeast extract (BD)<br>$CaCl_2$ x 2 H_2O<br>$MgSO_4$ x 7 H_2O (Grüssing)<br>HEPES (11.9g/l)<br>Fe-EDTA	4.0 g/l<br>4.0 g/l<br>8.0 g/l<br>2.0 g/l<br>1.0 g/l<br>1.0 g/l<br>50mM<br>8 mg/l	pH 7.4
A/2 –Medium	Glycerol (99,5g/l w/v)<br>Soy flour (degreased) HENSEL<br>Starch soluble (ROTH)<br>Yeast extract (BD)<br>$CaCl_2$ x 2 H_2O<br>$MgSO_4$ x 7 H_2O (Grüssing)<br>HEPES (11.9g/l)<br>Fe-EDTA	2.0 g/l<br>2.0 g/l<br>4.0 g/l<br>2.0 g/l<br>1.0 g/l<br>1.0 g/l<br>50mM<br>8 mg/l	pH 7.4
B-Medium	Starch soluble (ROTH)<br>Glucose monohydrate (Applichem)<br>Soy flour (degreased) HENSEL<br>Probion FM582<br>Peptone (Marcor M)<br>$CaCl_2$ x 2 H_2O<br>$MgSO_4$ x 7 H_2O (Grüssing)<br>HEPES (11.9g/l)<br>Fe-EDTA	8.0 g/l<br>2.0 g/l<br>4.0 g/l<br>0.2 g/l<br>0.2 g/l<br>1.0 g/l<br>1.0 g/l<br>50mM<br>8 mg/l	pH 7.2

CLF-Medium	Fructose<br>Glucose monohydrate (Applichem)<br>Skim milk<br>Yeast extract (BD)<br>$CaCl_2$ x 2 H_2O<br>$MgSO_4$ x 7 H_2O (Grüssing)<br>HEPES (11.9g/l)	4.0 g/l<br>6.0 g/l<br>10.0 g/l<br>2.0 g/l<br>1.0 g/l<br>1.0 g/l<br>50mM	pH 7.0
CLF(sugar free)-Medium	Skim milk<br>Yeast extract (BD)<br>$CaCl_2$ x 2 H_2O<br>$MgSO_4$ x 7 H_2O (Grüssing)<br>HEPES (11.9g/l)	10.0 g/l<br>2.0 g/l<br>1.0 g/l<br>1.0 g/l<br>50mM	pH 7.0
CY-Medium	Casitone (Marcor typ M)<br>Yeast extract (BD)<br>$CaCl_2$ x 2 H_2O<br>HEPES (11.9g/l)	3.0 g/l<br>1.0 g/l<br>1.0 g/l<br>50mM	pH 7.2
CY+H-Medium	Casitone (MBD)<br>Yeast extract (BD)<br>Glucose monohydrate (Applichem)<br>Soy flour (degreased) HENSEL<br>Starch soluble (ROTH)<br>$CaCl_2$ x 2 H_2O<br>$MgSO_4$ x 7 H_2O (Grüssing)<br>Na-Fe-EDTA<br>HEPES (11.9g/l)	1.5 g/l<br>1.5 g/l<br>1.0 g/l<br>1.0 g/l<br>4.0 g/l<br>1.0 g/l<br>0.5 g/l<br>0.004 g/l<br>50mM	pH 7.2
E - Medium	Skim milk<br>Soy flour (degreased) HENSEL<br>Yeast extract (BD)<br>Starch soluble (ROTH)<br>$MgSO_4$ x 7 H_2O (Grüssing)<br>HEPES (11.9g/l)<br>Fe-EDTA<br>Glycerol (99.5% w/v)	4.0 g/l<br>4.0 g/l<br>2.0 g/l<br>10.0 g/l<br>1.0 g/l<br>50mM<br>8 mg/l<br>5.0 g/l	pH 7.4
E/2 – Medium	Skim milk<br>Soy flour (degreased) HENSEL<br>Yeast extract (BD)<br>Starch soluble (ROTH)<br>$MgSO_4$ x 7 H_2O (Grüssing)<br>HEPES (11.9g/l)<br>Fe-EDTA<br>Glycerol (99.5% w/v)	2.0 g/l<br>2.0 g/l<br>1.0 g/l<br>5.0 g/l<br>1.0 g/l<br>50mM<br>8 mg/l<br>2.5 g/l	pH 7.4
H-Medium	Soy flour (degreased) HENSEL<br>Glucose monohydrate (Applichem)<br>Starch soluble (ROTH)<br>Yeast extract (BD)<br>$CaCl_2$ x 2 H_2O<br>$MgSO_4$ x 7 H_2O (Grüssing)<br>HEPES (11.9g/l)<br>Fe-EDTA	2.0 g/l<br>2.0 g/l<br>8.0 g/l<br>2.0 g/l<br>1.0 g/l<br>1.0 g/l<br>50mM<br>8 mg/l	pH 7.4
K-Medium (Gerth)	Dextrose<br>Glucose monohydrate (Applichem)<br>Peptone (Marcor Typ S)	40.0 g/l<br>2.0 g/l<br>2.0 g/l	pH 7.2

	$(NH_4)_2NO_3$<br>KH_2PO_4<br>K_2HPO_4<br>$CaCl_2$ x 2 H_2O<br>$MgSO_4$ x 7 H_2O (Grüssing)<br>HEPES (11.9g/l)<br>Fe-EDTA	1.0 g/l<br>0.9 g/l<br>2.3 g/l<br>1.0 g/l<br>1.0 g/l<br>50mM<br>8 mg/l	
K-Medium (fermentation)	Peptone (Marcor Typ S)<br>Starch, soluble (Roth)<br>Soytone (BD)<br>Phytone (BD)<br>Yeast extract (BD)<br>$CaCl_2$ x 2 H_2O<br>$MgSO_4$ x 7 H_2O (Grüssing)<br>HEPES (23.8g/l)<br>Fe-EDTA	2.0 g/l<br>8.0 g/l<br>4.0 g/l<br>2.0 g/l<br>2.0 g/l<br>1.0 g/l<br>1.0 g/l<br>100mM<br>8 mg/l	pH 7.2
M – Medium	Soytone (BD)<br>Maltose Monohydrate (Roth)<br>$CaCl_2$ x 2 H_2O<br>$MgSO_4$ x 7 H_2O (Grüssing)<br>HEPES (11.9 g/l)<br>Fe-EDTA	10.0 g/l<br>10.0 g/l<br>1.0 g/l<br>1.0 g/l<br>50mM<br>8 mg/l	pH 7.2
M/2-Medium	Soytone (BD)<br>Maltose Monohydrate (Roth)<br>$CaCl_2$ x 2 H_2O<br>$MgSO_4$ x 7 H_2O (Grüssing)<br>HEPES (11.9 g/l)<br>Fe-EDTA	5.0 g/l<br>5.0 g/l<br>1.0 g/l<br>1.0 g/l<br>50mM<br>8 mg/l	pH 7.2
POL - Medium	Probion FM582<br>Starch, soluble (Roth)<br>$CaCl_2$ x 2 H_2O<br>$MgSO_4$ x 7 H_2O (Grüssing)<br>HEPES (11.9g/l)<br>Vitamin B12	3.0 g/l<br>3.0 g/l<br>0.5 g/l<br>2.0 g/l<br>50mM<br>0.5 mg/l	pH 7.2
P - Medium	Peptone (Marcor S)<br>Starch soluble (ROTH)<br>Probion FM582<br>Yeast extract (BD)<br>$CaCl_2$ x 2 H_2O<br>$MgSO_4$ x 7 H_2O (Grüssing)<br>HEPES (23.8g/l)<br>Fe-EDTA	2.0 g/l<br>8.0 g/l<br>4.0 g/l<br>2.0 g/l<br>1.0 g/l<br>1.0 g/l<br>100mM<br>8 mg/l	pH 7.5
S-Medium	Soy flour (degreased) HENSEL<br>Glucose monohydrate (Applichem)<br>Starch soluble (ROTH)<br>$CaCl_2$ x 2 H_2O<br>$MgSO_4$ x 7 H_2O (Grüssing)<br>HEPES (11.9g/l)<br>Fe-EDTA	4.0 g/l<br>2.0 g/l<br>8.0 g/l<br>1.0 g/l<br>1.0 g/l<br>50mM<br>8 mg/l	pH 7.4
S/2+V-Medium	Soy flour (degreased) HENSEL<br>Glucose monohydrate (Applichem)<br>Starch soluble (ROTH)	2.0 g/l<br>1.0 g/l<br>4.0 g/l	pH 7.4

	$CaCl_2$ x 2 H_2O<br>$MgSO_4$ x 7 H_2O (Grüssing)<br>HEPES (11.9g/l)<br>Fe-EDTA<br>Vitamin solution (Schlegel)	1.0 g/l<br>1.0 g/l<br>50mM<br>8 mg/l<br>1 ml/l	
Myxovirescin - Medium	Casein Peptone (Marcor typ M)<br>$CaCl_2$ x 2 H_2O<br>$MgSO_4$ x 7 H_2O (Grüssing)<br>$CoCl_2$<br>HEPES (23.8 g/l)	10.0 g/l<br>0.05 g/l<br>0.1 g/l<br>1 mg/l<br>100mM	pH 7.0
M7 – Medium	Probion FM582<br>Starch, soluble (Roth)<br>Glucose monohydrate (Applichem)<br>Yeast extract (BD)<br>$CaCl_2$ x 2 H_2O<br>$MgSO_4$ x 7 H_2O (Grüssing)<br>HEPES	5.0 g/l<br>5.0 g/l<br>2.0 g/l<br>1.0 g/l<br>1.0 g/l<br>1.0 g/l<br>10.0 g/l	pH 7.4
M7P – Medium (adapted)	Soytone (BD)<br>Phytone (BD)<br>Starch, soluble (Roth)<br>Glucose monohydrate (Applichem)<br>Yeast extract (BD)<br>$CaCl_2$ x 2 H_2O<br>HEPES	2.5 g/l<br>2.5 g/l<br>5.0 g/l<br>2.0 g/l<br>1.0 g/l<br>1.0 g/l<br>10.0 g/l	pH 7.4
MD1 – Medium (modified from Behrens et al. 1976)	Peptone (Marcor M)<br>$CaCl_2$ x 2 H_2O<br>$MgSO_4$ x 7 H_2O (Grüssing)<br>HEPES	3.0 g/l<br>0.5 g/l<br>0.2 g/l<br>10.0 g/l	pH 7.2
AMB medium (Ringel et al., 1977)	Starch, soluble (Roth)<br>Casitone (BD)<br>$MgSO_4$ x 7 H_2O (Grüssing)<br>K_2HPO_4	5.0 g/l<br>2.5 g/l<br>0.5 g/l<br>0.25 g/l	pH 7.0
Vitamin solution 1000x (Schlegel)	Biotin<br>Nicotinsäure<br>Thiamin<br>4-Aminobenzoesäure<br>Pantothenat<br>Pyridoxamin<br>Cyanocobalamin	2.0 g/l<br>20.0 g/l<br>10.0 g/l<br>10.0 g/l<br>5.0 g/l<br>50.0 g/l<br>20.0 g/l	Sterilized by filtration

Media used for growth of myxobacteria: All media components were suspended in highly purified H_2O (Milli-Q Millipore Merck) and then autoclaved. Agar was produced by adding 16 g/l of agar to the according medium before autoclaving; soft-agar by adding 8 g/l agar. Myxobacteria were grown in shake flasks at 30 °C and 200 rpm. Shake flasks were chosen at size of five times the media volume.

Media used for growth of *E. coli* strains: All media components were dissolved in purified H_2O and then autoclaved. Agar of this media was produced by adding 16 g/l of agar before autoclaving. *E. coli* strains were grown in shake flasks at 37 °C and 180 rpm. Shake flasks were chosen at size of five times the media volume.

.

Fermentation: Fermentation was conducted with an Infors HT Labfors 3 equipped with Mettler Toledo oxygen electrodes. 2L and 7.5 L vessels were chosen for cultivation. For process control and evaluation IRIS 5.2 software was used.

Antibiotics used as selection marker in liquid and solid media

Kanamycin 50 mg/ mL in H_2O_{dest} → final concentration 50 µg/ mL

Ampicillin 100 mg/ mL in H_2O_{dest} → final concentration 100 µg/ mL

Analytical HPLC-MS systems

Agilent 1260 LC, Bruker amaZon, Waters Acquity C18 BEH 50x2mm, 1.7 um	40 °C, 0.6 ml/min, A: H_2O, B:ACN, with 0.1% formic acid	linear gradient from 1 to 20 min, 5% to 95% B
Dionex RSLC, LTQ-Orbitrap, NanoMate, Waters Acquity C18 BEH 50x2 mm, 1.7 um	45°C, 0.6 ml/min, A: H_2O, B:ACN, with 0.1% formic acid	linear gradient from 1 to 10 min, 5% to 95% B
Dionex RSLC, LTQ-Orbitrap, NanoMate, Waters Acquity C18 BEH 100x2mm, 1.7 um	45°C, 0.55 ml/min, A: H_2O, B:ACN, with 0.1% formic acid	linear gradient from 1 to 19 min, 5% to 95% B
Dionex RSLC, Bruker maXis 4G, Acquity C18 BEH 50x2mm, 1.7 um	45°C, 0.6 ml/min, A: H_2O, B:ACN, with 0.1% formic acid	linear gradient from 1 to 6 min, 5% to 95% B
Dionex RSLC, Bruker maXis 4G, Acquity C18 BEH 100x2mm, 1.7 um	45°C, 0.6 ml/min, A: H_2O, B:ACN, with 0.1% formic acid	linear gradient from 1 to 19 min, 5% to 95% B

(Semi-)preparative LC systems

Waters Autopurifier UV/ MS; Phenomenex® Gemini C18 250 x 21.20 mm, 5 µm	RT, 25 ml/ min; A:H_2O, B:ACN, with 0.1% formic acid	MassLynx
Dionex Ultimate® 3000; Bruker HCT; Phenomenex	40°C, 0.6 ml/min, A: H_2O, B:ACN, with 0.1% formic acid	Chromeleon

Luna C18, 250 x 4.6 mm, 5 µm		
Biotage® Isolera^{TM} Prime, Biotage® SNAP Cartridge KP-Sil	RT, Flow and run time dependent on cartridge size. A: DCM, B: MeOH + 1% Acetic acid or 0.1 % formic acid	Build-in software

Crude extracts: A 50 ml culture was centrifuged at 8000 rpm and twice extracted with 30 ml methanol for 20 minutes at a stirring rate of 300 rpm. The solvent was evaporated and the residue resuspended in 1 ml MeOH. This procedure was scaled-up or -down according to the culture volume.

Quantification of MXV A production titers in the native producer *Corallococcus coralloides* 1071 (MCy6431)

The MCy6431 strain was transferred from VY/2 agar plates into Erlenmeyer flasks containing 25 mL liquid AMD media until sufficient cell density was reached after three days. Then, 5 mL of the culture was transferred into each of three Erlenmeyer flasks with 50 mL liquid AMD medium. Further, the adsorber resin XAD-16 was added to bind the produced antibiotic. The cell cultures were harvested after six days, once the culture broth took on a brown color.

For analysis of the MXV A production, the cultures were transferred into 50 mL falcon tubes and centrifuged for 10 min at 8000 rpm, supernatant was discarded and cell/XAD-16 pellets stored at -20° C for a couple of hours until they were frozen and then lyophilized overnight. Subsequently, the pellets were extracted twice with 30 mL MeOH for 20 minutes. After extraction, the liquid was filtered and the eluate collected in a round flask. The solvent was then evaporated at a Rotavapor until the extracts were completely dry. Now, the extracts were re-dissolved in 1 mL MeOH and pipetted into glass vials. Before analysis, samples were centrifuged for at least 10 min at 15000 rpm and 10 µL of the supernatants were 10 times diluted in MeOH. An Ultimate 3000 LC System with a Acquity UPLC BEH C-18 column (1.7 µm, 100 x 2 mm), equipped with a VanGuard BEH C-18 (1.7 µm) guard column, was coupled to an Apollo II ESI source and hyphenated to an amaZon speed 3D ion trap mass spectrometer. Separation was performed at a flow rate of 0.6 mL/min (eluent A: deionised H2O + 0.1 % formic acid (FA), eluent B: acetonitrile + 0.1 % FA) at 45 °C using the following gradient: 5 % B for 30 s, followed by a linear gradient up to 95 % B in 18 min and a constant percentage of 95 % B for further 2 min. Original conditions were adjusted with 5 % B within 30 s and kept constant for 1.5 min. The LC flow was split to 75 µL/min before entering the mass spectrometer. Mass spectra were acquired in centroid mode ranging from 150–2,500 m/z at a 2 Hz full scan rate in positive mode. Source parameters were set to 500 V end plate offset, 4000 V capillary voltage, 1 bar nebulizer

gas pressure, 5 L/min dry gas flow and 200 °C dry temperature. Calibration curve was set up with purified MXV A dissolved in MeOH. The trend line of the calibration curve was used to calculate the MXV A concentrations of the samples based on the peak area.

Characterization of myxovalargin producers

Choice of producers: In order to find a suitable myxovalargin producer, screening of myxobacterial LC-MS datasets was performed using a query selective for the mass of myxovalargin A. Besides the known producer *Myxococcus fulvus* Mx f65 [MCy8286], we identified additional strains belonging to the suborder of *Cystobacterinae*: *Corallococcus coralloides* 1071 (Ccc1071) [MCy6431] and *Angiococcus sp.* 983 (Ang983) [MCy5730] [8]. As in the course of this project and due to new taxonomical characterization techniques strain classification of some myxovalargin producers was revised, the MCy naming will be referred to in this document. All strains were evaluated for their suitability in terms of production, application of genetic tools and growth properties. The initial experiments were conducted using Mcy8286 (Mx f65). Therefore, mutagenesis and growth were studied in depth for this strain, although the low frequency of mutants obtained lead to disqualification of this strain for further experiments on the biosynthesis of the myxovalargins. The strain showed fast growth in CTT medium while cells mainly remained planktonic. Random mutagenesis by transposon insertion was possible in high yield Nevertheless, site directed mutagenesis was only achieved with one out of five constructs in several attempts and only a single mutant was generated, which was verified by PCR to be correct. The method established did not yield further mutants with other constructs.

MCy5730 (Ang983) and MCy6431 (Ccc1071) showed similar characteristics regarding their growth. Both occur in nutrient poor VY/2 medium in red, roundish cell colonies of up to approximately 0.5 cm. However, in AMB medium suspended, cultivation is obtained to high cell density (OD_{600} > 2.0). Random mutagenesis was successful in both strains; nevertheless, single crossover recombination was only achieved in MCy6431. It has to be noted that site directed mutagenesis has not been studied in depth for MCy5730, hence this strain is a putative alternative candidate for biotechnological modification. The production levels of myxovalargin compared relatively against the wildtype, showed a superiority of MCy6431 and MCy5730 over the *Myxococci*. The strain MCy6431 was chosen as feasible producer for further development. A reliable mutasynthesis method was established (Figure), furthermore growth conditions were optimized, and production transferred to fermentation scale. **Yield:** Myxovalargin A yield from strain MCy6431 was determined as 23.9 mg/L from a shaking flask fermentation using the LC-MS method described above.

In addition, *Myxococcus sp.* 171 (MCy9171) as myxovvalargin producer was identified in a late stage of this project. The strain shows fast growth in AMB and CTT medium. Mutagenetic tools were not applied to this strain. The production seems to be lower than achieved with MCy5730 and MCy6431. This strain was used for reverse feeding experiments due to its growth in nutrient poor medium[8].

A

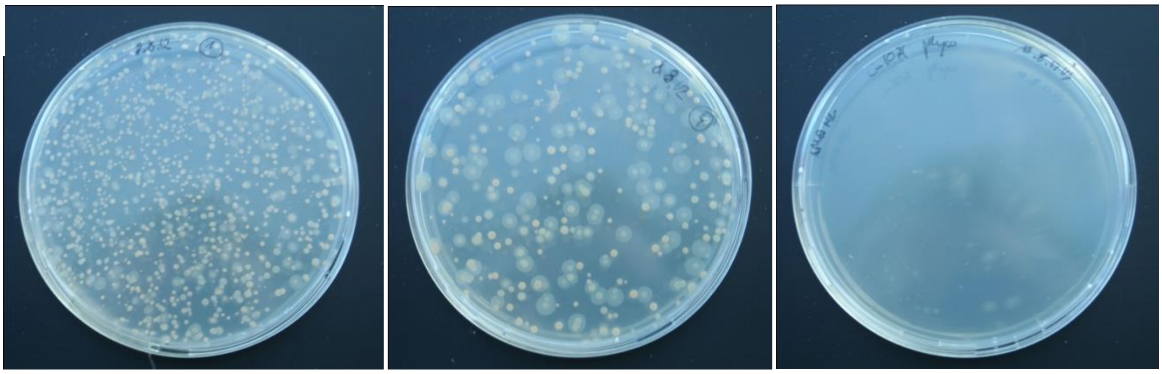

B

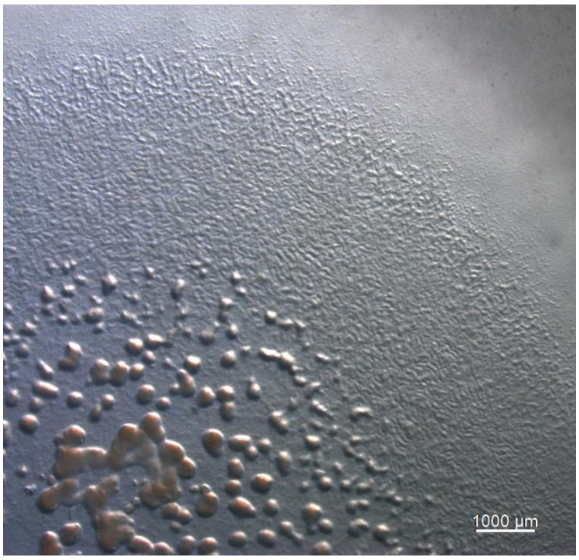

Figure S2-1: A, Study of different mutagenesis procedures for MCy6431 by transposon mutagenesis with pMycoMarKan on AMB agar plus 50 µl/ml kanamycin; left panel: 2 ml cells of liquid culture were centrifuged, washed once, resuspended in 50 µl, electroporated and plated out in soft agar; mid panel: cells from agar plate were scratched off and resuspended in 1ml purified water, washed once, resuspended, electroporated and plated out in soft agar; right panel: cells from liquid culture as prepared in left panel were plated out after electroporation with a Drigalski spatula. **B,** srain MCy6431 growing on VY2 agar. The colony shows formation of fruiting bodies in the left lower corner. Around the fruiting bodies, swarming cells of MCy6431 are visible.

Table S2-1: Comparison of microbiological and biotechnological parameters of different myxovalargin producers regarding time span to reach end of exponential growth by comparable cultural conditions, liquid growth characteristics, production of myxovalargin A and applicability of mutagenetic tools.

Strain	Appr. time in liquid until stationary phase	Liquid growth characteristics	Production of Myxovalargin A	Mutagenesis
MCy8286	TS (2d)<br><br>CTT (2-3d)<br><br>AMB (3-4d)	planktonic<br><br>planktonic<br><br>planktonic	+<br><br>+<br><br>+	Transposon +++<br><br>Site directed +
MCy6431	AMB (4d)<br><br>VY/2 (>4d)	planktonic<br><br>aggregated	++<br><br>+	Transposon +++<br><br>Site directed +++
MCy5730	AMB (4d)<br><br>VY/2 (>4d)	planktonic<br><br>aggregated	++<br><br>+	Transposon ++<br><br>Site directed -
MCy9171	CTT (4d)<br><br>AMB (4d)	planktonic	+<br><br>+	Not evaluated

Characterization of strain MCy6431

Taxonomy: Formerly named as *Corallococcus coralloides* 1071 (Ccc1071), strain MCy6431 in this study was the most promising candidate for further optimization of biotechnological manipulation, but also for production enhancement, and was thus further studied and characterized. Due to its production profile and its genetic accessibility the former classification of this strain in the family of Myxococcaceae[9] was questioned. 16S rRNA comparison in cooperation with Dr. Ronald Garcia revealed a close relationship to *Pyxidococcus fallax* DSM14698^{T} [10], rather than the *Corallococci* (**Figure S1**). This clade is also closely related to the Myxococci, which exhibit a similar production profile. The myxoprincomides[11] are predominantly found in *Myxococcus* strains as well as the myxovalargins, which are produced by four additional *Myxococci*. Also, 16S rRNA of the unclassified strain *Angiococcus* sp. 983 (MCy5730) groups with the *Pyxidicocci*, as well as *Myxococcus* sp.171 (MCy9171), which correlates with similar growth characteristics like MCy6431. Further investigation of morphology and fatty acid profile will shed light on a correct taxonomic classification.

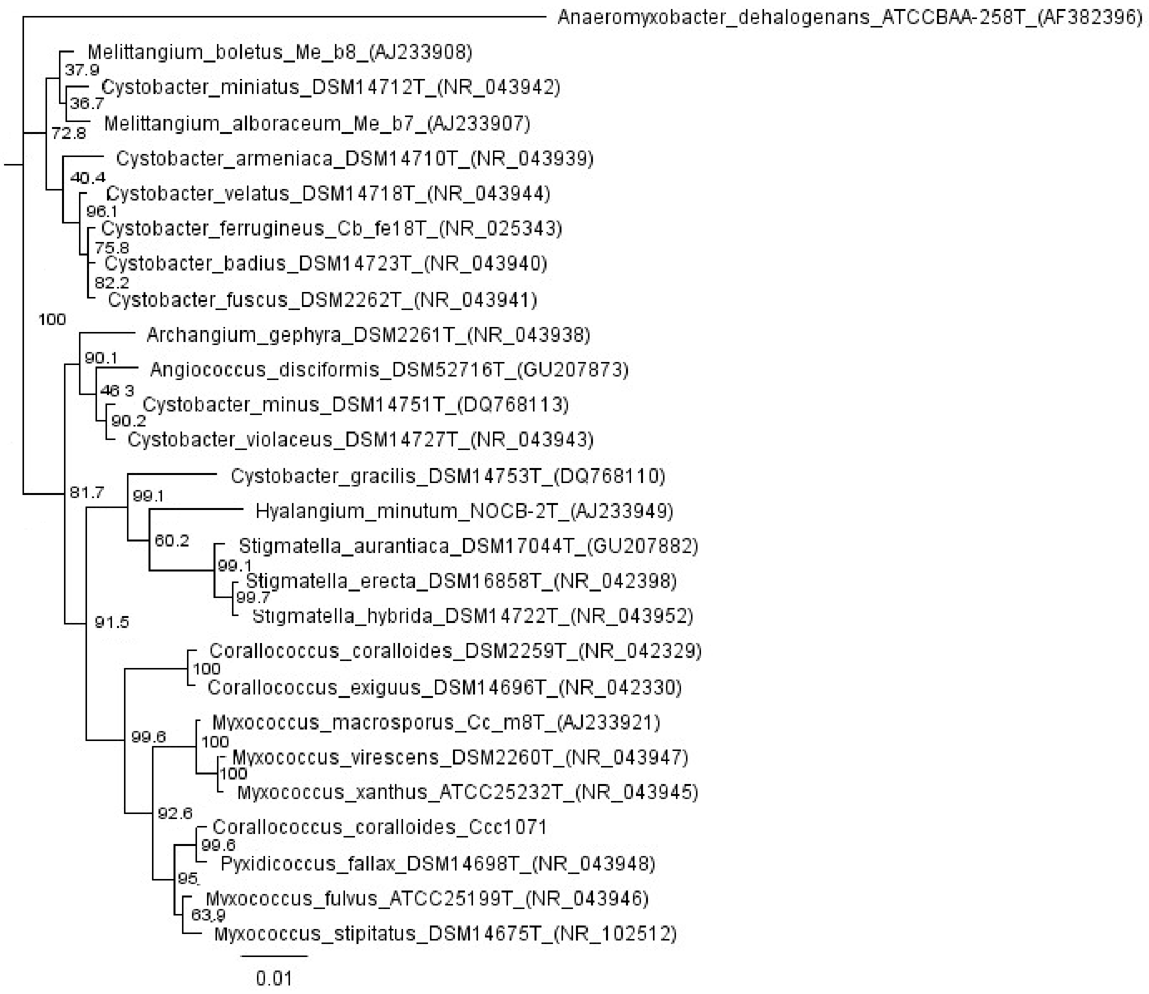

Figure S1-2: 16S rRNA comparison of the strain MCy6431 (Ccc1071), formerly characterized as *Corallococcus coralloides* strain. The comparison supports the assignment of MCy6431 (Ccc1071) to the group of *Pyxidicocci*, instead of the *Corallococci*.

Genome sequencing

The Ccc1071 strain was sequenced by Illumina, Roche 454 and Pacific Bioscience (Pacbio) at the DSMZ, Braunschweig/Germany. For the latter, in house protocols for myxobacteria were combined with commonly used techniques to yield high quality genomic DNA. Quality of genomic DNA was assessed by pulsed field gel electrophoresis. Comparative assembly of Pacbio data was performed by combining Pacbio and Illumina data and by using solely Pacbio scaffolds. This comparison was conducted due to putative miss-assembly and repetitive regions in the myxovalargin cluster. Nevertheless, both sequences showed an equal circular genome. The sequence was optimized assembled resulting in a 13,193,010 bp genome without gaps in the nucleic acid sequence **(Figure S2-2).**

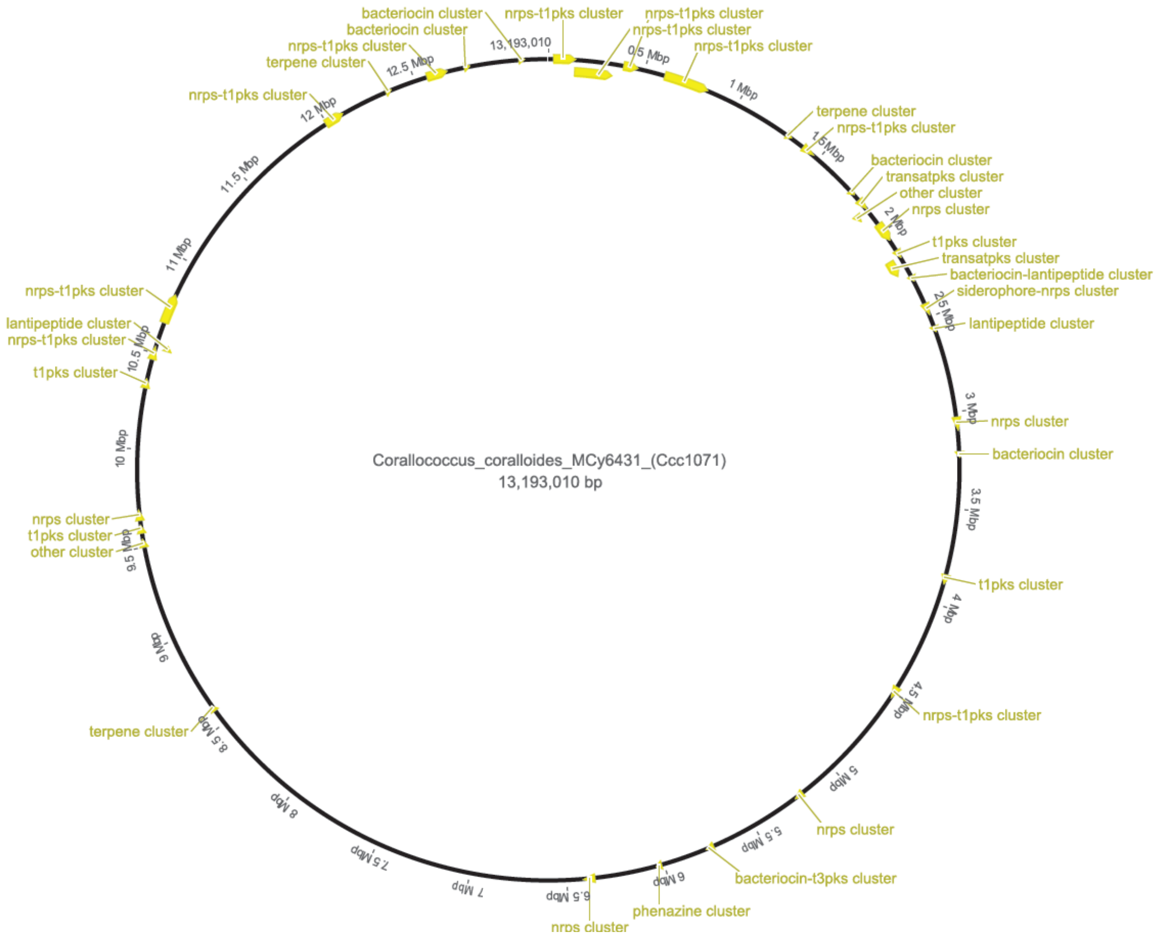

Figure S2-2: The complete genome of *Corallococcus coralloides* MCy6431 is approximately 13.2 Mbp in size. It holds approximately 25 PKS and NRPS gene clusters, furthermore several other secondary metabolite biosynthesis pathways besides the myxovalargin gene cluster.

Media optimization

General modifications on media components were conducted to find optimal production conditions[8]. Since the siderophore myxochelin is produced in high amounts and thereby might influence the production profile of other secondary metabolites, a suppression by supplementation of iron in form of FeEDTA was performed. A total loss of myxochelin production by supplementation with 8 mg/l FeEDTA could be achieved. Production levels of myxovalargin A were slightly increased, whereas myxovalargin B yield was raised about 10-fold (**Figure S2-5**). Further increase of FeEDTA concentration did alter the ratio between myxovalargin A and B in favor of the latter.

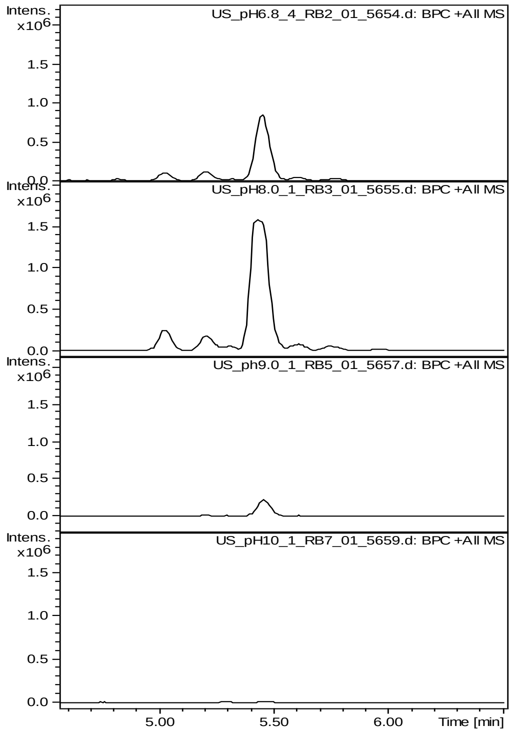

Figure S2-3: Production of myxovalargin A could be observed in a range of pH-6.8 – 10. The maximum of production was observed at pH 8.0

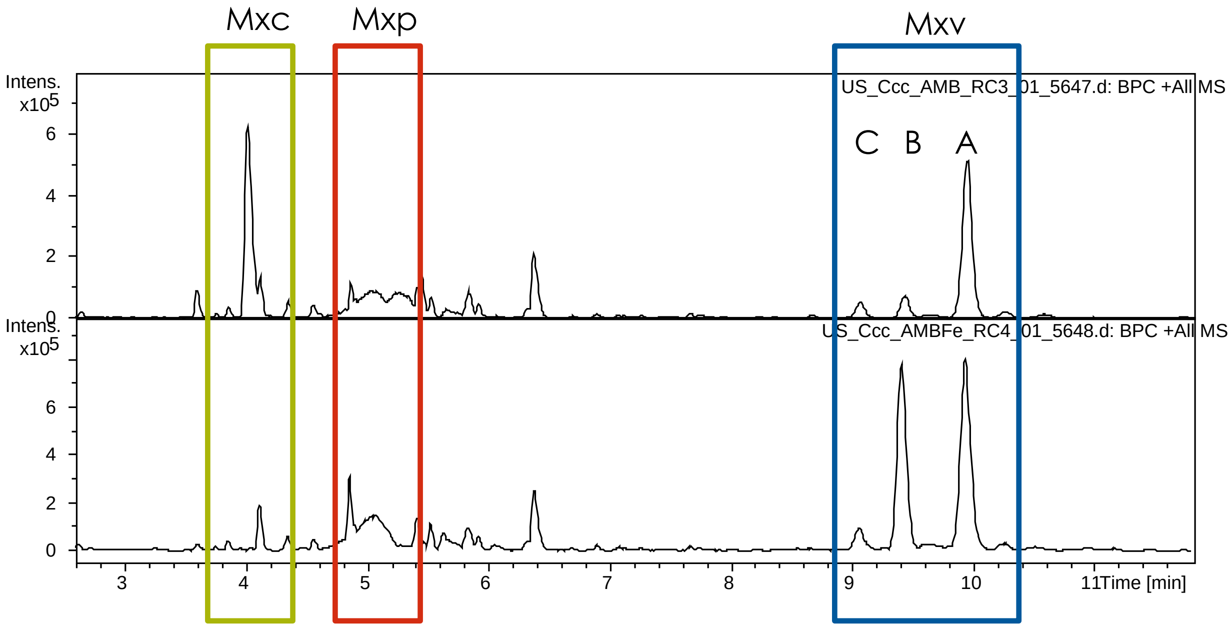

Figure S2-4: Chromatograms of Ccc1071 grown in AMB medium without FeEDTA (upper chromatogram) and with 8 mg/l FeEDTA (lower chromatogram). The production of the iron chelator myxochelin (Mxc; green box) diminishes under FeEDTA supplementation, whereas the myxovalargin production (Mxv; blue box) is increased, especially for myxovalargin B. The production of the myxoprincomides (Mxp; red box) is also influenced by this change.

pH range: Further studies were conducted to specify a suitable pH range for MCy6431 (**Figure S2-4**). The general pH of AMB medium is set at pH 6.8, therefore the strain was cultivated ranging from pH 5 to 10. All cultures were harvested simultaneously when no additional growth could be observed in the cultures. Growth and production in pH 5 and 6 were not detectable. The standard pH 6.8 as reference yielded reasonable growth and production. Nevertheless, at pH 8 a twofold production increase could be achieved compared to pH 6.8. At pH 9 production decreased and pH 10 showed minimal growth and production. Therefore, a pH of 8.0 was determined as optimal production condition with a fast growth of cells. These results were underlined by pH monitoring in shake flask versus fermenter, where an increase of pH from 7.0 to a stable 8.0 during cultivation was observed. However, since with pH 8.0 other growth challenges occurred during fermentation, the pH 7.0, which allows almost equal growth properties, was used for all described experiments.

Choice of media: By cultivation in different media using the 24 deep-well Duetz systems (Kuhner shaker), additional information on suitable media combinations could be determined. Several commonly used media for myxobacteria were used at 8 mL each. Following addition of XAD 16 the strain was grown for several days at 30 °C and 180 rpm. Extracts were harvested, extracted und compared. In general, a superiority of media containing larger amounts of nitrogen source yielded in an increased production of myxovalargin. Probion containing media were shown to be a suitable nitrogen source, as well as other peptones. Nevertheless, in several media, the strain showed conglomerates, which reduced their suitability for further upscale development. As this phenomenon was observed for strains with high production yields, a putative reason could be the fast growth with the nutrient rich media, which leads to local high cell densities resulting in agglomeration. Cultivation in P-medium, a nutrient rich medium with high production yields in Duetz cultivation and small scale used in the MWIS group of the HZI Braunschweig, support these findings since strong aggregation was reported, which led to foam formation in the cultivation process and clogging of filters in the downstream process. Therefore, adapted AMB medium still seems reasonable for microbiological and biotechnological improvement of growth. However, the low nutrient supply of 2.5 g/l casitone can be optimized by adding additional nitrogen sources or replacing the nitrogen. Preliminary experiments suggest a concentration of 5 g/l or the substitution of casitone by other nitrogen rich peptones, but this was not studied into detail. Using these further optimizations with casitone increase and pH adjustment, cultivation was transferred to small scale fermentation.
Cultivation was repeated with the second potent myxovalargin producer, Ang983. Results for MCy5730 are comparable to MCy6431 (**Figure S2-6**). The cultivation of MCy5730 also shows its high production yields of myxovalargins in different media. For high throughput production of myxovalargins MCy5730 seems to be superior to MCy6431. Fermentation of MCy5730 was also conducted at the HZI Braunschweig. However, due to the biotechnological achievements with MCy6431, this strain was used for further experiments.

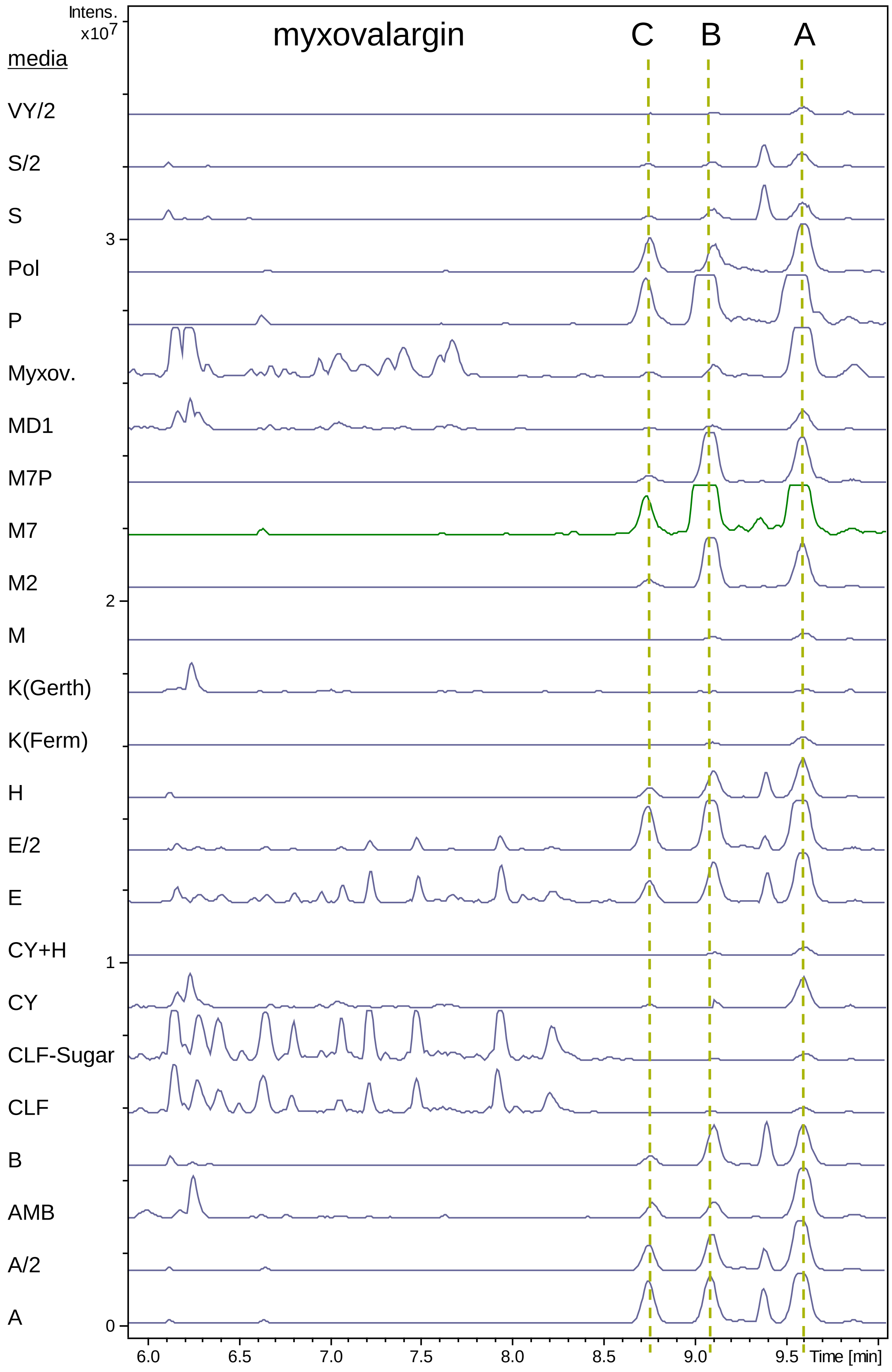

Figure S2-5: 25 deep-well cultivation of MCy6431. Nitrogen rich media show increased production of the myxovalargins.

Fermentation and growth specification

To allow large scale production of myxovalargins, cultivation was transferred to fermentation scale including downstream process development for MCy6431. First cultivation experiments in 1 L fermentation vessels led to massive aggregation on steel surfaces like baffle, electrodes, and air tubing. This problem was overcome completely by removal of baffles, which lead to a turbulent flow and shear stress, a putative reason for the strong agglomeration. Cultivation was conducted as batch culture and in preliminary experiments compared to shake flask cultivation. As it was shown that production and growth were optimized by pH adjustment, pH trend was analysed in shake flask, since during fermentation this parameter can be hold steady. A pH adjustment could be detected, leading from pH 7.0 to a pH of 8.0 during cultivation process. When comparing growth rates between shake flask and fermenter, only slight differences could be detected, 0.081 h^{-1} versus 0.076 h^{-1}, respectively. In a following experiment in 1 L scale cultivation with XAD-16 was compared to no XAD addition. Results showed only small difference in growth rates of 0.076 h^{-1} for the XAD culture versus 0.084 h^{-1} without XAD. Nevertheless, in shake flask it could be shown that lower cell density is achieved, when cultivating without XAD 16.
To analyse growth and metabolite production, samples of supernatant and cell extract were taken separately from the broth of fermenter and shake flasks (**Figure S2-7**). OD_{600} measurements were taken in triplicates at each time point. Log phase was reached after 20h, while stationary phase followed after approximately 40h. Stationary phase quickly fades off into dying phase within about 20 h. XAD was not added in this experiment, since it cannot be ensured to extract reproducible amounts from the culture broth, especially from the fermenter. Therefore, the previously reached maximal cell density of OD600 2.2 could not be reached in this experiment. Nevertheless, a dependency of secondary metabolite production and cell growth could be determined. Amounts are given relatively in normalized units. Three main compound classes were analysed: phenalamides (A1 and A2), myxoprincomides (mxp547) and the myxovalargins (A-C). Phenalamides can be found in cell extracts and supernatant, while myxoprincomides and myxovalargins mainly occur in the supernatant. In the shake flask experiment phenalamides are primarily accumulated in the cells. In the fermenter phenalamides are produced during log-phase and decrease in die-off phase, which is also true for the myxoprincomides. A similar production trend for the myxoprincomide can be seen in the shake flask, though with a time delay of approximately 20 h. Myxovalargins on the other hand are constantly excreted in the supernatant. Even during dying phase the myxovalargin concentration increases in the broth. Nevertheless, the fastest increase in production rate is achieved during log phase.

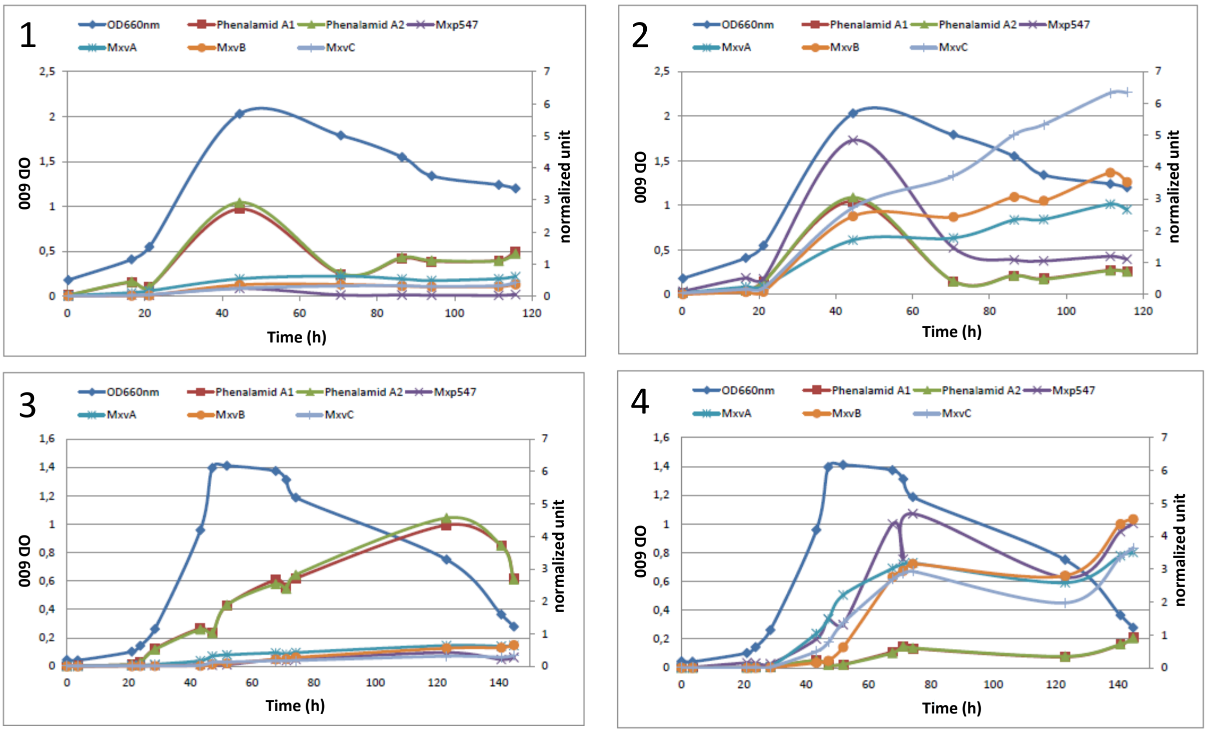

Figure S2-6: Comparison of fermentation and shake flask cultivation. The upper panels (1, 2) show cultivation in shake flasks, while panel 3 and 4 show fermenter cultivation. Samples of cell extract (left panels) and supernatant (right panels) were taken separately and extracted. Production levels of major secondary metabolites are given in normalized units as factor compared to the internal standard sulfadimethoxin (6,75 mg/l), allowing a relative comparison, since pure standards of the compounds were not available for the experiment. Production of the myxovalargins is constant, although the main increase takes place during log and stationary phase. Myxoprincomide and phenalamide production on the other hand decreases in death phase.

3. Myxovalargin biosynthetic gene cluster analysis

Gene cluster identification: Using the established mutagenesis method, biosynthetic gene clusters (BGCs) of the most abundant metabolites in the chromatographic profile of MCy6431 were knocked out[8]. Comparison between the knockout mutants and the wildtype showed no prominent difference in their antibiotic profile against chosen gram negative and positive bacteria except for the myxovalargin knock-out, which led to almost a total loss of antibiotic activity of the crude extract.

All references to genetic information from the myxovalargin BGC relate to sequences from MCy6431, if not mentioned otherwise. Knockout experiments narrowed down the core region of the myxovalargin cluster to a sequence of almost 70 kbp. The genetic sequence encoding the domains of the fourteen NRPS modules of the myxovalargin BGC comprises approximately 58 kbp, while an upstream region of approximately 8 kbp encodes additional proteins participating in the myxovalargin production. The NRPS domains encoding region is subdivided in five genes (*mxvA* – *E*), while the upstream part covers additional 6 genes (*mxvF* – *K*). With 58 kbp the myxovalargin BGC is one of the largest reported and functionally assigned NRPS-type gene clusters up to date. Beside most of the core genes, several adjacent genes were knocked out to narrow down the cluster boundaries (**Table S3-1**). Furthermore, putative genes or pathways, which could be responsible for production of myxovalargin precursors, were targeted for knockout experiments like the arginine decarboxylase or the branched chain ketoacid dehydrogenase complement.

Note: The sequence information for the myxovalargin BGC is publicly available through GenBank accession number OQ092403.

Table S3-1: List of genes of the myxovalargin biosynthesis cluster, adjacent genes, or putatively associated genes.

ORF	Similarities	Proposed function	Knockout/production	Distance from NRPS start
mxvA	NRPS	module 1 – 3	yes/ no	1 – 10854 bp
mxvB	NRPS	PCP domain	Not achieved	10866 – 11378 bp
mxvC	NRPS	module 4 – 9	yes/ no	11375 – 36451 bp
mxvD	NRPS	module 10 – 13	yes/ no	36454 – 52122
mxvE	NRPS	module 14	Not achieved	52128 – 58064 bp
mxvF	Microcystin synthetase associated protein	TE II function	-	79-903 bp (up)
mxvG	MbtH like domain containing protein	MbtH function	-	1506 – 1721 bp (up)
mxvH	Antibiotic biosynthesis protein	Val-/Ile-*β* hydroxylase	yes/ no	1798 – 3381 bp (up)
mxvI	ABC transporter related protein	ABC transporter – Permease	yes/ yes	3394 – 5232 bp (up)
mxvJ	Tyrosine 2,3 aminomutase	Tyrosine aminomutase	yes/ no	5304 – 6914 bp (up)
mxvK	ABC transporter related protein	ABC transporter – ATPase	yes/ yes	6911 – 8431 bp (up)
ORF 1	UPF0059 membrane protein	unknown	-	8625 – 9218 bp (up)
ORF 2	Cupin	Unknown	yes/ yes	9237 – 10364 bp (up)
ORF 3	Transcriptonal regulator, LysR family	Unknown	Not achieved	10478 – 11365 bp (up)
ORF 4	DNA alkylation repair enzyme	Unknown	-	11376 – 12119 bp (up)

ORF 5	Dehydrogenase/ reductase SDR family member	dehydrogenase	Yes/yes	12277 – 13179 bp (up)
ORFdown1	Adenylate cyclase	Unknown	Yes/ yes	76 – 4259 bp (dn)
ORFdown2	Hypothetical protein	Unknown	-	4400 – 4802 bp (dn)
ORFdown3	Multicopper oxidase domain containing protein	Unknown	Yes/yes	5002 – 6463 bp (dn)
ORFdown4	Hypothetical protein	Unknown	-	6559 – 6712 bp (dn)
ORFdown5	Deoxyhypusine synthase	Unknown	Yes/ yes	6753 – 7722 bp (dn)
ORFdown6	Long chain fatty acid CoA ligase	Unknown	Not achieved	7963 – 9787 bp (dn)
adc	Arginine decarboxylase	Agmatine biosynthesis	Not achieved	Distant
gct	Glutaconate transferase	Isovaleryl biosynthesis	Yes/ yes	~ 30 kbp (dn)
bkd	Branched chain ketoacid dehydrogenase	Isovaleryl biosynthesis	Yes/ yes	~ 90 kbp (dn)

Proteins MxvABCDE

The NRPS assembly line is located on genes *mxvA – E*. The total assembly line spans fourteen modules, which are located on *mxvA, C, D* and *E*. All modules are organized in linear order from *mxv*A modules 1 – 3, *mxvC* modules 4 – 9, *mxvD* modules 10 – 13 and *mxvE* module 14.

Table S3-2: Predicted domain specificity in the myxovalargin gene cluster. C = condensation domain, A = adenylation domain, PCP = peptide carrier protein, MT = methyltransferase; DcL = condensation of upstream D amino acid with downstream L amino acid. LcL = condensation of upstream L amino acid with downstream L amino acid.

Gene	Module	Domain order	Domain specificity	Phenotype in Myxovalargin A
mxvA	**Module 1**	**C-A-PCP**	**C domain: LcL**<br><br>**A domain: Val**	**dehydro - valine**

	**Module 2**	**C-A-PCP**	**C domain: DcL**<br><br>**A domain: Ala**	**L-alanine**
	**Module 3**	**C-A-MT-PCP**	**C domain: LcL**<br><br>**A domain: Ala**	**N-methyl-L-alanine**
mxvB	**-**	**PCP**	**-**	**-**
mxvC	**Module 4**	**C-A-PCP-E**	**C domain: LcL**<br><br>**A domain:Val**	**D-valine**
	**Module 5**	**C-A-PCP-E**	**C domain: DcL**<br><br>**A domain: Val**	**D-valine**
	**Module 6**	**C-A-PCP**	**C domain: DcL**<br><br>**A domain: *β*-tyrosine**	**(S)-*β* -tyrosine**
	**Module 7**	**C-A-PCP-E**	**C domain: LcL**<br><br>**A domain:Val**	**D-valine**
	**Module 8**	**C-A-PCP**	**C domain: DcL**<br><br>**A domain: Val**	**dehydro - valine**
	**Module 9**	**C-A-PCP-E**	**C domain: DcL**<br><br>**A domain: (Gln)**	**D-arginine**
mxvD	**Module 10**	**C-A-PCP**	**C domain: DcL**<br><br>**A domain: (hydrophobic-aliphatic)**	**L-valine**
	**Module 11**	**C-A-PCP-E**	**C domain: LcL**<br><br>**A domain: Val**	**D-valine**
	**Module 12**	**C-A-PCP**	**C domain: DcL**<br><br>**A domain: Ile**	**dehydro-isoleucine**
	**Module 13**	**C-A-PCP-E**	**C domain: DcL**<br><br>**A domain: Ala**	**D-alanine**
mxvE	**Module 14**	**C-A-PCP-E**	**C domain: DcL**<br><br>**A domain: Val**	**hydroxy-valine**
	**-**	**C**	**C domain: DcL**	**-**

Modules 1 – 3 (MxvA) encoded on *mxvA*: *MxvA* has a size of 10854 bp. Module 1 consists of the domain order C-A-PCP, module 2 of C-A-PCP and module 3 of C-A-MT-PCP. Thus, all necessary basic domains for each module are present. A domain specificity (according to NRPS-predictor 2)[12] of module 1 shows similarity to valine incorporating domains, module 2 for alanine and module 3 again for alanine. All predictions for A domain specificity fit with the amino acid order in myxovalargin A. Module 1 shows LcL reaction specificity, nevertheless the module is closely related to condensation domains, which catalyse condensation with starter units and fits to the function of module 1. Module 2 interestingly shows DcL reaction specificity although module 1 incorporates L-valine and does not contain an epimerization domain, thus LcL reactivity would be expected. However, the valine incorporated by module 1 in the final molecule is present as dehydro-valine. While the module sequences show no hint on the biosynthesis of the dehydro amino acid, the C-domain specificity can be seen as a hint on the timing of this modification. Comparison with the two other condensation domains (module 9 and 13) catalysing condensation with a dehydro-amino acid building block, show the same specificity. Moreover, these domains group together in a separate clade. This indicates that the condensation performed by C domains of modules 2, 9 and 13 couple an amino acid to a building block from the previous module, which is not in the L-form. As the dehydro amino acids exhibit no stereocenter, the grouping of the condensation domains imply that the modification of the L-amino acids is performed before the condensation. Considering that a hydroxy moiety is present in myxovalargin A as well as the presence of dehydro amino acids in parallel with hydroxy moieties in other molecules like the yaku´amides, the proposed mechanism is a hydroxylation followed by a dehydration. Module 3 again exhibits the expected LcL specificity. The N-methyltransferase (MT) in module 3 is responsible for the N-methylation of the alanine as described for the structure. Additional information on the origin of the starter unit 3-methylbutyryl is not present on this gene.

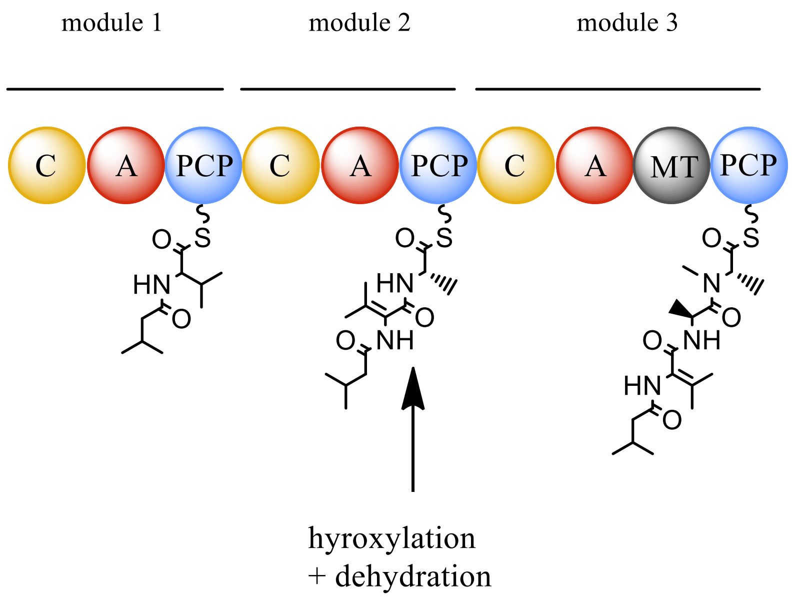

Figure S3-3: Domain organization of MxvA. Module 1 incorporates the valine building block, which is tethered to the 3-methylbutyryl starter. The proposed mechanism is a hydroxylation followed by a dehydration of the valine, thus, forming the dehydro-valine moiety. The second elongation step by module 2 adds an alanine, while module 3 adds the second alanine. The methyltransferase in module 3 performs the respective N-methylation as present in myxovalargin A.

Single PCP domain encoded on *mxvB:* *MxvB* is a short reading frame of appr. 600 bp. It encodes a PCP domain in the downstream part of the sequence, while the upstream region shows similarities to an A domain terminus. The function of this PCP-like domain is unclear. Due to its small size it was not possible to knockout this gene. A double crossover approach was not successful. The gene is conserved in all accessible myxovalargin BGCs. Thus, it is postulated that the gene holds a function during incorporation of precursors.

Modules 4 – 9 (MxvC) encoded on *mxvC*: *MxvC* encodes module 4 (C-A-PCP-E), module 5 (C-A-PCP-E), module 6 (C-A-PCP), module 7 (C-A-PCP-E), module 8 (C-A-PCP) and module 9 (C-A-PCP-E). A domain specificity in order of modules is valine, valine, *β*-tyrosine, valine, valine and arginine. Modules 4 and 5 contain an epimerization domain, which form the D-amino acids as expected by the chemical structure of myxovalargin A. The A-domain of module 6 incorporates the unusual *β*-tyrosine building block, which is derived from tyrosine by the tyrosine aminomutase of *mxvJ,* characterized and described by Krug et al.[13]. In contrast to the assigned stereochemistry of valine in myxovalargin A, module 7 shows an epimerization domain. This discrepancy to the described stereochemistry of myxovalargin A is discussed in further below. Module 8 incorporates valine. While module 9 must incorporate arginine, domain specificity by NRPS predictor 2 indicates glutamine (80% similarity). Several examples like the myxoprincomide cluster exhibit discrepancies between A domain prediction and actual substrate.[14] This inaccuracy can be caused by substrate promiscuity[15] or closely related building blocks with similar chemical or stereochemical properties. The module 9 A domain region is conserved in all sequenced myxovalargin

BGCs. The module exhibits an epimerization domain, which is required to form the D-amino acid. Module 4 connects L-alanine of module 3 with valine and shows LcL specificity. C domain specificity of modules 5 and 6 is DcL, which is in alignment with the epimerization domains in module 4 and 5. Module 7 conjugates LcL in accordance with L-valine of module 6, while module 8 connects DcL caused by the epimerization domain in module 7. As module 9 couples the dehydro amino acid with valine, the C-domain specificity fits with the predicted model as described for module 2 in MxvA. Thus, C-domain reaction predictions fit with the module order and epimerization domain organization.

Modules 10 – 13 (MxvD) encoded on *mxv*D: Module 10 consists of the domain order C-A-PCP. The A domain specificity for module 10 is weak. The condensation domain specification for DcL matches the incorporation of D-arginine in module 9. Module 10 does not show an epimerization domain; this discrepancy to the described stereochemistry of myxovalargin A is discussed further below. Module 11 with C-A-PCP-E order and A domain specificity for valine fits with the chemical structure. The LcL specificity further supports the missing epimerization domain in module 10. Module 12 consists of C-A-PCP and the A domain is specific for isoleucine. Condensation domain specification for DcL is in accordance with module 11. Isoleucine is present as dehydro amino acid. The module gives no hint on the modification reaction, whereas module 13 again shows C domain specificity for a DcL reaction as described for module 2 in MxvA and modeule 9 in MxvC. Furthermore, domain order of module 13 is C-A-PCP-E, with an A domain specificityfor alanine. The epimerization domain catalyzes the formation of the D-alanine.

Terminal module 14 (MxvE) encoded on *mxvE:* *MxvE* encodes module 14, which consists of C-A-PCP-E with an A domain specificity for valine. The specificity of valine matches with the appearance of the hydroxy valine in this position. However, there is no particular hint on the hydroxylation process. The epimerization domain in module 14 shows no abnormalities in sequence alignment and is therefore regarded as functional epimerization domain. The condensation domain catalyzes the expected DcL reaction. After module 14 a single C domain follows as last domain of the open reading frame and thus of the myxovalargin NRPS cluster. The C domain does not show any noticeable difference to regular C domains and groups in the C domain alignment with DcL domains, which supports the functionality of the E domain in module 14. The *mxvE* region including the stop codon after the terminal C-domain was identified as highly conserved in all sequenced myxovalargin producers. A thioesterase domain I (TEI) is not present in module 14 or in the BGC. The role of the C-domain in the termination mechanism and incorporation of agmatine is discussed later.

Thioesterase and MbtH like protein – Genes *mxvF* and *mxvG*

Two short genes are located upstream of the NRPS assembly line. *MxvF* encodes a single thioesterase (TE) of ~850 bp. Thioesterases type I are responsible for the cleavage of the thioester between the pPant arm of the PCP domain from the terminal module and the molecule assembled. Thioesterase II domains on the other hand cleave intermediates from the assembly line, when blocked by false assembly or other issues. Since a TEI is not present in the terminal NRPS module of myxovalargin, the thioesterase on *mxvF* was analysed *in silico* to specify its putative function. The comparison shows, that *mxvF* clearly groups with thioesterases type II (TE II) and therefore the above described function as defined for other TE II is assumed.

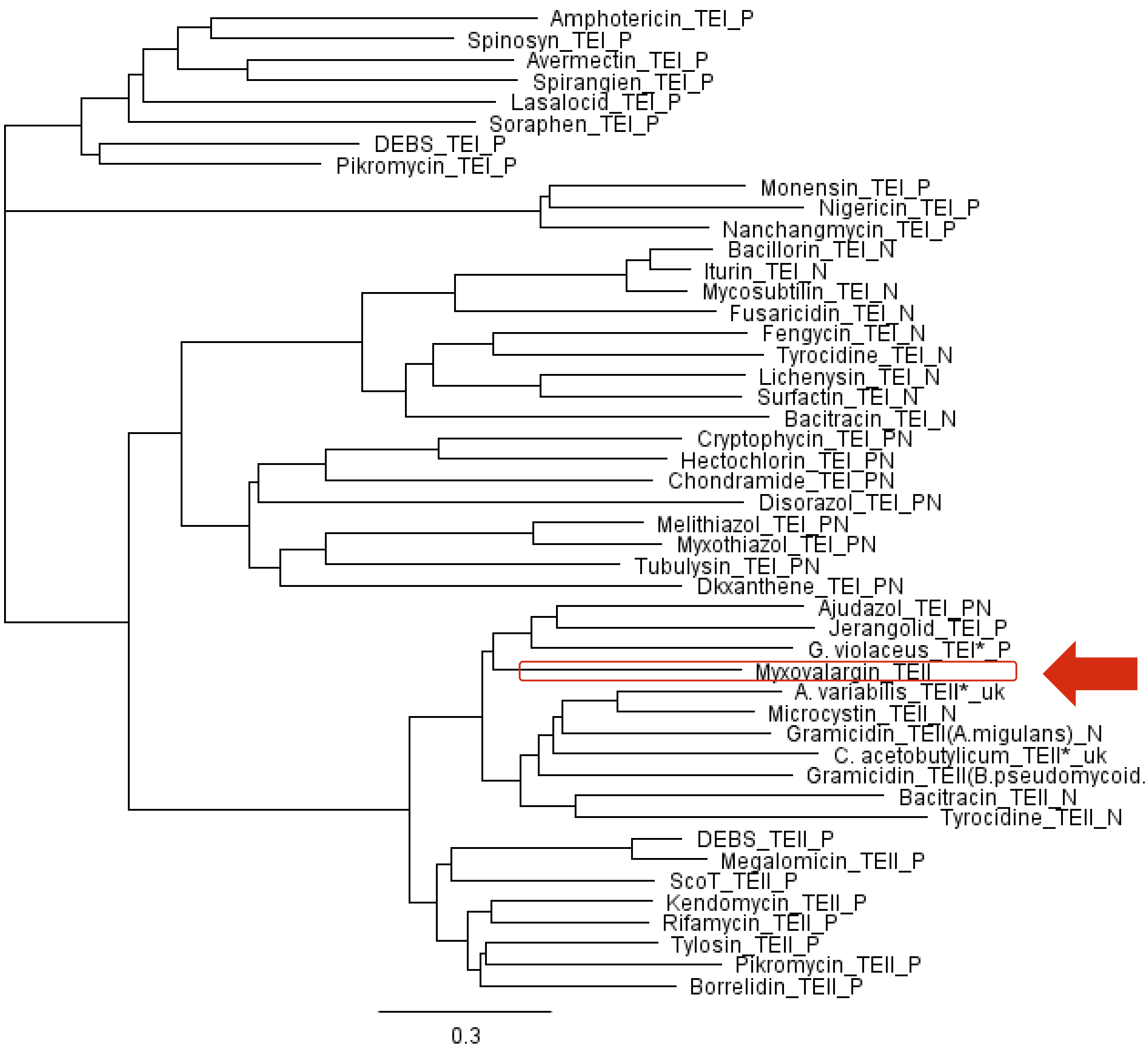

Figure S3-4: Thioesterase type I (TEI) and type II (TEII) were aligned and their similarity visualized in a tree diagram. The gene *mxvF*, marked in red, groups with other TEII domains and is therefore proposed to have the function of cleaving intermediates from blocked PCP domains.

MxvG (~230 bp) the second ORF upstream of *mxvA* shows similarity to MbtH-like proteins. Those proteins were shown in several cases to facilitate activation by the adenylation domain in NRPS assembly lines. Thus, their activity correlates with the production rate of a secondary metabolite, but they are not essential.

Putative β-hydroxylase - *MxvH*

The formation of dehydro-valine/isoleucine and hydroxy-valine building blocks is not fully explained by enzymatic functions encoded within the MXV locus. Nevertheless, the results from feeding experiments in combination with sequence characteristics of respective C domains, and results from targeted inactivation of the putative hydroxylase encoded by *mxvH* hint at a mechanism involving sequential hydroxylation and dehydration of peptidic intermediates on the assembly line. The putative candidate gene for this functionality, the 1632 bp ORF *mxvH,* is located ~1800 bp downstream of the myxovalargin NRPS assembly line. Blastp search did not yield significant hits to known enzymes. Nevertheless, a knockout of this gene resulted in total loss of myxovalargin production. To rule out a polar effect on the consecutive genes, including the essential tyrosine aminomutase (*mxvJ*), feeding of β-tyrosine to the *mxvH* knockout mutant was analysed, but did not lead to a restoration of myxovalargin production in contrast to a supplementation of β-tyrosine to the *mxvJ* mutant. Furthermore, does the knockout of the following gene *mxvI* not lead to production abolishment and thereby no polar effect exists between *mxvH* on *mxvI* and *mxvJ*. As mentioned above, blastp search yielded a range of remotely similar sequences from general hydrolase families. Thus, a 3D model of MxvH using Phyre 2 was compared to proteins of the protein data bank (PDB).[16] The β-hydroxylase of the chloramphenicol biosynthesis, CmlA, showed 39% similarity. This enzyme catalyzes the hydroxylation of 4-aminophenlyalanine in β-position during the assembly process. Thus, a functional relationship is postulated, implying that *mxvH* could be the central enzyme in β-hydroxylation of the amino acid building blocks in myxovalargin. Furthermore, two structural elements might be derived by this enzyme which were not yet described in literature, *i.e.*, the hydroxyvaline and the dehydro building blocks of valine and isoleucine, which we reason to be synthesized by hydroxylation and subsequent dehydration. Different observations support this hypothesis. Another large polypeptide which was identified from the marine sponge *Ceratopsion* sp. are the yaku´amides. These large putative NRPS products exhibit concurrent presence of dehydro as well as hydroxy valine and isoleucine moieties in their primary sequence. To date, only few examples of either hydroxy valine/isoleucine or dehydro valine/isoleucine are known. The antrimycins and pyridomycins are examples of dehydro-isoleucine in secondary metabolites. Examples of hydroxy valine can be found in the myxoprincomides [14], as well as the corramycins [17]. Nevertheless, none of these compounds show a simultaneous occurrence of dehydro and hydroxy amino acids, as known for the myxovalargins and yaku´amides. Thus, it seems likely that the biosynthesis mechanism in the myxovalargins and the yaku´amides differs from the other mentioned metabolites. Hence, it explains the lack of *mxvH* in the cluster of pyridomycin, coramycin or myxoprincomide, which was

determined by alignment with *mxvH*. Two closely located putative dehydrogenases, appr. 15 kbp and 30 kbp upstream of *mxvA*, were knocked out in separate mutants by gene disruption, but a loss or alteration of myxovalargin production did not appear. To determine the exact precursor and get hints on the mechanistic timing of the hydroxylation, d6-hydroxy valine was supplemented to a wildtype strain, as well as the *mxvH* knockout mutant. Additionally, d6-hydroxy valine was supplemented together with *β*-tyrosine to rule out any effect on the tyrosine aminomutase. An incorporation of a hydroxy valine precursor was not observed. Thus, it is assumed, that the PCP bound valine intermediate is hydroxylated, which is in accordance to the mechanism described for CmlA, which might be another indication of their substantial biological similarity.

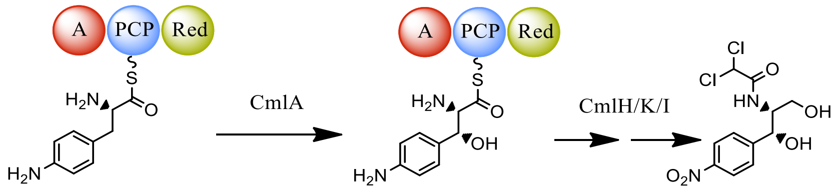

Figure S3-5: CmlA catalyzes the *β*-hydroxylation of 4-aminophenylalanine in chloramphenicol biosynthesis. Due to structural homology, MxvH is assumed to catalyse the *β*-hydroxylation of valine in the case of myxovalargins by a similar reaction mechanism.

Tyrosine aminomutase - *mxvJ*

The gene *mxvJ* encodes a tyrosine aminomutase, which has been *in vitro* characterized previously for its product specificity, identifying selectivity for (S)-*β*-tyrosine in comparison to the tyrosine aminomutase of the chondramides, which generates (R)-*β*-tyrosine[13]. The knockout of *mxvJ* led to a loss of myxovalargin production. Since it was shown by *in vitro* studies that the reaction is independent from the assembly line, it was possible to restore production by supplementation of *β*-tyrosine. Although the production yield of myxovalargin in the knockout mutant by addition of synthesized (S)-*β*-tyrosine was not 100% compared to the wildtype, a production of 60% unambiguously shows the possibility to complement the gene knockout through feeding.

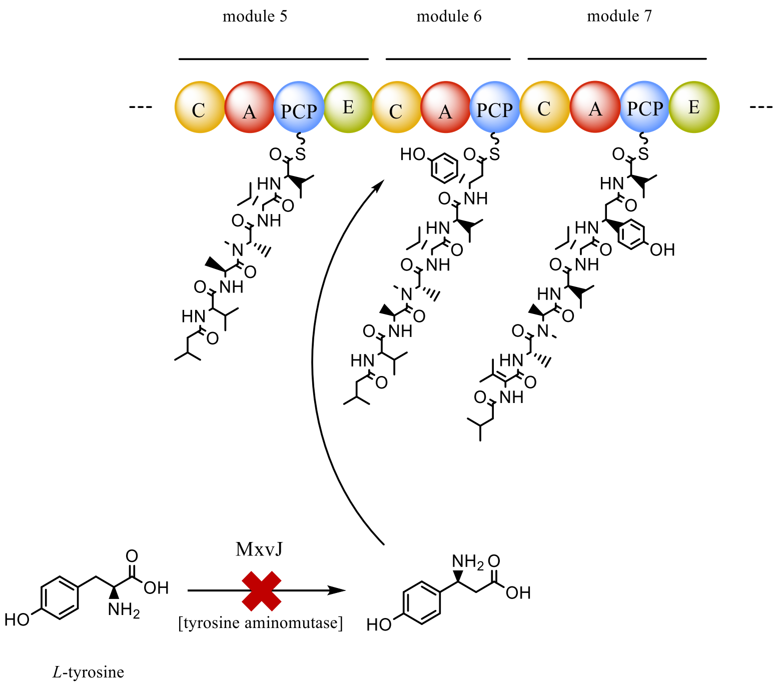

Figure S3-6: (S)-*β*-tyrosine is stereospecifically generated from L-tyrosine by the tyrosine aminomutase (*mxvJ*). The building block is incorporated in the growing polypeptide by module 6 of the NRPS assembly line. The A domain in this module shows specificity for (S)- *β* -tyrosine. By knockout of *mxvJ* myxovalargin production is abolished, but can be restored by supplementation of (S)- *β*-tyrosine.

Agmatine incroporation

Agmatine is the biogenic amine of arginine and is generated via decarboxylation by the arginine decarboxylase (adc). It functions as intermediate of the putrescine biosynthesis.

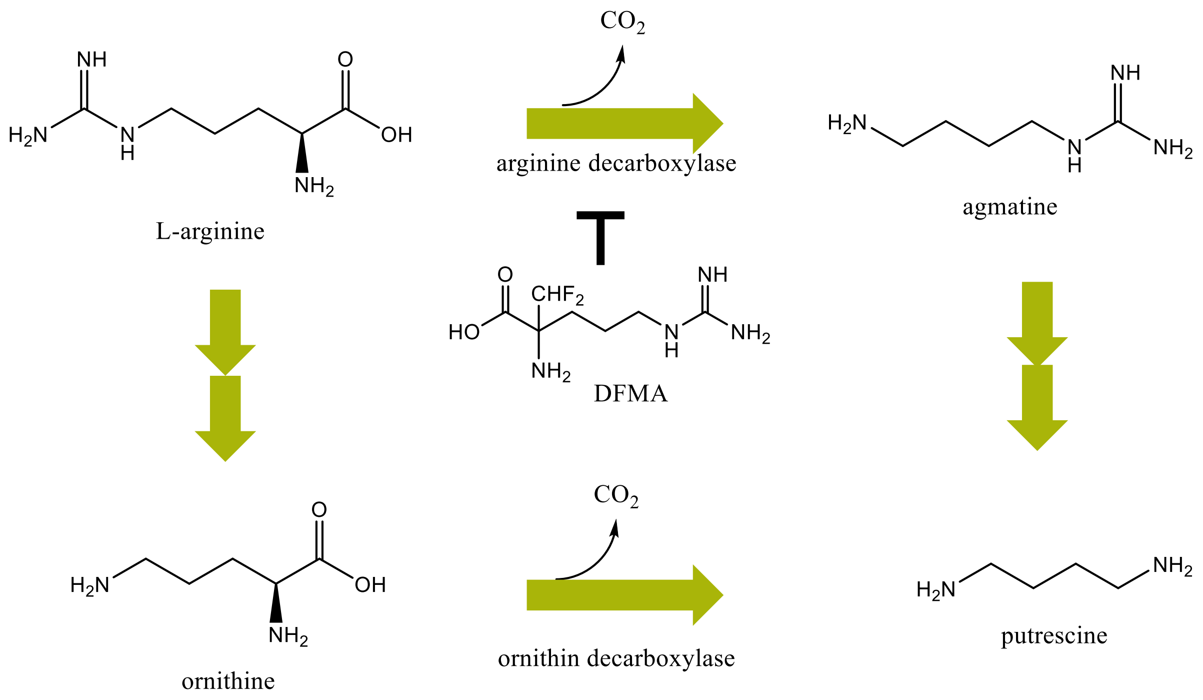

Figure S3-7: The role of agmatine in primary metabolism. Agmatine is generated from arginine, catalysed by the arginine decarboxylase. In further steps, it can be transformed into putrescine, which can also be produced from arginine *via* ornithine by the ornithine decarboxylase. Difluoromethyl arginine (DFMA) irreversibly blocks the arginine decarboxylase by competitive inhibition.

Only one second natural product is known so far, which exhibits an agmatine moiety. The NRPS assembly line of this compound, called aeruginoside, shows a terminal C- and PCP-domain following the final module.[18] The PCP domain was postulated to be inactive and the dissociation to be facilitated by condensation of the agmatine residue with the polypeptide chain catalysed by the C-domain, thus offering no carboxy group to form a thioester with the consecutive PCP domain. [18] The actual incorporation of agmatine instead of an incorporation of arginine followed by decarboxylation could, however, not be proven. The compound class of aeruginosides is produced by several Planktothrix and Microcystis strains. Interestingly also the presence of argininol and argininal, instead of agmatine was shown for certain Mycrocystis strains, but not for Planktothrix. Yet, these producers harbour a complete module (C-A-PCP-Red) instead of the rudimentary C-PCP domains.[19] Also the spumigins, which are structurally related to the aeruginosides offer argininal, argininol and even complete arginine C-terminal residues.[20] Hence, these biosynthesis mechanisms seem to vary from the mechanism of aeruginoside B from Planktothrix and the myxovalargins from Myxobacteria. A terminal condensation domain, which does not belong to a complete module, does also occur in case of the myxovalargins located on *mxvE*, and is assumed to catalyse the condensation of the free agmatine with the polypeptide chain. Hence, an *in silico* analysis was conducted, which did not provide evidence for inactivity. Furthermore, no unusual features or grouping to condensation domain-like proteins which are able to catalyse thioesterase reactions, as known for CroK from the crocacin pathway in Myxobacteria, were found.

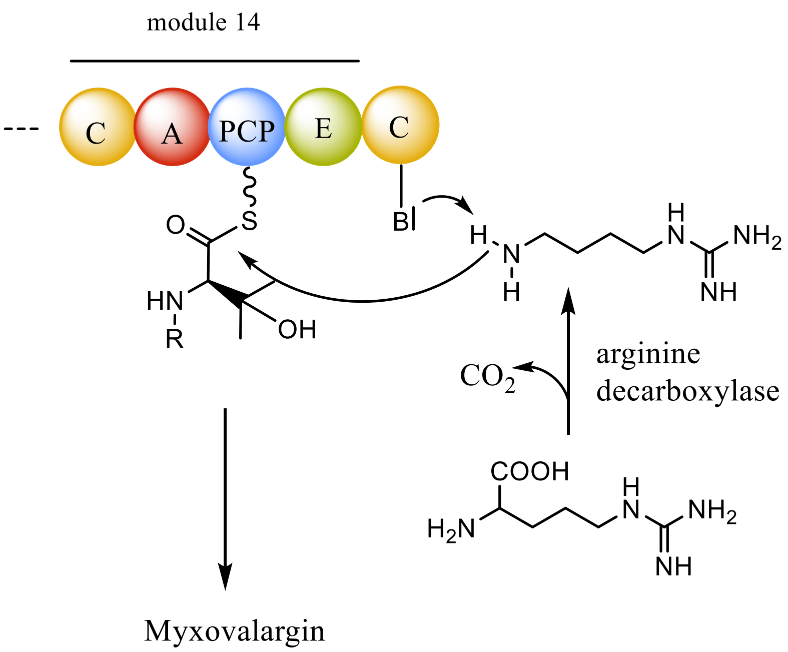

Figure S3-8: Suggested mechanism of the incorporation of agmatine in the polypeptide chain by the terminal condensation domain. The lack of a carboxy group in the terminal agmatine residue results in a dissociation of the assembly line. The enzyme arginine decarboxylase (adc) was shown to catalyse the synthesis of arginine from agmatine and thus is assumed to be the precursor building protein.

By blast comparison, the arginine decarboxylase gene could be found in most myxobacterial genomes, thus underpinning its central biosynthetic role. Site-directed mutagenesis was carried out, leading to numerous mutant colonies in several attempts. Nevertheless, none were shown to interrupt the gene of interest. Furthermore, extracts of these mutants did not show a loss of myxovalargin production. Since the single crossover construct was shown to be correct by SANGER sequencing, the knockout of this enzyme seems to be impossible in these organisms. From literature a variety of reversible and irreversible inhibitors of the arginine decarboxylase are known. [21] The most potent irreversible inhibitor 2-amino-2-(difluoromethyl)-5-quanidinopentanoic acid was fed at 0.2 mM to inhibit adc. Neither production loss, nor growth inhibition could be observed.

Since the chemical synthesis of agmatine was achieved in the total synthesis project of myxovalargin A, it seemed possible to synthesize, supplement and detect the isotope labelled and incorporated agmatine by mass spectrometry. Unfortunately, the synthesis of ^{13}C, ^{14}N-labelled agmatine was not successful. Therefore, a more sophisticated approach by reverse feeding was pursued.

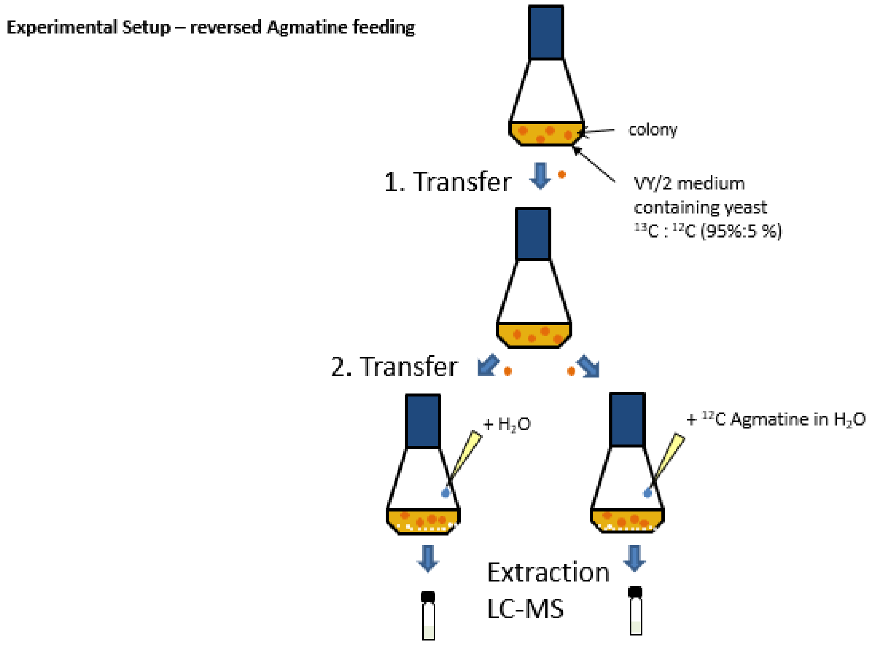

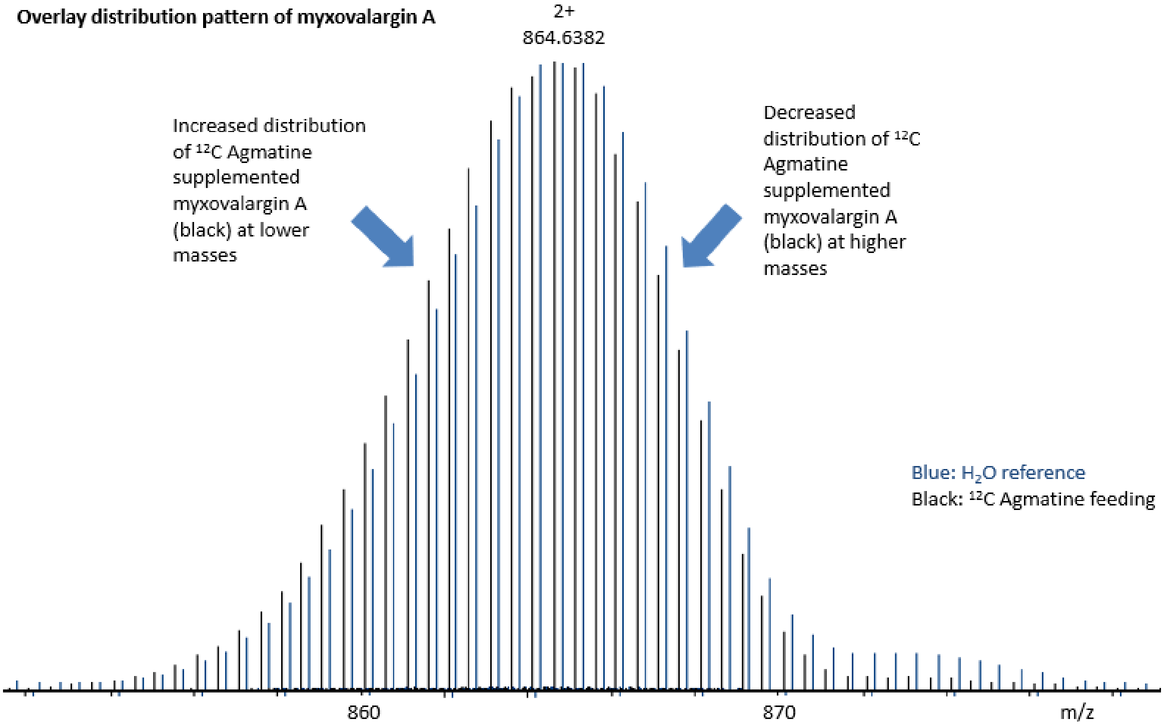

Figure S3-9: In the reverse feeding experiment cells of MCy6431 were cultivated in VY/2 medium containing yeast of ^{13}C: ^{12}C (95:5 %) as only carbon source. Cells were transferred twice to reduce ^{12}C contamination. One sample was fed with ^{12}C agmatine, the other with H2O as reference. After extraction of the adsorber resin, the isotope distribution pattern of myxovalargin taken by LC-MS was compared as overlay. The sample fed with ^{12}C agmatine (black) shows an increased distribution of lower mass variants compared to the reference control. This indicates the incorporation of agmatine.

The reverse feeding experiment was conducted using Isogro®-^{13}C Powder-Growth medium (99 atom % ^{13}C). Strain MCy9171 and MCy6431 were transferred from agar plate directly in the labelled medium to reduce contamination with ^{12}C. Supplementation of either agmatine, *β*-tyrosine (pos. control) or water (neg. control) at 0.2 mM or at equivolume, respectively, lead to a detectable incorporation of agmatine and of the positive control *β* -tyrosine, thus confirming in a second experiment the incorporation of agmatine as precursor for the terminal building block.

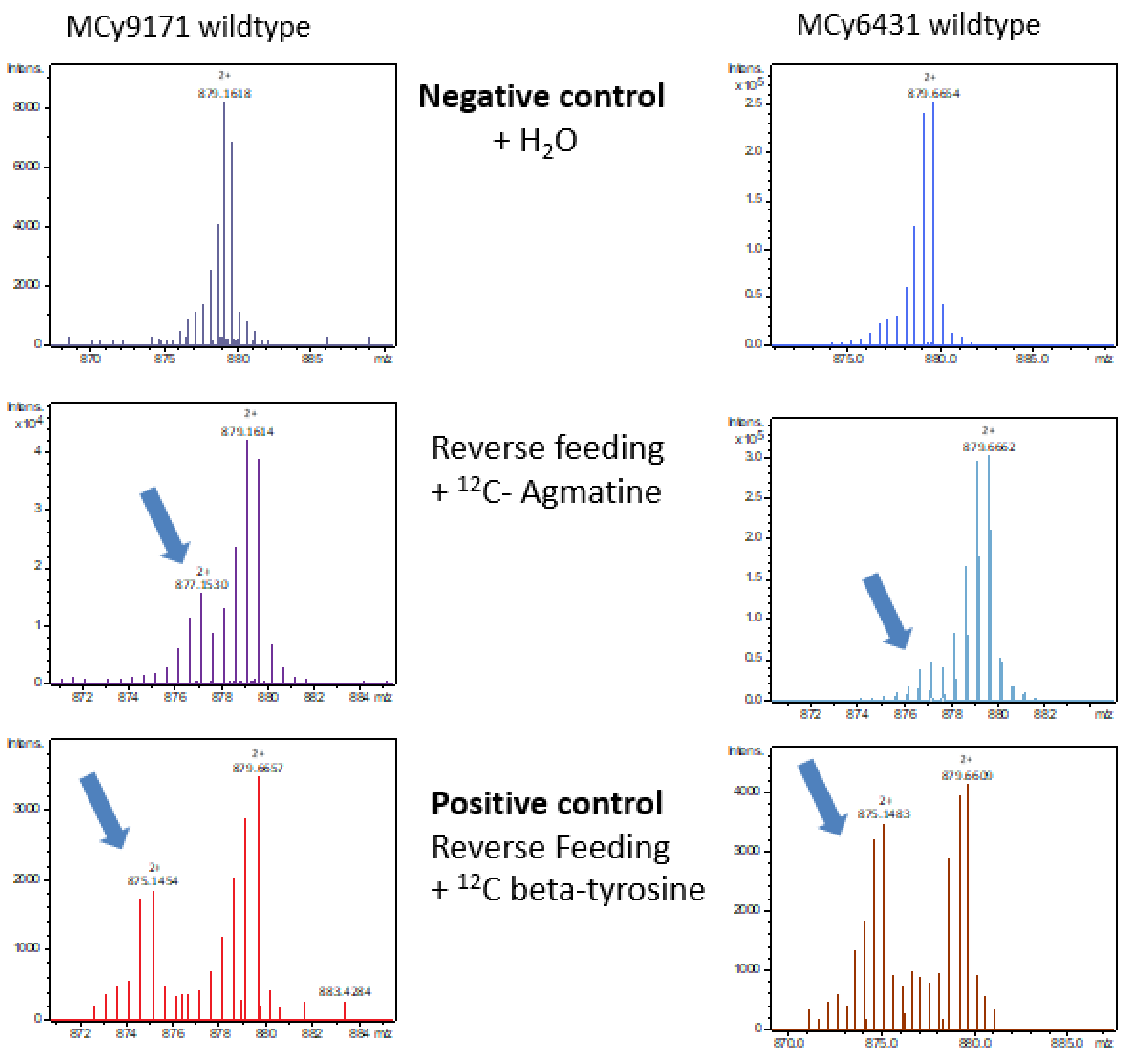

Figure S3-10: A reverse feeding experiment in 99% ^{13}C Isogro® medium showing the incorporation of different ^{12}C precursor in ^{12}C labelled myxovalargin A in the wildtype. Left panels were acquired from wildtype strain MCy9171, while right panels belong to wildtype strain MCy6431. The upper row shows the wildtype with blank solution (H_2O) and a myxovalargin A mass of 879 m/z. The second row demonstrates the incorporation of ^{12}C-agmatine resulting in a mass of 877 Da, which leads to a mass shift of 5 m/z, thus showing a 2.5 Da mass shift for the $[M+H]^{2+}$ ion in both strains. The positive control with C^{12}-*β*-tyrosine in the bottom panel exhibits the expected mass shift for its incorporation, proving the suitability of the experimental setup with ^{12}C precursor reverse feeding in ^{13}C Isogro® medium.

The starter unit: Isovaleryl-CoA

The starter unit in the case of myxovalargin A likely stems from isovaleryl-CoA as[22–24] previously demonstrated for other myxobacterial secondary metabolites. Its biogenesis from leucine was confirmed by feeding with leucine-d10, whereas the isobutyryl starter unit found in myxovalargins B and C derives from valine. The described 3-methyl butyric acid starter unit is commonly found in different myxobacterial secondary metabolites like the myxalamids, myxothiazol and aurafuron. The origin of the starter as isovaleryl-CoA (IV-CoA) has been studied in detail and lead to the description of two different mechanism in myxobacteria[24]. Beside the decarboxylation of leucine by a branched chain ketoacid dehydrogenase complex, an alternative IV-CoA biosynthesis (Aib) has been described in myxobacteria. Both gene complexes can potentially provide the precursor for the myxovalargins.

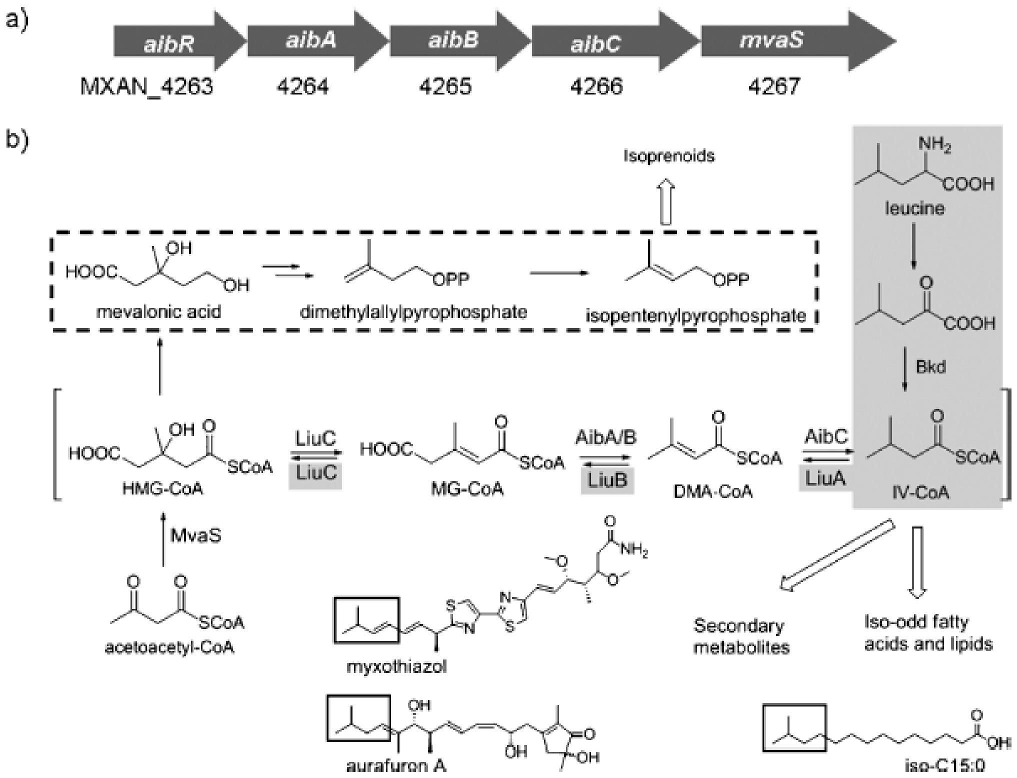

Figure S3-11: Pathways, which lead to the production of the Isovaleryl-CoA starter, which was shown to be involved in the myxothiazol and aurafuron A biosynthesis. Since neither a knockout of genes encoding the branched chain ketoacid dehydrogenase complex nor the Hydroxymethylglutaryl-Coenzyme A (HMG-CoA) pathway by disruption of *aibA/B* led to loss of myxovalargin A production, it is assumed that both pathways can provide the starter for myxovalargin A production (picture taken from Li et al.[24])

To confirm leucine as precursor for the isovaleryl starter unit, d10-leucine was supplemented to the myxovalargin producer. Incorporation could be detected by a mass shift of 9 Da, due to the loss of one *α* –deuterium. As expected derivatives with

the starter of myxovalargin A show the respective mass shift, while myxovalargin B and C do not integrate leucine. Further, myxovalargin E with the dehydrated starter unit is incorporating leucine as building block.

Table S3-3: The incorporation of d10-leucine in different myxovalargins.

Myxovalargin	M_r	Incorporation
Mxv A	1676	Yes
MxvB	1662	No
MxvC	1648	No
MxvE	1674	Yes
MxvG	1690	Yes
MxvH	1660	Yes

For myxovalargins with the isobutyryl starter like myxovalargin B and C, it is postulated to originate from valine. The incorporation of valine as precursor for the isobutyryl starter could be demonstrated by the determination of valines incorporated in the completely d8-valine labelled myxovalargin. Myxovalargin B shows an additional mass shift regarding to one valine, while myxovalargin C incorporates two additional valines. By fragmentation patterns, in both myxovalargins the starter can be determined as valine derived. Myxovalargin B shows a mass shift of minus 7 Da compared to myxovalargin A although the unlabelled myxovalargin B shows a difference of minus 14 Da due to its missing CH_2 in the starter unit. Therefore, an additional valine with + 7 Da, is incorporated. Myxovalargin C on the other hand, which holds an additional valine in the starter plus an additional valine instead of the isoleucine shows a mass shift of only +14 Da compared to its unlabelled form, which is 28 Da smaller than myxovalargin A. Thus, the difference between labelled myxovalargin C to myxovalargin A is 14 Da.

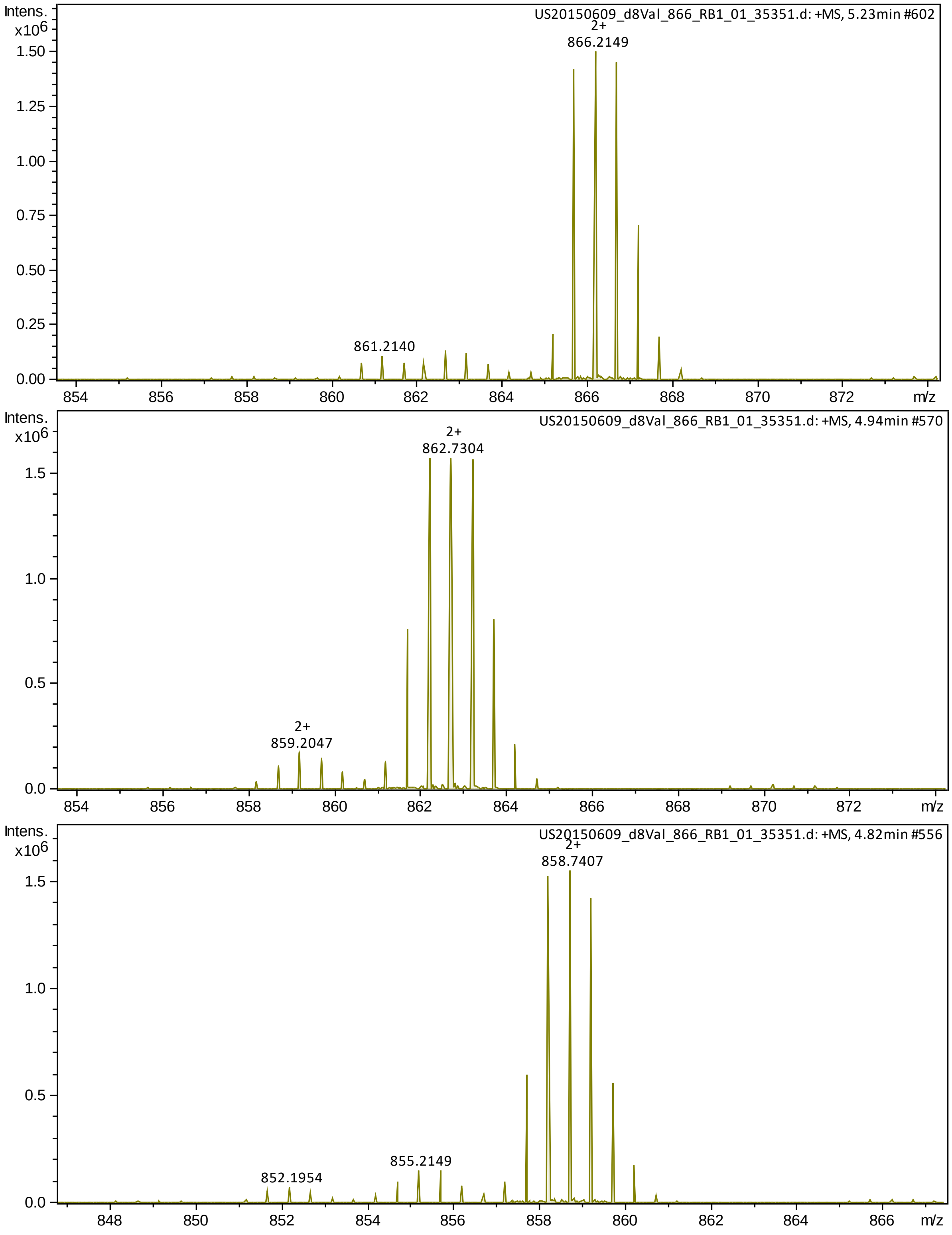

Figure S3-12: Isotope pattern of myxovalargins, produced by Ccc1071 cultivated in C^{13} medium. The upper panel shows myxovalargin A with a mass of $[M+2H]^{2+}$ 866.2 m/z. Myxovalargin B in the second channel shows a shift of 7 Da (3.5 m/z), which is due to its lower mass of minus 14 Da, but on the other hand an additional valine incorporated (+ 7 Da). Myxovalargin C incorporates two additional valines (+ 14 Da), but is 28 Da smaller than myxovalargin A, thus a mass shift of approximately 13 Da (6.5 m/z) appears.

Since valine and leucine could be identified to serve as precursor for branched chain ketoacid starter, which are incorporated in high yields, we probed the incorporation of isoleucine as alternative precursor. Isoleucine-d10 was fed to the wildtype strain and compared to the wildtype. The incorporation experiment also corroborates structural assignments of myxovalargin derivatives: A single isoleucine integration was visible in case of all myxovalargins except of myxovalargin C, which obtains no isoleucine moiety but valine, proving the position of the additional valine in myxovalargin C. On the other hand, myxovalargin G shows an additional shift for a second isoleucine moiety, which was found by MS fragmentation to be a hydroxy isoleucine instead of a hydroxy valine. Furthermore, within the myxovalargin A peak with a tendency to a shorter retention time, an additional pattern for a second incorporated isoleucine can be observed. Thus, this compounds peak overlaps completely with the actual myxovalargin A. They are isobaric under usual conditions, as both starters exhibit the same atomic composition. Since the second isoleucine is not dehydrogenated it leads to a mass shift of + 9 Da, whereas the regular isoleucine at the dehydro-isoleucine position gives a shift of + 8 Da. In summary this myxovalargin derivative named myxovalargin K shows a mass shift of + 16 Da in the double labelled compound. Incorporation of a second isoleucine therefore can be observed for the appearance of the mass $[M+2H]^{2+}$ 847.5770 m/z, but also by the shift and yield increase of the single labeled myxovalargin incorporating only the + 9 Da isoleucine as starter leading to a mass of $[M+2H]^{2+}$ 843.5542 m/z compared to $[M+2H]^{2+}$ 843.0506 m/z for the + 8 Da dehydro isoleucine incorporation. These findings led to the identification of myxovalargin K with the isoleucine starter instead of the leucine derived isovaleryl starter. It has to be noted that this concludes a mixture of myxovalargin A and K, when they are purified. Due to their chromatographic comparable behavior and the same mass at an assumed same stereochemistry, these compounds can only be distinguished by this feeding experiment.

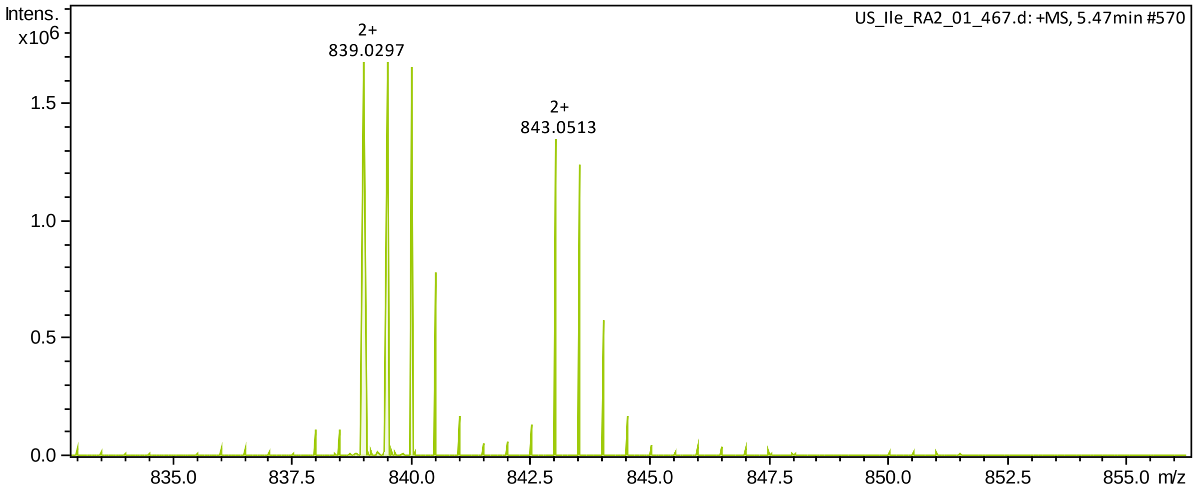

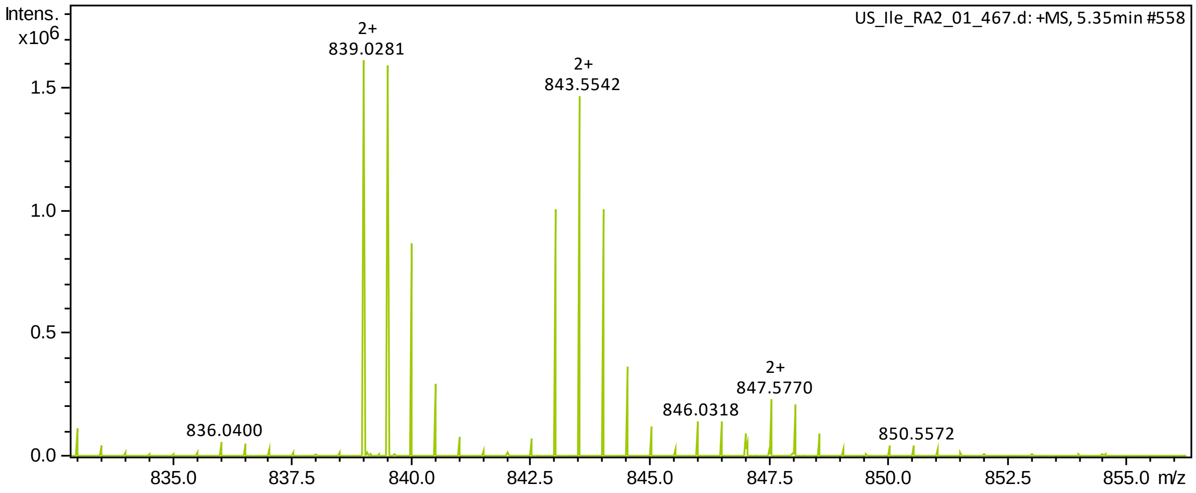

Figure S3-13: The upper panel shows the distribution pattern of Myxovalargin A at a retention time of 5.47 min, while the lower panel shows the pattern at 5.35 min. The additional incorporation of a second isoleucine with $[M+2H]^{2+}$ 847.5770 m/z, + 9 Da or 4.5 m/z compared to single labelled myxovalargin A (843.0514 m/z), can be observed. Furthermore, the mono labeled myxovalargin shows primarily a mass of $[M+2H]^{2+}$ 843.5542 m/z, which corresponds to the mass shift of + 9 Da (4.5 m/z), when integrating isoleucine as starter unit and not as dehydro isoleucine (+ 8 Da; 4 m/z).

This promiscuity of incorporation of different branched chained keto acids partially explains the vast amount of different myxvovalargins and the appearance of several peaks with comparable or identical chemical masses throughout the chromatographic region, where the myxovalargins elute. However, the results of bioactivity screening showed the superiority of myxovalargin A against all other derivatives.

4. On the stereochemical configuration of Myxovalargin A

A major discrepancy between the results and the interpretation of the initial stereochemical structure determination of myxovalargin by Steinmetz et al. in 1987 and the genetic analysis of the biosynthetic gene cluster became evident, when the sequence of the myxovalargin biosynthesis cluster was fully identified[8]. Module 7, which should incorporate L-valine according to the initial stereochemical assignment exhibits an epimerization domain while module 10, which should result in D-valine did not contain an epimerization domain. Epimerization domains are common enzymes in NRPS assembly lines changing stereocenters of carrier protein coupled proteinogenic L amino acids to a D-configuration during the assembly process. Therefore, these domains are present as expected in modules 4, 5, 6, 9, 11, 13, 14. Modules 1, 2, 3, 8, 12, which lead to L or dehydro amino acids do not contain an E domain. Sequence alignment of all E domains did not show any unusual signs, which would suggest an inactivity of the enzymes.

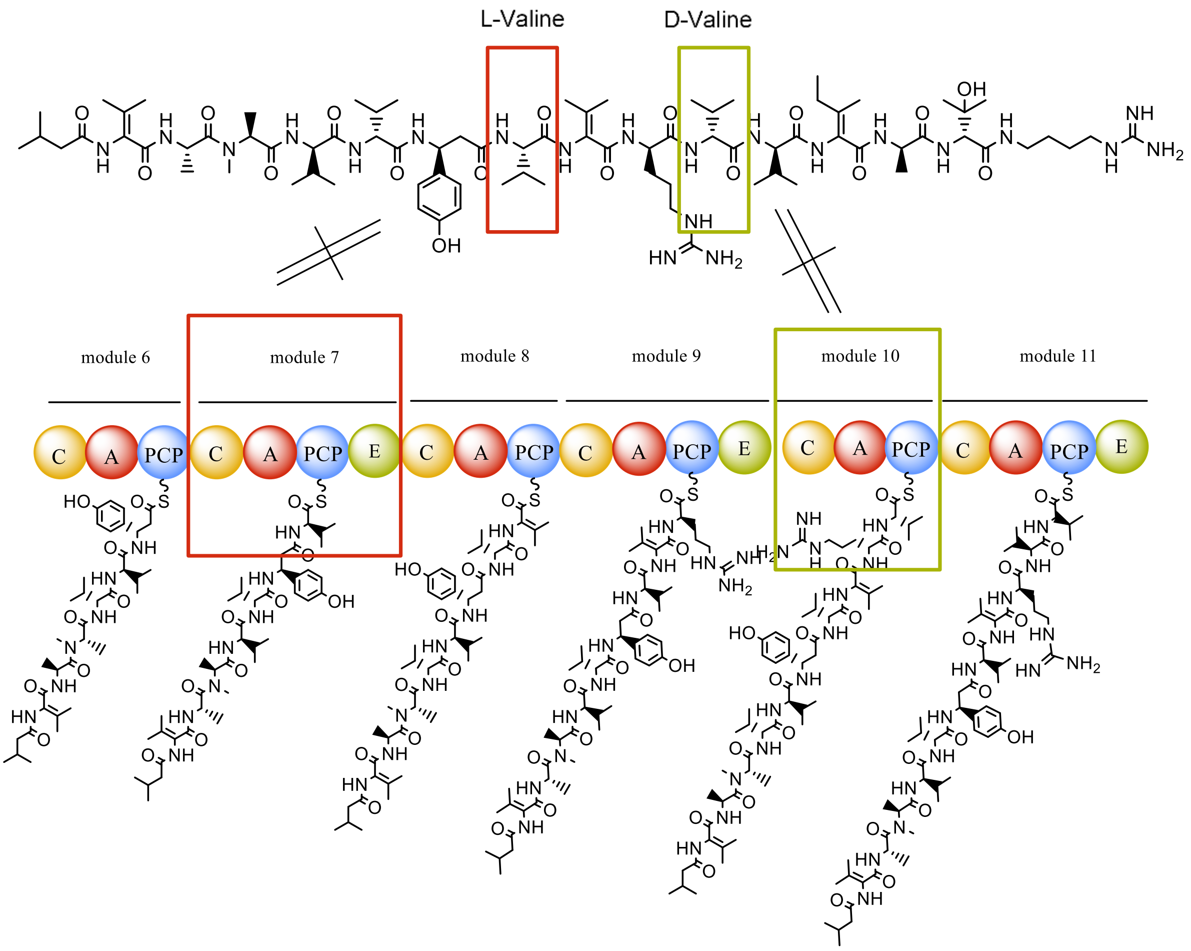

Figure S4-1: Discrepancy between assigned stereochemistry of myxovalargin A in literature and the sequence of the myxovalargin cluster at module 7 and module 10. Module 7 exhibits an epimerization domain, which suggests a D-valine in position 7, while module 10 lacks an epimerization domain, which is contrary to the assigned D-Valine in position 10 of myxovalargin A

Nevertheless, since the region of module 8 – 10 has shown to impede assembly of sequencing scaffolds due to its repetitive sequence sections, a verification of these scaffolds was challenging. Assembly of Illumina and Roche454 sequencing data already showed the final domain organization, but still appeared to leave two gaps in highly repetitive regions. The final sequence was achieved after Pacific Bioscience sequencing, which spans the repetitive regions. Because of the described assembly difficulties, gene clusters identified in strains MCy8286, MCy9171 and MCy5730 showed gaps at similar positions, nevertheless assembly of MCy9171 scaffolds suggests the same domain organization. Due to highly repetitive regions in module 7 and module 11, alignments of Illumina sequencing and Roche 454 sequencing were not unequivocally indicating the genomic sequence of the myxovalargin cluster. Roche 454 and especially Illumina sequencing are techniques, which both yield relatively short sequence scaffolds that need to be aligned to result in a consecutive sequence compared to PacBio sequencing. The repetitive regions left a rest of uncertainty about

a correct alignment, especially with regards to the observed inconsistency. Though PacBio sequencing provided a high-quality end-to-end sequence, southern blot experiments were carried out to verify the correct alignment. Probes within the regions of module 7 (probe E) and module 10 (probe 10) were chosen. Due to the size of an epimerization domain of approximately 2000 bp, the presence or absence of this domain can be clearly identified by this technique. Nevertheless, the repetitive regions limit the suitability of different restriction enzymes. Three enzymes were chosen to yield suitable scaffolds to underline the sequence alignment: *Not*I, *Kpn*I, and *EcoR*I. Southern Blot experiments of module 10 with probe 10 and three different restriction digests were successfully performed and confirm the lack of an epimerization domain in module 10 in the genome of the producer. The results for probe E binding to the epimerization domain in module 7 confirm with two digests the expected scaffolds. The third digest with the restriction enzyme *Not*I, which would yield a large scaffold of >35 kbp only gives a weak signal above the 8576 bp marker band. Due to the large, expected scaffold and the competitive binding of probe E in the repetitive homolog in module 11, this result is plausible. This result also does not support the presence of an E domain in module 7. Thus, the southern blot experiments support the sequence assembly in this region as described in this study.

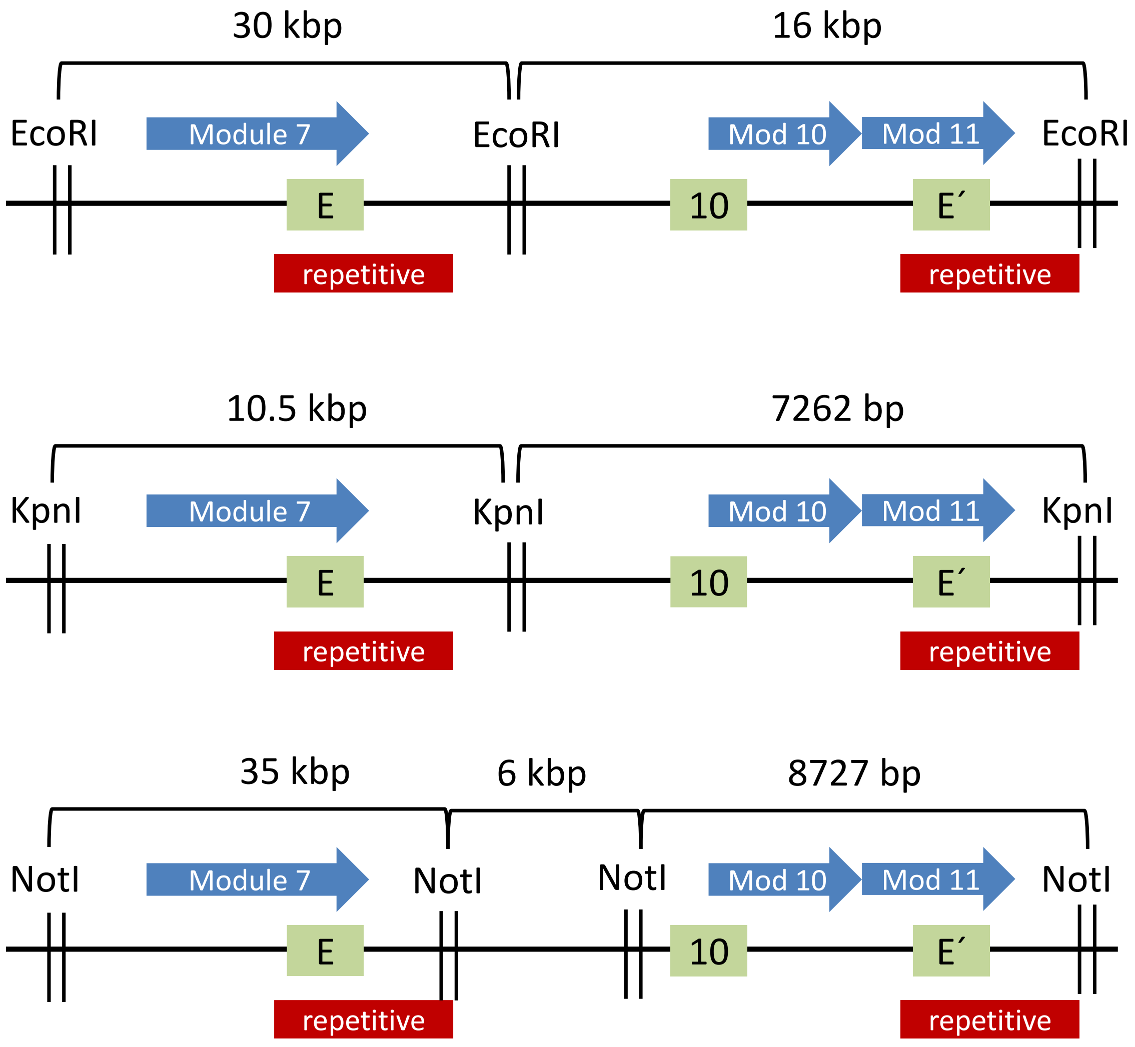

Figure S4-2: Restriction digest of genomic DNA from Ccc1071. The green squares show the binding region of the probes E and 10. E´ is a putative second binding region for E, due to high sequence similarity, marked in red as repetitive region. The blue arrows show the affected modules, while the discrepancy between sequence and stereochemistry is appearing in module 7 (sequence has E domain, though stereochemistry is L-valine) and module 10 (sequence lacks E domain, though stereochemistry is D-valine). The cutting sites of the chosen restriction enzymes and the expected scaffolds are given.

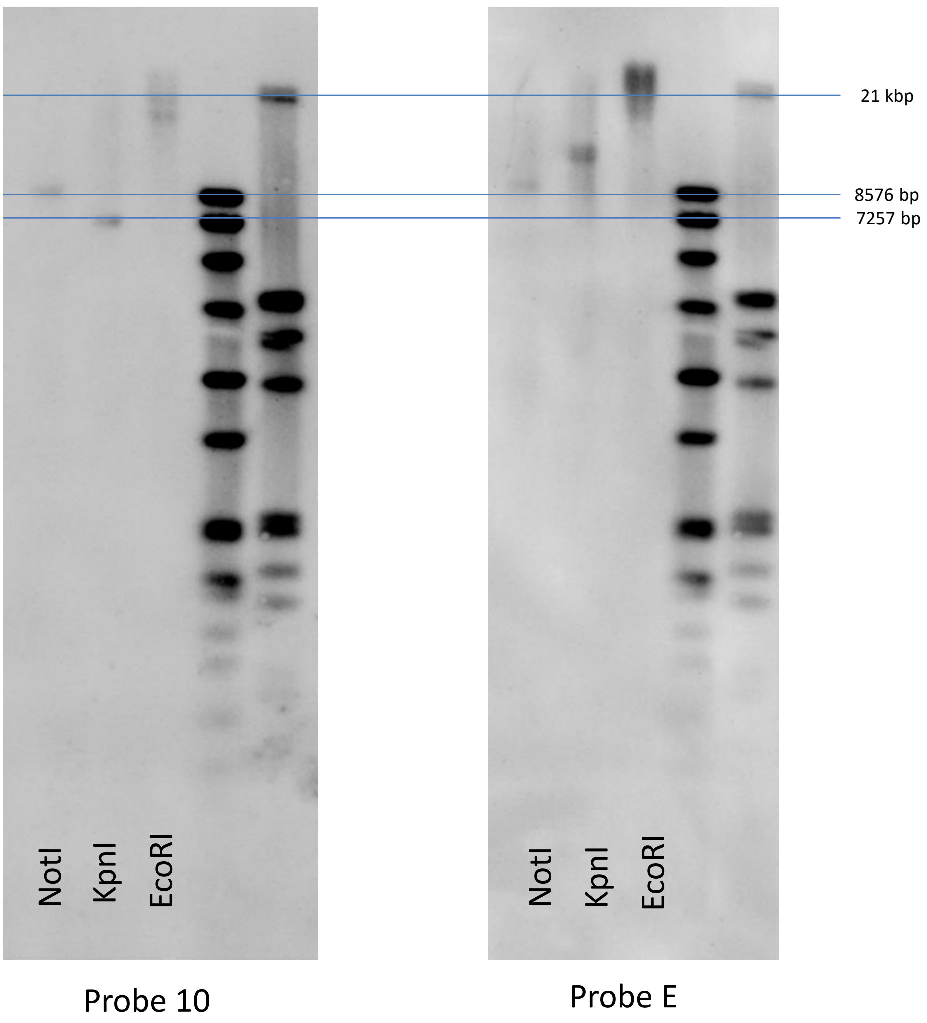

Figure S4-3: Southern Blot of probe 10 and probe E to confirm the sequence assembly of the regions between module 7 and 10. The pattern for probe 10 fits with the estimated scaffolds: *Not*I: 8727 bp, *Kpn*I: 7262 bp, *EcoR*I: 16 kbp. The pattern for probe E fits for *Kpn*I: 7262 bp, *EcoR*I 35 kbp, but not for *Not*I appr. 8800 bp. This can be explained by binding to the repetitive region in module 11. All digests support the sequence assembly.

As the sequence by PacBio sequencing and additional Southern Blot was confirmed, the condensation domains in this region were further investigated. It was shown that condensation domains are related regarding their specificity for educts they condensate. This investigation was in particularly interesting, since it has been reported that condensation and epimerization domains are closely related, and even bifunctional enzymes (C/E domains) exist. However, all C domains present in the myxovalargin assembly line belong to the DcL and the LcL groups (**Figure S4-4**). DcL domains condensate D amino acids from the previous module with the L amino acid attached at the PCP domain of the same module the C domain is located in; LcL domains analogously condensate L amino acids with L amino acids. Thus, no unusual

C/E mechanism is expected. The C domain of module 1, although grouping to LcL, is closely related to starter incorporating domains, which is reasonable, since this domain condensates the precursor to the first building block in module 1. Furthermore, all other C domains except of modules 8 and 11 fit with the stereochemical assignments of the original structure elucidation. On the other hand, all C domain functionalities concur with the presence of epimerization domains in the cluster sequence. The critical C domain of module 8 belongs to the DcL clade, indicating that module 7 must form a D amino acid, while C domain of module 11 groups with LcL domains suggesting module 10 to build a L-amino acid. Thus, the C domain comparison supports the revision of the stereochemistry.

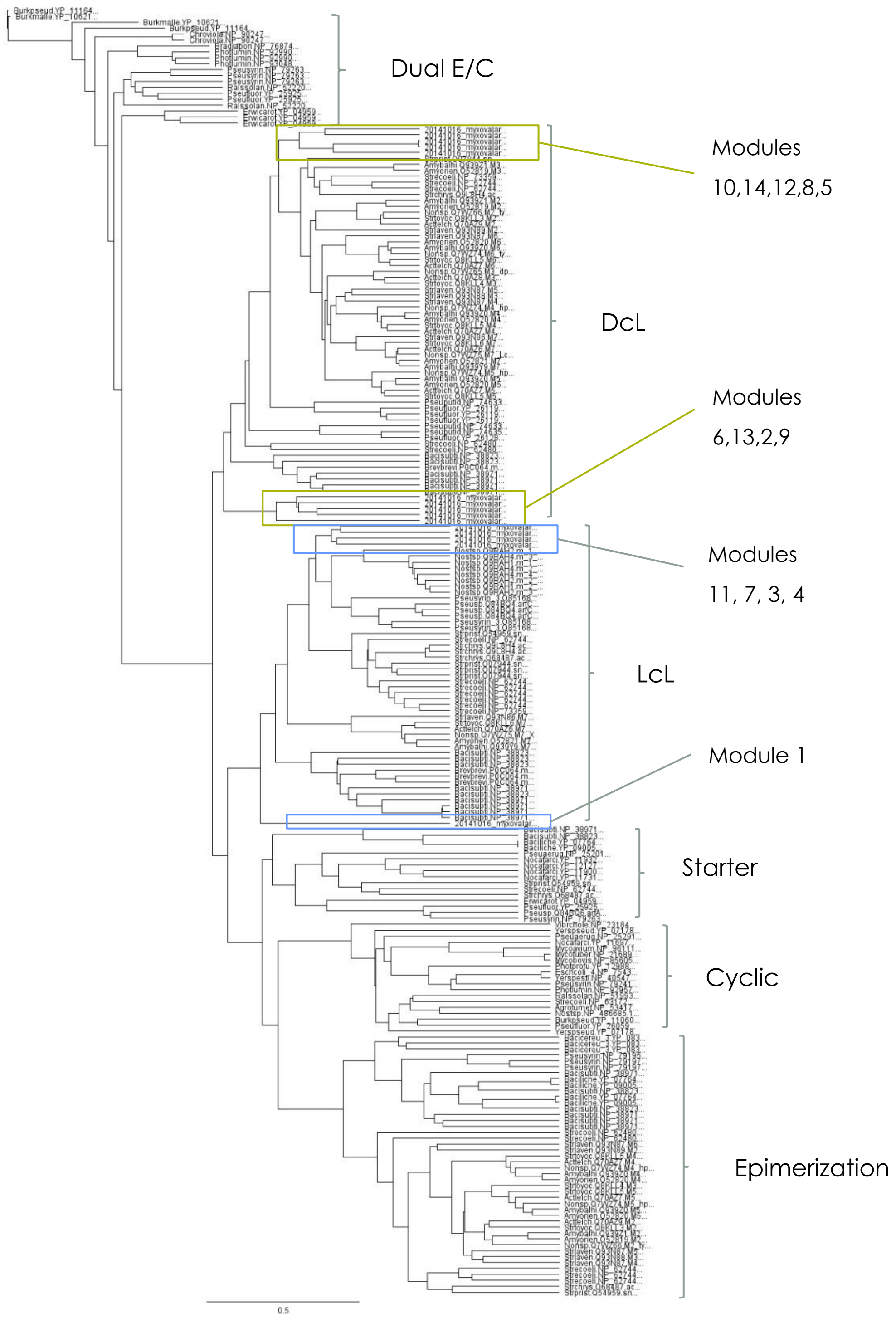

Figure S4-4: Tree of C domains in the myxovalargin biosynthesis cluster. All C domains group to the DcL and LcL group which condensate D with L or L with L amino acids, respectively. The predictions mostly correspond to functional reactions, anticipated by the chemical structure.

The confirmation of the myxovalargin cluster sequence and its discrepancy with the stereochemical assignment, which was demonstrated by *in silico* characterization, was further underlined by combined approaches of chemical and biological tools on the actual molecule.

The conversion of the stereocenter at the α-carbon by the epimerization domain is composed of an elimination of the hydrogen atom leading to a sp2 hybridization, followed by an addition of a hydrogen atom stereochemically directed by the epimerization domain to form the D-amino acid. This elimination-addition reaction leads to the substitution of the hydrogen atom at the stereocenter. Therefore, it is possible to detect the difference of an incorporated amino acid, which is labelled by deuterium at the α-carbon, in terms of their D or L-configuration after epimerization.[25] The elimination of deuterium and subsequent addition of hydrogen results in a 1 Da mass shift. In case of myxovalargin, the presence of 8 – 10 valines (depending on myxovalargin derivatives) made a direct approach by feeding of labeled valine to the culture in regular medium with a full set of amino acids in the nitrogen source impossible. Although the calculated fully labelled myxovalargin could be detected, it would be overlaid by isotope peaks of less labelled molecules, which hinders an assignment of labelling to the structural moieties. Therefore, a sophisticated minimal medium was composed where valine was solely substituted as d8-valine on basis of a reported minimal medium for *Myxococcus xanthus* [26] and by including results from microbiological experiments (**Figure S4-5**). Hence, fully labelled myxovalargin derivatives could be produced. By MS-MS fragmentation and known detectable fragments from previous high resolution MS data acquired by Orbitrap direct infusion experiments with unlabeled myxovalargin derivatives, it was possible to directly compare fully labelled and unlabeled derivatives. Beside the identification and confirmation of structural assignments by detection of incorporated valines at *e.g.*, the starter positions, additional confirmation of the stereochemical configuration could be achieved.

US_TPM17

100 mM L-Arginine
100 mM L-Alanine
100 mM L.-Asparagine
50 mM L-Cystine
50 mM L-Glycine
50 mM L-Histidine
100 mM L-Isoleucine
500 mM L-Leucine
100 mM L-Lysine
100 mM L-Methionine
100 mm L-Phenylalanine
100 mM L-Serine
100 mM L-Threonine
100 mM L-Tryptophane
200 mM L-Tyrosine
100 mM d8-Valine
10 mM Tris-HCl (pH 7.6)
1 mM KH_2PO_4-K_2PO_4 (pH 7.6)
0.8 mM $MgSO_4$
1 mg/mL $(NH_4)_2SO_4$
8 µg/mL FeEDTA
pH 7.0

Figure S4-5: US_TPM17 medium with 100 mM d8-valine replacing L-valine.

Feeding of the deuterium labelled valine leads to a mass increase of +8 Da for L-valine, while D-valine results in a +7 Da mass shift caused by substitution of the deuterium ion at the α-carbon by hydrogen. The dehydrogenated valines show a difference of + 6 Da due to their loss of two deuterium ions. Though not all scaffolds of a linear fragmentation by peptide bond opening can be observed, most of the y and b fragments are typically observed. The difference of L-valine compared to D-valine at the critical positions val7 and val10 can therefore be observed by the fragments y8, y7 and y6, which contain val10, but not val7 and by the fragments b10, b9, b8 harboring val7, but not val10. As b9 and b8 are not observed for myxovalargins, especially b10, y8, y7, y6 were investigated. Those fragments confirm the stereochemical revision of

val7 and val10. Furthermore, the correct identification of all other listed b and y fragments shows the suitability of this approach and supports the stereochemical as well as the structural assignment of the other valine building blocks.

The comparison of fully d8-valine labeled myxovalargin A (**Figure S4-6**) as well as myxovalargin B (**Figure S4-7**) and myxovalargin C (**Figure S4-8**) against the respective unlabeled natural occurring myxovalargin A unequivocally confirms the stereochemical assignment of D-valine in position val7 and L-valine in position val10. Thus, the stereochemistry of myxovalargin A-C needs to be revised as suggested by the genomic sequence-based approach and as confirmed by the chemical determination.

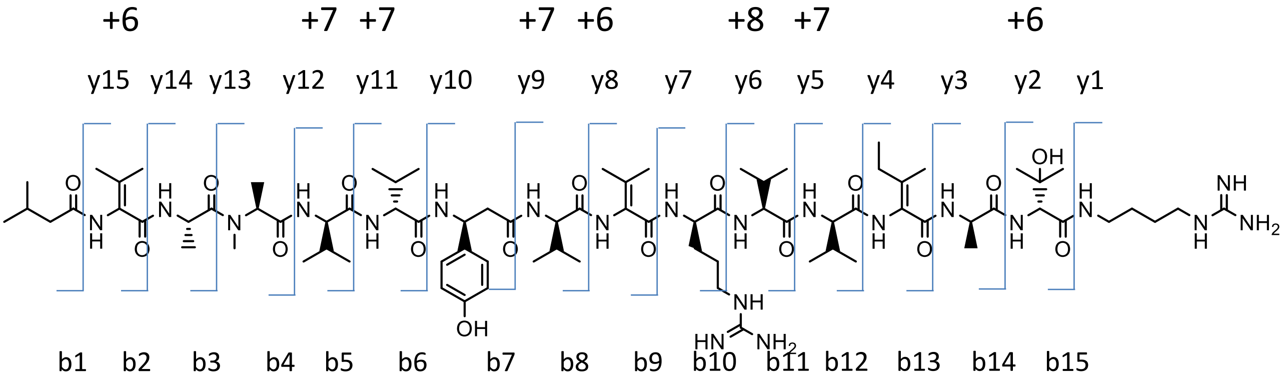

Fragment	MxvA (m/z)	d8 Val (m/z)	Mass shift	Fragment	MxvA (m/z)	d8 Val (m/z)	Mass shift
b1	nd	nd	nd	y15	810.00996	nd	nd
b2	nd	nd	nd	y14	1496.94739	nd	nd
b3	253.15433	259.1931	6	y13	1424.9093	nd	nd
b4	338.20697	344.2462	6	y12	1339.85632	1388.1685	48
b5	437.27594	450.3584	13	y11	1240.78943	1282.0391	41
b6	536.3429	556.4700	20	y10	1141.72009	1175.9459	34
b7	nd	nd	nd	y9	nd	nd	nd
b8	nd	nd	nd	y8	879.58759	906.7569	27
b9	nd	nd	nd	y7	782.53497	803.6676	21
b10	1051.62927	1084.8395	33	y6	626.43378	647.5637	21
b11	1150.69629	1191.8191	41	y5	527.36523	540.4480	13
b12	1249.76611	1298.1736	48	y4	428.28727	434.3373	6
b13	1360.83398	1409.1454	48	y3	317.22919	323.2676	6
b14	1431.8723	nd	nd	y2	246.99221	nd	nd
b15	nd	nd	nd	y1	nd	nd	nd

Figure S4-6: Fragmentation pattern of myxovalargin A and detected corresponding fragments of the fully labelled myxovalargin A by d8-valine feeding. Myxovalargin A incorporates 8 valines, thereby showing an overall mass shift of 54 Da. The expected mass shifts (Da) for deuterium labelled valine incorporation are shown above the molecule. The fragments of b10, y6, y7 and y8 which only obtain val7 or val10, respectively confirm the stereochemical revision. All other listed fragments fit with the expected shifts and highlight the suitability of the approach; nd = not detected.

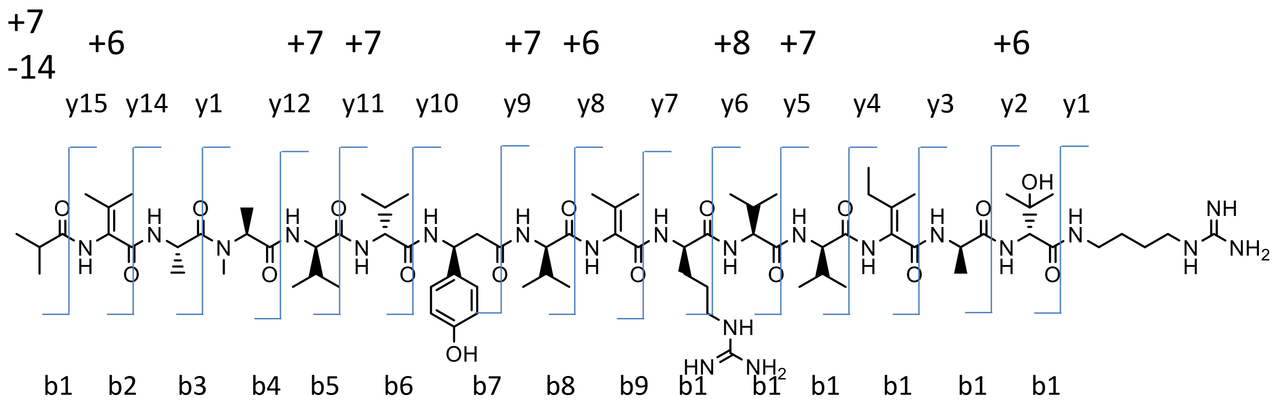

Fragment	MxvA (m/z)	d8 Val (m/z)	Mass shift	Fragment	MxvA (m/z)	d8 Val (m/z)	Mass shift
b1	nd	nd	nd	y15	810.00996	nd	nd
b2	nd	nd	nd	y14	1496.94739	nd	nd
b3	253.15433	252.2224	-1	y13	1424.9093	nd	nd
b4	338.20697	337.2751	-1	y12	1339.85632	1388.1695	+48
b5	437.27594	nd	nd	y11	1240.78943	1282.0556	+41
b6	536.3429	548.5170	+12	y10	1141.72009	1175.9398	+34
b7	nd	nd	nd	y9	nd	nd	nd
b8	nd	nd	nd	y8	879.58759	906.7613	+27
b9	nd	nd	nd	y7	782.53497	803.6677	+21
b10	1051.62927	1084.8395	+33	y6	626.43378	647.5680	+21
b11	1150.69629	1191.8191	+41	y5	527.36523	540.4517	+13
b12	1249.76611	1291.1083	+41	y4	428.28727	434.3355	+6
b13	1360.83398	1401.1738	+41	y3	317.22919	323.2685	+6
b14	1431.8723	nd	nd	y2	246.99221	nd	nd
b15	nd	nd	nd	y1	nd	nd	nd

Figure S4-7: Fragmentation pattern of myxovalargin A and detected corresponding fragments of the fully labelled myxovalargin B by d8-valine feeding. Myxovalargin B incorporates 9 valines caused by an additional valine as starter, thereby showing an overall mass shift of 61 Da. The expected mass shifts (Da) for each incorporated d8-valine against myxovalargin A are indicated above the molecule. The shifts of b3 – y13 show a difference of -7 Da compared to the shifts of myxovalargin A due to one additional valine incorporated (+7 Da) and subtracting the mass loss for a starter with one CH2 extender unit less (- 14 Da). The fragments of b10, y6, y7 and y8 which only obtain val7 or val10, respectively confirm the stereochemical revision. All other listed fragments fit with the expected shifts and highlight the suitability; nd = not detected

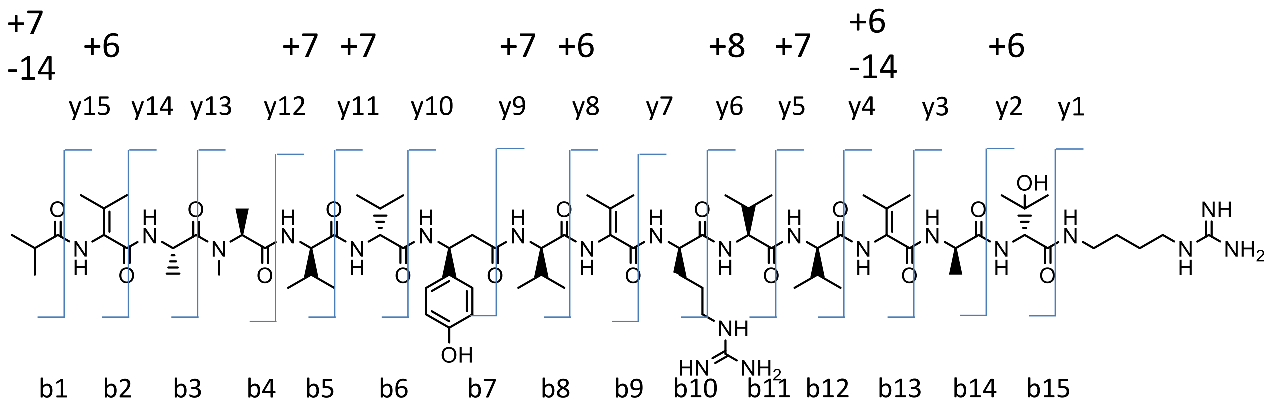

Fragment	MxvA (m/z)	d8 Val (m/z)	Mass shift	Fragment	MxvA (m/z)	d8 Val (m/z)	Mass shift
b1	nd	nd	nd	y15	810.00996	nd	nd
b2	nd	nd	nd	y14	1496.94739	nd	nd
b3	253.15433	252.2223	-1	y13	1424.9093	nd	nd
b4	338.20697	337.2742	-1	y12	1339.85632	nd	nd
b5	437.27594	443.2178	+6	y11	1240.78943	nd	nd
b6	536.3429	556.4700		y10	1141.72009	nd	nd
b7	nd	nd	nd	y9	nd	nd	nd
b8	nd	nd	nd	y8	879.58759	898.7832	+19
b9	nd	nd	nd	y7	782.53497	795.6867	+13
b10	1051.62927	1084.8395	+33	y6	626.43378	639.5884	+13
b11	1150.69629	1191.8191	+41	y5	527.36523	532.4694	+5
b12	1249.76611	1298.1736	+48	y4	428.28727	426.3593	-2
b13	1360.83398	1409.1454	+48	y3	317.22919	323.2684	+6
b14	1431.8723	nd	nd	y2	246.99221	nd	nd

Figure S4-8: Fragmentation pattern of myxovalargin A and detected corresponding fragments of the fully labelled myxovalargin C by d8-valine feeding. Myxovalargin C incorporates 10 valines, an additional valine as starter and an additional valine instead of isoleucine, thereby showing an overall mass shift of 67 Da. The expected mass shifts (Da) for each incorporated d8-valine against myxovalargin A are indicated above the molecule. The shifts of y4 – y8 show a difference -8 Da compared to the shifts of myxovalargin A due to one additional dehydro valine incorporated (+6 Da) and subtracting the mass loss for substituting dehydro isoleucine (- 14 Da). For a fragments the same shifts as described for myxovalargin B apply. The fragments of b10, y6, y7 and y8 which only obtain val7 or val10, respectively confirm the stereochemical revision. All other listed fragments fit with the expected shifts and highlight the suitability of this approach; nd = not detected.

Myxovalargin – overview of structural variants

The myxovalargins are a predominant compound class in the chromatogram of MCy6431 as well as in other myxovalargin producers. The peptide structure of all identified myxovalargins is highly preserved. Few substitutions by similar building blocks could be observed. The interchange of isoleucine or leucine by valine in several

positions is the most prevalent observation. Especially the starter unit offers a certain flexibility for different substrates, this is supported by the precursor directed feeding experiments, which allowed the incorporation of different chemically related substrates.

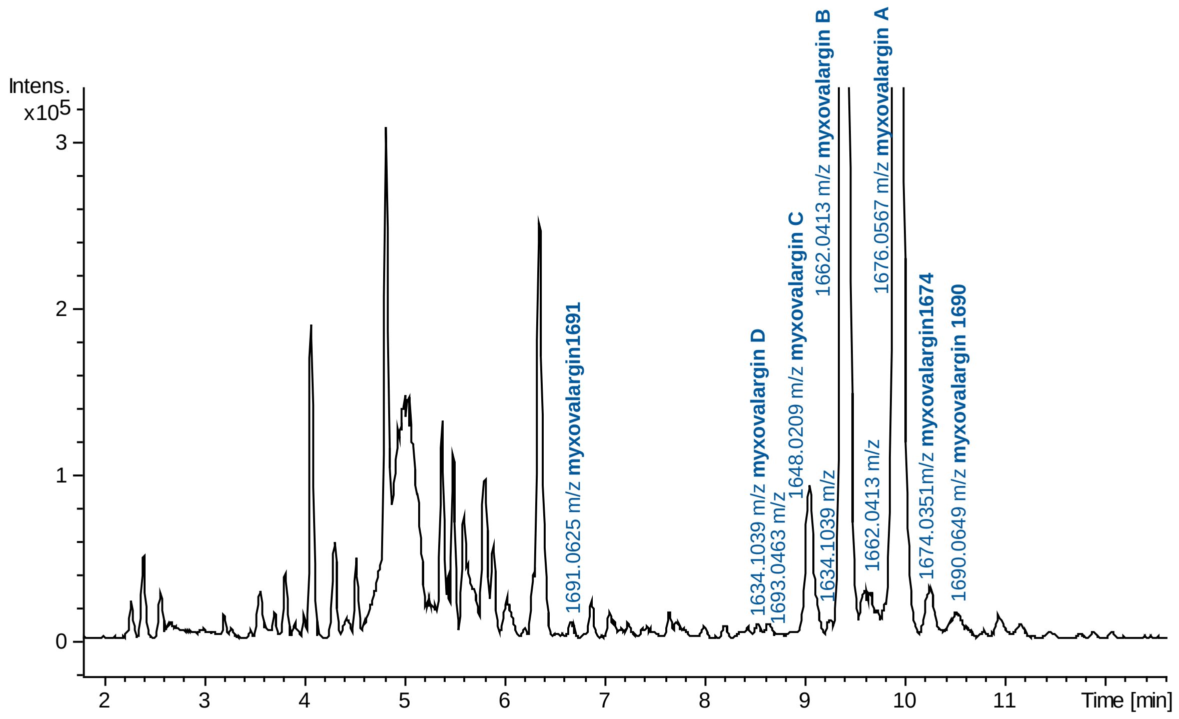

Figure S4-9: Chromatoram of MCy6431 in AMB + FeEDTA Medium. The myxovalargins elute in a time frame of approximately 6.5 – 11 minutes.

Myxovalargin A: Myxovalargin A with a mass of 1676 Da is the predominant myxovalargin in all myxobacterial strains. The structure of myxovalargin A was elucidated and confirmed by MS and NMR. The stereochemistry is determined and confirmed.

Myxovalargin B: Myxovalargin B is the second most dominant derivative in all myxobacterial strains eluting prior to myxovalargin A. Cultivation parameters allow a direct influence on the production rate of myxovalargin B, allowing to achieve a comparable yield as A. With a mass of 1662 Da it lacks 14 Da compared to myxovalargin A. By MS fragmentation the mass loss could be identified at the starter unit. By feeding experiments and MS fragmentation the incorporation of valine could be identified in this position. Using this experimental evidence and biosynthesis considerations the myxovalargin B structure is elucidated. Though a full stereochemical characterization was not performed for myxovalargin B, the abundant occurrence and biosynthesis considerations imply that myxovalargin B shows the same stereochemistry as myxovalargin A.

Myxovalargin C: Myxovalargin C is another common myxovalargin eluting prior to A and B. With a mass of 1448 it lacks 28 Da to A and 14 Da to B. MS fragmentation narrows 14 Da mass loss to the starter unit and 14 Da mass loss to the dehydro-isoleucine moiety in myxovalargin A. By feeding experiments with valine, also here the incorporation at the starter unit could be confirmed. Furthermore, a valine incorporation instead of the isoleucine could be verified. Thus, the constitution of myxovalargin C is determined. Although also for myxovalargin C a full stereochemical characterization was not performed, the abundant occurrence and biosynthesis considerations imply that myxovalargin C shows the same stereochemistry as myxovalargin A.

Myxovalargin D: While myxovalargin D was one of the first identified myxovalargins and was found in sufficient amount in *Myxococcus fulvus* Mxf65 (MCy8286) eluting prior to myxovalargin C, it is a minor derivative in MCy6431 and only produced in very low amounts. Myxovalargin D with a mass of 1434 Da shows a loss of 32 Da compared to Myxovalargin A. This is equal to 3 CH_2 extender units. MS fragmentation indicates a loss of 28 Da at the starter unit and 14 Da at the dehydro isoleucine moiety. Myxovalargin D therefore is assumed to contain a dehydro valine moiety at the isoleucine position. However, the starter unit of myxovalargin D remains unclear. Although the loss of 28 Da suggests a loss of 2 CH_2 extender, as the starter could not be determined, any assumption on the putative starter is theoretical.

High resolution MS fragmentation of myxovalargins

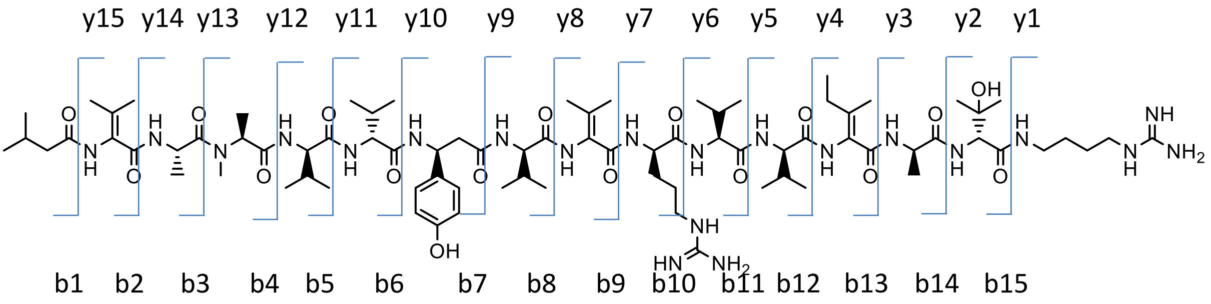

Figure S4-10: expected b and y fragments according to a linear fragmentation of the peptide by cleavage of the terminal amino acid from C-terminus and N-terminus, respectively.

Table S4-1: Detected fragments by high resolution MS fragmentation of the myxovalargin derivatives Myxovalargin B (MxvB) to Myxovalargin K (MxvK) and their shift in comparison to the fragments of Myxovalargin A (MxvA). [nd = not detected]

Fragment	MxvA	MxvB	Shift	MxvC	Shift	MxvD	Shift

b1	nd	nd	nd	nd	nd	nd	nd
b2	nd	nd	nd	nd	nd	nd	nd
b3	253.15433	239.13837	-14	239.13852	-14	225.1235	-28
b4	338.20697	324.19119	-14	324.19086	-14	310.1763	-28
b5	437.27594	429.25961	-14	n.d.	/	n.d.	/
b6	536.3429	522.32703	-14	522.32672	-14	nd	nd
b7	nd	nd	nd	nd	nd	nd	nd
b8	nd	nd	nd	nd	nd	nd	nd
b9	nd	nd	nd	nd	nd	nd	nd
b10	1051.62927	1037.61243	-14	1037.61121	-14	1023.6000	-28
b11	1150.69629	1136.69944	14	nd	nd	nd	nd
b12	1249.76611	1235.74915	14	1235.75256	14	1221.7441	-28
b13	1360.83398	1346.81775	14	1332.80225	28	1318.7933	nd
b14	1431.8723	1417.854149	14	1403.83630	28	nd	nd
b15	nd	nd	nd	nd	nd	nd	nd
y15	810.00996	nd	nd	nd	nd	nd	nd
y14	1495.94739	1495.94519	0	nd	nd	1481.9351	-14
y13	1424.9093	1424.94519	0	nd	nd	1410.9010	-14
y12	1339.85632	1339.85535	0	1325.83887	-14	1325.8468	-14
y11	1240.78943	1240.78821	0	1226.78015	-14	1226.7731	-14
y10	1141.72009	1141.71899	0	1127.70557	-14	1127.7063	-14
y9	nd	nd	0	nd	nd	nd	nd
y8	879.58759	879.58728	0	nd	nd	nd	nd
y7	782.53497	782.55467	0	768.51855	-14	768.5183	-14
y6	626.43378	626.43347	0	612.41718	-14	626.4272	-14
y5	527.36523	527.36493	0	513.34844	-14	513.3496	-14
y4	428.28727	428.29727	0	414.28125	-14	428.2811	-14
y3	317.22919	317.22897	0	nd	nd	317.2301	-14
y2	246.99221	246.64648	0	nd	nd	nd	nd
y1	nd	nd	nd	nd	nd	nd	nd

fragment	MxvA	MxvE	shift	MxvF	Shift	MxvG	Shift
b1	nd	nd	nd	nd	nd	nd	nd
b2	nd	nd	nd	nd	nd	nd	nd
b3	253.15433	251.1379	-2	268.1640	+15	253.1123	0
b4	338.20697	336.1905	-2	353.2170	+15	338.1306	0
b5	437.27594	nd	nd	nd	nd	nd	nd
b6	536.3429	nd	nd	551.3549	+15	536.2454	0
b7	nd	nd	nd	714.4139	nd	nd	nd
b8	nd	nd	nd	nd	nd	nd	nd
b9	nd	nd	nd	910.53925	nd	nd	nd
b10	1051.62927	1049.6122	-2	1066.6398	+15	1051.4943	0
b11	1150.69629	1148.6792	-2	n.d.	nd	1150.7410	0
b12	1249.76611	1247.7469	-2	1264.7818	+15	1249.7610	0
b13	1360.83398	1358.8165	-2	1375.8418	+15	1360.7780	0

b14	1431.8723	1429.8544	-2	1446.3789	+15	1431.5782	0
b15	nd	nd	nd	nd	nd	1560.7540	+14
y15	810.00996	nd	nd	nd	nd	nd	nd
y14	1495.94739	1495.9439	0	1495.94348	0	1509.9647	+14
y13	1424.9093	1424.9072	0	1424.9055	0	1438.6038	+14
y12	1339.85632	1339.8546	0	1339.8538	0	1353.8084	+14
y11	1240.78943	1240.7882	0	1240.7850	0	1254.8623	+14
y10	1141.72009	1141.7188	0	1141.7198	0	1155.7403	+14
y9	nd	nd	nd	nd	nd	nd	nd
y8	879.58759	879.5867	0	879.5880	0	893.6996	+14
y7	782.53497	782.5334	0	782.5341	0	796.5099	+14
y6	626.43378	626.4330	0	626.4330	0	640.3928	+14
y5	527.36523	527.6942	0	527.3642	0	546.3624	+14
y4	428.28727	428.2965	0	428.2958	0	442.3987	+14
y3	317.22919	317.2283	0	317.2287	0	331.3264	+14
y2	246.99221	nd	nd	246.2593	0	nd	nd
y1	nd	nd	nd	nd	nd	nd	nd

fragment	MxvA	MxvH	shift	MxvI	Shift	MxvJ	Shift
b1	nd	nd	nd	nd	nd	nd	nd
b2	nd	nd	nd	nd	nd	nd	nd
b3	253.15433	nd	nd	239.2185	-14	253.1449	0
b4	338.20697	338.2091	0	324.1295	-14	338.0612	0
b5	437.27594	437.2779	0	nd	nd	nd	nd
b6	536.3429	536.3476	0	522.0714	-14	nd	nd
b7	nd	nd	nd	nd	nd	nd	nd
b8	nd	nd	nd	nd	nd	nd	nd
b9	nd	nd	nd	nd	nd	nd	nd
b10	1051.62927	1051.6382	0	1037.4348	-14	1051.4927	0
b11	1150.69629	1150.7068	0	1136.6009	-14	1150.6816	0
b12	1249.76611	1249.7763	0	1233.7118	-14	1249.5070	0
b13	1360.83398	1360.8458	0	1348.4648	-12	1362.9725	+2
b14	1431.8723	nd	nd	1419.9084	-12	1433.2811	+2
b15	nd	nd	nd	nd	nd	nd	nd
y15	810.00996	nd	nd	nd	nd	nd	nd
y14	1495.94739	1479.9643	-16	1497.8822	+2	1497.7963	+2
y13	1424.9093	1408.9255	-16	1426.7817	+2	1426.7006	+2
y12	1339.85632	1323.8726	-16	1341.8357	+2	1341.8364	+2
y11	1240.78943	1224.8035	-16	1242.7562	+2	1242.6579	+2
y10	1141.72009	1125.7340	-16	1143.8298	+2	1143.6873	+2
y9	nd	nd	nd	nd	nd	nd	nd
y8	879.58759	nd	nd	881.5538	+2	881.513373	+2
y7	782.53497	766.5467	-16	784.5770	+2	784.5289	+2
y6	626.43378	nd	nd	628.4280	+2	628.5396	+2
y5	527.36523	511.3750	-16	529.4816	+2	529.3279	+2

y4	428.28727	412.3086	-16	430.1964	+2	430.47510	+2
y3	317.22919	301.2360	-16	317.4724	0	317.2895	0
y2	246.99221	nd	nd	nd	nd	nd	nd
y1	nd	nd	nd	nd	nd	nd	nd

fragment	MxvA	MxvL	shift
b1	nd	nd	nd
b2	nd	nd	nd
b3	253.15433	239.0288	-14
b4	338.20697	324.1874	-14
b5	437.27594	nd	nd
b6	536.3429	522.1089	-14
b7	nd	nd	nd
b8	nd	nd	nd
b9	nd	nd	nd
b10	1051.62927	1039.1877	-12
b11	1150.69629	1138.5553	-12
b12	1249.76611	1237.7727	-12
b13	1360.83398	1348.6334	-12
b14	1431.8723	1419.7814	-12
b15	nd	nd	nd
y15	810.00996	nd	nd
y14	1495.94739	1497.9204	+2
y13	1424.9093	1426.8767	+2
y12	1339.85632	1341.7972	+2
y11	1240.78943	1242.9379	+2
y10	1141.72009	1143.5996	+2
y9	nd	nd	nd
y8	879.58759	881.1407	+2
y7	782.53497	782.4567	0
y6	626.43378	626.3668	0
y5	527.36523	527.4862	0
y4	428.28727	428.3771	0
y3	317.22919	317.2504	0
y2	246.99221	nd	nd
y1	nd	nd	nd

5. Total synthesis – Experimental data

5.1. Materials and methods

Reagents and solvents: All non-aqueous reactions were carried out under an inert atmosphere (argon) with dried glassware, using standard techniques. Anhydrous solvents (MeCN, CH_2Cl_2) were obtained from a MB solvent purification system (MBRAUN) or commercial solvents were used. Petroleum ether (60 °C) and THF were distilled before application and triethylamine was dried over KOH and distilled as well. Commercial reagents were used as supplied.

Thin layer chromatography (TLC): Analytical thin-layer chromatography was performed on precoated aluminium-backed silica gel plates with a layer thickness of 0.2 mm. Visualization of the developed chromatogram was performed by UV absorbance (254 nm) and/or stained with aqueous potassium permanganate solution with subsequent heat treatment.

Flash column chromatography: Flash column chromatography was performed using mesh silica (grain size 40-63 μm), with the indicated solvent system according to the standard techniques. Alternatively, a BÜCHI purification system was applied containing two pump modules (C-605), a UV-Vis detector (C-630), a fraction collector (C-660) and the control unit C-620. The separation was performed with a Cartridge PP 12/150 column and a FC60 (60 x 20 mL) rack. The system was controlled via Sepacore control software.

Nuclear magnetic resonance (NMR) spectroscopy: NMR spectra were recorded on a BRUKER Ultrashield 500 MHz with Avance-III HD console, an Ascend 400 MHz with Avance-III console, an Ascend 400 MHz with Avance-III HD console, an Ultrashield 400 MHz with Avance-I console and an Ascend 600 MHz with Avance Neo console.

Chemical shifts for ^{1}H-NMR spectra are recorded in parts per million from tetramethylsilane with the residual protic solvent resonance as the internal standard ($CDCl_3$: δ 7.26 ppm, CD_3OD: δ 3.31 ppm, $(CD_3)_2SO$: δ 2.50 ppm, C_6D_6: δ 7.16 ppm, D_2O: δ 4.79 ppm, CD_3CN: δ 1.94 ppm). Data are reported as follows: chemical shift (multiplicity [s = singlet, bs = broad singlet, d = doublet, dd = doublet of doublets, t = triplet, q = quartet,

quin = quintet, oct = octet and m = multiplet], coupling constant (in Hz), integration and assignment). All multiplet signals were quoted over a chemical shift range.

^{13}C-NMR spectra are recorded with complete proton decoupling. Chemical shifts are reported in parts per million from tetramethylsilane with the solvent resonance as the internal standard ($CDCl_3$: *δ* 77.00 ppm, CD_3OD: *δ* 49.00 ppm, $(CD_3)_2SO$: *δ* 39.52 ppm, C_6D_6: *δ* 128.06 ppm, CD_3CN: *δ* 1.32 ppm, 118.26 ppm). The multiplicities are corresponding to the non-decoupled spectra and are described as follows: p = primary, s = secondary, t = tertiary, q = quaternary.

Assignments of ^{1}H- and ^{13}C-spectra were based upon the analysis of *δ*- and *J*-values, as well as COSY, HMBC, HSQC and adequate experiments where appropriate.

Mass spectrometry (MS): High resolution mass spectrometry (HRMS) was measured with a Micromass LCT with lockspray source. The injection proceeded in loop-mode with a HPLC system by WATERS (Alliance 2695). Alternatively, mass spectra were recorded with a Acquity-UPLC system by WATERS in combination with a QTOF Premier mass spectrometer by WATERS in lockspray mode. The ionization happened by electrospray ionization (ESI) or by chemical ionization at atmospheric pressure (APCI). The calculated and found mass are reported.

High performance liquid chromatography (HPLC): Semi-preparative HPLC was performed using an Alliance 2695 HPLC-system by WATERS with a WATERS 996 diode array detector (λ = 200-350 nm) and a Nucleodur C18 HTec column (5 *μ*m, 250 mm, Ø 8 mm) by MACHEREY-NAGEL. Mass detection was conducted with a WATERS Quattro micro API mass spectrometer in negative ionization mode.

Preparative HPLC was performed using a GILSON HPLC-system (pump 331/332) with additional MERCK HITACHI Split-Pump (L-6200A, UV-Vis detector L-4250) and a MACHEREY-NAGEL Nucleodur C18 ISIS column (5 *μ*m, 250 mm, Ø 21 mm with guard cartridge, 40 mm, Ø 21 mm). Mass detection was conducted with a WATERS Micromass ZQ mass spectrometer in negative ionisation mode.

Operating conditions and retention times (t_R) are reported in the experimental details.

Melting points: Melting points were determined on a SRS OptiMelt apparatus and are not corrected.

Optical Rotation: Specific optical rotation values $[\alpha]^t_D$ were measured in a quartz cuvette on a polarimeter 341 by PERKINELMER at a wavelength of 589 nm (D) and given temperature *t*. Concentrations *c* are given in g/100 mL solvent.

Freeze-pump-thaw-technique (fpt): Degassed solvents were prepared by the fpt technique. For this, the appropriate dry solvent was placed under an argon atmosphere in a SCHLENK flask being connected to the SCHLENK line. The solvent was frozen in the flask using liquid nitrogen. Then the stopcock was opened to vacuum and the atmosphere was evaporated for 5 minutes. The flask was sealed and thawed until the solvent melted using an acetone bath being replaced by the cooling bath in order to repeat these steps until a gas evolution at the solution was no longer seen. A minimum of three cycles was needed. Subsequently, the flask was filled with argon gas and sealed. The solvent was ready to use.

Peptide synthesizer: Peptides were synthesized with a LIBERTY BLUE™ Automated Microwave Peptide Synthesizer from CEM following a standard Fmoc-protocol (Table S1). A 2-chlorotrityl chloride resin (200-400 mesh, 1.50-1.90 mmol/g) from BACHEM was used. The appropriate reagents were prepared as stock solutions before they were added to the synthesizer. Standard couplings of Fmoc-protected amino acids (5.00 equiv. in regard to resin) were performed with DIC (5.00 equiv.) and OxymaPure (5.00 equiv.) in DMF. The corresponding Fmoc-deprotection was conducted with 10% piperazine (w/v) in EtOH:NMP (10:90, 5.00 equiv.). As required, the reaction mixture was irradiated with microwaves. The amino acids that were used are depicted in Table S5-2. The specific cleavages of the final peptides are described in the respective experimental.

Table S5-1. Overview of the settings used for the coupling as well as the deprotection.

	**Temp. [°C]**	**Power [W]**	**Time [s]**	**ΔT [°C]**
**standard coupling**	75	170	15	2
	90	30	110	1
**deprotection**	75	155	15	2
	90	30	50	1

Table S5-2: Natural amino acids used for solid phase synthesis of peptides, orthogonally protected natural amino acids and unnatural (amino) acids.

(amino)acid	one-letter code	three-letter code	reagent
**Alanine**	A	Ala	Fmoc-Ala-OH
**Arginine**	R	Arg	Fmoc-Arg(Pbf)-OH
**Valine**	V	Val	Fmoc-Val-OH
***N*-Me-Alanine**	-	-	Fmoc-*N*-Me-Ala-OH
***D*-Val**	v	D-Val	Fmoc-*D*-Val-OH
**Ornithine**	-	-	Fmoc-Ornithin(Boc)-OH
**(*R*)-3-(4-(Allyloxy)phenyl)-3-aminopropanoic acid**	-	-	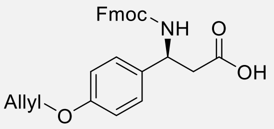
**Amino-3-methyl-3-nitrobutanoic acid**	-	-	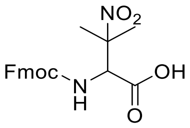
**Isovaleric acid**	-	-	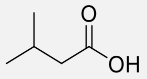

5.2. Experimental Procedures

β-Phenylalanine

(*R*)-2-((*tert*-Butoxycarbonyl)amino)-2-(4-hydroxyphenyl) acetic acid (S1)

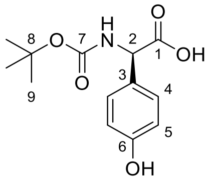

S1

4-Hydroxy-D-phenylglycine (20.4 g, 122 mmol, 1.00 equiv.) was suspended in 1,4-dioxane/H_2O (1:1, 800 mL). $NaHCO_3$ (51.1 g, 609 mmol, 5.00 equiv.) was added before addition of Boc_2O (29.2 g, 134 mmol, 1.10 equiv.) at 0 °C. The suspension was warmed to rt and stirred for 20 h. The organic solvent was removed under reduced pressure. EtOAc and a 5M HCl solution were added. The organic phase was washed with a 5M HCl solution, H_2O and brine, dried over $MgSO_4$ and concentrated under reduced pressure to furnish carbamate **S1** (35.4 g, quant.) as colorless solid, which was used in the next step without further purification.

$[\alpha]_D^{21}$: - 107.7° (*c* 0.10, MeOH); **^{1}H-NMR** (400 MHz, DMSO-d_6) = δ [ppm] 9.49 (bs, 1H, O*H*), 7.38 (d, *J* = 8.1 Hz, 1H, N*H*), 7.17 (d, *J* = 8.5 Hz, 2H, Ar-*H*), 6.71 (d, *J* = 8.4 Hz, 2H, Ar-*H*), 4.95 (d, *J* = 8.1 Hz, 1H, *H*-2), 1.37 (s, 9H, *H*-9).

The analytical data are consistent with those reported in the literature (G. M. Salituro, C. A. Townsend, *J. Am. Chem. Soc*. **1990**, *112*, 760-770).

***tert*-Butyl (*R*)-(2-hydroxy-1-(4-hydroxyphenyl)ethyl)carbamate (S2)**

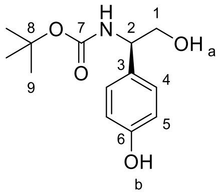

S2

Acid **S1** (33.4 g, 124 mmol, 1.00 equiv.) was dissolved in THF (165 mL) and BH_3 (0.9M in THF, 276 mL, 249 mmol, 2.00 equiv.) was added dropwise at 0 °C. The mixture was stirred at 0 °C for 2 h. The mixture was treated with H_2O and EtOAc and stirred at rt for 30 min. The aqueous phase was extracted with EtOAc. The combined organic phases were washed with H_2O and brine, dried over $MgSO_4$, filtered and concentrated under reduced pressure to furnish diol **S2** (25.2 g, 99.4 mmol, 80% yield) as colorless solid.

R$_f$ = 0.24 (5% MeOH in CH_2Cl_2); **[α]$_D^{22}$**: - 72.7° (*c* 0.10, MeOH); **^{1}H-NMR** (400 MHz, DMSO-d$_6$) = δ [ppm] 9.20 (s, 1H, O*H*$_b$), 7.06 (d, *J* = 8.5 Hz, 2H, Ar-*H*), 6.67 (d, *J* = 8.5 Hz, 2H, Ar-*H*), 4.68 (t, *J* = 5.8 Hz, 1H, O*H*$_a$), 4.41 (q, *J* = 7.2 Hz, 1H, *H*-2), 3.43-3.40 (m, 2H, *H*-1), 1.38 (s, 9H, *H*-9); **^{13}C-NMR** (100 MHz, DMSO-d$_6$) = δ [ppm] 156.1 (*C*-6), 155.1 (*C*-7), 132.1 (*C*-3), 127.8 (2C, Ar-*C*), 114.7 (2C, Ar-*C*), 77.5 (*C*-8), 65.0 (*C*-1), 56.2 (*C*-2), 28.3 (*C*-9); **HRMS** (ESI) *m/z* calculated for $C_{13}H_{19}NO_4Na$ $[M+Na]^+$: 276.1212; found: 276.1212.

***tert*-Butyl (*R*)-(1-(4-(allyloxy)phenyl)-2-hydroxyethyl)carbamate (S3)**

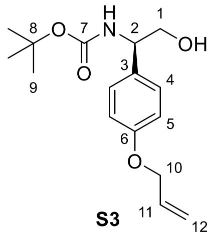

S3

Diol **S2** (405 mg, 1.60 mmol, 1.00 equiv.) was dissolved in MeOH (1.30 mL) and a NaOH solution (0.5M in H_2O, 3.20 mL, 1.60 mmol, 1.00 equiv.) was added. The solution was stirred at rt for 1 h. The solvent was removed under reduced pressure. The residue was dissolved in DMF (2.20 mL) before addition of allyl bromide (170 μL, 1.92 mmol, 1.20 equiv.) at 0 °C. The solution was stirred at rt for 3 h and subsequently concentrated under reduced pressure. The residue was purified by column chromatography (dry load, PE/EtOAc = 7:1, 5:1, 4:1, 3:1) to furnish primary alcohol **S3** (423 mg, 1.44 mmol, 90% yield) as colorless solid.

R$_f$ = 0.19 (PE/EtOAc = 2:1); **[α]$_D^{21}$**: - 63.0° (*c* 0.10, MeOH); **^{1}H-NMR** (400 MHz, DMSO-d$_6$) = *δ* [ppm] 7.19 (d, *J* = 8.6 Hz, 2H, Ar-*H*), 7.12 (d, *J* = 8.2 Hz, 1H, N*H*), 6.88 (d, *J* = 8.7 Hz, 2H, Ar-*H*), 6.07-5.98 (m, 1H, *H*-11), 5.38 (dq, *J* = 1.7, 17.3 Hz, 1H, *H*-12), 5.24 (dq, *J* = 1.6, 10.5 Hz, 1H, *H*-12), 4.72 (t, *J* = 5.7 Hz, 1H, O*H*), 4.53 (dt, *J* = 1.5, 5.2 Hz, 2H, *H*-10), 4.46 (q, *J* = 7.2 Hz, 1H, *H*-2), 3.46-3.42 (m, 2H, *H*-1), 1.36 (s, 9H, *H*-9); **^{13}C-NMR** (100 MHz, DMSO-d$_6$) = *δ* [ppm] 157.0 (*C*-6), 155.1 (*C*-7), 134.0 (*C*-11), 133.9 (*C*-3), 127.9 (Ar-*C*), 117.3 (*C*-12), 114.2 (Ar-*C*), 77.6 (*C*-8), 68.1 (*C*-10), 64.9 (*C*-1), 56.1 (*C*-2), 28.3 (*C*-9); **HRMS** (ESI) *m/z* calculated for $C_{13}H_{19}NO_4Na$ $[M+Na]^+$: 316.1525; found: 316.1523.

(*R*)-2-(4-(Allyloxy)phenyl)-2-((*tert*-butoxycarbonyl)amino)ethyl methanesulfonate (S4)

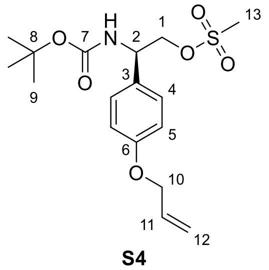

S4

Alcohol **S3** (200 mg, 0.68 mmol, 1.00 equiv.) was dissolved in CH_2Cl_2 (5.00 mL) and Et_3N (143 μL, 1.02 mmol, 1.50 equiv.) was added. The solution was cooled to 0 °C and MsCl (80.0 μL, 1.02 mmol, 1.50 equiv.) was added. The solution was stirred at 0 °C for 1 h before addition of a sat. NH_4Cl solution. The aqueous phase was extracted with CH_2Cl_2 (3x). The combined organic phases were washed with brine, dried over $MgSO_4$, filtered and concentrated under reduced pressure to furnish crude product **S4** (283 mg, quant.) as yellow solid, which was used in the next step without further purification.

$\mathbf{R_f}$ = 0.42 (PE/EtOAc = 2:1); **^{1}H-NMR** (400 MHz, DMSO-d_6) = δ [ppm] 7.59 (d, *J* = 8.9 Hz, 1H, N*H*), 7.28 (d, *J* = 8.4 Hz, 2H, Ar-*H*), 6.93 (d, *J* = 8.5 Hz, 2H, Ar-*H*), 6.08-5.98 (m, 1H, *H*-11), 5.39 (dd, *J* = 1.3, 17.2 Hz, 1H, *H*-12), 5.25 (dd, *J* = 1.2, 10.4 Hz, 1H, *H*-12), 4.82 (q, *J* = 5.6 Hz, 1H, *H*-2), 4.55 (d, *J* = 5.1 Hz, 2H, *H*-10), 4.22-4.19 (m, 2H, *H*-1), 3.15 (s, 3H, *H*-13), 1.37 (s, 9H, *H*-9).

***tert*-Butyl (*S*)-(1-(4-(allyloxy)phenyl)-2-cyanoethyl)carbamate (S5)**

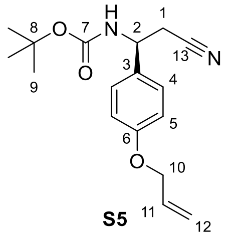

S5

Mesylate **S4** (0.68 mmol) was dissolved in DMSO (5.00 mL) and NaCN (100 mg, 2.05 mmol, 3.00 equiv.) was added. The solution was stirred at 40°C for 4 h before addition of H_2O. The aqueous phase was extracted with Et_2O (4x). The combined organic phases were washed with brine, dried over $MgSO_4$, filtered and concentrated under reduced

pressure. The residue was purified by column chromatography (PE/EtOAc = 4:1, 2:1) to furnish nitrile **S5** (120.8 mg, 0.40 mmol, 59% yield) as colorless solid.

R_f = 0.65 (PE/EtOAc = 2:1); **$[\alpha]_D^{22}$**: - 50.4° (*c* 0.12, MeOH); **^{1}H-NMR** (400 MHz, DMSO-d_6) = δ [ppm] 7.66 (d, *J* = 8.8 Hz, 1H, N*H*), 7.26 (d, *J* = 8.5 Hz, 2H, Ar-*H*), 6.92 (d, *J* = 8.7 Hz, 2H, Ar-*H*), 6.06-5.98 (m, 1H, *H*-11), 5.38 (dd, *J* = 1.3, 17.3 Hz, 1H, *H*-12), 5.25 (d, *J* = 10.4 Hz, 1H, *H*-12), 4.82-4.80 (m, 1H, *H*-2), 4.55 (d, *J* = 5.1 Hz, 2H, *H*-10), 2.86-2.82 (m, 2H, *H*-1), 1.37 (s, 9H, *H*-9); **^{13}C-NMR** (100 MHz, DMSO-d_6) = δ [ppm] 157.5 (*C*-6), 154.7 (*C*-7), 133.7 (*C*-11), 133.2 (*C*-3), 127.5 (Ar-*C*), 118.6 (*C*-13), 117.4 (*C*-12), 114.5 (Ar-*C*), 78.3 (*C*-8), 68.1 (*C*-10), 50.3 (*C*-2), 28.2 (*C*-9), 24.8 (*C*-1); **HRMS** (ESI) *m/z* calculated for $C_{17}H_{22}N_2O_3Na$ $[M+Na]^+$: 325.1528; found: 325.1528.

(*S*)-3-(4-(Allyloxy)phenyl)-3-((*tert*-butoxycarbonyl)amino)propanoic acid (S6)

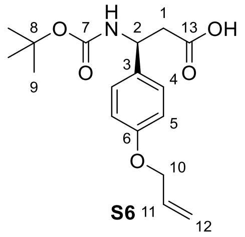

S6

Nitrile **S5** (8.71 g, 28.8 mmol) was dissolved in EtOH (350 mL) and NaOH (2M in H_2O, 144 mL, 288 mmol, 10.0 equiv.) was added. The solution was stirred at 90 °C for 3 h. After cooling down to rt the solvent was removed under reduced pressure. The residue was acidified with a 2M HCl solution and extracted with EtOAc (4x). The combined organic phases were washed with brine, dried over $MgSO_4$, filtered, concentrated under reduced pressure and to furnish carboxylic acid **S6** (9.08 g, 28.2 mmol, 98% yield) as colorless solid.

$[\alpha]_D^{24}$: - 52.6° (*c* 1.38, MeOH); **^{1}H-NMR** (400 MHz, DMSO-d_6): *δ* [ppm] 12.10 (bs, 1H, CO_2*H*), 7.35 (d, *J* = 8.7 Hz, 1H, N*H*), 7.20 (d, *J* = 8.6 Hz, 2H, Ar-*H*), 6.87 (d, *J* = 8.7 Hz, 2H, Ar-*H*), 6.07-5.98 (m, 1H, *H*-11), 5.38 (dq, *J* = 1.7, 17.3 Hz, 1H, *H*-12), 5.24 (dq, *J* = 1.6, 10.5 Hz, 1H, *H*-12), 4.83 (q, *J* = 7.3 Hz, 1H, *H*-2), 4.54-4.52 (m, 2H, *H*-10), 2.67-2.55 (m, 2H, *H*-1), 1.34 (s, 9H, *H*-9); **^{13}C-NMR** (100 MHz, DMSO-d_6) = δ [ppm] 171.9 (*C*-13), 157.1 (*C*-6), 154.7 (*C*-3), 135.4 (*C*-11), 133.8 (*C*-3), 127.5 (Ar-*C*), 117.4 (*C*-12), 114.3 (Ar-*C*),

77.8 (*C*-8), 68.1 (*C*-10), 50.5 (*C*-2), 41.4 (*C*-1), 28.3 (*C*-9); **HRMS** (ESI) *m/z* calculated for $C_{17}H_{22}NO_5$ [M-H]$^-$: 320.1498; found: 320.1497.

(*S*)-3-(4-(Allyloxy)phenyl)-3-aminopropanoic acid (S7)

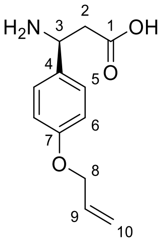

S7

To a solution of carbamate **S6** (2.30 g, 7.15 mmol, 1.00 equiv.) in CH_2Cl_2 (55.0 mL) at 0 °C, TFA (27.4 mL, 358 mmol, 50.0 equiv.) was added and the reaction mixture was stirred at 0 °C for 15 h. Then the solvent was removed under reduced pressure, affording deprotected amino acid **S7** as a colorless foam, which was used in the next step without further purification.

(*S*)-3-((((9*H*-fluoren-9-yl)methoxy)carbonyl)amino)-3-(4-(allyloxy)phenyl)propanoic acid (S8)

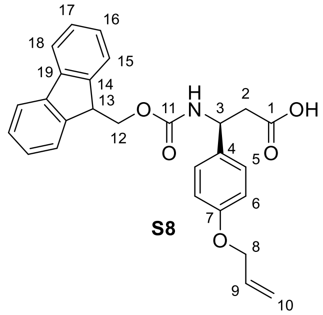

S8

To a solution of amino acid **S7** (1.58 g, 7.15 mmol, 1.00 equiv.) in 1,4-dioxane (72 mL) and a 10% Na_2CO_3 solution (143 mL) at 0 °C FmocCl (2.22 g, 8.57 mmol, 1.2 equiv.) in 1,4-dioxane (72.0 mL) was added over a period of 10 min. The reaction mixture was stirred at rt for 16 h. Then the reaction was diluted with H_2O (20.0 mL) and the aqueous phase was washed with Et_2O (3x 40.0 mL) and the pH was adjusted to 1 with conc. HCl. Then the aqueous phase was extracted with EtOAc (3x 20.0 mL). The combined organic extracts

were dried over $MgSO_4$ and filtered. The solvent was removed under reduced pressure. Purification by washing the crude product with H_2O and petroleum ether afforded acid **S8** (3.08 g, 6.94 mmol, 97% yield) as a colorless foam.

[*α*]$_D^{27}$: - 39.2° (*c* 0.38, MeOH); **^{1}H-NMR** (400 MHz, DMSO-d$_6$) = δ [ppm] 12.57-11.85 (bs, 1H, O*H*), 7.89-7.85 (m, 3H, NH, Ar-*H*), 7.67 (d, *J* = 7.4 Hz, 2H, Ar-*H*), 7.43-7.21 (m, 6H, Ar-*H*), 6.89 (d, *J* = 8.6 Hz, 2H, Ar-*H*), 6.08-5.98 (m, 1H, *H*-9), 5.41-5.36 (m, 1H, *H*-10), 5.26-5.23 (m, 1H, *H*-10), 4.92-4.86 (m, 1H, *H*-3), 4.54 (d, *J* = 5.2 Hz, 2H, *H*-12), 4.30-4.17 (m, 3H, *H*-8, *H*-13), 2.74-2.58 (m, 2H, *H*-2); **^{13}C-NMR** (100 MHz, DMSO-d$_6$) = δ [ppm] 171.8 (q, *C*-1), 157.2 (q, *C*-7), 155.3 (q, *C*-11), 143.9 (q, Ar-*C*), 143.8 (q, Ar-*C*), 140.7 (q, Ar-*C*), 135.0 (q, Ar-*C*), 133.8 (t, *C*-9), 127.6 (t, Ar-*C*), 127.5 (t, Ar-*C*), 127.1 (t, Ar-*C*), 127.0 (t, Ar-*C*), 125.2 (t, Ar-*C*), 125.1 (t, Ar-*C*), 120.1 (t, Ar-*C*), 117.4 (s, *C*-10), 114.4 (t, Ar-*C*), 68.1 (s, *C*-8), 65.3 (s, *C*-12), 51.0 (t, *C*-3), 46.7 (t, *C*-13), 41.1 (s, *C*-2); **HRMS** (ESI) *m/z* calculated for $C_{27}H_{25}NO_5Na$ $[M+Na]^+$: 466.1630; found: 466.1630.

Fragment A

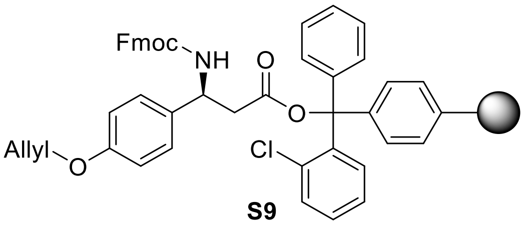

S9

Activation of 2-chlorotrityl chloride resin

The 2-chlorotrityl chloride resin (1.5 mmol/g, 2.10 g, 3.15 mmol, 1.00 equiv.) was suspended in CH_2Cl_2 (21.0 mL) at 0 °C. Then pyridine (609 *µ*L, 7.56 mmol, 2.40 equiv.) and thionyl chloride (274 *µ*L, 3.78 mmol, 1.20 equiv.) were added and the mixture was stirred at reflux. After 3 h the resin was filtered, washed with CH_2Cl_2 (6x) and dried *in vacuo*.

Loading of chlorotrityl resin

A mixture of acid **S8** (1.68 g, 3.78 mmol, 1.20 equiv.) and DIPEA (2.74 mL, 15.8 mmol, 5.00 equiv.) in CH_2Cl_2 (21.0 mL) was added to the activated chlorotrityl resin. The

suspension was stirred for 18 h at ambient temperature. Then, the resin was filtered, washed with CH_2Cl_2/MeOH/DIPEA, CH_2Cl_2, DMF, and CH_2Cl_2 and dried *in vacuo* to give resin **S9** in 3.10 g.

(3*S*,6*R*,9*R*,12*S*,15*S*)-3-(4-(Allyloxy)phenyl)-6,9-diisopropyl-12,13,15,22-tetramethyl-18- (2-nitropropan-2-yl)-5,8,11,14,17,20-hexaoxo-4,7,10,13,16,19-hexaazatricosanoic acid (fragment A)

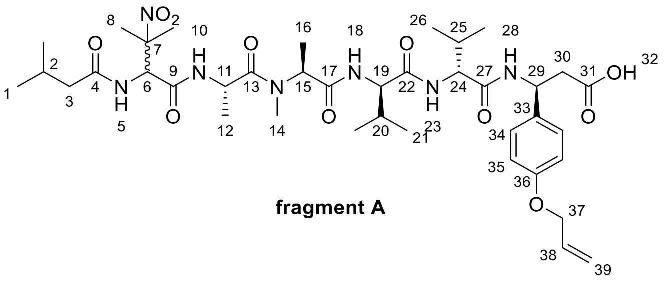

The title compound was prepared according to the general procedure for the synthesis of peptides with a Liberty Blue™ Automated Microwave Peptide Synthesizer from CEM in a 5x 0.50 mmol scale. Pre-loaded 2-chlorotrityl chloride resin **S9** (5x 333 mg), Fmoc-D-Val-OH (3.12 g), Fmoc-*N*-Me-Ala-OH (1.50 g), Fmoc-Ala-OH (1.43 g), amino-3-methyl-3-nitrobutanoic acid (1.77 g) and isovaleric acid (470 mg) were used. After peptide synthesis was completed, the resin was transferred into a 20.0 mL syringe with filter. The solvent was exhausted, and the resin was washed with DMF (3x 15.0 mL) and CH_2Cl_2 (3x 15.0 mL). The cleavage of the peptide was performed by adding 3.00 mL of 1% TFA in CH_2Cl_2 to the syringe vessel which was shaken for 2 min at rt. The liquid was filtered into a flask containing 3.00 mL of 10% pyridine in MeOH. The cleavage and filtration steps were repeated four more times. All the filtrates were combined in a 500 mL round bottom flask and the solvent was removed under reduced pressure. The sequence was checked via LCMS. Purification by flash column chromatography (RP- BÜCHI, solvent A: water + 0.1% FA, solvent B: MeOH + 0.1% FA, 15x150 mm column, flow rate: 80.0 mL/min, 15 sec/fr., gradient: (*t* [min]/solvent B [%]): 0/10; 3/10; 13/100; 15/100; t_R = 10.0 min) afforded fragment A (272 mg, 338 μmol, 68% yield) as a colorless foam.

^{1}H-NMR (DMSO-d$_6$, 400 MHz): δ [ppm] 8.58 – 8.50 (m, 1H, *H*-10), 8.38 – 8.36 (m, 1H, *H*-28), 8.21 – 8.15 (m, 1H, *H*-5), 7.81 – 7.78 (m, 1H, *H*-18/*H*-23), 7.59 – 7.29 (m, 1H, *H*-18/*H*-23), 7.20 (d, *J* = 8.6 Hz, 2H, 2x *H*-34), 6.84 (d, *J* = 8.6 Hz, 2H, 2x *H*-35), 6.06 – 5.97 (m,

1H, *H*-38), 5.39 – 5.34 (m, 1H, *syn H*-39), 5.25 – 5.22 (m, 1H, *anti H*-39), 5.14 (q, *J* = 7.6 Hz, 1H, *H*-29), 5.07 – 5.01 (m, 1H, *H*-15), 4.73 – 4.56 (m, 1H, *H*CCH_3), 4.53 – 4.51 (m, 2H, *H*-37), 4.19 – 4.14 (m, 1H, *H*-19/*H*-24), 4.09 (t, *J* = 8.3 Hz, 1H, (m, 1H, *H*-19/*H*-24), 3.17 – 2.81 (2s, 3H, *H*-14), 2.64 – 2.62 (m, 2H, *H*-30), 2.11 – 1.99 (m, 2H, *H*-3), 1.95 – 1.87 (m, 3H, *H*-2, *H*-20, *H*-25), 1.66 – 1.48 (m, 6H, *H*-8), 1.23 – 1.17 (m, 6H, *H*-12, *H*-16), 0.86 – 0.71 (m, 18H, *H*-1, *H*-21, *H*-26); **^{13}C-NMR** (DMSO-d$_6$, 100 MHz): δ [ppm] 172.0 (q, *C*-4), 171.9 (q, *C*=O), 171.7 (q, *C*=O), 170.9 (q, *C*=O), 170.8 (q, *C*=O), 170.6 (q, *C*=O), 169.7 (q, *C*=O), 157.1 (q, *C*-36), 134.3 (q, *C*-33), 133.8 (t, *C*-38), 127.6 (t, 2x *C*-34), 117.3 (s, *C*-39), 114.3 (t, 2x *C*-35), 89.0 (q, *C*-7), 68.1 (s, *C*-37), 57.9 (t, *C*-19/*C*-24), 57.3 (t, *C*-19/*C*-24), 56.8 (t, *C*-6), 51.5 (t, *C*-15), 48.6 (t, *C*-29), 45.6 (t, *C*-11), 44.2 (s, *C*-3), 40.7 (s, *C*-30), 30.4 (p, *C*-14), 30.2 (t, *C*-20/*C*-25), 25.7 (t, *C*-2), 25.6 (t, *C*-20/*C*-25), 23.6 – 22.3 (p, *C*-8), 23.3 (p, *C*-8), 22.3 (p, *C*-8), 22.2 (p, *C*-1/*C*-21/*C*-26), 22.1 (p, *C*-1/*C*-21/*C*-26), 22.0 (p, *C*-1/*C*-21/*C*-26), 21.6 (p, *C*-8), 19.2 (*C*-1/*C*-21/*C*-26), 19.1 (p, *C*-1/*C*-21/*C*-26), 18.3 (p, *C*-1/*C*-21/*C*-26), 18.2 (p, *C*-1/*C*-21/*C*-26), 17.9 (p, *C*-1/*C*-21/*C*-26), 16.5 (p, *C*-12), 14.5 (p, *C*-16); **HRMS** (ESI) *m/z* calculated for $C_{39}H_{62}N_7O_{11}$ $[M+H]^+$ 804.4507; found 804.4502.

Fragment B-D,L

(D,L)-3-Nitrovaline (S10)

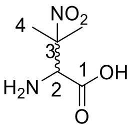

S10

To a solution of KOH (14.1 g, 251 mmol, 2.20 equiv.) in H_2O (280 mL), 2-nitropropane (20.0 mL, 223 mmol, 1.95 equiv.), NH_3 (25% in H_2O, 0.93 mol, 150 mL, 8.54 equiv.) and a solution of glyoxylic acid monohydrate (10.5 g, 114 mmol, 1.00 equiv.) in H_2O (40.0 mL) were added. The mixture was stirred for 2 h at ambient temperature. Then, the reaction was terminated by adjusting the pH to 0 with conc. HCl whereby a blue coloration of the solution appeared. The aqueous phase was washed with CH_2Cl_2 (3x) and the solvent was removed under reduced pressure. The residue was diluted in EtOH, filtered and the filtrate was concentrated to about half under reduced pressure, mixed in equal parts with Et_2O, filtered again and aniline (10-30 mL) was added to the filtrate until a turbidity of the solution appeared. The turbid solution was stored at 2-8 °C for 16 h. It was then filtered, the solid

was washed with ethanol and dried under high vacuum for 16 h. Nitrovaline **S10** (10.0 g, 61.8 mmol, 54% yield) was obtained as a colorless solid.

T_M: 142 °C; decomp. (Lit.: 143-144°C); **IR:** $\tilde{\nu}$ = 2975 (w), 2071 (w), 2021 (w), 1650 (m), 1592 (m), 1536 (s), 1490 (s), 1390 (s),1376 (m), 1357 (m), 1343 (s), 1204 (w), 1140 (w), 1090 (m), 906 (w), 853 (m), 779 (w), 734 (w), 678 (w) cm^{-1}; **^{1}H-NMR** (D_2O, 400 MHz): δ [ppm] 4.35 (s, 1H, *H*-2), 1.81 (s, 3H, *H*-4a), 1.77 (s, 3H, *H*-4b); **^{13}C-NMR** (D_2O, 100 MHz): δ [ppm] 170.2 (q, *C*-1), 88.3 (q, *C*-3), 60.8 (t, *C*-2), 24.6 (p, *C*-4a), 23.5 (p, *C*-4b).

The analytical data are consistent with those reported in the literature (P. A. Coghlan, C. J. Easton, *J. Chem. Soc., Perkin Trans. 1*, **1999**, 2659-2660).

***N*-Fmoc-D,L-3-Nitrovaline (1)**

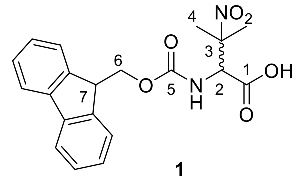

1

To a suspension of D,L-3-nitrovaline **S10** (6.28 g, 38.7 mmol, 1.00 equiv.) in 1,4-dioxane (175 mL) a Na_2CO_3 solution (10%, 350 mL) was added. Then, Fmoc-Cl (11.0 g, 42.6 mmol, 1.10 equiv.) dissolved in 1,4-dioxane (175 mL) was added via a dropping funnel at 0 °C over a period of 1 h. The reaction was stirred for 6 h at 0 °C. For terminating the reaction, water and Et_2O were added, the phases were separated, and the aqueous phase was washed with Et_2O (3x). Then, the pH of the aqueous phase was lowered to pH = 0-1 with semi-concentrated HCl and the aqueous phase was extracted with EtOAc (3x). The combined organic phases were washed with a sat. NaCl solution, dried over MgSO4, filtered, and the solvent was removed under reduced pressure. Product **1** was initially obtained as a yellowish resin that still contained residual solvent. Coevaporation with toluene (4x), MeOH (3x) and CH_2Cl_2 (2-4x) gave a colorless solid (10.7 g, 27.8 mmol, 72% yield).

T_M: 58-60 °C **IR:** $\tilde{\nu}$ =3426 (w), 3038 (w), 3003 (w), 2967 (w), 2882 (w), 1736 (s), 1690 (s), 1547 (s), 1518 (s), 1477 (w), 1449 (m), 1402 (w), 1377 (w), 1346 (m), 1310 (m), 1298 (m), 1223 (s), 1099 (w), 1055 (s), 1016 (w), 989 (w), 939 (w), 864 (w), 797 (w), 760 (s), 741 (s),

729 (s), 679 (w), 650 (w), 621 (w), 584 (w), 540 (m), 511 (m), 426 (m) cm^{-1}; **^{1}H-NMR** (DMSO-d_6, 400 MHz) *δ* [ppm] 8.11 (d, *J* = 9.9 Hz, 1H, N*H*), 7.89 (d, *J* = 7.5 Hz, 2H, Ar-*H*), 7.76-7.70 (m, 2H, Ar-*H*), 7.44-7.38 (m, 2H, Ar-*H*), 7.35-7.29 (m, 2H, Ar-*H*), 4.98 (d, *J* = 9.9 Hz, 1H, *H*-2), 4.40 (dd, *J* = 10.6, 7.2 Hz, 1H, *H*-6a), 4.33 (dd, *J* = 10.6, 7.2 Hz, 1H, *H*-6b), 4.25 (t, *J* = 7.0 Hz, 1H, *H*-7), 1.58 (s, 3H, *H*-4a), 1.50 (s, 3H, *H*-4b); **^{13}C-NMR** (DMSO-d_6, 100 MHz) *δ* [ppm] 170.0 (q, *C*-1), 156.7 (q, *C*-5), 143.8 (q, Fmoc, Ar-*C*), 143.7 (q, Fmoc, Ar-*C*), 140.8 (q, Fmoc, Ar-*C*), 140.8 (q, Fmoc, Ar-*C*), 127.8 (t, Fmoc, Ar-*C*), 127.8 (t, Fmoc, Ar-*C*), 127.2 (t, Fmoc, Ar-*C*), 127.2 (t, Fmoc, Ar-*C*), 125.4 (t, Fmoc, Ar-*C*), 125.3 (t, Fmoc, Ar-*C*), 120.2 (t, Fmoc, Ar-*C*), 120.2 (t, Fmoc, Ar-*C*), 88.5 (q, *C*-3), 66.2 (s, *C*-6), 59.3 (t, *C*-2), 46.7 (t, *C*-7), 24.7 (p, *C*-4b), 21.2 (p, *C*-4a); **HRMS** (ESI): *m/z* calculated for $C_{20}H_{19}N_2O_6$ [M - H^+]: 383.1243, found 383.1240.

Methyl ((*R*)-2-((((9*H*-fluoren-9-yl)methoxy)carbonyl)amino)-5-((*tert*-butoxycarbonyl)-amino)pentanoyl)-L-valinate (S11)

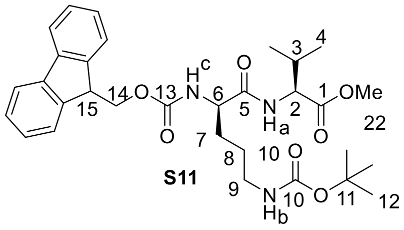

To a solution of Fmoc-D-Orn(Boc)-OH (**2**, 5.10 g, 11.2 mmol, 1.00 equiv.), L-Val-OMe•HCl (2.20 g, 13.5 mmol, 1.20 equiv.), EDC·HCl (2.36 g, 12.3 mmol, 1.10 equiv.) and HOAt (1.67 g, 12.3 mmol, 1.10 equiv.) in dry CH_2Cl_2 (100 mL) and dry DMF (20 mL), $NaHCO_3$ (4.71 g, 56.1 mmol, 5.00 equiv.) was added at 0 °C and the reaction mixture was stirred for 16 h at ambient temperature under an Argon atmosphere. The solvent was removed under reduced pressure. Purification by flash column chromatography (CH_2Cl_2/MeOH, 100:0 -> 99.5:0.5 -> 99:1 -> 98:2 -> 97:3) afforded peptide **S11** (6.26 g, 11.0 mmol, 98% yield) as a colorless foam.

$[\alpha]_D^{21.9}$: + 6.2°(*c* 1.75, CH_2Cl_2); **^{1}H-NMR** ($CDCl_3$, 400 MHz): *δ* [ppm] 7.75 (d, *J* = 7.6 Hz, 2H, Ar-*H*, Fmoc), 7.59 (d, *J* = 7.4 Hz, 2H, Ar-*H*, Fmoc), 7.38 (t, *J* = 7.4 Hz, 2H, Ar-*H*, Fmoc), 7.29 (t, *J* = 7.4 Hz, 2H, Ar-*H*, Fmoc), 6.88 (bs, 1H, NH_a), 5.77 (bs, 1H, NH_c), 4.78 (bs, 1H, NH_b), 4.51 (dd, *J* = 8.7, 5.2 Hz, 1H, *H*-2), 4.43-4.33 (m, 3H, *H*-6 u. *H*-14), 4.21 (t, *J* = 7.0 Hz, 1H, *H*-15), 3.68 (s, 3H, *H*-22), 3.26-3.04 (m, 2H, *H*-9), 2.13 (okt, *J* = 6.5 Hz,

1H, *H*-3), 1.97-1.82 (m, 1H, *H*-7a), 1.73-1.61 (m, 1H, *H*-7b), 1.61-1.48 (m, 2H, *H*-8), 1.43 (s, 9H, *H*-12), 0.92 (d, *J* = 6.7 Hz, 3H, *H*-4a), 0.88 (d, *J* = 6.7 Hz, 3H, *H*-4b); **^{13}C-NMR** ($CDCl_3$, 100 MHz): *δ* [ppm] 172.2 (q, *C*-1), 171.9 (q, *C*-5), 156.5 (q, *C*-10 u. *C*-13), 144.0 (q, Ar-*C*, Fmoc), 143.8 (q, Ar-*C*, Fmoc), 141.4 (q, Ar-*C*, Fmoc), 141.4 (q, Ar-*C*, Fmoc), 127.8 (2C, t, Ar-*C*, Fmoc), 127.2 (2C, t, Ar-*C*, Fmoc), 125.2 (2C, t, Ar-*C*, Fmoc), 120.1 (t, Ar-*C*, Fmoc), 120.1 (t, Ar-*C*, Fmoc), 79.4 (q, *C*-11), 67.2 (s, *C*-14), 57.4 (t, *C*-2), 54.4 (t, *C*-6), 52.3 (p, *C*-22), 47.2 (t, *C*-15), 40.0 (s, *C*-9), 31.3 (t, *C*-3), 30.3 (s, *C*-7), 28.5 (3C, p, *C*-12), 26.4 (s, *C*-8), 19.1 (p, *C*-4a), 18.0 (p, *C*-4b); **HRMS** (ESI): *m/z* calculated for $C_{31}H_{41}N_3O_7Na$ [M + Na^+]: 590.2842, found 590.2842.

Methyl ((2R)-2-(2-((((9*H*-fluoren-9-yl)methoxy)carbonyl)amino)-3-methyl-3-nitrobutan-amido)-5-((tert-butoxycarbonyl)amino)pentanoyl)-L-valinate (3)

Fmoc-*Deprotection:*

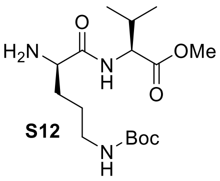

S12

To a solution of carbamate **S11** (2.19 g, 3.86 mmol, 1.00 equiv.) in dry CH_2Cl_2 (40.0 mL) tris(2-aminoethyl)-amine (4.00 mL) was added at 0 °C and the reaction mixture was stirred at ambient temperature. After 4 h (TLC control: CH_2Cl_2/MeOH, 9:1) silica gel was added and the solvent was removed under reduced pressure. Purification by flash column chromatography (CH_2Cl_2/ MeOH/ Et_3N, 98:1:1) afforded amine **S12** (1.32 g, 3.82 mmol, 99% yield) as a yellow oil which still contained traces of Et_3N. It was coevaporated with CH_2Cl_2 (3x) and used in the next step without further purification.

Peptide coupling:

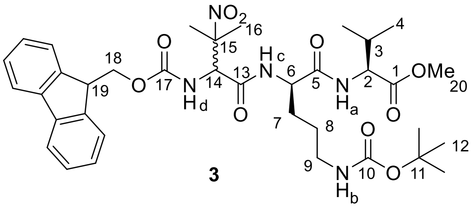

3

To a solution of (*R,S*)-Fmoc-3-nitrovaline (**1**, 1.91 g, 4.97 mmol, 1.30 equiv.), EDC·HCl (0.95 g, 4.97 mmol, 1.30 equiv.) and HOAt (0.68 g, 4.97 mmol, 1.30 equiv.) in dry CH_2Cl_2 (40.0 mL) and dry DMF (8.00 mL), $NaHCO_3$ (1.60 g, 19.1 mmol, 5.00 equiv.) was added at 0 °C. Amine **S12** was dissolved in dry CH_2Cl_2 (5.00 mL) and added to the reaction mixture which was stirred for 18 h at ambient temperature. The reaction was filtered and the solvent was removed under reduced pressure. The residue was coevaporated with toluene (3x). Purification by flash column chromatography (CH_2Cl_2/MeOH, 100:0 -> 99.5:0.5 -> 99:1 -> 98:2 -> 97:3 -> 96:4) afforded peptide **3** (2.34 g, 3.29 mmol, 86% yield, *d.r.* = 1.5:1) as a yellow foam.

The compound was obtained as a mixture of diastereoisomers (1.5:1). The individual diastereoisomers were not separated and the exact configuration at C 14 remains unknown. In the following, the signals of the major diastereoisomer and the minor diastereoisomer Are *given separately. Only the mixture is shown in the spectra appendix.*

Major: **^{1}H-NMR** ($CDCl_3$, 400 MHz): δ [ppm] 7.78-7.62 (m, 3H, NH_c, Ar-*H*, Fmoc), 7.59-7.53 (m, 2H, Ar-*H*, Fmoc), 7.41-7.33 (m, 2H, Ar-*H*, Fmoc), 7.32-7.24 (m, 2H, Ar-*H*, Fmoc), 7.02-6.90 (m, 1H, NH_a), 6.43-6.33 (m, 1H, NH_d), 5.01 (d, *J* = 8.2 Hz, 1H, *H*-14), 4.91-4.76 (m, 1H, NH_b), 4.70-4.56 (m, 1H, *H*-6), 4.56-4.43 (m, 2H, *H*-2, *H*-18a), 4.37-4.25 (m, 1H, *H*-18b), 4.23-4.13 (m, 1H, *H*-19), 3.66 (s, 3H, *H*-20), 3.22-3.01 (m, 2H, *H*-9), 2.19-2.04 (m, 1H, *H*-3), 1.92-1.80 (m, 1H, *H*-7a), 1.75-1.62 (m, 4H, *H*-16a, *H*-7b), 1.59 (s, 3H, *H*-16b), 1.54-1.45 (m, 2H, *H*-8), 1.40 (s, 9H, *H*-12), 0.91 (d, *J* = 6.5 Hz, *H*-4a), 0.87 (d, *J* = 7.5 Hz, *H*-4b); **^{13}C-NMR** ($CDCl_3$, 100 MHz): δ [ppm] 172.0 (q, *C*-1), 171.2 (q, *C*-5), 168.0 (q, *C*-13), 156.7 (q, *C*-10 od. *C*-17), 156.5 (q, *C*-10 od. *C*-17), 143.7 (q, Ar-*C*, Fmoc), 143.7 (q, Ar-*C*, Fmoc), 141.4 (2C, q, Ar-*C*, Fmoc), 127.9 (2C, t, Ar-*C*, Fmoc), 127.2 (2C, t, Ar-*C*, Fmoc), 125.3 (t, Ar-*C*, Fmoc), 125.1 (t, Ar-*C*, Fmoc), 120.1 (t, Ar-*C*, Fmoc), 120.1 (t, Ar-*C*, Fmoc), 89.0 (q, *C*-15), 79.5 (q, *C*-11), 67.6 (s, *C*-18), 60.0 (t, *C*-14), 57.5 (t, *C*-2), 53.0 (*C*-6), 52.2 (p, *C*-20), 47.1 (t, *C*-19), 39.8 (s, *C*-9), 31.2 (t, *C*-3), 29.2 (s, *C*-7), 28.5 (3C, p, *C*-12), 26.5 (s, *C*-8), 24.4 (p, *C*-16a), 23.3 (p, *C*-16b), 19.1 (p, *C*-4a), 18.1 (p, *C*-4b); *Minor:* **^{1}H-NMR** ($CDCl_3$, 400 MHz): δ [ppm] 7.78-7.69 (m, 2H, Ar-*H*, Fmoc), 7.59-7.53 (m, 2H, Ar-*H*, Fmoc), 7.41-7.33 (m, 3H, NH_c, Ar-*H*, Fmoc), 7.32-7.24 (m, 2H, Ar-*H*, Fmoc), 7.02-6.90 (m, 1H, NH_a), 6.33-6.22 (m, 1H, NH_d), 4.96 (d, *J* = 9.6 Hz, 1H, *H*-14), 4.91-4.76 (m, 1H, NH_b), 4.70-4.56 (m, 1H, *H*-6), 4.56-4.43 (m, 2H, *H*-2, *H*-18a), 4.37-4.25 (m, 1H, *H*-18b), 4.23-4.13 (m, 1H, *H*-19), 3.62 (s, 3H, *H*-20), 3.22-3.01 (m, 2H, *H*-9), 2.19-2.04 (m, 1H, *H*-3), 1.92-1.80 (m, 1H, *H*-7a), 1.75-1.62 (m, 4H, *H*-16a, *H*-7b), 1.57 (s, 3H, *H*-16b), 1.54-1.45 (m, 2H, *H*-8), 1.42 (s, 9H, *H*-12), 0.89 (d, *J* = 7.2 Hz, *H*-4a), 0.86 (d, *J* = 7.2 Hz, *H*-4b); **^{13}C-**

NMR ($CDCl_3$, 100 MHz): *δ* [ppm] 172.2 (q, *C*-1), 171.2 (q, *C*-5), 168.0 (q, *C*-13), 156.6 (q, *C*-10 od. *C*-17), 156.5 (q, *C*-10 od. *C*-17), 143.8 (q, Ar-*C*, Fmoc), 143.5 (q, Ar-*C*, Fmoc), 141.4 (2C, q, Ar-*C*, Fmoc), 127.9 (2C, t, Ar-*C*, Fmoc), 127.3 (2C, t, Ar-*C*, Fmoc), 125.2 (t, Ar-*C*, Fmoc), 125.1 (t, Ar-*C*, Fmoc), 120.1 (t, Ar-*C*, Fmoc), 120.1 (t, Ar-*C*, Fmoc), 89.2 (q, *C*-15), 79.5 (q, *C*-11), 67.5 (s, *C*-18), 59.9 (t, *C*-14), 57.5 (t, *C*-2), 52.9 (t, *C*-6), 52.3 (p, *C*-20), 47.2 (t, *C*-19), 39.8 (s, *C*-9), 31.2 (t, *C*-3), 29.4 (s, *C*-7), 28.5 (3C, p, *C*-12), 26.4 (s, *C*-8), 24.4 (p, *C*-16a), 23.2 (p, *C*-16b), 19.1 (p, *C*-4a), 18.0 (p, *C*-4b); **HRMS** (ESI): *m/z* calculated for $C_{36}H_{49}N_5O_{10}Na$ [M + Na^+]: 734.3377, found 734.3380.

Teoc-D-Val-OH (S13)

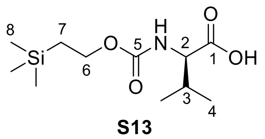

S13

To a solution of D-valine (1.22 g, 10.4 mmol, 1.00 equiv.) in H_2O (10 mL), TeocOSu (3.00 g, 11.5 mmol, 1.10 equiv.) in 1,4-dioxane (10.0 mL) and Et_3N (2.16 mL, 15.6 mmol, 1.50 equiv.) were added and the reaction was stirred for 24 h at ambient temperature. Then the reaction was terminated with a solution of 1M $KHSO_4$ and EtOAc. The aqueous layer was extracted with EtOAc (3x). The combined organic extracts were washed with H_2O (4x), dried with $MgSO_4$ and filtered. The solvent was removed under reduced pressure. Acid **S13** (2.72 g, 10.4 mmol, quant.) was obtained as a colorless oil and used in the next step without further purification.

$[\alpha]_D^{26.6}$: + 6.9° (*c* 2.52; MeOH); **^{1}H-NMR** ($CDCl_3$, 400 MHz): *δ* [ppm] 10.86 (bs, 1H, COO*H*), 5.15 (d, *J* = 8.8 Hz, 1H, N*H*), 4.32 (dd, *J* = 9.0, 4.2 Hz, 1H, *H*-2), 4.21-4.11 (m, 2H, *H*-6), 2.26-2.15 (m, 1H, *H*-3), 1.01-0.92 (m, 8H, *H*-4 u. *H*-7), 0.03 (s, 9H, *H*-8).

(*N*-Teoc-D-valyl)-(*α,β*-dehydrovalyl)-(*Nδ*-Boc-D-ornityl)-D-valinemethylester (S15)

Fmoc *deprotection:*

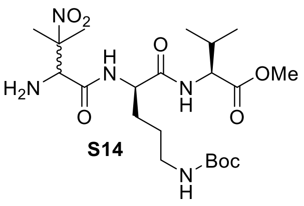

S14

To a solution of carbamate **3** (3.31 g, 4.65 mmol, 1.00 equiv.) in DMF (50.0 mL), dimethylamine (40% in H_2O, 5.90 mL, 46.5 mmol, 10.0 equiv.) was added at ambient temperature. After 6 h the solvent was removed under reduced pressure (at 40°C) and the residue was used in the next step without further purification.

Peptide coupling:

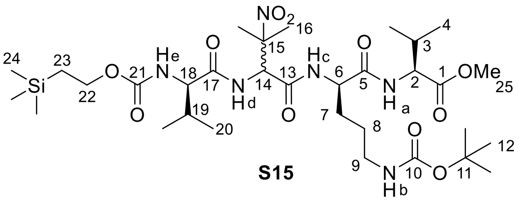

S15

To a solution of HOAt (700 mg, 5.81 mmol, 1.25 equiv.) and EDC·HCl (1.11 g, 5.81 mmol, 1.25 equiv.) in dry CH_2Cl_2 (10 mL), dissolved *N*-Teoc-D-valine (**S13**, 1.34 g, 5.12 mmol, 1.10 equiv.) was added and the reaction mixture was diluted with further 40.0 mL of dry CH_2Cl_2. Then, DIPEA (4.10 mL, 23.3 mmol, 5.00 equiv.) was added dropwise over a period of 10 min at 0 °C. Finally, amine **S14** was diluted in DMF (10.0 mL) and added over a period of 10 min to the reaction mixture that was stirred at ambient temperature for 18 h. The, the solvent was removed under reduced pressure and the residue was dissolved in EtOAc and a half saturated NaCl-solution. The organic layer was washed with half saturated NaCl solution (4 x). The combined organic extracts were dried with $MgSO_4$, filtered and the solvent was removed under reduced pressure. Purification by flash column chromatography (CH_2Cl_2/MeOH, 100:0 -> 99.5:0.5 -> 99:1 -> 98.5:1.5 -> 98:2) afforded peptide **S15** (2.70 g, 3.68 mmol, 79% yield, *d.r.* = 3:1) as a yellow foam.

The compound was obtained as a mixture of two diastereoisomers with a ratio of about 3:1. The mixture was not further separated. Therefore, the total sum of all signals is given below, with the signals of the major diastereoisomer labeled "major" and the minor diastereoisomer labeled "minor". **^{1}H-NMR** (DMSO-d$_6$, 400 MHz): δ [ppm] 8.40 (d, J = 9.8 Hz, 0.25H, NH_d, minor), 8.37 (d, J = 8.2 Hz, 0.25H, NH_c, minor), 8.23-8.11 (m, 2.5H,

NH_a u. NH_c, major u. NH_d, major), 7.15 (d, J = 8.8 Hz, 0.75H, NH_e, major), 6.98 (d, J = 8.2 Hz, 0.25H, NH$_e$, minor), 6.77-6.75 (m, 1H, NH_b), 5.28 (d, J = 9.7 Hz, 0.25 H, *H*-14, minor), 5.24 (d, J = 9.6 Hz, 0.75H, *H*-14, major), 4.40-4.32 (m, 1H, *H*-6), 4.21-4.15 (m, 1H, *H*-2), 4.10-3.95 (m, 2.25H, *H*-22 u. *H*-18, minor), 3.89 (t, J = 8.1 Hz, 0.75H, *H*-18, major), 3.62 (s, 3H, *H*-25), 2.92-2.81 (m, 2H, *H*-9), 2.08-1.96 (m, 1H, *H*-3), 1.96-1.84 (m, 1H, *H*-19), 1.68-1.43 (m, 8H, *H*-7 u. *H*-16a/b), 1.43-1.26 (m, 11H, *H*-8 u. *H*-12), 0.92 (t, J = 8.3 Hz, 2H, *H*-23), 0.87-0.77 (m, 12H, *H*-4a/b u. *H*-20a/b), 0.01 (s, 6.75H, *H*-25, major), 0.01 (s, 2.25H, *H*-25, minor); **^{13}C-NMR** (DMSO-d$_6$, 100 MHz): δ [ppm] 171.9 (q, *C*-17, minor), 171.8 (q, *C*-17, major), 171.8 (q, *C*-1, major), 171.8 (q, *C*-1, minor), 171.3 (q, *C*-5, major), 171.2 (q, *C*-5, minor), 167.0 (q, *C*-13, minor), 166.8 (q, *C*-13, major), 156.4 (q, *C*-21, minor), 156.2 (q, *C*-21, major), 155.5 (q, *C*-10), 88.8 (q, *C*-15, major), 88.7 (q, *C*-15, minor), 77.4 (q, *C*-11), 61.9 (s, *C*-22, minor), 61.8 (s, *C*-22, major), 60.2 (t, *C*-18, major), 59.8 (t, *C*-18, minor), 57.3 (t, *C*-14, minor), 57.2 (t, *C*-2, major), 57.1 (t, *C*-2, minor), 57.0 (t, *C*-14, major), 52.3 (t, *C*-6), 51.7 (p, *C*-25, major), 51.7 (p, *C*-25, minor), 30.3 (t, *C*-19, minor), 30.2 (t, *C*-3, minor), 30.1 (t, *C*-19, major), 30.1 (t, *C*-3, major), 29.7 (s, *C*-7, major), 29.6 (s, *C*-7, minor), 28.3 (3C, p, *C*-12), 25.9 (s, *C*-8), 23.4 (p, *C*-16a, minor), 23.4 (p, *C*-16a, major), 22.1 (p, *C*-16b, minor), 22.0 (p, *C*-16b, major), 19.2 (p, *C*-4a, major od. *C*-20a, major), 19.1 (p, *C*-4a, minor od. *C*-20a, minor), 19.0 (p, *C*-4a, major od. *C*-20a, major), 19.0 (p, *C*-4a, minor od. *C*-20a, minor), 18.2 (p, *C*-4b, major od. *C*-20b, major), 18.1 (p, *C*-4b, major od. *C*-20b, major), 18.1 (p, *C*-4b, minor), 17.8 (p, *C*-20b, minor), 17.3 (s, *C*-23, minor), 17.3 (s, *C*-23, major), -1.5 (3C, p, *C*-24, major), -1.5 (3C, p, *C*-24, minor);

Storage of the final compound together with DMF residues at 4 °C resulted in quantitative epimerization of the nitro-valine stereocenter and consequent conversion of the major diastereoisomer to the minor diastereoisomer. The signals of the major diastereomer (formerly minor diastereomer) after isomerization in DMF are given below.: **^{1}H-NMR** (DMSO-d$_6$, 400 MHz): δ [ppm] 8.41 (d, J = 9.6 Hz, 1H, NH_d), 8.36 (d, J = 8.0 Hz, 1H, NH_c), 8.11 (d, J = 8.5 Hz, 1H, NH_a), 6.96 (d, J = 7.8 Hz, 1H, NH_e), 6.75 (t, J = 4.9 Hz, 1H, NH_b), 5.28 (d, J = 9.6 Hz, 1H, *H*-14), 4.38 (dt, J = 8.4, 5.3 Hz, 1H, *H*-6), 4.20 (dd, J = 8.5, 6.6 Hz, 1H, *H*-2), 4.10-3.95 (m, 3H, *H*-18 u. *H*-22), 3.62 (s, 3H, *H*-25), 2.95-2.80 (m, 2H, *H*-9), 2.01 (okt, J = 6.5 Hz, 1H, *H*-3), 1.90 (okt, J = 6.3 Hz, 1H, *H*-19), 1.69-1.46 (m, 8H, *H*-7 u. *H*-16), 1.46-1.26 (m, 11H, *H*-8 u. *H*-12), 0.92 (t, J = 8.4 Hz, 2H, *H*-23), 0.83 (d, J = 6.8 Hz, 6H, *H*-4a/b), 0.81 (d, J = 6.7 Hz, 3H, *H*-20a), 0.78 (d, J = 6,8 Hz, 3H, *H*-20b), 0.00 (s, 9H, *H*-24); **^{13}C-NMR** (DMSO-d$_6$, 100 MHz): δ [ppm] 171.9 (q, *C*-17), 171.8 (q, *C*-1), 171.3 (q, *C*-5), 167.0 (q, *C*-13), 156.5 (q, *C*-21), 155.5 (q, *C*-10), 88.7 (q, *C*-15), 77.3 (q, *C*-11), 61.9 (s, *C*-22), 59.8 (t, *C*-18), 57.3 (t, *C*-14), 57.2 (t, *C*-2), 52.3 (t, *C*-6), 51.7 (p, *C*-25), 30.3 (t, *C*-

19), 30.2 (t, *C*-3), 29.6 (s, *C*-7), 28.3 (3C, p, *C*-12), 25.9 (s, *C*-8), 23.4 (p, *C*-16a), 22.2 (p, *C*-16b), 19.1 (p, *C*-20a), 19.0 (p, *C*-4a), 18.1 (p, *C*-4b), 17.8 (p, *C*-20b), 17.4 (s, *C*-23), -1.5 (3C, p, *C*-24) *{ C 9 is located below the DMSO signal and is therefore not visible in the 13C spectrum }*; **HRMS** (ESI): *m/z* calculated for $C_{32}H_{61}N_6O_{11}Si$ [M + H^+]: 733.4168, found 733.4176.

Methyl ((2R)-2-(2-((R)-2-amino-3-methylbutanamido)-3-methyl-3-nitrobutanamido)-5-((tert-butoxycarbonyl)-amino)pentanoyl)-L-valinate (fragment B-D,L)

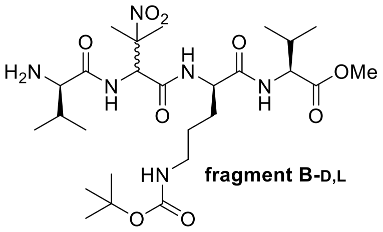

To a solution of Teoc-protected peptide **S15** (575 mg, 785 *µ*mol, 1.00 equiv.) in THF (15.7 mL), TBAF (1M in THF, 7.85 mL, 0.1M) was added and the reaction mixture was stirred for 15 h at ambient temperature. Then the solvent was removed under reduced pressure. Purification by flash column chromatography (RP- Büchi, solvent A: water + 0.1% FA, solvent B: MeCN + 0.1% FA; 12x150 mm column; flow rate: 10.0 ml/min; 60 sec/fr; gradient: (*t* [min]/solvent B [%]): 0/0; 5/0; 55/100; 60/100; t_R = 7.0 – 11.5 min) afforded fragment B-D,L (487.1 mg, 575 *µ*mol, quant.) as a colorless foam that was used in the next step without further purification.

Fragment AB-D,L

((2*R*)-2-(2-((2*R*)-2-((3*S*)-3-(4-(Allyloxy)phenyl)-3-((2*R*)-3-methyl-2-((2*R*)-3-methyl-2-((2*S*)-2-((2*S*)-*N*-methyl-2-(3-methyl-2-(3-methylbutanamido)-3-nitrobutanamido) propanamido)-propanamido)butanamido)butan-amido)propanamido)-3-methyl-butanamido)-3-methyl-3-nitrobutanamido)-5-((*tert*-butoxycarbonyl)amino) pentanoyl)-L-valine (S16)

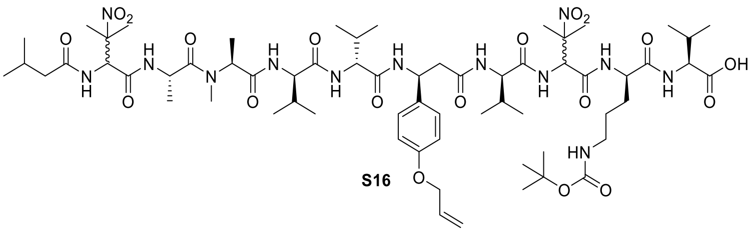

S16

To a solution of fragment A (78.7 mg, 97.9 μmol, 1.00 equiv.) and fragment B-D,L (125 mg, 212 μmol, 2.16 equiv.) in DMF (1.00 mL) at 0 °C, HOAt (29.3 mg, 215 μmol, 2.20 equiv.), PyAOP (153 mg, 294 μmol, 3.00 equiv.) and DIPEA (85.3 µL, 490 μmol, 5.00 equiv.) were added and the reaction mixture was stirred for 23 h at ambient temperature. The reaction was terminated with MeOH (1.00 mL) and the solvent was removed under reduced pressure. Purification by flash column chromatography (RP-BÜCHI; solvent A: water + 0.1% FA, solvent B: MeCN + 0.1% FA, 4 g WP C18 column, flow rate: 10.0 mL/min, 30 sec/fr., gradient: (t [min]/solvent B [%]): 0/0; 24/95; 30/95; t_R = 22.0 – 27.0 min) afforded peptide **S16** (95.2 mg, 69.0 μmol, 71% yield) as a colorless foam.

Due to the number of diastereomers caused by the two nitro groups, peptide **S16** was used in the next step without further purification or characterization.

((*R*)-2-(2-((*R*)-2-((*S*)-3-(4-(Allyloxy)phenyl)-3-((*R*)-3-methyl-2-((*R*)-3-methyl-2-((*S*)-2-((*S*)-*N*-methyl-2-(3-methyl-2-(3-methylbutanamido)but-2-enamido)pro-panamido) propanamido)butanamido)butanamido)propan-amido)-3-methylbutan-amido)-3-methyl-but-2-enamido)-5-((*tert*-butoxycarbonyl)amino)pentanoyl)-L-valine (fragment AB-D,L)

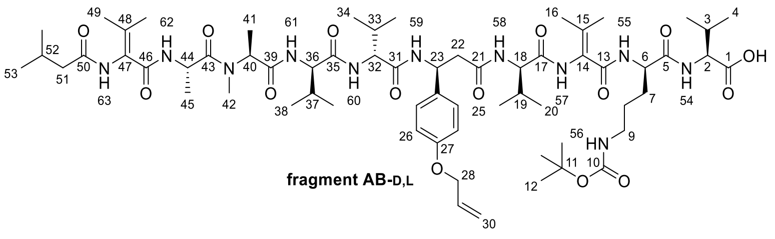

fragment AB-D,L

To a solution of peptide **S16** (27.3 mg, 20.0 μmol, 1.00 equiv.) in THF (500 μL), LiOH (1M in H_2O, 8.40 mg, 200 μmol, 10.0 equiv.) was added dropwise at 0 °C and the reaction mixture was stirred at ambient temperature. After 4 h, the reaction was terminated with phosphate buffer solution (pH 7) and adjusted to pH 7. The solvent was removed under reduced pressure. Purification by flash column chromatography (RP-BÜCHI; solvent A:

water + 0.1% FA, solvent B: MeCN + 0.1% FA, 4 g WP C18 column, flow rate: 10.0 mL/min, 30 sec/fr., gradient: (*t* [min]/solvent B [%]): 0/0; 25/95; 30/95; t_R = 14.0 – 20.5 min) afforded fragment AB-D,L (25.6 mg, 20.0 µmol, quant., *d.r.* = 9.7:0.3) as a colorless foam.

$[\alpha]_D^{21.4}$: - 7.7° (*c* 0.3, DMSO-d_6); **^{1}H-NMR** (DMSO-d_6, 600 MHz): *δ* [ppm] 9.13 (s, 1H, *H*-57/*H*-63), 8.91 (s, 1H, *H*-57/*H*-63), 8.32 – 8.27 (m, 1H, N*H*), 7.92 – 7.88 (m, 2H, N*H*), 7.80 – 7.78 (m, 1H, N*H*), 7.63 – 7.61 (m, 1H, N*H*), 7.54 – 7.49 (m, 2H, N*H*), 7.20 (d, *J* = 7.9 Hz, 2H, *H*-25), 6.80 (d, *J* = 9.2 Hz, 2H, *H*-26), 6.71 (t, *J* = 5.6 Hz, 1H, *H*-56), 6.03 – 5.97 (m, 1H, *H*-29), 5.36 – 5.33 (m, 1H, *H*-30), 5.23 – 5.21 (m, 1H, *H*-30), 5.15 – 5.12 (m, 1H, *H*-23), 5.05 – 4.98 (m, 1H, *H*-40), 4.71 – 4.67 (m, 1H, *H*-44), 4.51 – 4.50 (m, 2H, *H*-28), 4.33 – 4.29 (m, 1H, *H*-6), 4.16 – 4.03 (m, 4H, *H*-2, *H*-18, *H*-32, *H*-36), 2.89 – 2.84 (m, 2H, *H*-9), 2.85 (s, 3H, *H*-42), 2.77 – 2.74 (m, 1H, *H*-22), 2.62 – 2.61 (m, 1H, *H*-19/*H*-33/*H*-37), 2.52 – 2.51 (m, 1H, *H*-22), 2.46 – 2.45 (m, 2H, *H*-19/*H*-33/*H*-37), 2.06 – 1.65 (m, 18H, *H*-3, *H*-7, *H*-16, *H*-49, *H*-51, *H*-52), 1.51 – 1.50 (m, 1H, *H*-7), 1.43 – 1.39 (m, 2H, *H*-8), 1.35 (s, 9H, *H*-12), 1.25 – 1.16 (m, 6H, *H*-41, *H*-45), 0.89 – 0.67 (m, 30H, *H*-4, *H*-20, *H*-34, *H*-38, *H*-53); **^{13}C-NMR** (DMSO-d_6, 150 MHz): *δ* [ppm] 172.9 (q, *C*=O), 172.1 (q, *C*=O), 171.7 (q, *C*=O), 171.0 (q, *C*=O), 170.9 (q, *C*=O), 170.8 (q, *C*=O), 170.6 (q, *C*=O), 169.7 (q, *C*=O), 164.9 (q, *C*=O), 164.5 (q, *C*=O), 163.2 (q, *C*=O), 157.1 (q, *C*-27), 155.6 (q, *C*-10), 134.4 (q, 2x *C*-14, *C*-47), 134.1 (q, *C*-24), 133.8 (t, *C*-29), 127.9 (t, 2x *C*-25), 125.5 (q, *C*-15/*C*-48), 125.1 (q, *C*-15/*C*-48), 117.2 (s, *C*-30), 114.1 (t, 2x *C*-26), 77.4 (q, *C*-11), 68.1 (s, *C*-28), 58.2 (t, *C*-2/*C*-18/*C*-32/*C*-36), 57.8 (t, *C*-2/*C*-18/*C*-32/*C*-36), 57.5 (t, *C*-2/*C*-18/*C*-32/*C*-36), 57.3 (t, *C*-2/*C*-18/*C*-32/*C*-36), 52.3 (t, *C*-6), 51.7 (t, *C*-40), 49.5 (t, *C*-23), 45.1 (t, *C*44), 44.2 (s, *C*-51), 41.8 (s, *C*-22), 40.0 (s, *C*-9), 30.4 (p, *C*-42), 30.3 (t, *C*-3/*C*-19/*C*-33/*C*-37), 30.1 (t, *C*-3/*C*-19/*C*-33/*C*-37), 30.0 (t, *C*-3/*C*-19/*C*-33/*C*-37), 29.9 (s, *C*-7), 29.6 (t, *C*-3/*C*-19/*C*-33/*C*-37), 28.3 (s, 3x *C*-12), 25.8 (s, *C*-8), 25.5 (t, *C*-52), 22.3 (p, 2x *C*-53), 20.7 (p, *C*-16/*C*-49), 20.7 (p, *C*-16/*C*-49), 20.3 (p, *C*-16/*C*-49), 20.1 (p, *C*-16/*C*-49), 19.2 (p, *C*-4/*C*-20/*C*-34/*C*-38), 19.2 (p, *C*-4/*C*-20/*C*-34/*C*-38), 19.1 (p, *C*-4/*C*-20/*C*-34/*C*-38), 19.1 (p, *C*-4/*C*-20/*C*-34/*C*-38), 18.9 (p, *C*-4/*C*-20/*C*-34/*C*-38), 18.4 (p, *C*-4/*C*-20/*C*-34/*C*-38), 18.3 (p, *C*-4/*C*-20/*C*-34/*C*-38), 18.2 (p, *C*-4/*C*-20/*C*-34/*C*-38), 17.4 (p, *C*-41/45), 14.4 (p, *C*-41/45); **HRMS** (ESI) *m/z* calculated for $C_{64}H_{104}N_{11}O_{15}$ $[M+H]^+$ 1266.7713; found 1266.7704.

Fragment C

The synthesis of the methyl ester of fragment C is described in F. Gille, A. Kirschning, *Beilstein J. Org. Chem*. **2016**, *12*, 564–570:

((*E*)-2-((*R*)-2-((*tert*-Butoxycarbonyl)amino)-3-methylbutanamido)-3-methylpent-2-enoyl)-D-alanine (fragment C)

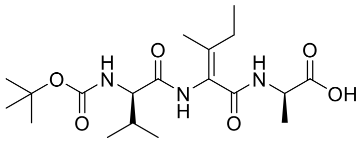

fragment C

Methyl ester **S17** (100 mg, 0.24 mmol, 1.00 equiv.) was dissolved in THF (2.50 mL) and LiOH (1M in H_2O, 2.50 mL, 2.50 mmol, 10.3 equiv.) was added dropwise at 0 °C. The reaction was stirred at rt for 20 h. H_2O and Et_2O were added and the aqueous phase was washed with Et_2O (2x). The aqueous phase was acidified with a 1M HCl solution and extracted with EtOAc (4x). The combined organic phases were dried over $MgSO_4$, filtered and concentrated under reduced pressure to furnish fragment C (96.6 mg, 0.24 mmol, quant.), which was used in the next step without further purification.

Fragment D

Methyl (*tert*-butoxycarbonyl)-L-serinate (S18)

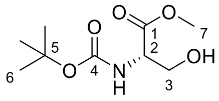

S18

L-Serine (15.0 g, 143 mmol, 1.00 equiv.) was suspended in MeOH (300 mL) and $SOCl_2$ (61.7 mL, 851 mmol, 6.00 equiv.) was added dropwise at 0 °C. The solution was stirred at rt for 14 h. The solvent was removed under reduced pressure and coevaporated with Et_2O (3x). The residue was dissolved in CH_2Cl_2 (300 mL) and cooled to 0 °C. Then Et_3N (54.0 mL, 358 mmol, 2.50 equiv.) and Boc_2O (34.3 g, 157 mmol, 1.10 equiv.) were added and the reaction mixture was stirred at rt for 16 h before the solvent was removed under reduced pressure. The residue was diluted with EtOAc and washed with a sat. $NaHCO_3$ solution and brine, dried over $MgSO_4$, filtered and concentrated under reduced pressure.

The residue was purified by column chromatography (CH_2Cl_2/MeOH = 100:0 to 95:5) to furnish alcohol **S18** (31.3 g, 143 mmol, quant.) as colorless oil.

^{1}H-NMR ($CDCl_3$, 400 MHz): δ [ppm] 5.47 (bs, 1H, N*H*), 4.38 (m, 1H, *H*-2), 3.92 (ddd, *J* = 11.6, 7.5, 3.9 Hz, 2H, *H*-3), 3.78 (s, 3H, *H*-7), 2.29 (bs, 1H, O*H*), 1.45 (s, 9H, *H*-6).

The analytical data are consistent with those reported in the literature (F. W. Foss Jr., A. H. Snyder, M. D. Davis, M. Rouse, M. D. Okusa, K. R. Lynch, T. L. MacDonald, *Bioorg. Med. Chem.* **2007**, *15*, 663-66).

***tert*-Butyl (*S*)-(1,3-dihydroxy-3-methylbutan-2-yl)carbamate (S19)**

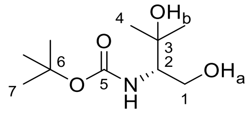

S19

Ester **S18** (14.2 g, 64.6 mmol, 1.00 equiv.) was suspended in Et_2O (350 mL). MeMgBr (3M in Et_2O, 129 mL, 129 mmol, 6.00 equiv.) was added at -78 °C. The mixture was allowed to warm to rt and stirred for 2 h. The reaction was cooled to 0 °C and a sat. NH_4Cl solution was added. The aqueous phase was extracted with EtOAc (3x). The combined organic phases were washed with brine, dried over $MgSO_4$, filtered and concentrated under reduced pressure. The residue was purified by column chromatography (PE/EtOAc = 2:3) to afford diol **S19** (13.4 g, 61.1 mmol, 95% yield) as a colorless solid.

T_M: 62 °C (Lit.: 87-89 °C); **$[\alpha]_D^{22.2}$**: - 4.6 ° (*c* 1.18; MeOH) {Lit.: -4.9 ° (c = 1.00; MeOH)}; **^{1}H-NMR** ($CDCl_3$, 400 MHz): δ [ppm] 5.39 (bs, 1H, N*H*), 4.02 (dd, *J* = 11.4, 2.9 Hz, 1H, *H*-1a), 3.80 (dd, *J* = 11.4, 2.9 Hz, 1H, *H*-1b), 3.46 (s, 1H, *H*-2), 2.56 (bs, 2H, OH_a u. OH_b), 1.45 (s, 9H, *H*-7), 1.35 (s, 3H, *H*-4a), 1.24 (s, 3H, *H*-4b).

The analytical data are consistent with those reported in the literature (J. E. Dettwiler, W. D. Lubell, *J. Org. Chem.* **2003**, *68*, 177-179).

(*R*)-2-((*tert*-Butoxycarbonyl)amino)-3-hydroxy-3-methylbutanoic acid (S20)

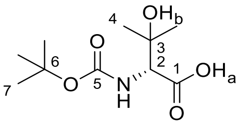

S20

Diol **S19** (6.68 g, 30.5 mmol, 1.00 equiv.) was dissolved in MeCN (50.0 mL). Phosphate buffer (pH = 7, 45.0 mL) and TEMPO (503 mg, 3.04 mmol, 0.10 equiv.) were added. The solution was warmed to 35 °C and $NaClO_2$ (2M in H_2O, 30.5 mL, 61.0 mmol, 2.00 equiv.) and NaOCl (0.04M in H_2O, 15.3 mL, 0.61 mmol, 0.02 equiv.) were added simultaneously over 2 h. The mixture was stirred at 35 °C for 24 h. A TLC check indicated that starting material was still present after 24 h, so further $NaClO_2$ (2M in H_2O, 30.5 mL, 61.0 mmol, 2.00 equiv.) and NaOCl (0.04M in H_2O, 15.3 mL, 0.61 mmol, 0.02 equiv.) were added in the same manner as described above and the reaction was again stirred for 24 h at 35°C. The pH was then lowered to pH = 2 with citric acid solution (10%) and the mixture was extracted with EtOAc (3x). The solvent was removed under reduced pressure and the residue was taken up with sat. $NaHCO_3$ solution. The aqueous phase was washed with EtOAc and the pH was lowered to pH = 2 with 1M phosphoric acid. Subsequently, the aqueous phase was extracted with EtOAc (4x). The combined organic phases were washed with a saturated NaCl solution, dried over $MgSO_4$, filtered, and the solvent was removed under reduced pressure. The residue was coevaporated with CH_2Cl_2 (3x) to furnish product **S20** (5.73 g, 24.6 mmol, 81% yield) as a colorless solid.

T_M: 116 °C (Lit.: 116 °C); **$[\alpha]_D^{22.2}$:** + 2.1 ° (*c* 2.13; MeOH) {Lit.: +2.2 ° (c = 2.97; MeOH)}; **^{1}H-NMR** (CD_3OD, 400 MHz): δ [ppm] 4.08 (s, 1H, *H*-2), 1.45 (s, 9H, *H*-7), 1.29 (s, 3H, *H*-4a), 1.26 (s, 3H, *H*-4b).

The analytical data are consistent with those reported in the literature (J. E. Dettwiler, W. D. Lubell, *J. Org. Chem.* **2003**, *68*, 177-179).

tert-Butyl (4-aminobutyl)carbamate (S21)

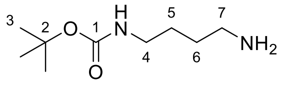

S21

1,4-Diaminobutane (**6**, 7.50 g, 85.1 mmol, 7.70 equiv.) was dissolved in 1,4-dioxane (30.0 mL) and Boc_2O (2.4 g, 11.0 mmol, 1.00 equiv.) in 1,4-dioxane (30.0 mL) was added at rt over 90 min. The solution was stirred at rt for 20 h. The solvent was removed under reduced pressure and H_2O was added to the residue. The mixture was filtered and the filtrate was extracted with CH_2Cl_2 (4x). The combined organic phases were dried over $MgSO_4$, filtered and concentrated under reduced pressure to furnish carbamate **S21** (1.85 g, 9.81 mmol, 89% yield) as colorless oil.

R*f* = 0.10 (CH_2Cl_2/CH_3OH 9:1); **^{1}H-NMR** (CD_3OD, 400 MHz): δ [ppm] 3.04 (2H, t, *J* = 6.5 Hz, *H*-4), 2.63 (2H, t, *J* = 6.5 Hz, *H*-7), 1.48 (4H, t, *J* = 6.5 Hz, *H*-5, *H*-6), 1.43 (9H, s, *H*-3); **^{13}C-NMR** (CD_3OD, 100 MHz): δ [ppm] 158.6 (q, *C*-1), 79.8 (q, *C*-2), 42.3 (s, *C*-7), 41.2 (s, *C*-4), 31.1 (s, *C*-6), 28.8 (p, 3C, *C*-3), 28.3 (s, *C*-5).

The analytical data are consistent with those reported in the literature (C. G. Evans, M. C. Smith, J. P. CArolan, J. E. Gestwicki, *Bioorg. Med. Chem. Lett.* **2011**, *21*, 2587-2590; K. Dąbrowa, M. Pawlak, P. Duszewski, J. Jurczak, *Org. Lett.* **2012**, *14*(24), 6298-6301).

***N*,*N*-Diallyloxycarbonyl-2-methyl-2-thiopseudourea (7)**

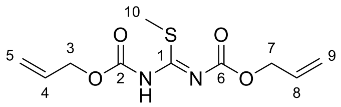

7

S-Methylthiuronium sulfate (5.00 g, 35.9 mmol, 1.00 equiv.) was dissolved in CH_2Cl_2 (50 mL), a 10% $NaHCO_3$ solution (50 mL) and a 5 M NaOH solution (7.20 mL, 35.9 mmol, 1.00 equiv.). Allyl chloroformate (11.5 mL, 108 mmol, 3.00 equiv.) was added dropwise. The mixture was stirred at rt for 3 h. H_2O and CH_2Cl_2 were added and the aqueous phase was extracted with CH_2Cl_2 (3x). The combined organic phases were washed with H_2O and brine, dried over $MgSO_4$, filtered and concentrated under reduced pressure. The residue was purified by column chromatography (PE/CH_2Cl_2 = 6:1, 5:1, 2:1, 0:1) to furnish product **7** (5.09 g, 19.7 mmol, 55% yield) as colorless solid.

T_M: 30-32 °C; **^{1}H-NMR** ($CDCl_3$, 400 MHz): *δ* [ppm] 11.8 (bs, 1H, N*H*), 6.01-5.88 (m, 2H, *H*-4 u. *H*-4‘), 5.37 (dq, *J* = 17.1, 1.4 Hz, 2H, *H*-5a u. *H*-5’a), 5.31-5.25 (m, 2H, *H*-5b u. *H*-5’b), 4.68-4.64 (m, 4H, *H*-3 u. *H*-3‘), 2.43 (s, 3H, *H*-6); **^{13}C-NMR** ($CDCl_3$, 100 MHz): *δ* [ppm] 172.9 (q, *C*-1), 161.0 (q, *C*-2 od. *C*-2‘), 151.6 (q, *C*-2 od. *C*-2‘), 131.6 (2C, t, *C*-4 u. *C*-4‘), 119.3 (2C, s, *C*-5 u. *C*-5’), 67.2 (2C, s, *C*-3 u. *C*-3‘), 14.7 (p, *C*-6).

The analytical data are consistent with those reported in the literature (Patent, Merck Sharp & Dohme LLC, Rahway, NJ, USA, US6140318A1, 2000).

Nα-Boc-*Nθ,Nθ’*-bisalloc-agmatine (8)

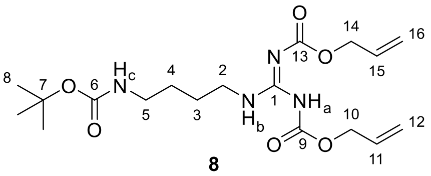

8

tert-Butyl (4-aminobutyl)carbamate (**S21**, 9.52 g, 50.6 mmol, 1.30 equiv.) and reagent **7** (10.1 g, 39.1 mmol, 1.00 equiv.) were dissolved in THF (400 mL). Et_3N (27.1 mL, 196 mmol, 5.00 equiv.) was added and the mixture was stirred at rt for 72 h. A 10% Na_2CO_3 solution was added and the organic solvent was removed under reduced pressure. The aqueous phase was extracted with EtOAc (3x) and the combined organic phases were washed with a 1M HCl solution and brine, dried over $MgSO_4$, filtered and concentrated under reduced pressure. The residue was purified by column chromatography (CH_2Cl_2/MeOH = 100:1, 99:1, 98:2) to furnish product **8** (14.5 g, 36.4 mmol, 93% yield) as colorless solid that was used in the next step without further purification and characterization.

4-(2,3-Bis((allyloxy)carbonyl)guanidino)butan-1-aminium iodide (S22)

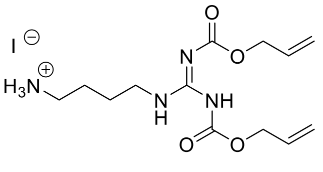

S22

Carbamate **8** (1.00 g, 2.51 mmol, 1.00 equiv.) was dissolved in CH_2Cl_2 (25.0 mL). TMSI (0.29 mL, 2.76 mmol, 1.10 equiv.) was added. The solution was stirred at rt for 5 min. MeOH was added and the solution was concentrated and coevaporated with CH_2Cl_2

(3x 5.00 mL) under reduced pressure. The crude product **S22** was used in the next step without further purification.

(*N*-Boc-D-3-hydroxyvalyl)-*Nθ,Nθ'*-bisalloc-*Nα*-agmatide (S23)

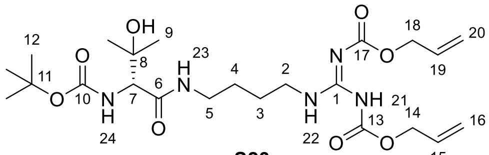

S23

To a solution of *N*-Boc-3-hydroxy-D-valine (**S20**, 532 mg, 2.28 mmol, 1.00 equiv.) and iodide **S22** (1.00 g, 2.51 mmol, 1.10 equiv.) in CH_2Cl_2 (30.0 mL) and DMF (5.00 mL), Oxyma (486 mg, 3.42 mmol, 1.50 equiv.), EDC·HCl (546 mg, 2.85 mmol, 1.30 equiv.) and $NaHCO_3$ (957 mg, 11.4 mmol, 5.00 equiv.) were added at 0 °C and the reaction mixture was stirred at rt for 20 h. An 1M HCl solution and EtOAc were added, and the organic phase was washed with an 1M HCl solution (2x), a sat. $NaHCO_3$ solution (2x) and brine, dried over $MgSO_4$, filtered and concentrated under reduced pressure. The residue was purified by flash column chromatography (MeOH in CH_2Cl_2 = 0 → 1.5%) to give product **S23** (509 mg, 991 µmol, 43% yield) as colorless oil.

^{1}H-NMR ($CDCl_3$, 400 MHz): δ [ppm] 11.80 (s, 1H, *H*-21), 8.43 (t, *J* = 5.1 Hz, 1H, *H*-22), 6.71 (s, 1H, *H*-23), 6.02 – 5.85 (m, 2H, *H*-15, *H*-19), 5.55 (d, *J* = 8.6 Hz, 1H, *H*-24), 5.39 – 5.29 (m, 3H, *H*-16, *H*-20), 5.24 – 5.21 (m, 1H, *H*-20), 4.66 – 4.60 (m, 4H, *H*-14, *H*-18), 4.18 (s, 1H, O*H*), 3.84 (d, *J* = 9.1 Hz, 1H, *H*-7), 3.46 (q, *J* = 6.8 Hz, 2H, *H*-2), 3.31 (q, *J* = 6.0 Hz, 2H, *H*-5), 1.63 – 1.57 (m, 4H, *H*-3, *H*-4), 1.42 (s, 9H, *H*-12), 1.29 (s, 3H, *H*-9), 1.18 (s, 3H, *H*-9); **^{13}C-NMR** ($CDCl_3$, 100 MHz): δ [ppm] 172.5 (q, *C*-6), 163.2 (q, *C*-17), 156.4 (q, *C*-1/*C*-10), 156.1 (q, *C*-1/*C*-10), 153.9 (q, *C*-13), 132.9 (t, *C*-19), 131.0 (t, *C*-15), 119.7 (s, *C*-16), 118.2 (s, *C*-20), 80.4 (q, *C*-11), 71.8 (q, *C*-8), 67.3 (s, *C*-14), 66.6 (s, *C*-18), 60.0 (t, *C*-7), 40.8 (s, *C*-2), 38.9 (s, *C*-5), 28.4 (3C, p, *C*-12), 27.6 (p, *C*-9a), 26.5 (s, *C*-3/*C*-4), 26.4 (s, *C*-3/*C*-4), 25.5 (p, *C*-9b); **HRMS** (ESI): *m/z* calculated for $C_{23}H_{39}N_5O_8Na$ $[M+Na]^+$: 536.2696; found: 536.2689.

(*R*,*Z*)-7-(((allyloxy)carbonyl)amino)-16-hydroxy-16-methyl-5,14-dioxo-4-oxa-6,8,13-triazaheptadeca-1,6-dien-15-aminium trifluoroacetate (fragment D)

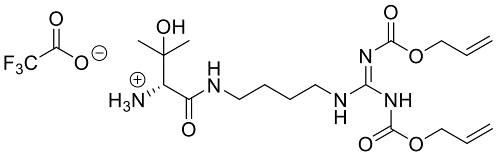

fragment D

Carbamate **S23** (62.0 mg, 0.12 mmol, 1.00 equiv.) was dissolved in CH_2Cl_2 (1.20 mL) and TFA (0.50 mL, 6.03 mmol, 50.0 equiv.) was added at 0 °C. The solution was stirred at 0 °C for 2 h. All volatiles were removed under reduced pressure and the residue was coevaporized with MeOH (2x, 0 mbar, rt) and CH_2Cl_2 (20 mbar, 40 °C). The crude product was used in the next step without further purification.

Fragment CD

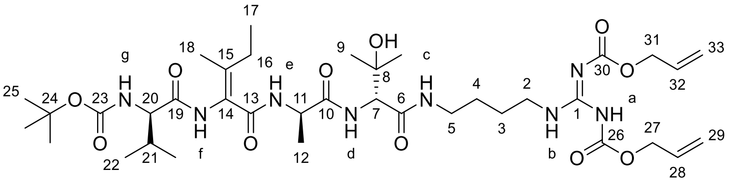

S24

Fragment C (79.0 mg, 0.20 mmol, 1.50 equiv.) and fragment D (68.0 mg, 0.13 mmol, 1.00 equiv.) were dissolved in MeCN (1.00 mL) and DMF (0.50 mL). EDC·HCl (47.0 mg, 0.25 mmol, 1.90 equiv.) and HOAt (40.4 mg, 0.30 mmol, 2.30 equiv.) in DMF (1.00 mL) were added at -15 °C dropwise over 10 min. $NaHCO_3$ (76.0 mg, 0.90 mmol, 7.00 equiv.) was added and the mixture was stirred at rt for 20 h. The reaction mixture was diluted with H_2O and MeOH. The solution was subjected to flash column chromatography (RP- BÜCHI, solvent A: water + 0.1% FA, solvent B: MeOH + 0.1% FA, 15x150 mm column, flow rate: 10.0 mL/min, 60 sec/fr., gradient: (*t* [min]/solvent B [%]): 0/10; 5/10; 50/100; 60/100; t_R = 38.0-46.0 min) to furnish product **S24** (62.0 mg, 0.08 mmol, 60%, *d.r.* = 14:1) as colorless film.

$[\alpha]_D^{26}$: - 3.0° (*c* 3.27, CH_2Cl_2); **$[\alpha]_D^{28}$**: + 9.5° (*c* 2.53, MeOH); **^{1}H-NMR** (DMSO-d$_6$, 400 MHz): δ [ppm] 11.57 (bs, 1H, NH_a), 9.27 (s, 1H, NH_f), 8.37 (t, J = 5.0 Hz, 1H, NH_b), 7.89 (d, J = 7.0 Hz, 1H, NH_e), 7.76 (d, J = 9.4 Hz, 1H, NH_d), 7.72 (t, J = 5.4 Hz, 1H, NH_c), 6.78 (d, J = 7.1 Hz, 1H, NH_g), 5.95 (ddt, J = 17.2, 10.8, 5.4 Hz, 1H, *H*-28), 5.93 (ddt,

J = 17.1, 10.6, 5.3 Hz, 1H, *H*-32), 5.39-5.32 (m, 1H, *H*-29a), 5.31-5.23 (m, 2H, *H*-29b, *H*-33a), 5.20-5.15 (m, 1H, *H*-33b), 4.76 (bs, 1H, OH), 4.68-4.64 (m, 2H, *H*-27), 4.51-4.47 (m, 2H, *H*-31), 4.28 (p, *J* = 7.2 Hz, 1H, *H*-11), 4.22 (d, *J* = 9.8 Hz, 1H, *H*-7), 3.86 (t, *J* = 7.3 Hz, 1H, *H*-20), 3.34-3.27 (m, 2H, *H*-2), 3.16 (dq, *J* = 13.0, 6.2 Hz, 1H, *H*-5a), 3.00 (dq, *J* = 12.4, 6.4 Hz, 1H, *H*-5b), 2.35-2.18 (m, 2H, *H*-16), 1.96 (oct, *J* = 6.6 Hz, 1H, *H*-21), 1.68 (s, 3H, *H*-18), 1.55-1.45 (m, 2H, *H*-3), 1.45-1.34 (m, 12H, *H*-4, *H*-25), 1.24 (d, *J* = 7.2 Hz, 3H, *H*-12), 1.11 (s, 3H, *H*-9a), 1.09 (s, 3H, *H*-9b), 0.99 (t, *J* = 7.5 Hz, 3H, *H*-17), 0.88 (d, *J* = 6.8 Hz, 3H, *H*-22a), 0.86 (d, *J* = 6.8 Hz, 3H, *H*-22b); **^{13}C-NMR** (DMSO-d_6 ,100 MHz): δ [ppm] 172.0 (q, *C*-10), 171.1 (q, *C*-19), 169.9 (q, *C*-6), 165.4 (q, *C*-13), 162.9 (q, *C*-30), 155.7 (q, *C*-23), 155.1 (q, *C*-1), 152.5 (q, *C*-26), 138.1 (q, *C*-14), 133.5 (t, *C*-32), 131.9 (t, *C*-28), 125.1 (q, C15), 118.8 (t, *C*-29), 117.4 (t, *C*-33), 78.3 (q, *C*-24), 71.0 (q, *C*-8), 66.5 (s, *C*-27), 65.4 (s, *C*-31), 59.9 (t, *C*-7/*C*-20), 59.8 (t, *C*-7/*C*-20), 48.9 (t, *C*-11), 40.1 (s, *C*-2), 38.1 (s, *C*-5), 30.2 (t, *C*-21), 28.1 (p, *C*-25), 27.3 (p, *C*-9a), 26.3 (s, *C*-16), 26.1 (p, *C*-9b) 26.1 (s, *C*-4), 25.9 (s, *C*-3), 19.1 (p, *C*-22a), 18.4 (p, *C*-22b), 17.5 (p, *C*-12, *C*-18), 12.6 (p, *C*-17); **HRMS** (ESI): *m/z* calculated for $C_{37}H_{63}N_8O_{11}$ [M+H]$^+$: 795.4616; found: 795.4614.

Fragment CD

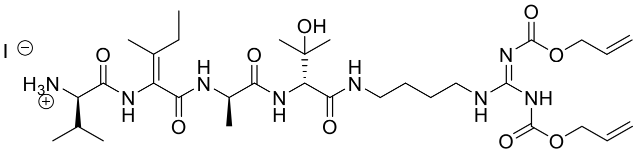

fragment CD

To a solution of carbamate **S24** (306 mg, 386 μmol, 1.00 equiv.) in CH_2Cl_2 (3.86 mL), TMSI (105 μL, 771 μmol, 2.00 equiv.) was added dropwise. The reaction was stirred at rt for 1 h. MeOH was added, and the solution was concentrated and coevaporated with CH_2Cl_2 (3x 5.00 mL) under reduced pressure. The crude fragment CD was used in the next step without further purification

Fragment ABCD

Boc-Protected Fragment ABCD-D,L (S25)

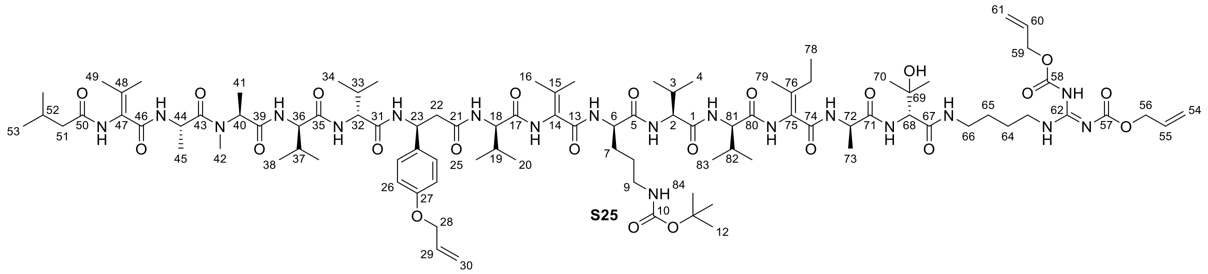

S25

To a solution of fragment AB-D,L (4.60 mg, 3.63 μmol, 1.00 equiv.) and fragment CD (3.03 mg, 4.36 μmol, 1.20 equiv.) in DMF (500 μL) at 0 °C, HOAt (740 mg, 76.6 μmol, 1.50 equiv.), HATU (29.1 mg, 76.6 μmol, 2.50 equiv.) and DIPEA (26.7 μL, 5.45 μmol, 5.00 equiv.) were added and the reaction mixture was stirred for 17 h at ambient temperature. The reaction was terminated with MeOH (1.00 mL) and the solvent was removed under reduced pressure. Purification by preparative HPLC (solvent A: water + 0.1% FA, solvent B: MeCN + 0.1% FA; flow rate: 15.0 mL/min; gradient: (*t* [min]/solvent B [%]): 0/20; 80/100; 100/100; t_R = 19.0 – 22.0 min) afforded Boc-protected fragment ABCD-D,L (**S25**, 3.90 mg, 2.00 μmol, 55% yield) as a rose foam.

[α]$_D^{21.3}$: + 1.0° (*c* 0.1, DMSO-d$_6$); **^{1}H-NMR** (DMSO-d$_6$, 600 MHz): δ [ppm] 9.33 – 8.88 (m, 3H, N*H*), 8.51 – 8.32 (m, 2H, N*H*), 8.31 – 8.15 (m, 2H, N*H*), 8.12 – 8.04 (m, 1H, N*H*), 7.89 – 7.69 (m, 6H, N*H*), 7.62 – 7.60 (m, 2H, N*H*), 7.23 – 7.16 (m, 2H, *H*-25), 6.81 – 6.80 (d, *J* = 8.7 Hz, 2H, *H*-26), 6.72 – 6.66 (m, 1H, N*H*-84), 6.04 – 5.90 (m, 3H, *H*-29, *H*-55, *H*-60), 5.37 – 5.15 (m, 8H, *H*-23, *H*-30, *H*-54, *H*-61, O*H*), 5.14 – 5.01 (m, 1H, *H*-40), 4.71 – 4.66 (m, 3H, *H*-44, *H*-56, *H*-59), 4.50 – 4.49 (m, 4H, *H*-28, *H*-56, *H*-59), 4.37 – 4.12 (m, 10H, *H*-2, *H*-6, *H*-18, *H*-32, *H*-36, *H*-63, *H*-68, *H*-72, *H*-81), 3.17 – 2.99 (m, 2H, *H*-66), 2.93 – 2.73 (m, 5H, *H*-9, *H*-42), 2.72 – 2.63 (m, 1H, *H*-22), 2.54 – 2.51 (m, 6H, *H*-3, *H*-19, *H*-22, *H*-33, *H*-37, *H*-82), 2.30 – 2.24 (m, 2H, *H*-77), 2.07 – 1.60 (m, 18H, *H*-16, *H*-49, *H*-51, *H*-52, *H*-79), 1.51 – 1.49 (m, 3H, *H*-7, *H*-64), 1.41 – 1.39 (m, 4H, *H*-8, *H*-65), 1.35 (s, 9H, *H*-12), 1.23 – 1.16 (m, 9H, *H*-41, *H*-45, *H*-73), 1.10 – 1.05 (m, 6H, *H*-70), 0.96 – 0.96 (m, 3H, *H*-78), 0.89 – 0.73 (m, 36H, *H*-4, *H*-20, *H*-34, *H*-38, *H*-53, *H*-83); **^{13}C-NMR** (DMSO-d$_6$, 150 MHz): δ [ppm] 173.1 (q, *C*=O), 171.9 (q, *C*=O), 171.5 (q, *C*=O), 170.8 (q, *C*=O), 170.8 (q, *C*=O), 170.5 (q, *C*=O), 170.5 (q, *C*=O), 170.5 (q, *C*=O), 169.8 (q, *C*=O), 169.6 (q, *C*=O),

169.5 (q, *C*=O), 169.3 (q, *C*=O), 165.0 (q, *C*=O), 165. 0 (q, *C*=O), 164.9 (q, *C*=O), 162.9 (q, *C*-57/*C*-58), 156.9 (q, *C*-27), 155.5 (q, *C*-10), 155.0 (q, *C*-62), 152.5 (q, , *C*-57/*C*-58), 134.3 (3x q, *C*-14, *C*-47, *C*-75), 134.1 (q, *C*-24), 133.7 (t, *C*-29/*C*-55/*C*-60), 133.5 (t, *C*-29/*C*-55/*C*-60), 131.8 (t, *C*-29/*C*-55/*C*-60), 127.5 (t, 2x *C*-25), 125.4 (q, *C*-15/*C*-48/*C*-76), 125.2 (q, *C*-15/*C*-48/*C*-76), 124.7 (q, *C*-15/*C*-48/*C*-76), 118.8 (s, *C*-30/*C*-54/*C*-61), 117.3 (s, *C*-30/*C*-54/*C*-61), 117.2 (s, *C*-30/*C*-54/*C*-61), 114.1 (t, 2x *C*-26), 77.3 (q, *C*-11), 70.9 (q, *C*-69), 68.0 (s, *C*-28), 66.4 (s, *C*-56/*C*-59), 65.4 (s, *C*-56/*C*-59), 59.9 (t, *C*-2/*C*-18/*C*-32/*C*-36/*C*-68/*C*-81), 59.8 (t, *C*-2/*C*-18/*C*-32/ *C*-36/*C*-68/*C*-81), 58.3 (t, *C*-2/*C*-18/*C*-32/*C*-36/*C*-68/*C*-81), 57.6 (t, *C*-2/*C*-18/*C*-32/*C*-36/*C*-68/ *C*-81), 57.6 (t, *C*-2/*C*-18/*C*-32/*C*-36/*C*-68/*C*-81), 57.4 (t, *C*-2/*C*-18/*C*-32/*C*-36/*C*-68/*C*-81), 52.5 (t, *C*-6/*C*-23/*C*-40/*C*-44/*C*-72), 51.6 (t, *C*-6/*C*-23/*C*--40/*C*44/*C*-72), 49.4 (t, *C*-6/*C*-23/*C*-40/*C*-44/ *C*-72), 48.8 (t, *C*-6/*C*-23/*C*-40/*C*-44/*C*-72), 45.0 (t, *C*-6/*C*-23/*C*-40/*C*-44/*C*-72), 44.2 (s, *C*-51), 41.7 (s, *C*-22), 40.1 (s, *C*-9 and *C*-63 beneath DMSO-signal), 38.1 (s, *C*-66), 30.7 (t, *C*-3/*C*-19/*C*-33/ *C*-37/*C*-82), 30.4 (t, *C*-3/*C*-19/*C*-33/*C*-37/*C*-82), 30.3 (t, *C*-3/*C*-19/*C*-33/*C*-37/*C*-82), 30.1 (t, *C*-3/*C*-19/*C*-33/*C*-37/*C*-82), 29.6 (t, *C*-3/*C*-19/*C*-33/*C*-37/*C*-82 and s, *C*-7), 28.2 (s, 3x *C*-12), 27.3 (p, *C*-70), 26.2 (s, *C*-77), 26.1 (s, *C*-65), 26.0 (s, *C*-64), 25.8 (s, *C*-8), 25.5 (t, *C*-53), 22.3 (p, 2x *C*-53), 20.6 (p, *C*-16/*C*-49), 20.4 (p, *C*-16/*C*-49), 20.2 (p, *C*-16/*C*-49), 20.0 (p, *C*-16/*C*-49), 19.3 (p, *C*-4/*C*-20/*C*-34/*C*-38/*C*-83), 19.2 (p, *C*-4/*C*-20/*C*-34/*C*-38/*C*-83), 19.1 (2x p, *C*-4/*C*-20/*C*-34/ *C*-38/*C*-83), 19.0 (p, *C*-4/*C*-20/*C*-34/*C*-38/*C*-83), 19.0 (p, *C*-4/*C*-20/*C*-34/*C*-38/*C*-83), 18.4 (p, *C*-4/*C*-20/*C*-34/*C*-38/*C*-83), 18.2 (p, *C*-4/*C*-20/*C*-34/*C*-38/*C*-83), 18.1 (p, *C*-4/*C*-20/*C*-34/*C*-38/ *C*-83), 18.0 (p, *C*-4/*C*-20/*C*-34/*C*-38/*C*-83), 17.8 (p, *C*-41/*C*-45/*C*-73/*C*-79), 17.6 (p, *C*-41/*C*-45/ *C*-73/*C*-79), 17.4 (p, *C*-41/*C*-45/*C*-73/*C*-79), 14.5 (p, *C*-41/*C*-45/*C*-73/*C*-79), 12.6 (p, *C*-78); **HRMS** (ESI) *m/z* calculated for $C_{96}H_{155}N_{19}O_{23}$ $[M+2H/2]^+$ 972.0850; found 972.0850.

Boc-Deprotected Fragment ABCD-D,L (S26)

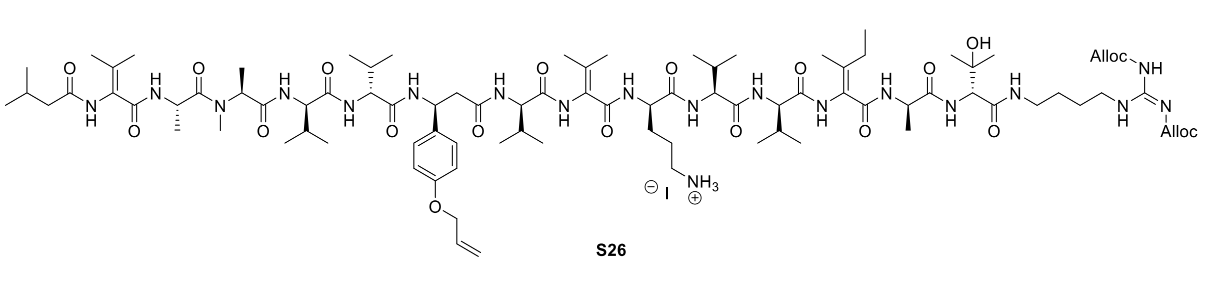

S26

To a solution of Boc-protected fragment **S25** (20.0 mg, 10.3 *µ*mol, 1.00 equiv.) in CH_2Cl_2 (650 *µ*L), TMSI (7.00 *µ*L, 51.5 *µ*mol, 5.00 equiv.) was added and the reaction mixture was stirred for 1 h at ambient temperature. Then the solvent was removed under reduced

pressure. affording deprotected peptide **S26** as a yellow foam that was used in the next step without further purification.

Guanidinylated Fragment ABCD-D,L (S27)

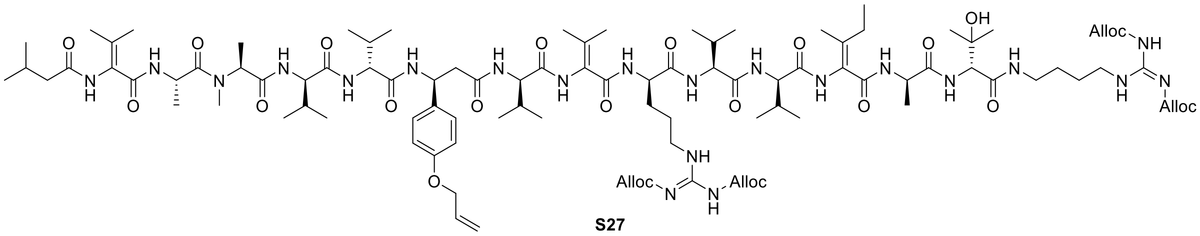

S27

To a solution of Boc-deprotected fragment ABCD-D,L (**S26**, 10.3 µmol, 1.00 equiv.) and *N,N*'-bisalloc-*S*-methylisothiourea (**7**, 10.6 mg, 41.2 µmol, 4.00 equiv.) in THF (700 µL) at 0 °C, Et_3N (7.10 µL, 51.5 µmol, 5.00 equiv.) was added dropwise and the reaction mixture was stirred for 16 h at ambient temperature. The reaction was terminated with H_2O (1.00 mL) and the solvent was removed under reduced pressure. Purification by flash column chromatography (RP-BÜCHI; solvent A: water + 0.1% FA, solvent B: MeCN + 0.1% FA, 4 g WP C18 column, flow rate: 10.0 mL/min, 30 sec/fr., gradient: (*t* [min]/solvent B [%]): 0/0; 22/95; 30/95; t_R = 17 – 33 min) afforded guanidinylated fragment ABCD-D,L (**S27**, 21.1 mg, 10.3 µmol, quant.) as a colorless foam that was used in the next step without further purification.

HRMS (ESI) *m/z* calculated for $C_{100}H_{159}N_{21}O_{25}$ [M+2H/2]$^+$ 1027.0908; found 1027.0896.

Myxovalargin A / Myxovalargin-D,L

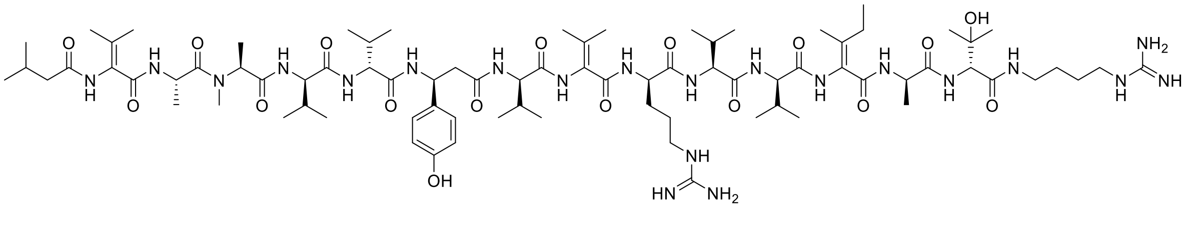

myxovalargin A / myxovalargin-D,L

To a solution of guanidinylated fragment ABCD-D,L (**S27**, 34.1 mg, 16.6 µmol, 1.00 equiv.) and phenylsilane (12.3 µL, 99.6 µmol, 6.00 equiv.) in CH_2Cl_2 (400 µL), $Pd(PPh_3)_4$ (3.80 mg, 3.30 µmol, 0.20 equiv.) was added and the reaction mixture was stirred for 2 h at ambient temperature. The reaction was terminated with H_2O (500 µL) and MeOH (500 µL) and the solvent was removed under reduced pressure. Purification by flash column

chromatography (RP-BÜCHI; solvent A: water + 0.1% FA, solvent B: MeCN + 0.1% FA, 12 g WP C18 column, flow rate: 20.0 mL/min, 30 sec/fr., gradient: (*t* [min]/solvent B [%]): 0/0; 25/95; 30/95; t_R = 14.5 – 25.0 min) afforded myxovalargin-D,L (31.4 mg, 18.7 *µ*mol, 33% yield o3s) as a colorless foam.

^{1}H-NMR (DMSO-d$_6$, 600 MHz): *δ* [ppm] 9.31 – 8.92 (m, 1H, N*H*), 8.46 – 8.19 (m, 4H, N*H*), 7.80 – 7.27 (m, 16H, N*H*, Tyr-O*H*), 7.09 – 7.07 (m, 2H, Ar-*H*), 6.62 – 6.61 (m, 2H, Ar-*H*), 5.22 – 4.67 (m, 3H, NC*H*), 4.39 – 4.09 (m, 9H, NC*H*), 3.12 – 3.04 (m, 4H, NCH_3, CH_2, HCC*H*), 2.86 – 2.82 (m, 2H, NCH_3, CH_2, HCC*H*), 2.74 – 2.65 (m, 1H, NCH_3, CH_2, HCC*H*), 2.52 – 2.51 (m, 1H, CH_2, HCC*H,* CH_3), 2.29 – 2.28 (m, 2H, C*H*/CH_2/,CH_3), 2.04 – 1.96 (m, 2H, CH_2/CH_3), 1.96 – 1.93 (m, 4H, CH_2/CH_3), 1.90 (m, 8H, CH_2/CH_3), 1.67 – 1.54 (m, 13H, CH_2/CH_3), 1.44 – 1.38 (m, 4H, CH_2/CH_3), 1.21 – 1.16 (m, 11H, CH_2/CH_3), 1.11 – 1.09 (m, 6H, CH_3), 0.98 (m, 3H, H_2CCH_3), 0.86–– 0.78 (m, 36H, CH_3); **^{13}C-NMR** (DMSO-d$_6$, 600 MHz): *δ* [ppm] 174.2 (q, *C*=O), 172.0 (q, *C*=O), 171.9 (q, *C*=O), 170.9 (q, *C*=O), 170.8 (q, *C*=O), 170.7 (q, *C*=O), 170.5 (q, *C*=O), 169.9 (q, *C*=O), 169.7 (q, *C*=O), 166.6 (q, *C*=O), 166.4 (q, *C*=O), 157.1 (q, OAr-*C*), 157.1 (q, *C*=N), 133.4 (q, *C*=C), 133.3 (q, *C*=C), 131.5 (q, *C*=C), 131.4 (q, *C*=C), 128.8 (q, *C*=C), 128.7 (q, *C*=C), 128.0 (q, *C*=C), 127.7 (q, *C*=C), 114.6 (q, *C*=C), 70.9 (q, HO*C*), 60.1 (t, N*C*H), 57.9 (t, N*C*H), 57.4 (t, N*C*H), 57.3 (t, N*C*H), 57.0 (t, N*C*H), 49.4 (t, N*C*H), 44.4 (t, N*C*H), 44.2 (t, N*C*H), 40.4 (t, N*C*H), 30.4 (s, *C*H_2), 29.1 (t, H_3C*C*H), 28.8 (t, H_3C*C*H), 28.7 (t, H_3C*C*H), 28.7 (t, H_3C*C*H), 28.6 (t/s, H_3C*C*H/*C*H_2), 27.4 (t/s, H_3C*C*H/*C*H_2), 26.3 (t/s, H_3C*C*H/*C*H_2), 26.1 (t/s, H_3C*C*H/*C*H_2), 25.8 (s, t/s, H_3C*C*H/*C*H_2), 25.1 (s/p, *C*H_2/*C*H_3), 22.3 (s/p, *C*H_2/*C*H_3), 20.3 (s/p, *C*H_2/*C*H_3), 19.2 (s/p, *C*H_2/*C*H_3), 19.2 (s/p, *C*H_2/*C*H_3), 19.1 (s/p, *C*H_2/*C*H_3), 18.4 (s/p, *C*H_2/*C*H_3), 18.0 (s/p, *C*H_2/*C*H_3), 17.5 (p, *C*H_3), 17.4 (p, *C*H_3), 17.0 (p, *C*H_3), 14.5 (p, *C*H_3), 12.7 (p, H_2C*C*H_3); **HRMS** (ESI) *m/z* calculated for $C_{181}H_{137}N_{21}O_{17}$ [M+2H/2]$^+$ 839.0329; found 839.0330.

High performance liquid chromatography of myxovalargin (D/L-epimer): An Alliance 2695 HPLC-system by WATERS with a WATERS 996 diode array detector (λ = 200-350 nm) and a Nucleodur C18 HTec column (5 *µ*m, 250 mm, Ø 8 mm) by MACHEREY-NAGEL were used. The eluant was 2-propanol / acetonitrile 3% in a pH 6.0 trimethyl formiate buffer (1:1:1.5).

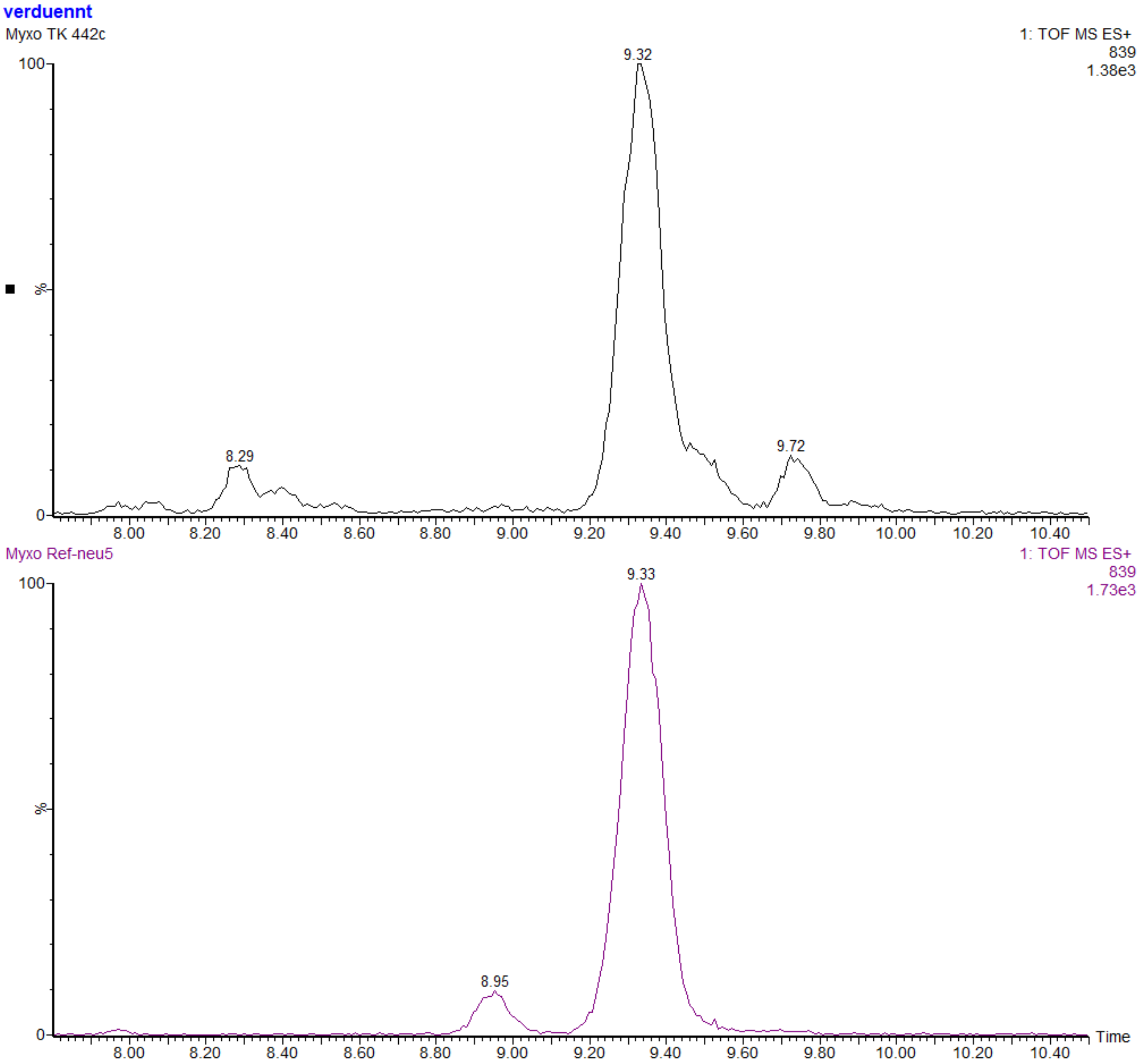

Figure S5-1: Top chromatogram: Synthetic Myxovalargin A / Myxovalargin D,L-epimer; Bottom chromatogram: Authentic sample of Myxovalargin A.

Fragment B-L,D

($N\alpha$-Fmoc-$N\delta$-Boc-D-ornityl)-D-valinemethylester (S28)

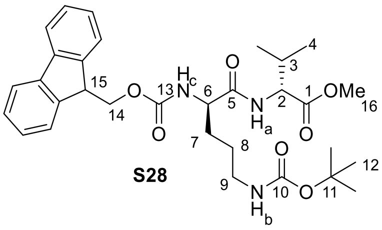

Fmoc-D-Orn(Boc)-OH (1.71 g, 3.76 mmol, 1.00 equiv.), D-Val-OMe·HCl (760 mg, 4.51 mmol, 1.20 equiv.), HOAt (560 mg, 4.14 mmol, 1.10 equiv.) and EDC·HCl (790 mg, 4.14 mmol, 1.10 equiv.) were dissolved in anhydrous CH_2Cl_2 (25.0 mL) and cooled to 0 °C. $NaHCO_3$ (1.58 g, 18.8 mmol, 5.00 equiv.) was added and the reaction was stirred for 16 h while the mixture was warmed to rt. The mixture was then filtered, the solvent was removed under reduced pressure, and the residue was purified by column chromatography (CH_2Cl_2/MeOH, 100:0 -> 99:1 -> 98:2 > 97.5:2.5). Product **S28** (1.98 g, 3.49 mmol, 93% yield) was obtained as a colorless foam.

$[\alpha]_D^{26.6}$: + 0.5° (*c* 0.99; CH_2Cl_2) {Lit.: -1.8° (*c* 1.44, CH_2Cl_2)}; **^{1}H-NMR** ($CDCl_3$, 400 MHz): *δ* [ppm] 7.77-7.73 (m, 2H, Ar-H, Fmoc), 7.61-7.57 (m, 2H, Ar-H, Fmoc), 7.42-7.36 (m, 2H, Ar-H, Fmoc), 7.32-7.27 (m, 2H, Ar-H, Fmoc), 6.79 (bs, 1H, NH_a), 5.71 (m, 1H, NH_c), 4.76 (bs, 1H, NH_b), 4.51 (dd, *J* = 8.9, 5.1 Hz, 1H, *H*-2), 4.42-4.32 (m, 2H, *H*-6 u. *H*-14), 4.20 (t, *J* = 7.1 Hz, 1H, *H*-14), 3.71 (s, 3H, *H*-16), 3.33-3.20 (m, 1H, *H*-9a), 3.16-3.05 (m, 1H, *H*-9b), 2.18 (okt, *J* = 6.6 Hz, 1H, *H*-3), 2.01-1.83 (m, 1H, *H*-7a), 1.71-1.47 (m, 3H, *H*-7b u. *H*-8), 1.43 (s, 9H, *H*-12), 0.92 (d, *J* = 7.0 Hz, 3H, *H*-4a), 0.90 (d, *J* = 6.7 Hz, 3H, *H*-4b).

The analytical data are consistent with those reported in the literature (F. Gille, A. Kirschning, *Beilstein J. Org. Chem*. **2016**, *12*, 564–570).

(*N*-Fmoc-(*R,S*)-3-Nitrovalyl)-($N\delta$-Boc-d-ornityl)-D-valinemethylester (S30)

Fmoc-*Deprotection:*

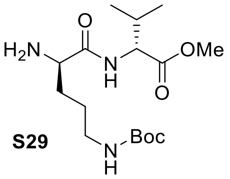

To a solution of carbamate **S28** (1.50 g, 2.65 mmol, 1.00 equiv.) in anhydrous CH_2Cl_2 (26.5 mL) tris(2-aminoethyl-)amine (2.65 mL) was added over 10 min at 0 °C. Then, the mixture was warmed to rt and stirred for 3 h at ambient temperature. The reaction was then terminated by addition of silica gel and the solvent was removed under reduced pressure. Purification of the residue by column chromatography (CH_2Cl_2, 1% MeOH, 1% Et_3N) afforded the product **S29** as a colorless oil containing residues of Et_3N. The product was used without further purification.

Peptide coupling:

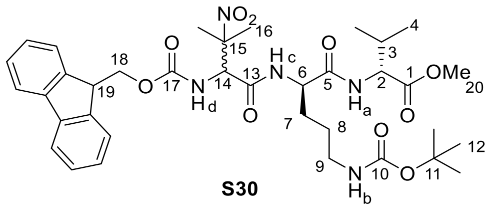

S30

To a solution of *N*-Fmoc-(*R,S*)-3-nitrovaline (**1**, 1.33 g, 3.45 mmol, 1.30 equiv.), EDC·HCl (763 mg, 3.98 mmol, 1.50 equiv.), and HOAt (542 mg, 3.98 mmol, 1.50 equiv.) in anhydrous CH_2Cl_2/DMF (36.0 mL, 5:1), $NaHCO_3$ (1.11 g, 13.3 mmol, 5.00 equiv.) was added at 0 °C. Then, amine **S29** dissolved in CH_2Cl_2 (5.00 mL) was added over a period of 10 min. The reaction was stirred for 16 h while it was slowly warmed to rt. Subsequently, it was filtered, the solvent was removed under reduced pressure, and the residue was purified by column chromatography (CH_2Cl_2/MeOH, 100:0 -> 99:1). The crude product still contained residual DMF and was therefore coevaporated with toluene (3x). Product **S30** (1.41 g, 1.98 mmol, 75% yield) was obtained as a yellowish foam.

*Compound **S30** was obtained as a mixture of two diastereoisomers with a ratio of about 1:1. The mixture was not separated. Therefore, the total sum of all signals is reported below:* **^{1}H-NMR** ($CDCl_3$, 400 MHz): *δ* [ppm] 7.79-7.72 (m, 2H, Ar-*H*, Fmoc), 7.61-7.55 (m, 2H, Ar-*H*, Fmoc), 7.43-7.36 (m, 2.5 H, Ar-*H*, Fmoc, NH_c), 7.34-7.27 (m, 2H, Ar-*H*, Fmoc), 7.22 (bs, 0.5H, NH_c), 6.89-6.70 (m, 1H, NH_a), 6.20 (bs, 0.5H, NH_d), 6.08 (bs, 0.5H, NH_d), 4.99-4.84 (m, 1H, *H*-14), 4.77 (bs, 1H, NH_b), 4.63-4.42 (m, 3H, *H*-2, *H*-6, *H*-18a), 4.42-4.30 (m, 1H, *H*-18b), 4.25-4.15 (m, 1H, *H*-19), 3.70 (s, 1.5H, *H*-20), 3.69 (s, 1.5H, *H*-20), 3.29-3.16 (m, 1H, *H*-9a), 3.12-3.01 (m, 1H, *H*-9b), 2.21-2.10 (m, 1H, *H*-3), 1.93-1.77 (m, 1H, *H*-7a), 1.73 (s, 1.5H, *H*-16a), 1.71 (s, 1.5H, *H*-16a), 1.69-1.53 (m, 4H, *H*-7b, *H*-16b), 1.53-

1.45 (m, 2H, *H*-8), 1.42 (s, 4.5H, *H*-12), 1.41 (s, 4.5H, *H*-12), 0.94-0.86 (m, 6H, *H*-4); **^{13}C-NMR** ($CDCl_3$, 100 MHz): *δ* [ppm] {172.1, 172.1} (q, *C*-1), 171.2 (q, *C*-5 or *C*-13), {167.9, 167.8} (q, *C*-5 or *C*-13), 156.8 (q, *C*-10 or *C*-17), 156.4 (q, *C*-10 or *C*-17), {143.7, 143.6, 143.5, 143.5} (q, Ar-*C*, Fmoc), 141.3 (q, Ar-*C*, Fmoc), {127.8, 127.8} (t, Ar-*C*, Fmoc), {127.2, 127.1} (t, Ar-*C*, Fmoc), {125.1, 125.0} (t, Ar-*C*, Fmoc), {120.1, 120.0} (t, Ar-*C*, Fmoc), {89.0, 88.8} (q, *C*-15), 79.6 (q, *C*-11), 67.5 (s, *C*-18), {59.9, 59.9} (t, *C*-14), {57.5, 57.5} (t, *C*-2), 52.7 (t, *C*-6), 52.1 (p, *C*-20), {47.1, 47.1} (t, *C*-19), 39.2 (s, *C*-9), 30.8 (t, *C*-3), 29.3 (s, *C*-7), 28.4 (p, *C*-12), 26.5 (s, *C*-8), {24.5, 24.3, 23.7, 23.4} (p, *C*-16), {19.0, 17.9} (p, *C*-4); **HRMS** (ESI): *m/z* calculated for $C_{36}H_{49}N_5O_{10}Na$ [M + Na$^+$]: 734.3377, found: 734.3372.

Teoc-L-Val-OH (S31)

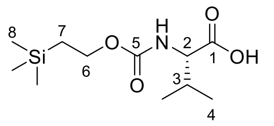

S31

To a solution of L-valine (1.49 g, 12.7 mmol, 1.00 equiv.) in H_2O (12.7 mL) Et_3N (1.92 g, 2.60 mL, 19.0 mmol, 1.50 equiv.) was added. TeocOSu (3.61 g, 13.9 mmol, 1.10 equiv.) was dissolved in 1.4 dioxane (12.7 mL) and added to the reaction mixture. The reaction was stirred for 24 h at rt. The reaction was then terminated by adding 1M $KHSO_4$ solution and EtOAc, the phases were separated, and the aqueous phase was extracted with EtOAc (3x). The combined organic phases were washed with H_2O (3x), dried over $MgSO_4$, filtered, and the solvent was removed under reduced pressure. Product **S31** (3.32 g, 12.7 mmol, quant.) was obtained as a colorless oil and used directly without further purification.

$[\alpha]_D^{26.1}$: - 7.5° (*c* 1.98; MeOH); **^{1}H-NMR** ($CDCl_3$, 400 MHz): *δ* [ppm] 5.10 (d, *J* = 8.2 Hz, 1H, N*H*), 4.32 (dd, *J* = 9.1, 4.3 Hz, 1H, *H*-2), 4.19-4.15 (m, 2H, *H*-6), 2.27-2.15 (m, 1H, *H*-3), 1.02-0.93 (m, 8H, *H*-4 u. *H*-7), 0.03 (s, 9H, *H*-8).

(*N*-Teoc-L-Valyl)-(*R,S*)-3-nitrovalyl-(*Nδ*-Boc-D-ornityl)-D-valinemethylester (S33)

Fmoc-*Deprotection:*

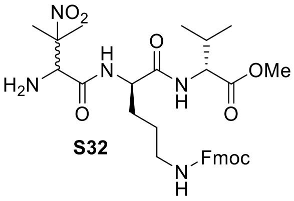

To a solution of carbamate **S30** (5.50 g, 7.73 mmol, 1.00 equiv.) in DMF (80 mL) dimethylamine (40% in H_2O, 9.78 mL, 77.3 mmol, 10.0 equiv.) was added. After 4 h (LCMS control), first the excess dimethylamine (0.5 mbar, rt, cold trap) and then the solvent (40°C, 9 mbar) were removed under reduced pressure. The residue was used without further purification.

Peptide coupling:

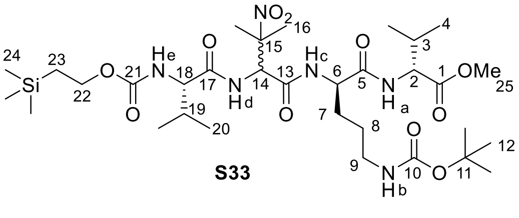

To a solution of EDC·HCl (1.85 g, 9.66 mmol, 1.25 equiv.) and HOAt (1.31 g, 9.66 mmol, 1.25 equiv.) in anhydrous CH_2Cl_2 (75.0 mL) Teoc-L-valine (**S31**, 2.22 g, 8.50 mmol, 1.10 equiv.) dissolved in anhydrous CH_2Cl_2 (5.00 mL) and DMF (10.0 mL) was added. The mixture was cooled to 0 °C and $NaHCO_3$ (3.25 g, 38.7 mmol, 5.00 equiv.) and amine **S33** (dissolved in 5.00 mL DMF) were added. The reaction was stirred for 16 h while the mixture was warmed to rt. The mixture was then filtered, the solvent was removed under reduced pressure, and the residue was purified by column chromatography (CH_2Cl_2/MeOH, 100:0 -> 99:1 -> 98.5:1.5 -> 98:2 -> 97.5:2.5). Product **S33** (4.33 g, 5.91 mmol, 76% yield, *d.r.* = 1:1) was obtained as a yellow foam.

Compound ***S33*** *was obtained as a mixture of two diastereoisomers with a ratio of about 1:1. The mixture was not separated. Therefore, the total sum of all signals is given below:*

^{1}H-NMR (DMSO-d$_6$, 400 MHz): δ [ppm] 8.41 (d, J = 10.2 Hz, 0.5H, NH_d), 8.33 (d, J = 8.3 Hz, 0.5H, NH_c), 8.32 (d, J = 6.7 Hz, 0.5H, NH_c), 8.17 (d, J = 8.2 Hz, 0.5 Hz, NH_a), 8.10 (d, J = 9.7 Hz, 0.5H, NH_d), 8.08 (d, J = 7.8 Hz, 0.5H, NH_a), 7.14 (d, J = 8.5 Hz, 0.5H, NH_e), 7.08 (d, J = 9.2 Hz, 0.5H, NH_e), 6.79-6.74 (m, 1H, NH_b), 5.28 (d, J = 9.8 Hz, 0.5H, *H*-14), 5.24 (d, J = 9.9 Hz, 0.5H, *H*-14), 4.36-4.27 (m, 0.5H, *H*-6), 4.27-4.21 (m, 0.5H, *H*-6), 4.18 (dd, J = 8.4, 6.4 Hz, 0.5H, *H*-2), 4.15 (dd, J = 8.1, 6.5 Hz, 0.5H, *H*-2), 4.12-3.98 (m, 2H, *H*-22), 3.95 (t, J = 7.5 Hz, 0.5H, *H*-18), 3.88 (t, J 8.1 Hz, 0.5H, *H*-18), 3.61 (s, 1.5H, *H*-25), 3.61 (s, 1.5H, *H*-25), 2.96-2.84 (m, 2H, *H*-9), 2.09-1.96 (m, 1H, *H*-3), 1.96-1.80 (m, 1H, *H*-19), 1.68-1.27 (m, 19H, *H*-7 u. *H*-8 u. *H*-12 u. *H*-16), 0.91 (t, J = 8.3 Hz, 2H, *H*-23), 0.88-0.76 (m, 12H, *H*-4 u. *H*-20), 0.01 (s, 4.5H, *H*-24), 0.00 (s, 4.5H, *H*-24); **^{13}C-NMR** (DMSO-d$_6$, 100 MHz): δ [ppm] {172.0, 171.8, 171.8, 171.7, 171.6, 171.5} (q, *C*-1 u. *C*-5 u. *C*-17), {167.2, 166.8} (q, *C*-13), {156.7, 156.3} (q, *C*-21), 155.6 (q, *C*-10), 88.8 (q, *C*-15), 77.4 (q, *C*-11), {62.0, 61.9} (s, *C*-22), {60.2, 60.0} (t, *C*-18), {57.3, 57.2, 57.1, 57.0} (t, *C*-2 u. *C*-14), {52.8, 52.3} (t, *C*-6), {51.7, 51.7} (p, *C*-25), {30.1, 30.1, 30.0, 29.9} (t, *C*-3 u. *C*-19), {29.4, 28.8} (s, *C*-7), 28.3 (3C, p, *C*-12), {26.1, 26.0} (s, *C*-8), {23.6, 23.1, 22.3, 21.9} (p, *C*-16), {19.2, 19.1, 18.9, 18.2, 18.1, 18.0} (p, *C*-4 u. *C*-20), {17.4, 17.4} (s, *C*-23), {-1.4, 1.5} (p, *C*-24); **HRMS** (ESI): *m/z* calculated for $C_{32}H_{60}N_6O_{11}SiNa$ [M + Na$^+$]: 755.3987, found: 755.3986.

Methyl ((2*R*)-2-(2-((*S*)-2-amino-3-methylbutanamido)-3-methyl-3-nitrobutanamido)-5-((*tert*-butoxycarbonyl)amino)pentanoyl)-D-valinate (fragment B-L,D)

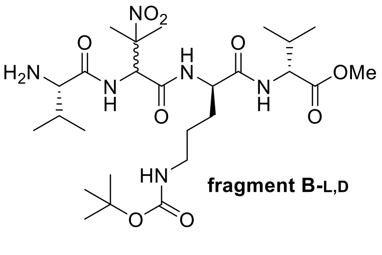

To a solution of Teoc-protected fragment B (**S33**, 519 mg, 708 μmol, 1.00 equiv.) in THF (14.2 mL), TBAF (1M in THF, 7.1 mL, 0.1M) was added and the reaction mixture was stirred for 16 h at ambient temperature. Then the reaction was terminated with MeOH (1.00 mL) and the solvent was removed under reduced pressure. Purification by flash column chromatography (RP- Büchi; solvent A: water + 0.1% FA, solvent B: MeCN + 0.1% FA; 4 g WP C18 column; flow rate: 20.0 ml/min; 30 sec/fr; gradient: (*t* [min]/solvent B [%]): 0/0; 25/95; 30/95; t_R = 9.5-17.0 min) afforded fragment B-L,D (416.2 mg, 707 μmol, quant.) as

a colorless foam that was used in the next step without further purification and characterization.

Fragment AB-L,D

((2*R*)-2-(2-((2*S*)-2-((3*S*)-3-(4-(allyloxy)phenyl)-3-((2*R*)-3-methyl-2-((2*R*)-3-methyl-2-((2*S*)-2-((2*S*)-*N*-methyl-2-(3-methyl-2-(3-methylbutanamido)- 3-nitrobutanamido) propan-amido)-propanamido)butanamido)butan-amido)propanamido)-3-methylbutanamido)-3-methyl-3-nitrobutanamido)-5-((*tert*-butoxycarbonyl)amino)pentanoyl)-D-valine (S34)

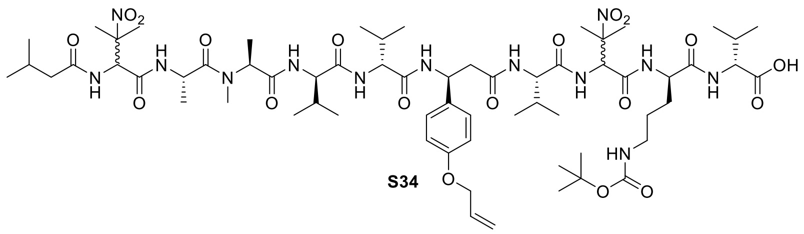

To a solution of fragment A (116 mg, 144 *µ*mol, 1.00 equiv.) and fragment B-L,D (156 mg, 265 *µ*mol, 1.80 equiv.) in two separate batches in DMF (1.60 mL) at 0 °C, HOAt (43.1 mg, 317 *µ*mol, 2.20 equiv.), PyAOP (225 mg, 432 *µ*mol, 3.00 equiv.) and DIPEA (125 µL, 720 *µ*mol, 5.00 equiv.) were added and the reaction mixture was stirred for 16 h at ambient temperature. The reaction was terminated with MeOH (1.00 mL), the batches were united and the solvent was removed under reduced pressure. Purification by flash column chromatography (RP-BÜCHI; solvent A: water + 0.1% FA, solvent B: MeCN + 0.1% FA, 40 g WP C18 column, flow rate: 30.0 mL/min, 30 sec/fr., gradient: (*t* [min]/solvent B [%]): 2/0; 23/100; 30/100; t_R = 17 – 28 min) afforded peptide **S34** (183 mg, 133 *µ*mol, 46% yield) as a colorless foam

Due to the number of diastereomers caused by the two nitro groups, peptide **S34** was used in the next step without further characterization:

((*R*)-2-(2-((*S*)-2-((*S*)-3-(4-(allyloxy)phenyl)-3-((*R*)-3-methyl-2-((*R*)-3-methyl-2-((*S*)-2-((*S*)-*N*-methyl-2-(3-methyl-2-(3-methylbutanamido)but-2-enamido)-propan-amido) propanamido)butanamido)butanamido)propan-amido)-3-methylbutan-amido)-3-methylbut-2-enamido)-5-((*tert*-butoxycarbonyl)amino)pentanoyl)-D-valine (fragment AB-L,D)

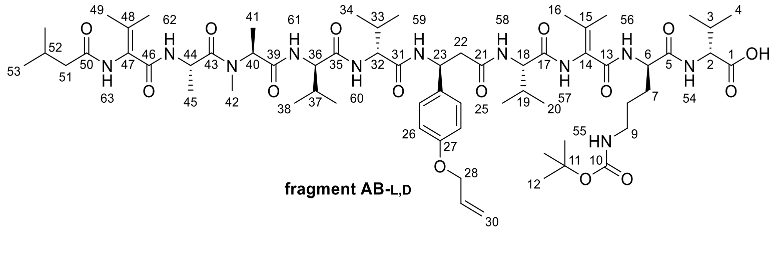

fragment AB-L,D

To a solution of peptide **S34** (182 mg, 132 *μ*mol, 1.00 equiv.) in THF (1.32 mL), LiOH (1M in H_2O, 55.6 mg, 1.32 mmol, 10.0 equiv.) was added dropwise at 0 °C and the reaction mixture was stirred at ambient temperature. After 17 h, the reaction was terminated with phosphate buffer solution (pH 7) and adjusted to pH 7. The solvent was removed under reduced pressure. Purification by flash column chromatography (RP- BÜCHI; solvent A: water + 0.1% FA, solvent B: MeCN + 0.1% FA, 24 g WP C18 column, flow rate: 20.0 mL/min, 30 sec/fr., gradient: (*t* [min]/solvent B [%]): 0/0; 3/0; 26/100; 30/100; t_R = 12.5 – 28.5 min) afforded fragment AB-L,D (127 mg, 100 *μ*mol, 76% yield, *d.r.* = 8.7:1.3) as a colorless foam.

$[\alpha]_D^{23.6}$: - 22.5° (*c* 0.4, DMSO-d6); **^{1}H-NMR** (DMSO-d6, 600 MHz): *δ* [ppm] 8.99 – 8.92 (m, 2H, *H*-57, *H*-63), 8.21 (s, 1H, N*H*), 7.78 – 7.51 (m, 6H, N*H*), 7.16 (d, *J* = 8.7 Hz, 2H, *H*-25), 6.79 (d, *J* = 8.8 Hz, 2H, *H*-26), 6.71 (t, *J* = 5.6 Hz, 1H, *H*-55), 6.03 – 5.98 (m, 1H, *H*-29), 5.37 – 5.34 (m, 1H, *H*-30), 5.24 – 5.22 (m, 1H, *H*-30), 5.13 – 5.11 (m, 1H, *H*-23), 5.02 – 5.01 (q, *J* = 7.1 Hz, 1H, *H*-40), 4.71 – 4.68 (m, 1H, *H*-44), 4.51 – 4.50 (m, 2H, *H*-28), 4.16 – 4.09 (m, 4H, *H*-2, *H-6*, *H*-18, *H*-32, *H*-36), 2.89 – 2.88 (m, 2H, *H*-9), 2.85 (s, 3H, *H*-42), 2.63 (m, 1H, *H*-22), 2.61 (m, 1H, *H*-19/*H*-33/*H*-37), 2.52 – 2.51 (m, 1H, *H*-22), 2.49 – 2.47 (m, 2H, *H*-19/*H*-33/*H*-37), 2.06 – 1.59 (m, 18H, *H*-3, *H*-7, *H*-16, *H*-49, *H*-51, *H*-52), 1.55 – 1.51 (m, 1H, *H*-7), 1.48 – 1.43 (m, 2H, *H*-8), 1.36 (s, 9H, *H*-12), 1.25 – 1.17 (m, 6H, *H*-41, *H*-45), 0.89 – 0.74 (m, 30H, *H*-4, *H*-20, *H*-34, *H*-38, *H*-53); **^{13}C-NMR** (DMSO-d6, 150 MHz): *δ* [ppm] 172.1 (q, C=O), 172.0 (q, C=O), 171.5 (q, C=O), 171.4 (q, C=O), 170.9 (q, C=O), 170.8 (q, C=O), 170.6 (q, C=O), 169.9 (q, C=O), 164.9 (q, C=O), 164.5 (q, C=O), 163.2 (q, C=O), 156.8 (q, *C*-27), 155.5 (q, *C*-10), 134.6 (q, 2x *C*-14, *C*-47), 134.4 (q, *C*-24), 133.8 (t, *C*-29), 127.2 (t, 2x *C*-25), 125.5 (q, *C*-15/*C*-48), 124.9 (q, *C*-15/*C*-48), 117.2 (s, *C*-30),

114.1 (t, 2x *C*-26), 77.3 (q, *C*-11), 68.1 (s, *C*-28), 58.3 (t, *C*-2/*C*-18/*C*-32/*C*-36), 58.0 (t, *C*-2/*C*-18/*C*-32/*C*-36), 57.8 (t, *C*-2/*C*-18/*C*-32/*C*-36), 57.4 (t, *C*-2/*C*-18/*C*-32/*C*-36), 52.3 (t, *C*-6), 51.6 (t, *C*-40), 49.3 (t, *C*-23), 45.0 (t, *C*44), 44.2 (s, *C*-51), 41.8 (s, *C*-22), 41.3 (s, *C*-9), 30.4 (p, *C*-42), 30.3 (s/t, *C*-3/*C*-7/*C*-19/*C*-33/*C*-37), 30.0 (s/t, *C*-3/*C*-7/*C*-19/*C*-33/*C*-37), 29.5 (s/t, *C*-3/*C*-7/*C*-19/*C*-33/*C*-37), 28.9 (s/t, *C*-3/*C*-7/*C*-19/*C*-33/*C*-37), 28.6 (s/t, *C*-3/*C*-7/*C*-19/ *C*-33/*C*-37), 28.3 (s, 3x *C*-12), 25.5 (s/t, *C*-8/*C*-52), 25.5 (s/t, *C*-8/*C*-52), 22.3 (p, 2x *C*-53), 20.7 (p, *C*-16/*C*-49), 20.7 (p, *C*-16/*C*-49), 20.3 (p, *C*-16/*C*-49), 20.1 (p, *C*-16/*C*-49), 19.3 (p, *C*-4/*C*-20/*C*-34/*C*-38), 19.3 (p, *C*-4/*C*-20/*C*-34/*C*-38), 19.2 (p, *C*-4/*C*-20/*C*-34/*C*-38), 19.1 (p, *C*-4/*C*-20/*C*-34/*C*-38), 18.2 (p, *C*-4/*C*-20/*C*-34/*C*-38), 18.0 (p, *C*-4/*C*-20/*C*-34/*C*-38), 17.8 (p, *C*-4/*C*-20/*C*-34/*C*-38), 17.7 (p, *C*-4/*C*-20/*C*-34/*C*-38), 17.4 (p, *C*-41/45), 14.4 (p, *C*-41/45); **HRMS** (ESI) *m/z* calculated for $C_{64}H_{104}N_{11}O_{15}$ $[M+H]^+$ 1266.7715; found 1266.7713.

Fragment ABCD-L,D

Boc-Protected Fragment ABCD-L,D (S35)

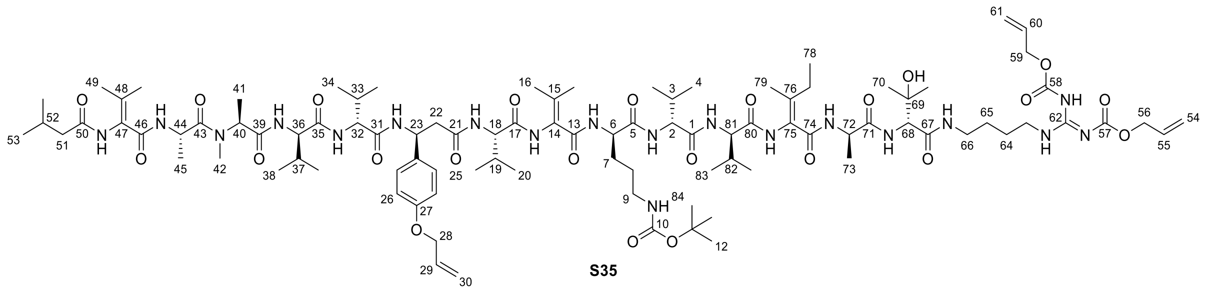

S35

To a solution of fragment AB-L,D (248 mg, 196 *µ*mol, 1.00 equiv.) and fragment CD (304 mg, 437 *µ*mol, 2.23 equiv.) in DMF (2.00 mL) at 0 °C, HOAt (66.7 mg, 490 *µ*mol, 2.50 equiv.), HATU (186 mg, 490 *µ*mol, 2.50 equiv.) and DIPEA (170 µL, 980 *µ*mol, 5.00 equiv.) were added and the reaction mixture was stirred for 17 h at ambient temperature. The reaction was terminated with MeOH (1.00 mL) and the solvent was removed under reduced pressure. Purification by flash column chromatography (RP-BÜCHI; solvent A: water + 0.1% FA, solvent B: MeCN + 0.1% FA, 40 g WP C18 column, flow rate: 30.0 mL/min, 30 sec/fr., gradient: (*t* [min]/solvent B [%]): 0/0; 2/0; 23/100; 30/100; t_R = 20.5 – 60.0 min) afforded Boc-protected fragment ABCD-L,D **S35** (220 mg, 113 *µ*mol, 58% yield) as a colorless foam.

[*α*]$_D^{24.5}$: - 72.0° (*c* 0.1, DMSO-d$_6$); **^{1}H-NMR** (DMSO-d$_6$, 500 MHz): *δ* [ppm] 9.72 – 8.63 (m, 1H, N*H*), 8.99 (m, 1H, N*H*), 8.91 – 8.88 (m, 1H, N*H*), 8.35 (t, *J* = 5.5 Hz, 1H, N*H*), 8.27 – 8.26 (m, 1H, N*H*), 8.15 – 8.14 (m, 1H, N*H*), 7.86 – 7.47 (m, 10H, N*H*), 7.16 – 7.15 (d, *J* = 8.4 Hz, 2H, *H*-25), 6.82 – 6.80 (d, *J* = 8.7 Hz, 2H, *H*-26), 6.74 (t, *J* = 5.4 Hz, 1H, N*H*-84), 6.04 – 5.88 (m, 3H, *H*-29, *H*-55, *H*-60), 5.37 – 5.16 (m, 8H, *H*- 23, *H*-30, *H*-54, *H*-61, O*H*), 5.00 – 4.86 (m, 1H, *H*-40), 4.69 – 4.65 (m, 3H, *H*-44, *H*-56, *H*-59), 4.51 – 4.48 (m, 4H, *H*-28, *H*-56, *H*-59), 4.42 – 3.93 (m, 10H, *H*-2, *H*-6, *H*-18, *H*-32, *H*-36, *H*-63, *H*-68, *H*-72, *H*-81), 3.13 – 2.99 (m, 2H, *H*-66), 2.94 – 2.82 (m, 5H, *H*-9, *H*-42), 2.79 – 2.69 (m, 1H, *H*-22), 2.54 – 2.51 (m, 6H, *H*-3, *H*-19, *H*-22, *H*-33, *H*-37, *H*-82), 2.34 – 2.21 (m, 2H, *H*-77), 2.06 – 1.65 (m, 18H, *H*-16, *H*-49, *H*-51, *H*-52, *H*-79), 1.52 – 1.39 (m, 7H, *H*-7, *H*-8, *H*-64, *H*-65), 1.36 (s, 9H, *H*-12), 1.25 – 1.17 (m, 9H, *H*-41, *H*-45, *H*-73), 1.11 (m, 6H, *H*-70), 0.98 (t, *J* = 7.4 Hz, 3H, *H*-78), 0.93 – 0.72 (m, 36H, *H*-4, *H*-20, *H*-34, *H*-38, *H*-53, *H*-83); **^{13}C-NMR** (DMSO-d$_6$, 150 MHz): *δ* [ppm] 172.5 (q, C=O), 172.1 (q, C=O), 172.0 (q, C=O), 171.9 (q, C=O), 171.8 (q, C=O), 171.1 (q, C=O), 170.9 (q, C=O), 170.7 (q, C=O), 170.7 (q, C=O), 170.4 (q, C=O), 170.0 (q, C=O), 169.7 (q, C=O), 165.5 (q, C=O), 165. 2 (q, C=O), 164.5 (q, C=O), 162.9 (q, *C*-57/*C*-58), 156.9 (q, *C*-27), 155.5 (q, *C*-10), 155.0 (q, *C*-62), 152.5 (q, , *C*-57/*C*-58), 134.6 (3x q, *C*-14, *C*-47, *C*-75), 134.1 (q, *C*-24), 133.7 (t, *C*-29/*C*-55/*C*-60), 133.5 (t, *C*-29/*C*-55/ *C*-60), 131.8 (t, *C*-29/*C*-55/*C*-60), 127.2 (t, 2x *C*-25), 125.5 (q, *C*-15/*C*-48/*C*-76), 124.6 (q, *C*-15/ *C*-48/*C*-76), 123.8 (q, *C*-15/*C*-48/*C*-76), 118.8 (s, *C*-30/*C*-54/*C*-61), 117.3 (s, *C*-30/*C*-54/*C*-61), 117.2 (s, *C*-30/*C*-54/*C*-61), 114.2 (t, 2x *C*-26), 77.3 (q, *C*-11), 70.9 (q, *C*-69), 68.0 (s, *C*-28), 66.4 (s, *C*-56/*C*-59), 65.4 (s, *C*-56/*C*-59), 60.2 (t, *C*-2/*C*-18/*C*-32/*C*-36/*C*-68/*C*-81), 59.7 (t, *C*-2/*C*-18/ *C*-32/*C*-36/*C*-68/*C*-81), 59.0 (t, *C*-2/*C*-18/*C*-32/*C*-36/*C*-68/*C*-81), 58.9 (t, *C*-2/*C*-18/*C*-32/*C*-36/ *C*-68/*C*-81), 58.3 (t, *C*-2/*C*-18/*C*-32/*C*-36/*C*-68/*C*-81), 57.6 (t, *C*-2/*C*-18/*C*-32/*C*-36/*C*-68/*C*-81), 53.9 (t, *C*-6/*C*-23/*C*-40/*C*-44/*C*-72), 51.9 (t, *C*-6/*C*-23/*C*-40/*C*-44/*C*-72), 49.2 (t, *C*-6/*C*-23/*C*-40/ *C*-44/*C*-72), 48.5 (t, *C*-6/*C*-23/*C*-40/*C*-44/*C*-72), 45.1 (t, *C*-6/*C*-23/*C*-40/*C*-44/*C*-72), 44.2 (s, *C*-51), 42.2 (s, *C*-22), 40.1 (s, *C*-9 and *C*-63 beneath DMSO-signal), 38.1 (s, *C*-66), 31.2 (t, *C*-3/*C*-19/*C*-33/*C*-37/*C*-82), 30.5 (t, *C*-3/*C*-19/*C*-33/*C*-37/*C*-82), 30.2 (t, *C*-3/*C*-19/*C*-33/*C*-37/ *C*-82), 30.1 (t, *C*-3/*C*-19/*C*-33/*C*-37/*C*-82), 29.4 (t, *C*-3/*C*-19/*C*-33/*C*-37/*C*-82 and s, *C*-7), 29.3 (t, *C*-3/*C*-19/*C*-33/*C*-37/*C*-82 and s, *C*-7), 28.2 (s, 3x *C*-12), 27.1 (p, *C*-70), 26.3 (s, *C*-77), 26.2 (s, *C*-65), 26.0 (s, *C*-64), 25.8 (s, *C*-8), 25.5 (t, *C*-53), 22.3 (p, *C*-53), 22.0 (p, *C*-53), 21.0 (p, *C*-16/*C*-49), 20.6 (p, *C*-16/*C*-49), 20.3 (p, *C*-16/*C*-49), 20.0 (p, *C*-16/*C*-49), 19.2 (p, *C*-4/*C*-20/*C*-34/*C*-38/*C*-83), 19.1 (p, *C*-4/*C*-20/*C*-34/*C*-38/*C*-83), 19.1 (2x p, *C*-4/*C*-20/*C*-34/ *C*-38/*C*-83), 18.9 (p, *C*-4/*C*-20/*C*-34/*C*-38/*C*-83), 18.9 (p, *C*-4/*C*-20/*C*-34/*C*-38/*C*-83), 18.6 (p, *C*-4/*C*-20/*C*-34/*C*-38/*C*-8), 18.4 (p, *C*-4/*C*-20/*C*-34/*C*-38/*C*-83), 18.3 (p, *C*-4/*C*-20/*C*-34/ *C*-

38/*C*-83), 18.2 (p, *C*-4/*C*-20/*C*-34/*C*-38/*C*-83), 18.0 (p, *C*-41/*C*-45/*C*-73/*C*-79), 17.6 (p, *C*-41/ *C*-45/*C*-73/*C*-79), 17.3 (p, *C*-41/*C*-45/*C*-73/*C*-79), 14.3 (p, *C*-41/*C*-45/*C*-73/*C*-79), 12.6 (p, *C*-78); **HRMS** (ESI) *m/z* calculated for $C_{96}H_{156}N_{19}O_{23}$ $[M+H]^+$ 1943.1621; found 1943.1627.

Boc-Deprotected Fragment ABCD-L,D (S36)

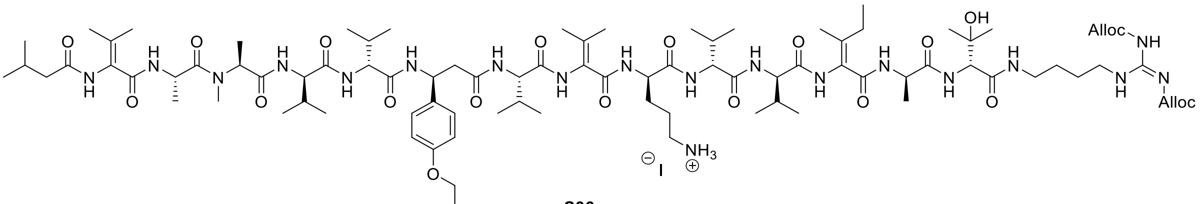

S36

To a solution of Boc-protected fragment ABCD-L,D (**S35**, 60.0 mg, 30.9 *μ*mol, 1.00 equiv.) in CH_2Cl_2 (500 *μ*L), TMSI (13.0 *μ*L, 92.6 *μ*mol, 3.00 equiv.) was added and the reaction mixture was stirred for 1 h at ambient temperature. The reaction was terminated with H_2O (500 *μ*L) and MeOH (500 *μ*L) and the solvent was removed under reduced pressure affording deprotected peptide **S36** as a yellow foam that was used in the next step without further purification.

Guanidinylated Fragment ABCD-L,D (S37)

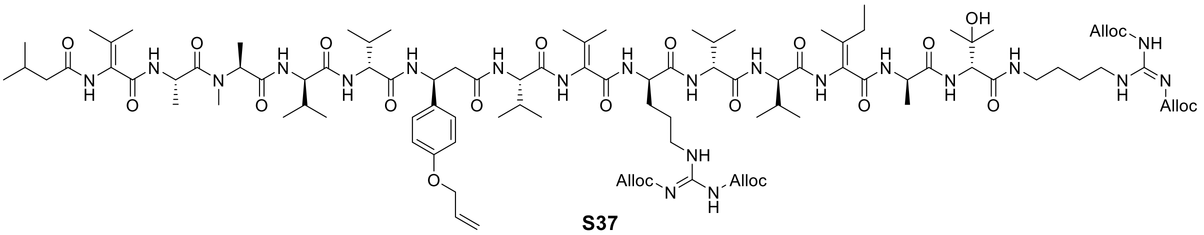

S37

To a solution of Boc-deprotected fragment ABCD-L,D (**S36**, 30.9 *μ*mol, 1.00 equiv.) and *N,N*'-bisalloc-*S*-methylisothiourea (**7**, 31.9 mg, 124 *μ*mol, 4.00 equiv.) in THF (800 *μ*L) at 0 °C, Et_3N (21.5 μL, 154.4 *μ*mol, 5.00 equiv.) was added dropwise and the reaction mixture was stirred for 17 h at ambient temperature. The reaction was terminated with H_2O (1.00 mL) and MeOH (1.00 mL) and the solvent was removed under reduced pressure. Purification by flash column chromatography (RP-BÜCHI; solvent A: water + 0.1% FA, solvent B: MeCN + 0.1% FA, 12 g WP C18 column, flow rate: 20.0 mL/min, 30 sec/fr., gradient: (*t* [min]/solvent B [%]): 0/0; 2/0; 23/100; 30/100; t_R = 20 – 60 min) afforded guanidinylated fragment ABCD-L,D (**S37**, 26.1 mg, 12.7 *μ*mol, 41% yield o2s) as a colorless foam that was used in the next step without further purification.

Myxovalargin-L,D

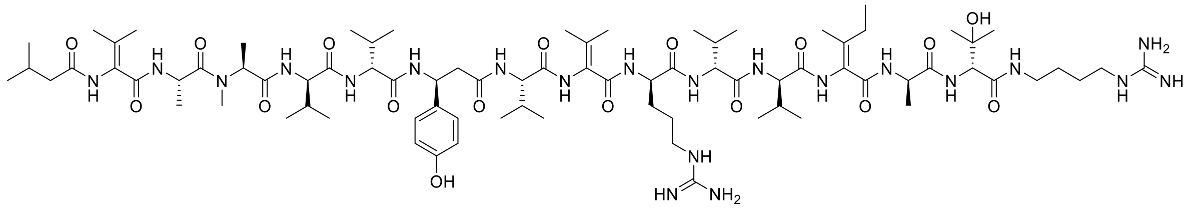

myxovalargin-L,D

To a solution of guanidinylated fragment ABCD-L,D (**S37**, 17.5 mg, 8.50 μmol, 1.00 equiv.) and phenylsilane (6.30 μL, 51.1 μmol, 6.00 equiv.) in CH_2Cl_2 (400 μL), $Pd(PPh_3)_4$ (2.00 mg, 1.70 μmol, 0.20 equiv.) was added and the reaction mixture was stirred for 2 h at ambient temperature under an Argon atmosphere. The reaction was terminated with H_2O (500 μL) and MeOH (500 μL) and the solvent was removed under reduced pressure. Purification by flash column chromatography (RP-BÜCHI; solvent A: water + 0.1% FA, solvent B: MeCN + 0.1% FA, 12 g WP C18 column, flow rate: 20.0 mL/min, 30 sec/fr., gradient: (*t* [min]/solvent B [%]): 0/0; 5/0; 27/100; 30/100, t_R = 12.5 – 30.5 min) afforded myxovalargin-L,D (8.70 mg, 5.20 μmol, 61% yield) as a colorless foam.

^{1}H-NMR (DMSO-d_6, 600 MHz): δ [ppm] 8.08 – 7.28 (m, 23H, N*H*, Tyr-O*H*), 7.22 – 7.18 (m, 2H, *H*-Ar), 6.65 – 6.62 (m, 2H, *H*-Ar), 5.72 – 5.61 (m, 1H, NC*H*), 5.41 – 5.35 (m, 1H, NC*H*), 5.29 – 5.10 (m, 2H, NC*H*), 5.06 – 4.63 (m, 1H, NC*H*), 4.52 – 4.36 (m, 1H, NC*H*), 4.28 – 3.90 (m, 5H, NC*H*), 3.51 – 3.50 (m, 10H, NC*H*$_3$, C*H*$_2$, HCC*H*), 3.14 – 3.06 (m, 2H, HCC*H*), 3.06 – 2.99 (m, 1H, HCC*H*), 2.54 – 2.51 (m, 3H, C*H*$_2$, HCC*H*), 2.28 – 2.17 (m, 1H, C*H*/C*H*$_2$), 2.02 – 1.97 (m, 12H, C*H*$_2$/C*H*$_3$), 1.80 – 1.59 (m, 9H, C*H*$_2$/C*H*$_3$), 1.48 – 1.41 (m, 4H, C*H*$_2$/C*H*$_3$), 1.29 – 1.27 (m, 11H, C*H*$_2$/C*H*$_3$), 1.15 – 1.09 (m, 6H, C*H*$_3$), 0.99 – 0.97 (m, 3H, H_2CC*H*$_3$), 0.89 – 0.75 (m, 36H, C*H*$_3$); **^{13}C-NMR** (DMSO-d_6, 150 MHz): δ [ppm] 174.7 (q, C=O), 174.3 (q, C=O), 174.0 (q, C=O), 173.9 (q, C=O), 173.4 (q, C=O), 173.3 (q, C=O), 173.2 (q, C=O), 172.7 (q, C=O), 172.0 (q, C=O), 170.9 (q, C=O), 170.1 (q, C=O), 169.9 (q, C=O), 169. 3 (q, C=O), 166.0 (q, C=O), 165.9 (q, C=O), 165.8 (q, C=O), 156.9 (q, OAr-*C*), 156.8 (q, *C*=N), 131.3 (q, *C*=C), 129.8 (q, *C*=C), 128.1 (q, *C*=C), 127.9 (q, *C*=C), 127.2 (q, *C*=C), 126.8 (q, *C*=C), 126.5 (q, *C*=C), 124.2 (q, *C*=C), 123.7 (q, *C*=C), 72.4 (q, HO*C*), 72.3 (t, N*C*H), 69.8 (t, N*C*H), 67.3 (t, N*C*H), 67.2 (t, N*C*H), 62.8 (t, N*C*H), 60.3 (t, N*C*H), 60.2 (t, N*C*H), 52.2 (t, N*C*H), 49.3 (t, N*C*H), 48.9 (t, N*C*H), 48.8 (t, N*C*H), 47.8 (t, N*C*H), 45.9 (s, *C*H$_2$), 41.4 (s, *C*H$_2$), 40.1 (s, *C*H$_2$ beneath DMSO-signal), 35.1 (s, *C*H$_2$), 31.3 (t, H_3C*C*H), 29.1 (t, H_3C*C*H), 29.0 (t, H_3C*C*H), 28.8 (t, H_3C*C*H), 28.7 (t/s, H_3C*C*H/*C*H$_2$), 28.7 (t/s, H_3C*C*H/*C*H$_2$), 28.7 (t/s, H_3C*C*H/*C*H$_2$), 28.6 (t/s, H_3C*C*H/*C*H$_2$), 28.6 (s, t/s, H_3C*C*H/*C*H$_2$),

27.5 (s/p, CH_2/CH_3), 27.2 (s/p, CH_2/CH_3), 26.6 (s/p, CH_2/CH_3), 26.6 (s/p, CH_2/CH_3), 25.1 (s/p, CH_2/CH_3), 25.1 (s/p, CH_2/CH_3), 24.5 (s/p, CH_2/CH_3), 23.6 (s/p, CH_2/CH_3), 22.3 (p, CH_3), 22.1 (p, CH_3), 22.1 (p, CH_3), 20.1 (p, CH_3), 20.0 (p, CH_3), 20.0 (p, CH_3), 19.9 (p, CH_3), 19.9 (p, CH_3), 19.9 (p, CH_3), 19.2 (p, CH_3), 19.2 (p, CH_3), 19.1 (p, CH_3), 15.9 (p, CH_3), 14.0 (p, H_2CCH_3); **HRMS** (ESI) *m/z* calculated for $C_{181}H_{137}N_{21}O_{17}$ $[M+2H/2]^+$ 839.0329; found 839.0332.

High performance liquid chromatography of myxovalargin (L/D-epimer): An Alliance 2695 HPLC-system by WATERS with a WATERS 996 diode array detector (λ = 200-350 nm) and a Nucleodur C18 HTec column (5 µm, 250 mm, Ø 8 mm) by MACHEREY-NAGEL were used. The eluant was 2-propanol / acetonitrile 3% in a pH 6.0 trimethyl formiate buffer (1:1:1.5).

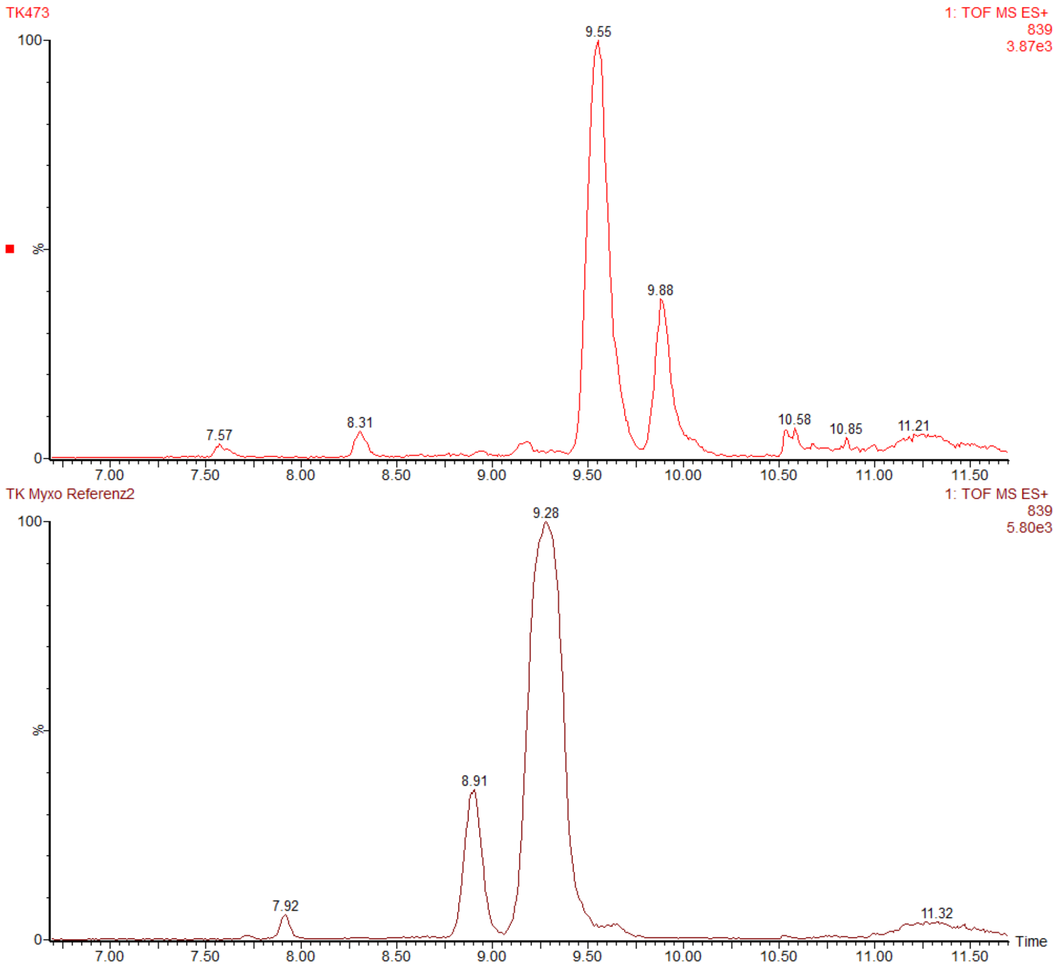

Figure S5-2: Top chromatogram: Synthetic Myxovalargin A / Myxovalargin-L,D epimer; Bottom chromatogram: Authentic sample of Myxovalargin A.

Note: The other peaks at R_t= 9.88 min (top) and R_t= 8.91 min (bottom) supposedly refer to the presence of epimers. In the synthetic sample (top), this isomer appeared first after the final synthetic steps, namely the removal of the protecting groups.

6. Spectra of synthesis products

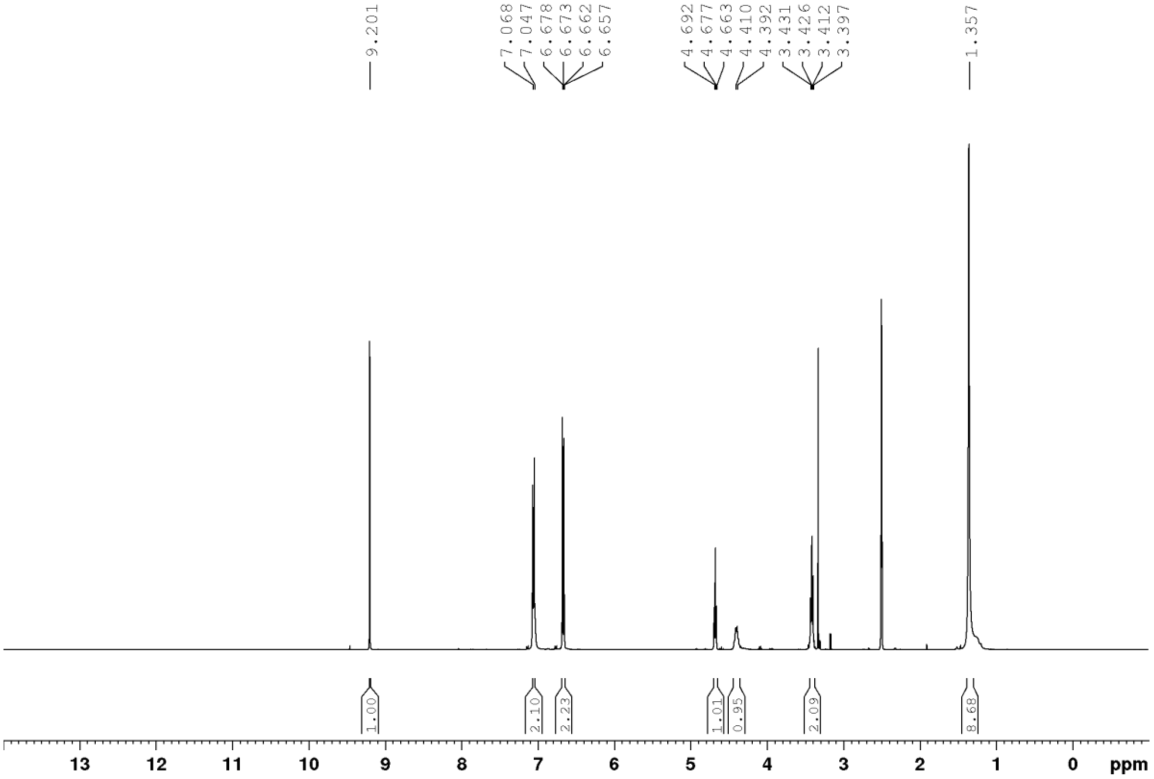

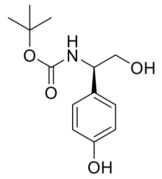

S1

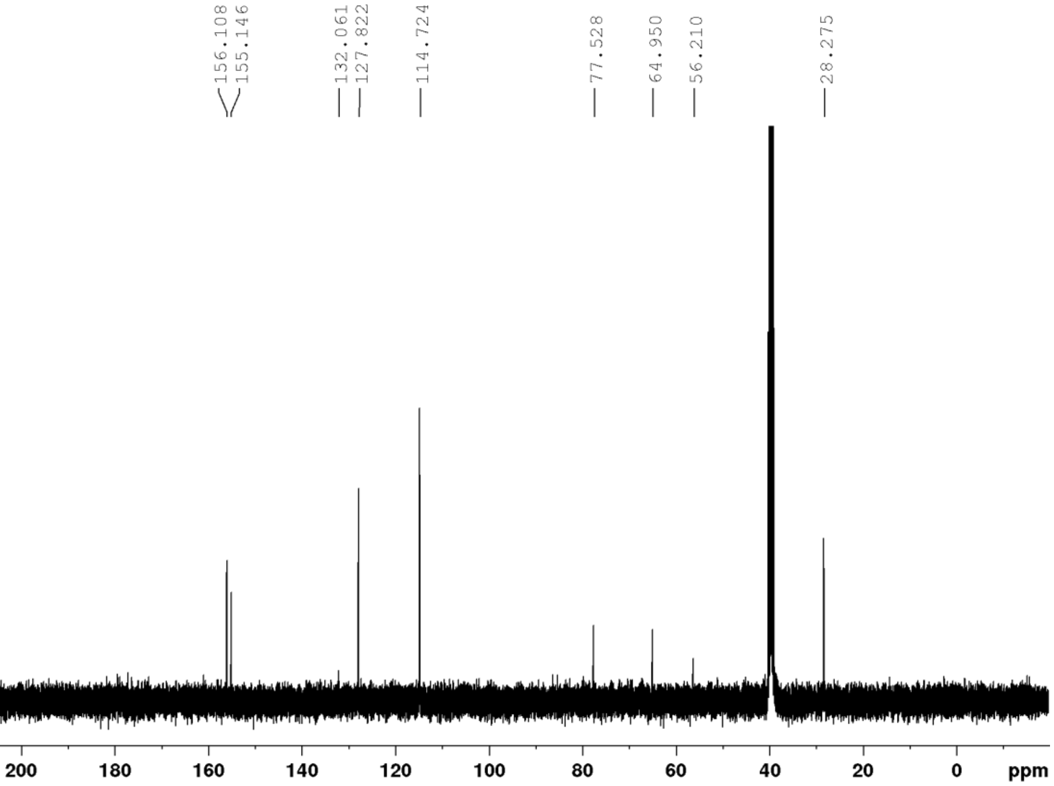

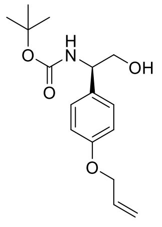
H
O
O
N
OH
O

S3

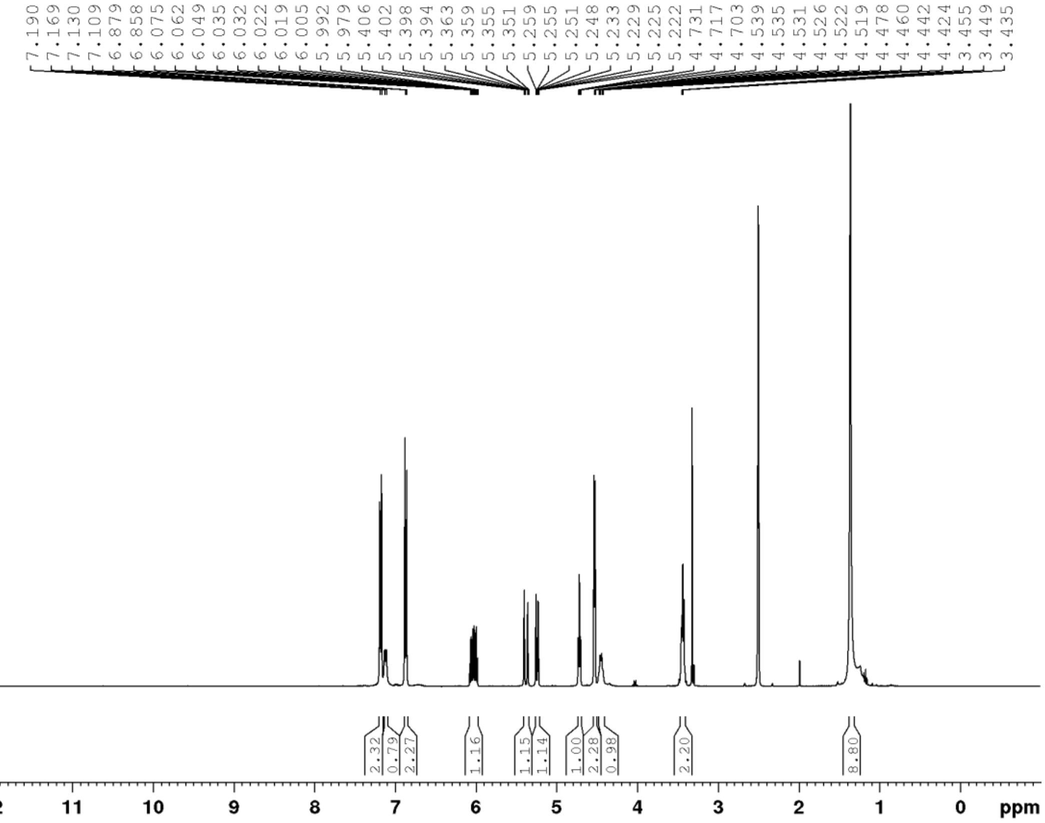
7.190
7.169
7.130
7.109
6.879
6.858
6.075
6.062
6.049
6.035
6.032
6.022
6.019
6.005
5.992
5.979
5.406
5.402
5.398
5.394
5.363
5.359
5.355
5.351
5.259
5.255
5.251
5.248
5.233
5.229
5.225
5.222
4.731
4.717
4.703
4.539
4.535
4.531
4.526
4.522
4.519
4.478
4.460
4.442
4.424
3.455
3.449
3.435
2.32
0.79
2.27
1.16
1.15
1.14
1.00
2.28
0.98
2.20
8.80
13
12
11
10
9
8
7
6
5
4
3
2
1
0
ppm

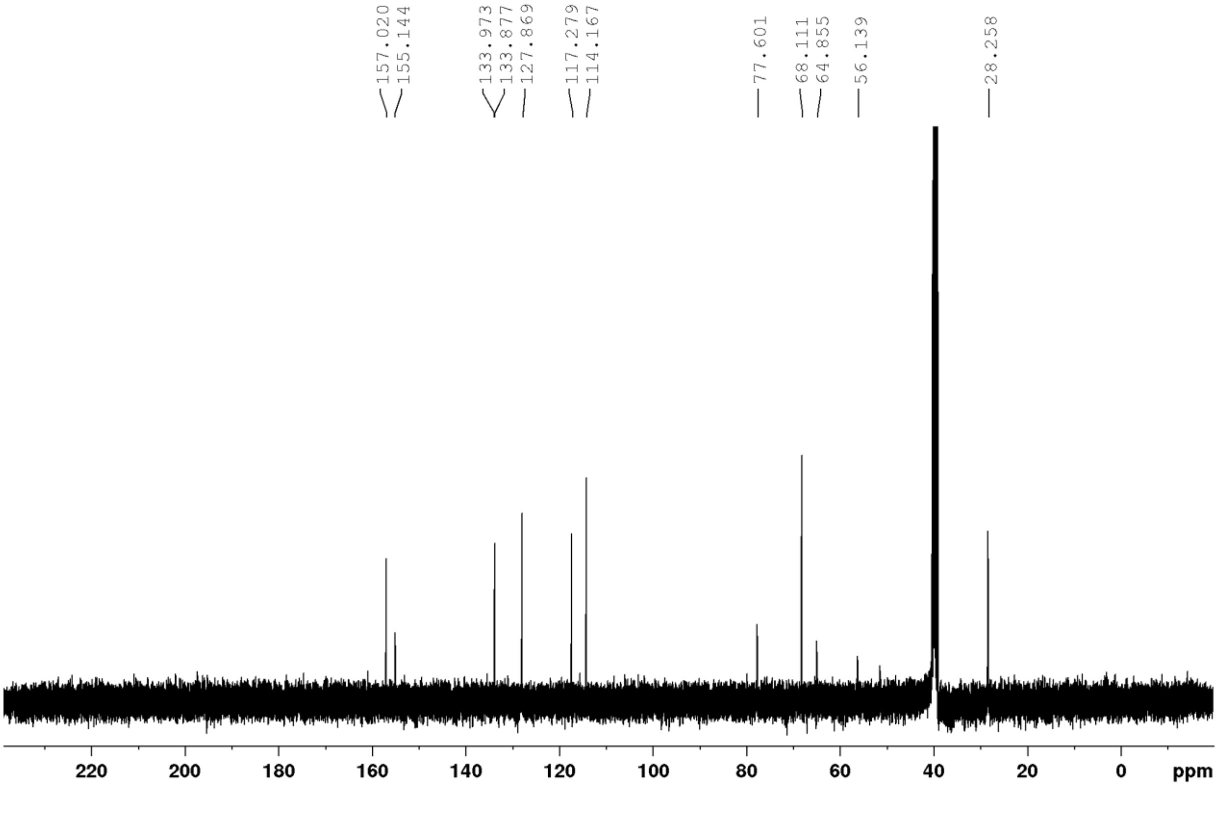
157.020
155.144
133.973
133.877
127.869
117.279
114.167
77.601
68.111
64.855
56.139
28.258
220
200
180
160
140
120
100
80
60
40
20
0
ppm

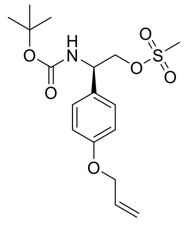

S4

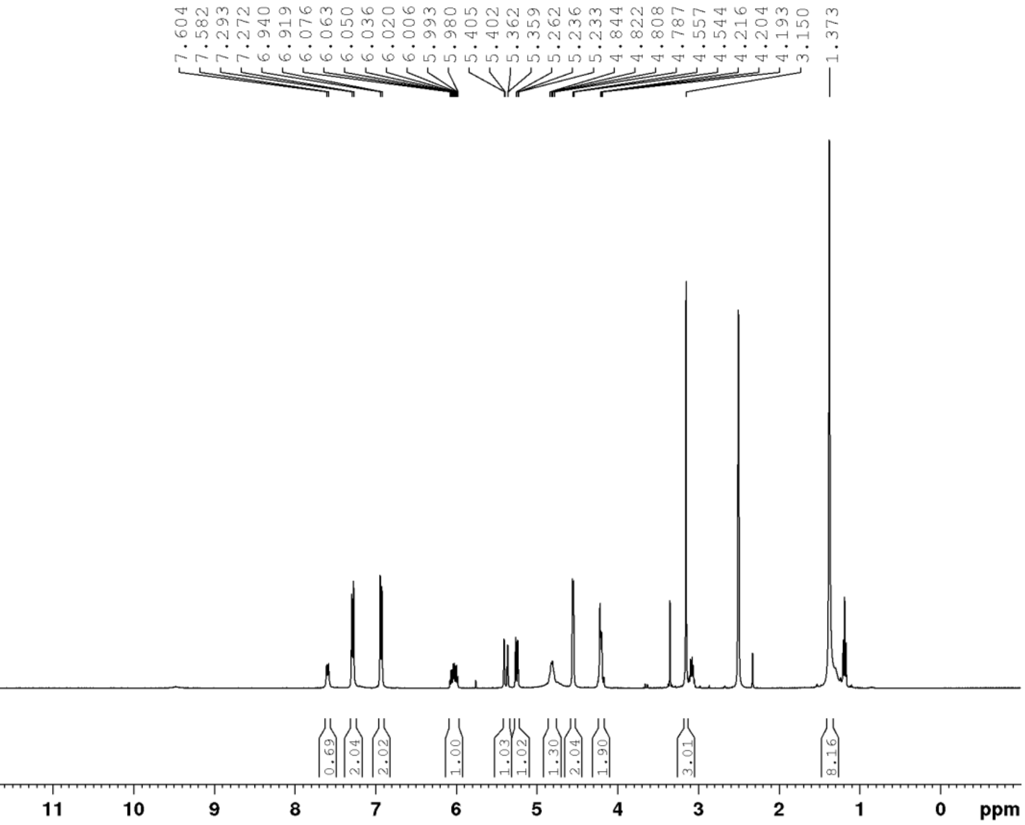
7.604
7.582
7.293
7.272
6.940
6.919
6.076
6.063
6.050
6.036
6.020
6.006
5.993
5.980
5.405
5.402
5.362
5.359
5.262
5.236
5.233
4.844
4.822
4.808
4.787
4.557
4.544
4.216
4.204
4.193
3.150
1.373
0.69
2.04
2.02
1.00
1.03
1.02
1.30
2.04
1.90
3.01
8.16
13 12 11 10 9 8 7 6 5 4 3 2 1 0 ppm

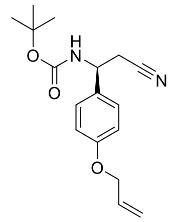
H
N
O
O
N
O

S5

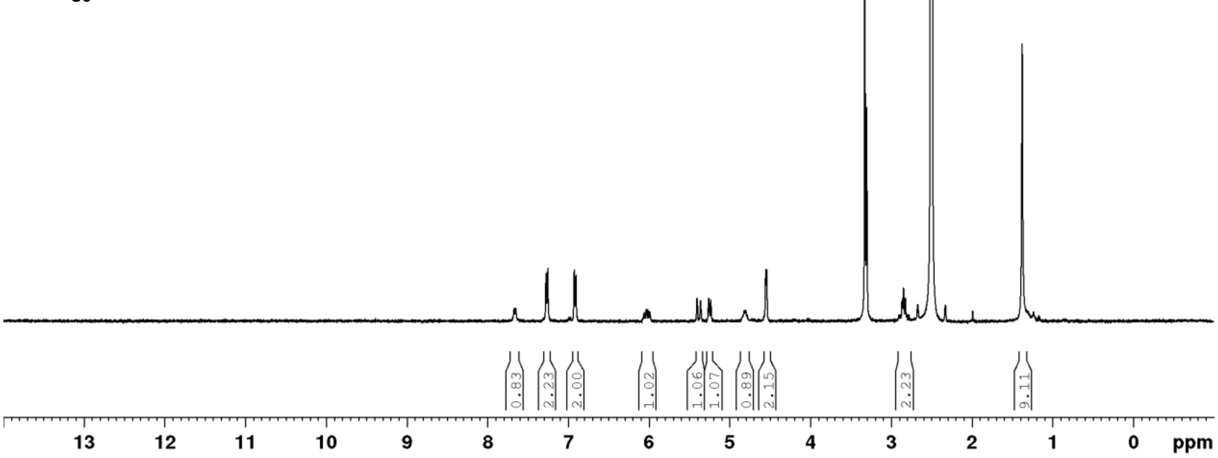
7.671
7.649
7.274
7.253
6.925
6.903
6.060
6.047
6.033
6.016
6.004
5.990
5.978
5.403
5.400
5.357
5.259
5.233
4.821
4.803
4.552
4.539
2.862
2.844
2.821
1.375
0.83
2.23
2.00
1.02
1.06
1.07
0.89
2.15
2.23
9.11
13
12
11
10
9
8
7
6
5
4
3
2
1
0
ppm

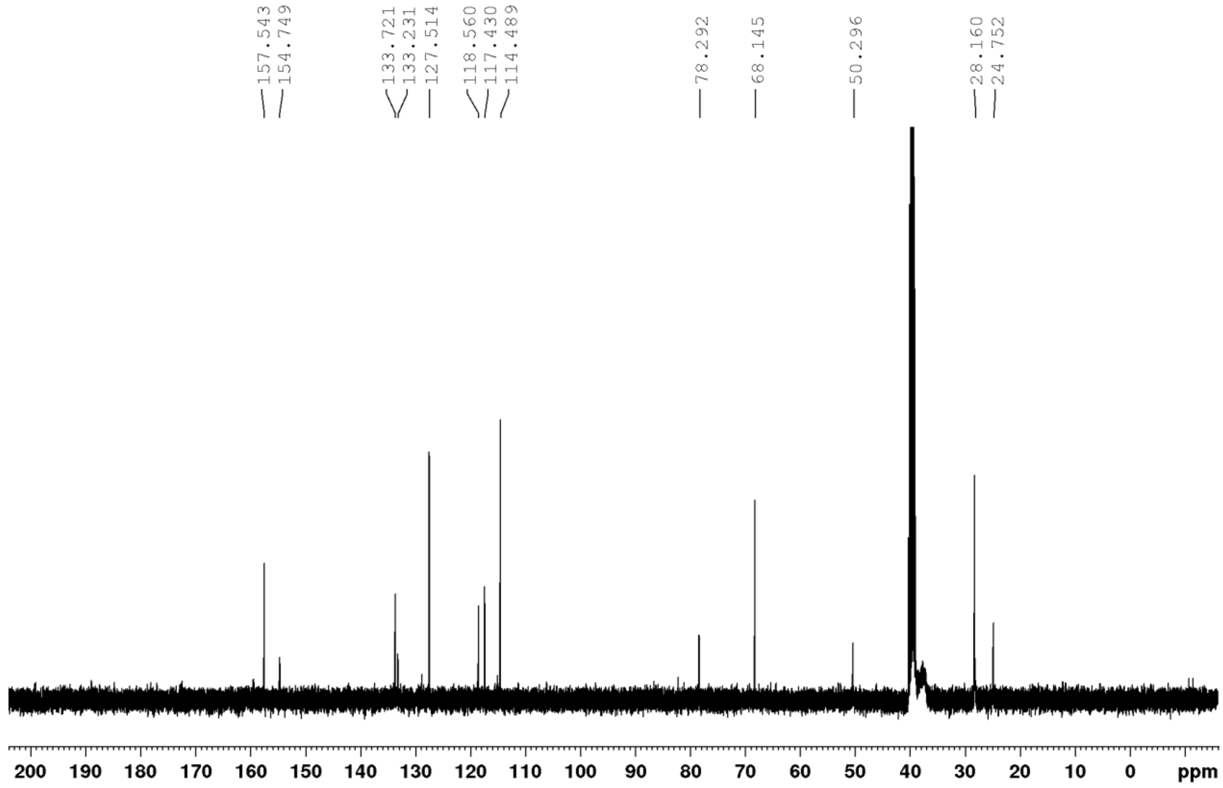
157.543
154.749
133.721
133.231
127.514
118.560
117.430
114.489
78.292
68.145
50.296
28.160
24.752
200
190
180
170
160
150
140
130
120
110
100
90
80
70
60
50
40
30
20
10
0
ppm

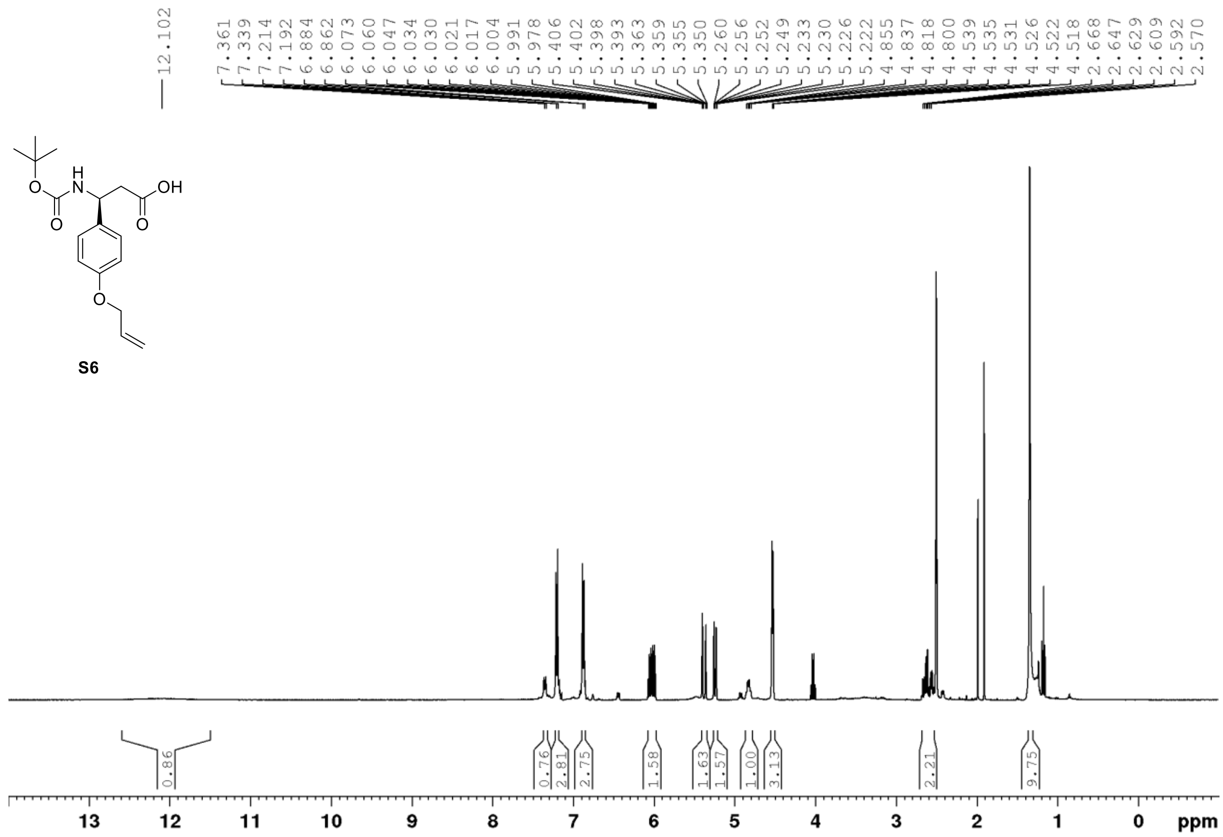
12.102
7.361
7.339
7.214
7.192
6.884
6.862
6.073
6.060
6.047
6.034
6.030
6.021
6.017
6.004
5.991
5.978
5.406
5.402
5.398
5.393
5.363
5.359
5.355
5.350
5.260
5.256
5.252
5.249
5.233
5.230
5.226
5.222
4.855
4.837
4.818
4.800
4.539
4.535
4.531
4.526
4.522
4.518
2.668
2.647
2.629
2.609
2.592
2.570
H
OH
O
O
O
O
S6
0.86
0.76
2.81
2.75
1.58
1.63
1.57
1.00
3.13
2.21
9.75
13 12 11 10 9 8 7 6 5 4 3 2 1 0 ppm

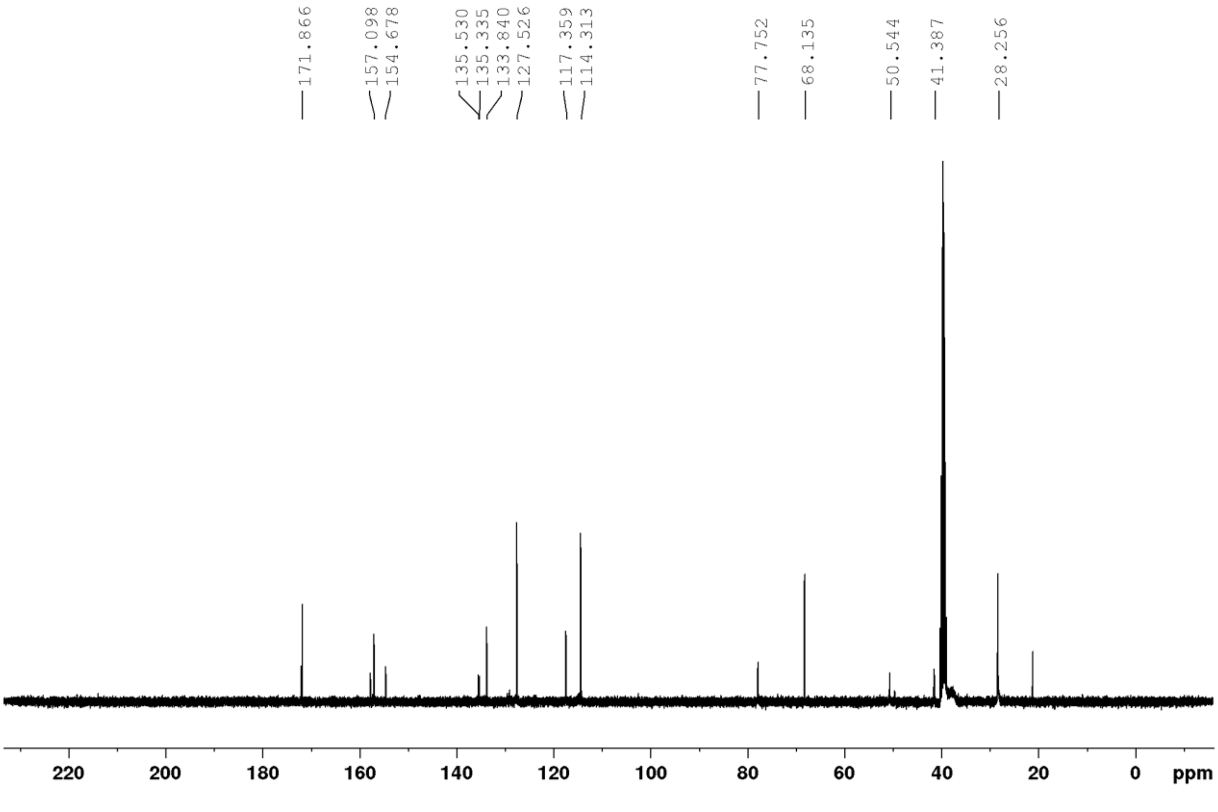
171.866
157.098
154.678
135.530
135.335
133.840
127.526
117.359
114.313
77.752
68.135
50.544
41.387
28.256
220 200 180 160 140 120 100 80 60 40 20 0 ppm

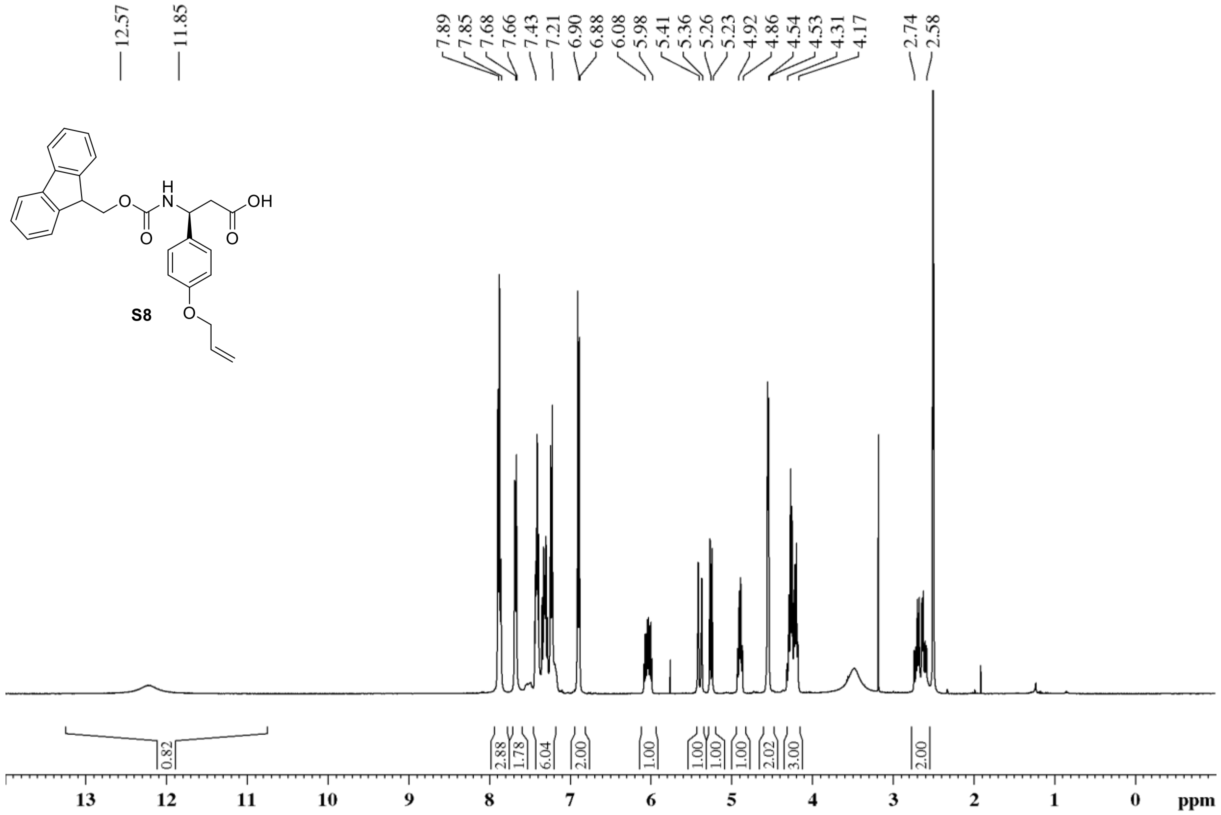
12.57
11.85
7.89
7.85
7.68
7.66
7.43
7.21
6.90
6.88
6.08
5.98
5.41
5.36
5.26
5.23
4.92
4.86
4.54
4.53
4.31
4.17
2.74
2.58
S8
OH
O
H
N
0.82
2.88
1.78
6.04
2.00
1.00
1.00
1.00
1.00
2.02
3.00
2.00
13 12 11 10 9 8 7 6 5 4 3 2 1 0 ppm

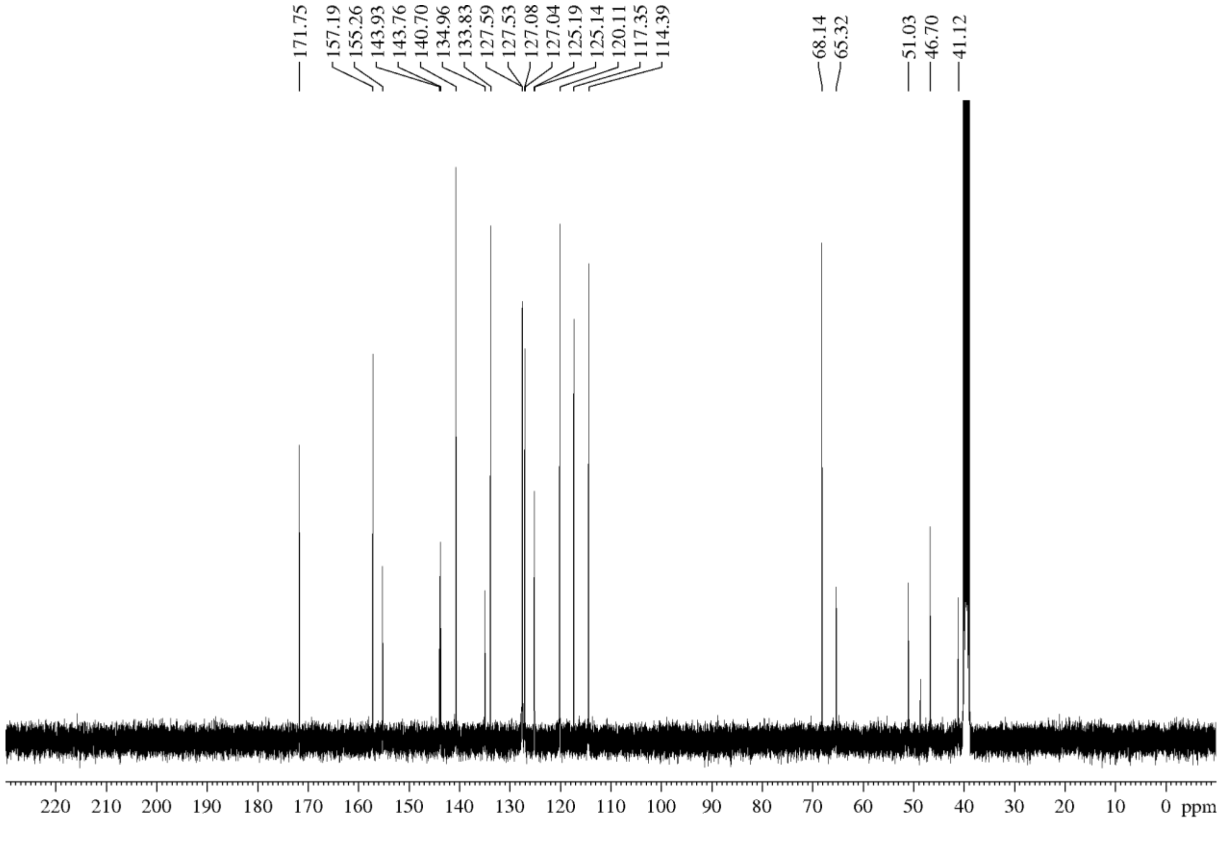
171.75
157.19
155.26
143.93
143.76
140.70
134.96
133.83
127.59
127.53
127.08
127.04
125.19
125.14
120.11
117.35
114.39
68.14
65.32
51.03
46.70
41.12
220 210 200 190 180 170 160 150 140 130 120 110 100 90 80 70 60 50 40 30 20 10 0 ppm

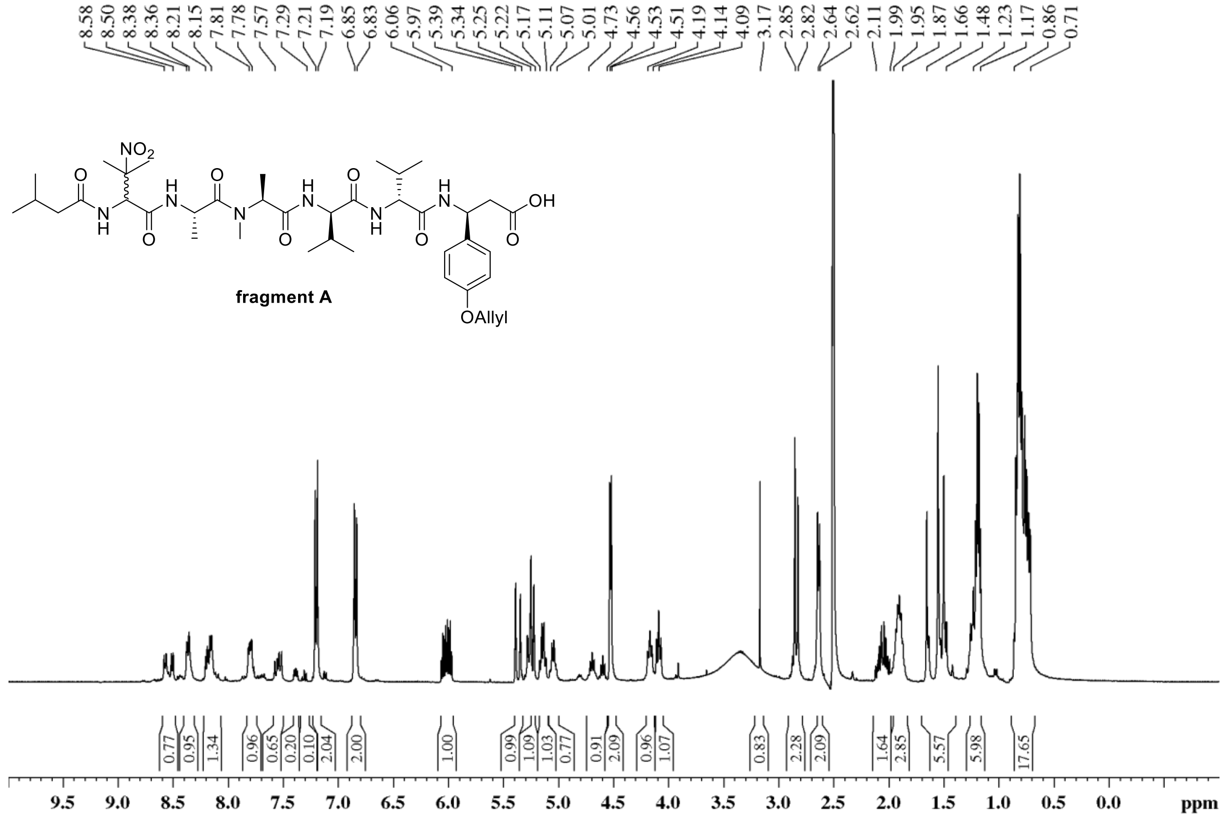
8.58 8.50 8.38 8.36 8.21 8.15 7.81 7.78 7.57 7.29 7.21 7.19 6.85 6.83 6.06 5.97 5.39 5.34 5.25 5.22 5.17 5.11 5.07 5.01 4.73 4.56 4.53 4.51 4.19 4.14 4.09 3.17 2.85 2.82 2.64 2.62 2.11 1.99 1.95 1.87 1.66 1.48 1.23 1.17 0.86 0.71
NO_2
fragment A
OAllyl
0.77 0.95 1.34 0.96 0.65 0.20 0.10 2.04 2.00 1.00 0.99 1.09 1.03 0.77 0.91 2.09 0.96 1.07 0.83 2.28 2.09 1.64 2.85 5.57 5.98 17.65
9.5 9.0 8.5 8.0 7.5 7.0 6.5 6.0 5.5 5.0 4.5 4.0 3.5 3.0 2.5 2.0 1.5 1.0 0.5 0.0 ppm

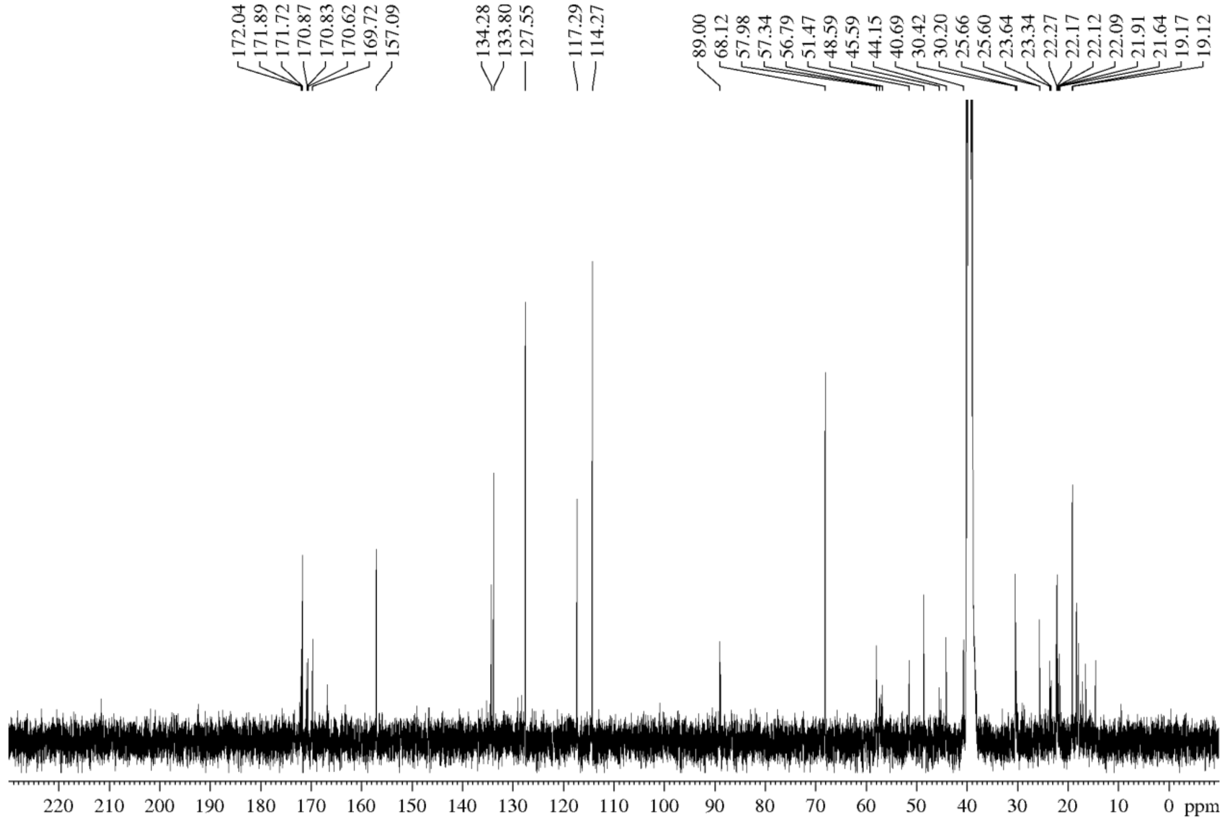
172.04 171.89 171.72 170.87 170.83 170.62 169.72 157.09 134.28 133.80 127.55 117.29 114.27 89.00 68.12 57.98 57.34 56.79 51.47 48.59 45.59 44.15 40.69 30.42 30.20 25.66 25.60 23.64 23.34 22.27 22.17 22.12 22.09 21.91 21.64 19.17 19.12
220 210 200 190 180 170 160 150 140 130 120 110 100 90 80 70 60 50 40 30 20 10 0 ppm

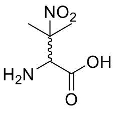
NO2
H2N
OH
O

S10

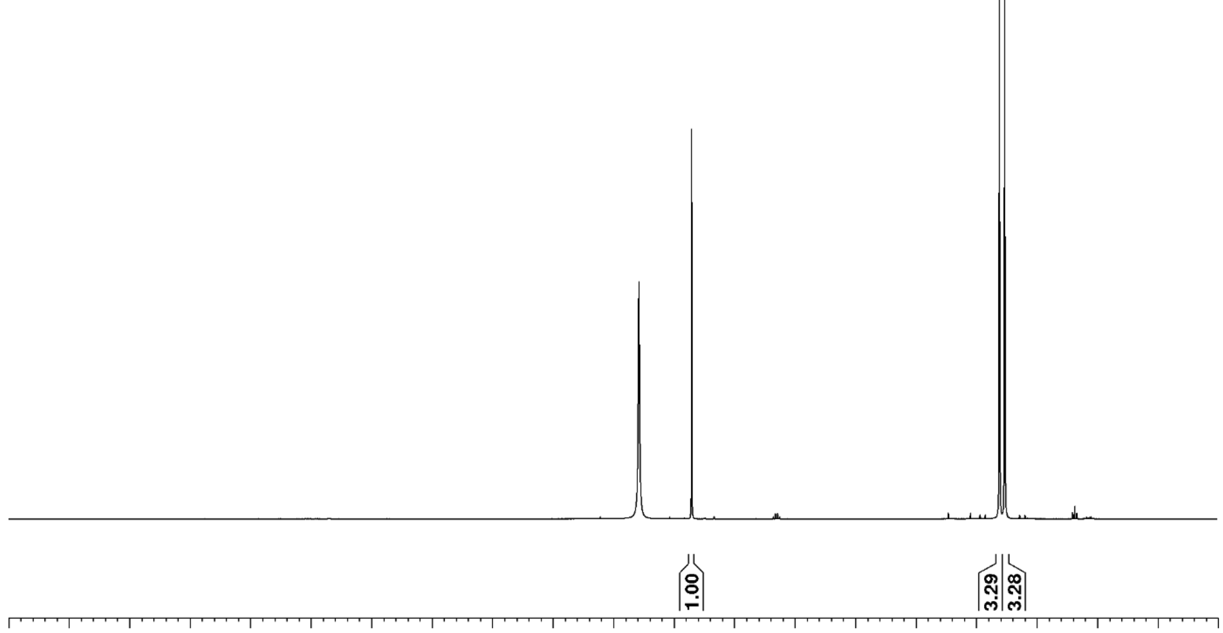
1.00
3.29
3.28
9.5 9.0 8.5 8.0 7.5 7.0 6.5 6.0 5.5 5.0 4.5 4.0 3.5 3.0 2.5 2.0 1.5 1.0 ppm

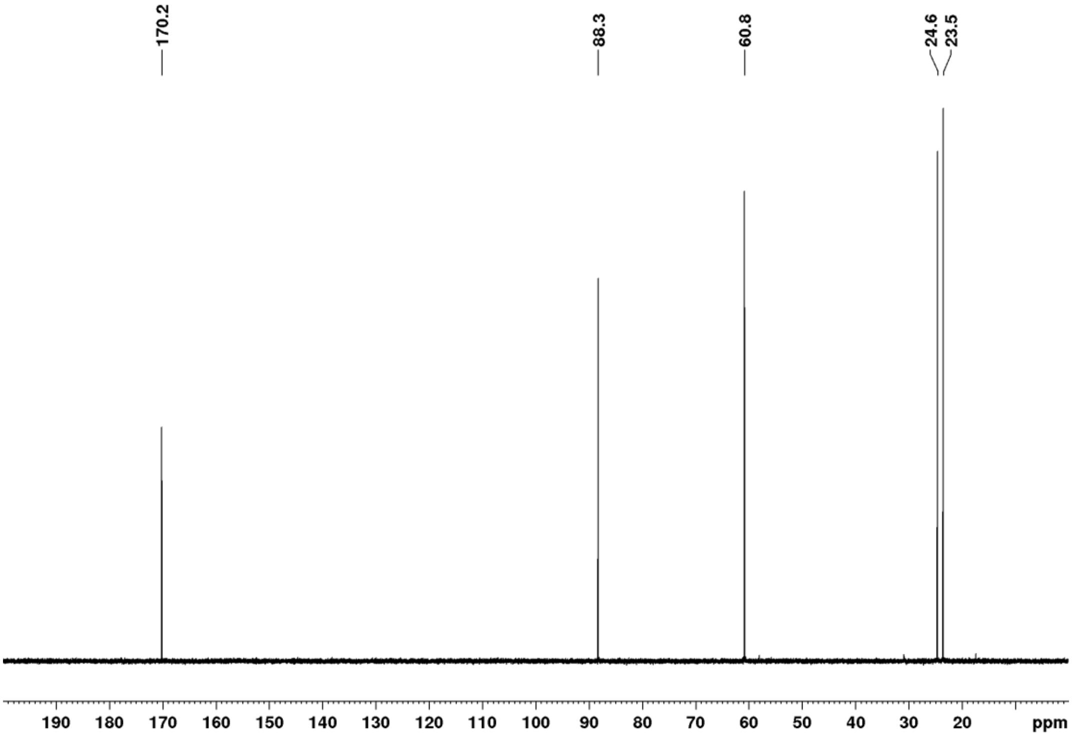
170.2
88.3
60.8
24.6
23.5
190 180 170 160 150 140 130 120 110 100 90 80 70 60 50 40 30 20 ppm

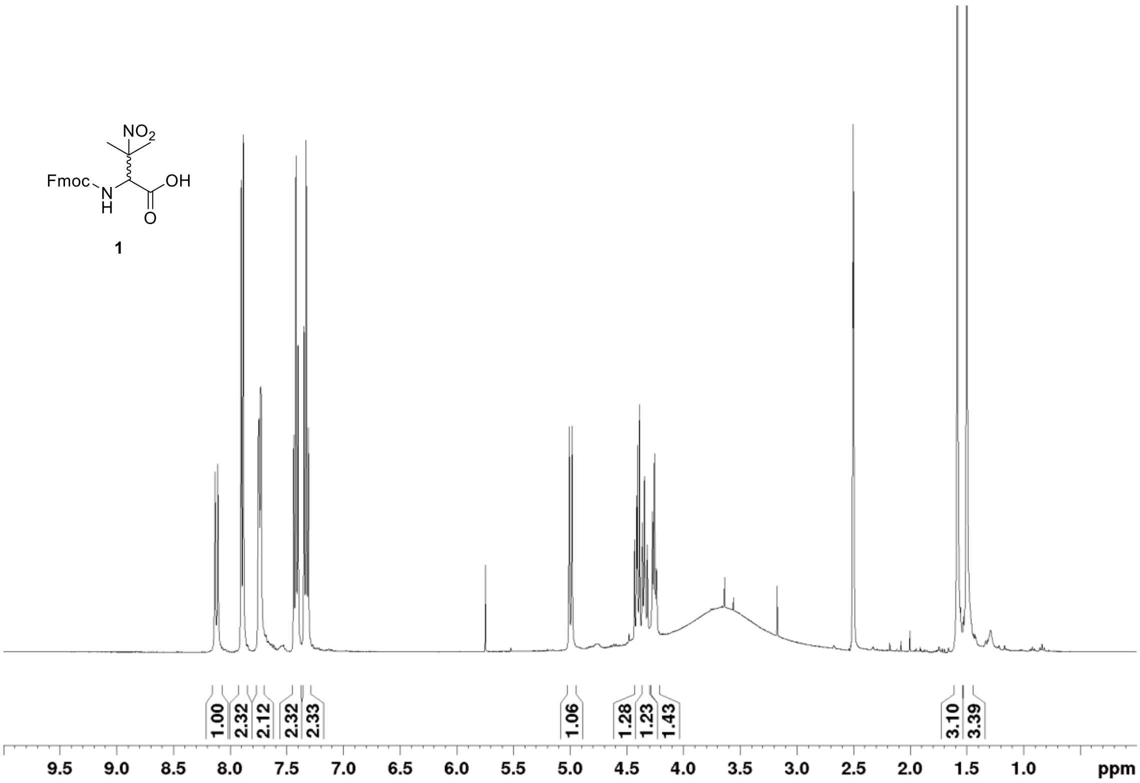
NO2
Fmoc
N
H
OH
O
1
1.00
2.32
2.12
2.32
2.33
1.06
1.28
1.23
1.43
3.10
3.39
9.5 9.0 8.5 8.0 7.5 7.0 6.5 6.0 5.5 5.0 4.5 4.0 3.5 3.0 2.5 2.0 1.5 1.0 ppm

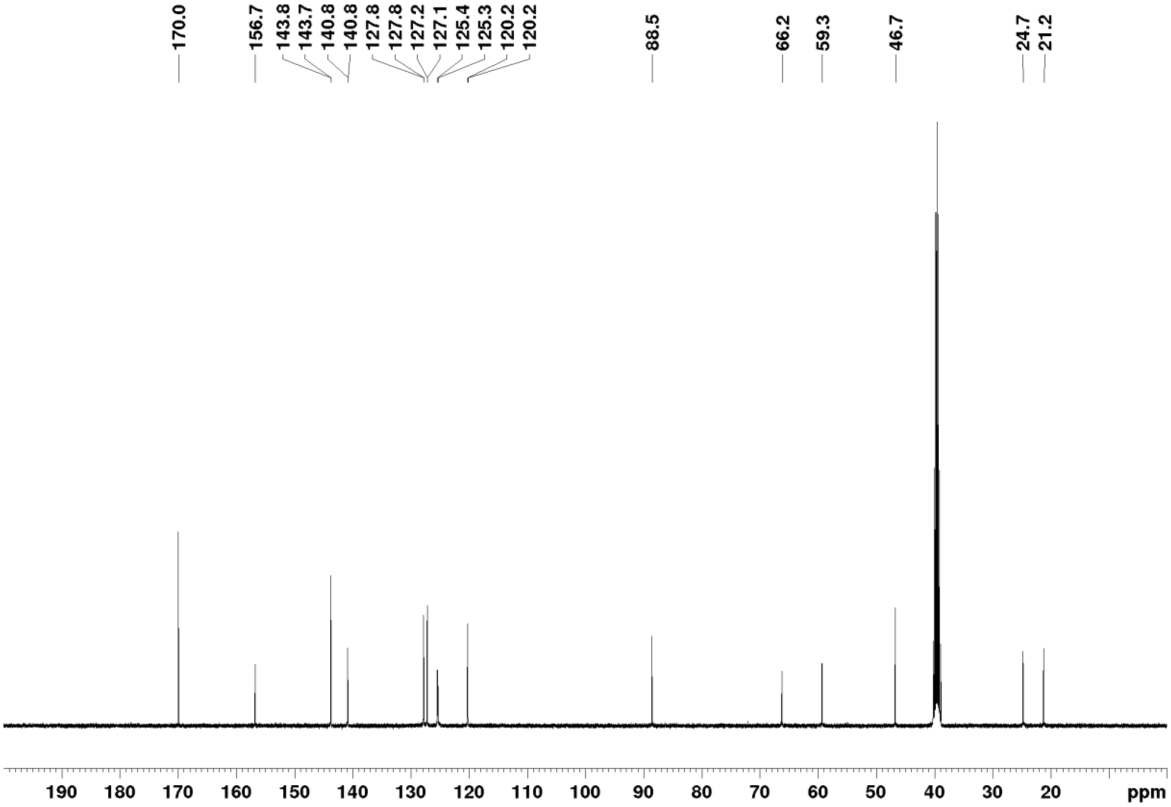
170.0
156.7
143.8
143.7
140.8
140.8
127.8
127.8
127.2
127.1
125.4
125.3
120.2
120.2
88.5
66.2
59.3
46.7
24.7
21.2
190 180 170 160 150 140 130 120 110 100 90 80 70 60 50 40 30 20 ppm

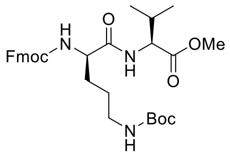
Fmoc
H
N
O
N
H
OMe
O
N
H
Boc

S11

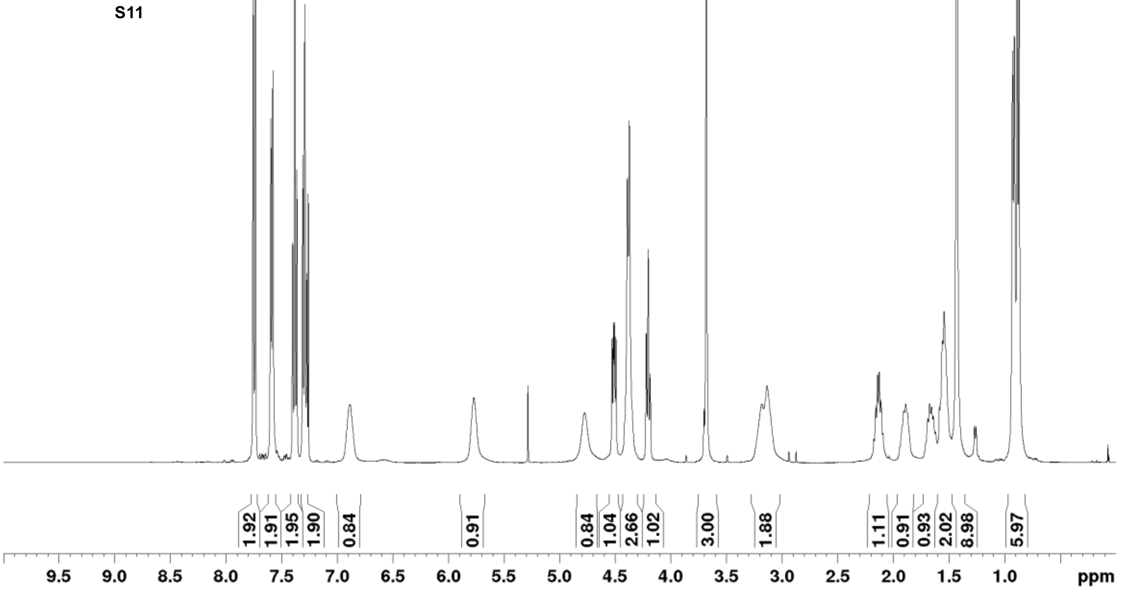
1.92
1.91
1.95
1.90
0.84
0.91
0.84
1.04
2.66
1.02
3.00
1.88
1.11
0.91
0.93
2.02
8.98
5.97
9.5 9.0 8.5 8.0 7.5 7.0 6.5 6.0 5.5 5.0 4.5 4.0 3.5 3.0 2.5 2.0 1.5 1.0 ppm

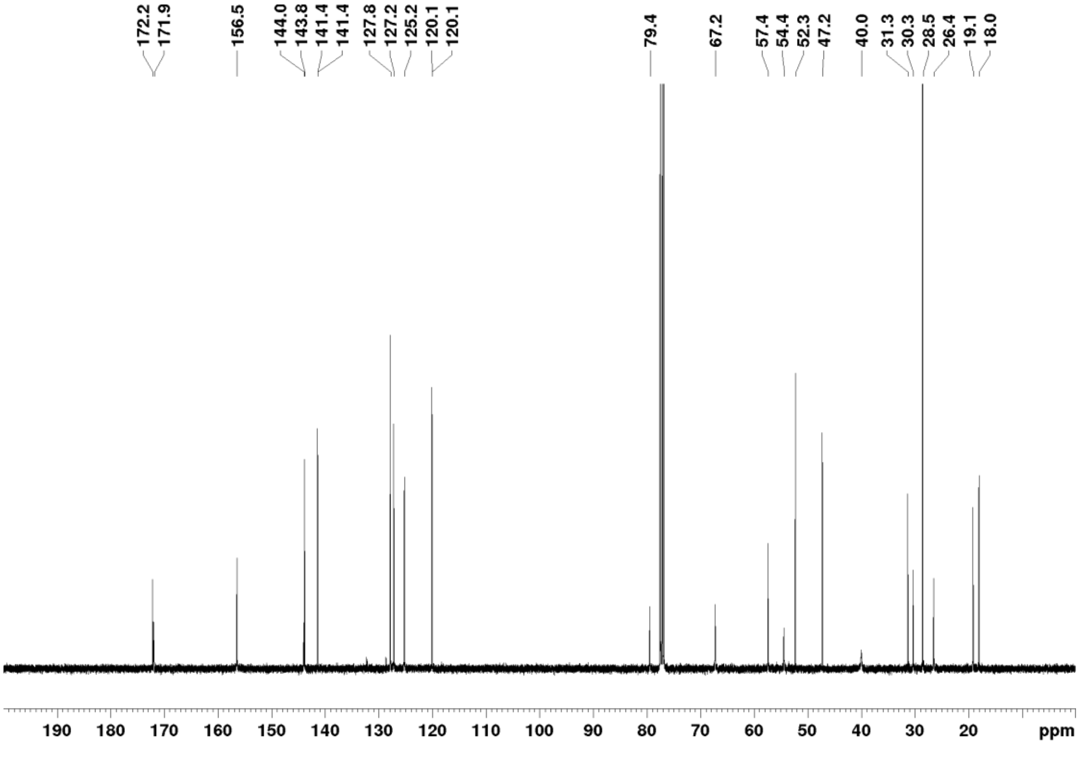
172.2
171.9
156.5
144.0
143.8
141.4
141.4
127.8
127.2
125.2
120.1
120.1
79.4
67.2
57.4
54.4
52.3
47.2
40.0
31.3
30.3
28.5
26.4
19.1
18.0
190 180 170 160 150 140 130 120 110 100 90 80 70 60 50 40 30 20 ppm

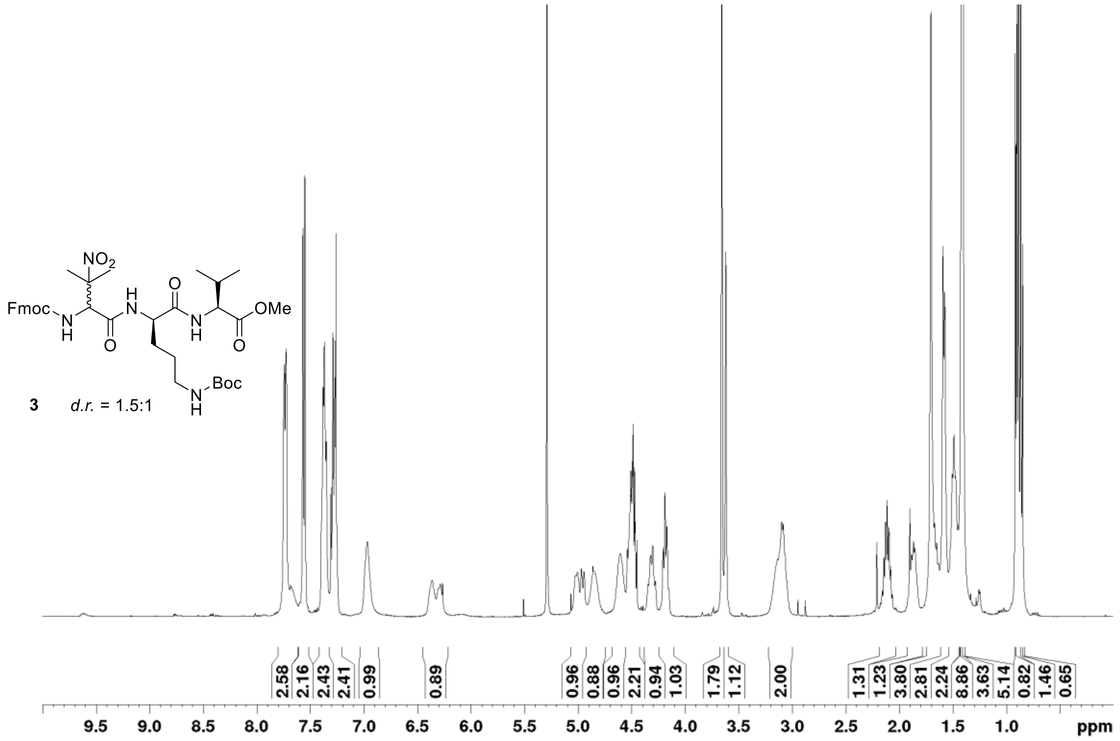
NO2
Fmoc
N
H
O
H
N
O
N
H
O
OMe
N
H
Boc
2.58
2.16
2.43
2.41
0.99
0.89
0.96
0.88
0.96
2.21
0.94
1.03
1.79
1.12
2.00
1.31
1.23
3.80
2.81
2.24
8.86
3.63
5.14
0.82
1.46
0.65
9.5 9.0 8.5 8.0 7.5 7.0 6.5 6.0 5.5 5.0 4.5 4.0 3.5 3.0 2.5 2.0 1.5 1.0 ppm

3 *d.r.* = 1.5:1

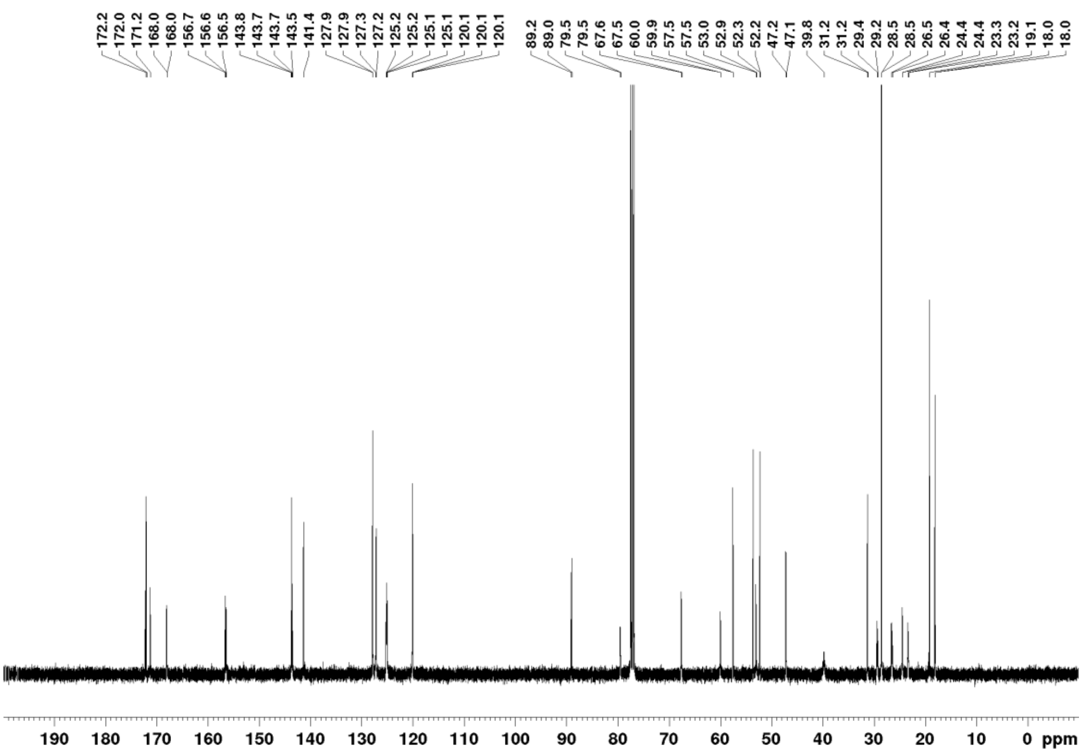
172.2
172.0
171.2
168.0
168.0
156.7
156.6
156.5
143.8
143.7
143.7
143.5
141.4
127.9
127.9
127.3
127.2
125.2
125.2
125.1
125.1
120.1
120.1
120.1
89.2
89.0
79.5
79.5
67.6
67.5
60.0
59.9
57.5
57.5
53.0
52.9
52.3
52.2
47.2
47.1
39.8
31.2
31.2
29.4
29.2
28.5
28.5
26.5
26.4
24.4
24.4
23.3
23.2
19.1
18.0
18.0
190 180 170 160 150 140 130 120 110 100 90 80 70 60 50 40 30 20 10 0 ppm

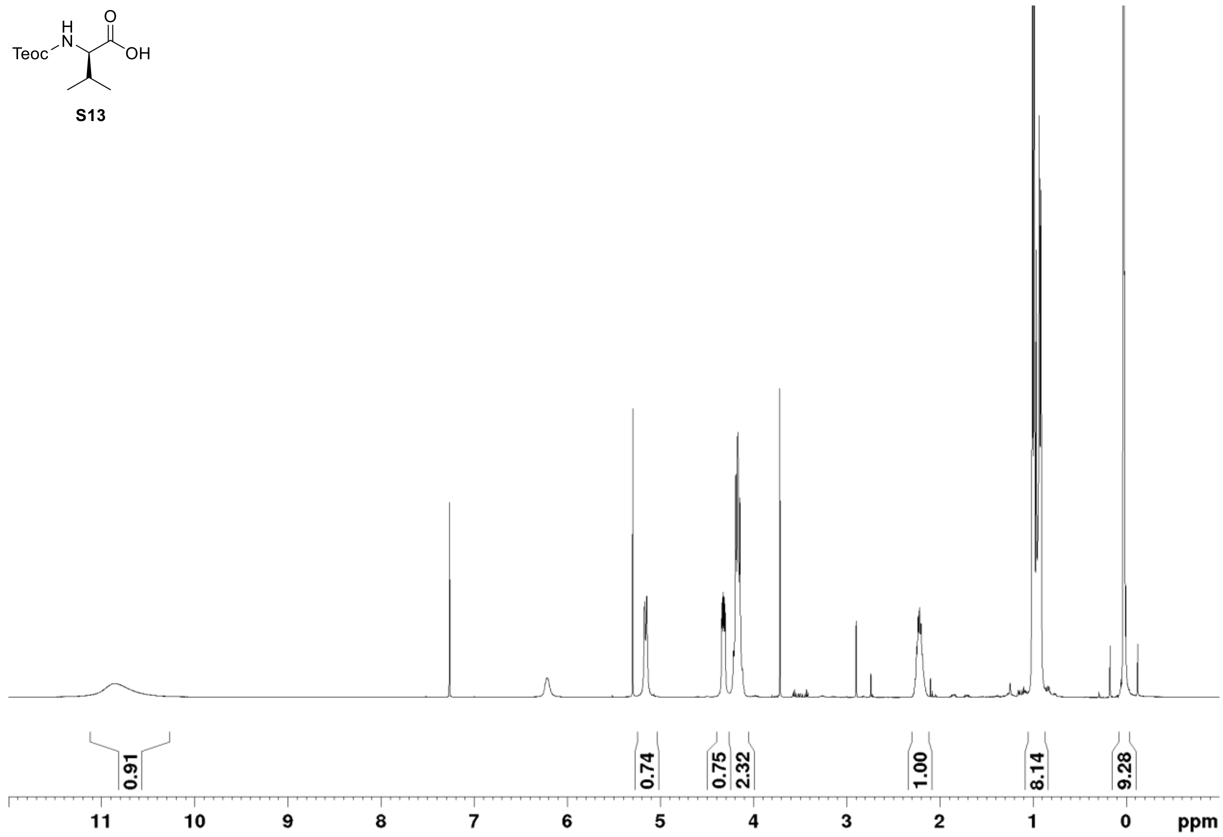
Teoc
N
H
O
OH
S13
0.91
0.74
0.75
2.32
1.00
8.14
9.28
11
10
9
8
7
6
5
4
3
2
1
0
ppm

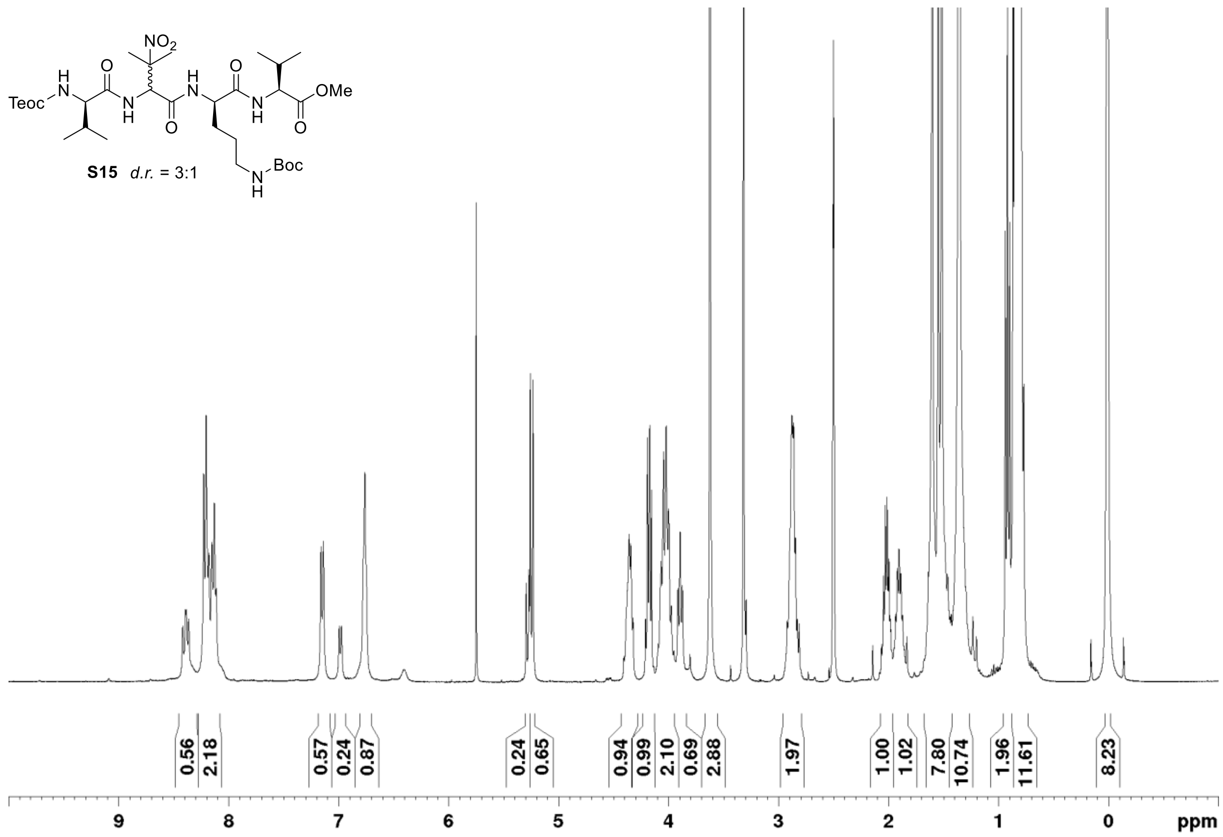
NO2
H
N
Teoc
O
N
H
O
H
N
O
N
H
OMe
O
N
H
Boc

S15 *d.r.* = 3:1

0.56
2.18
0.57
0.24
0.87
0.24
0.65
0.94
0.99
2.10
0.69
2.88
1.97
1.00
1.02
7.80
10.74
1.96
11.61
8.23
9
8
7
6
5
4
3
2
1
0
ppm

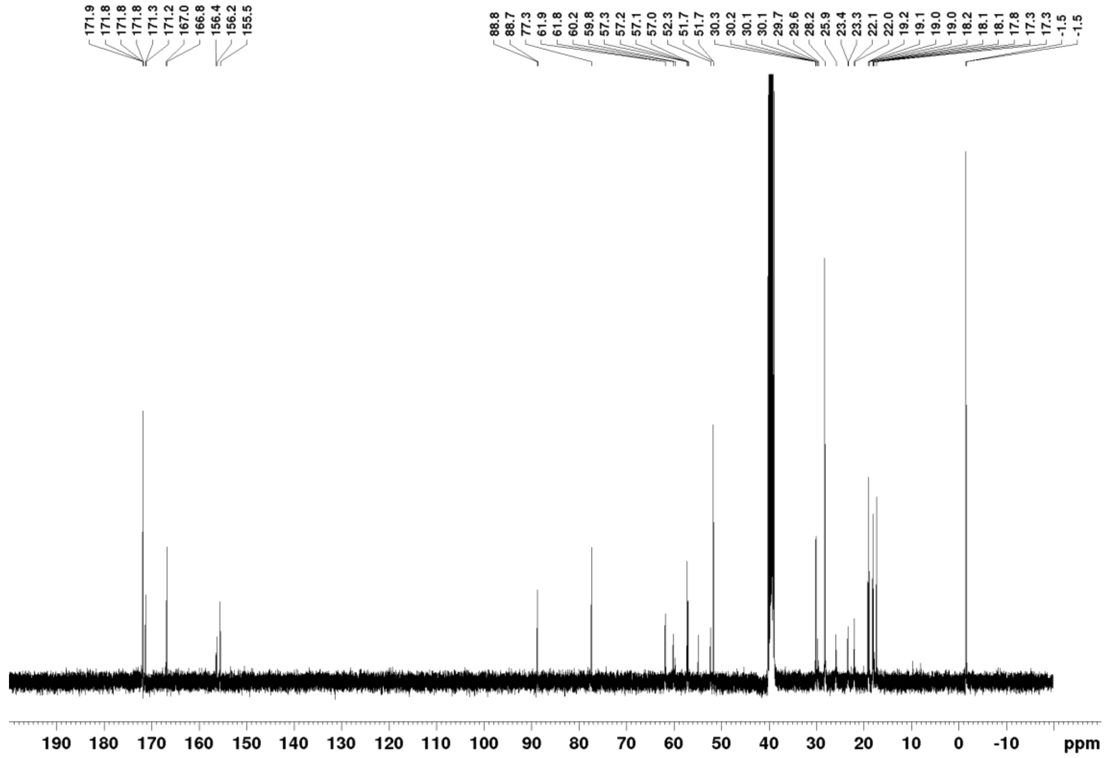
171.9
171.8
171.8
171.8
171.3
171.2
167.0
166.8
156.4
156.2
155.5
88.8
88.7
77.3
61.9
61.8
60.2
59.8
57.3
57.2
57.1
57.0
52.3
51.7
51.7
30.3
30.2
30.1
30.1
29.7
29.6
28.2
25.9
23.4
23.3
22.1
22.0
19.2
19.1
19.0
19.0
18.2
18.1
18.1
17.8
17.3
-1.5
-1.5
190
180
170
160
150
140
130
120
110
100
90
80
70
60
50
40
30
20
10
0
-10
ppm

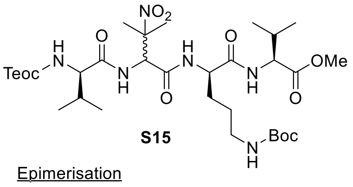

S15

Epimerisation
d.r. = 3:1 -> *d.r.* = 0:1

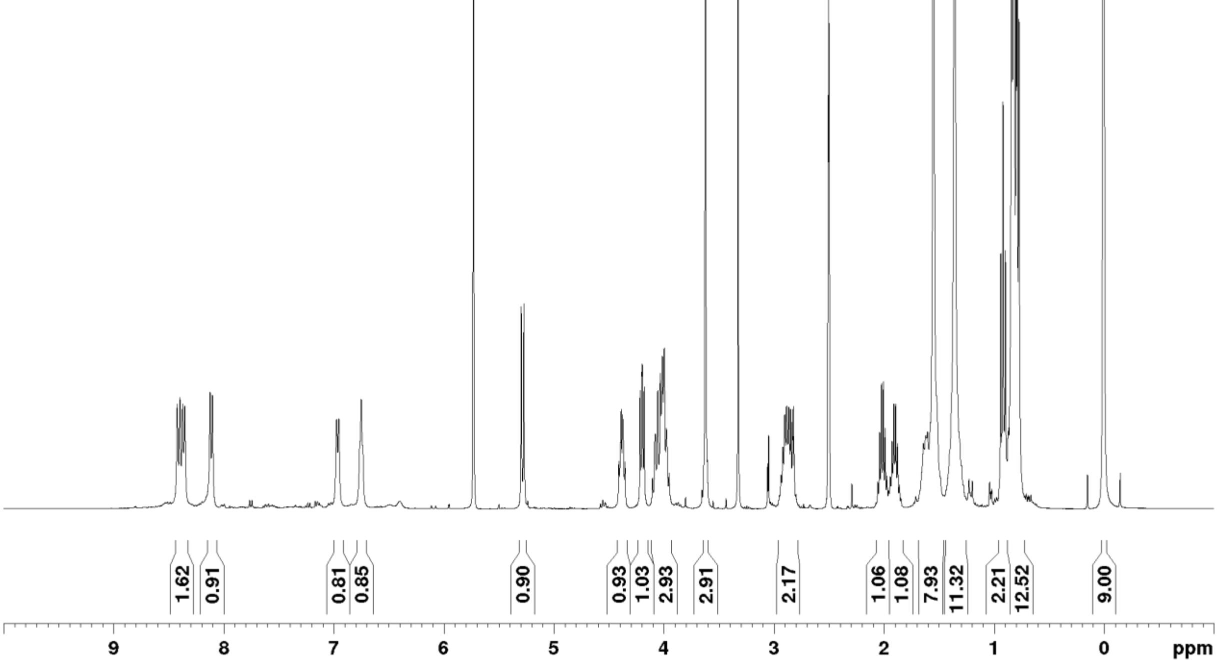

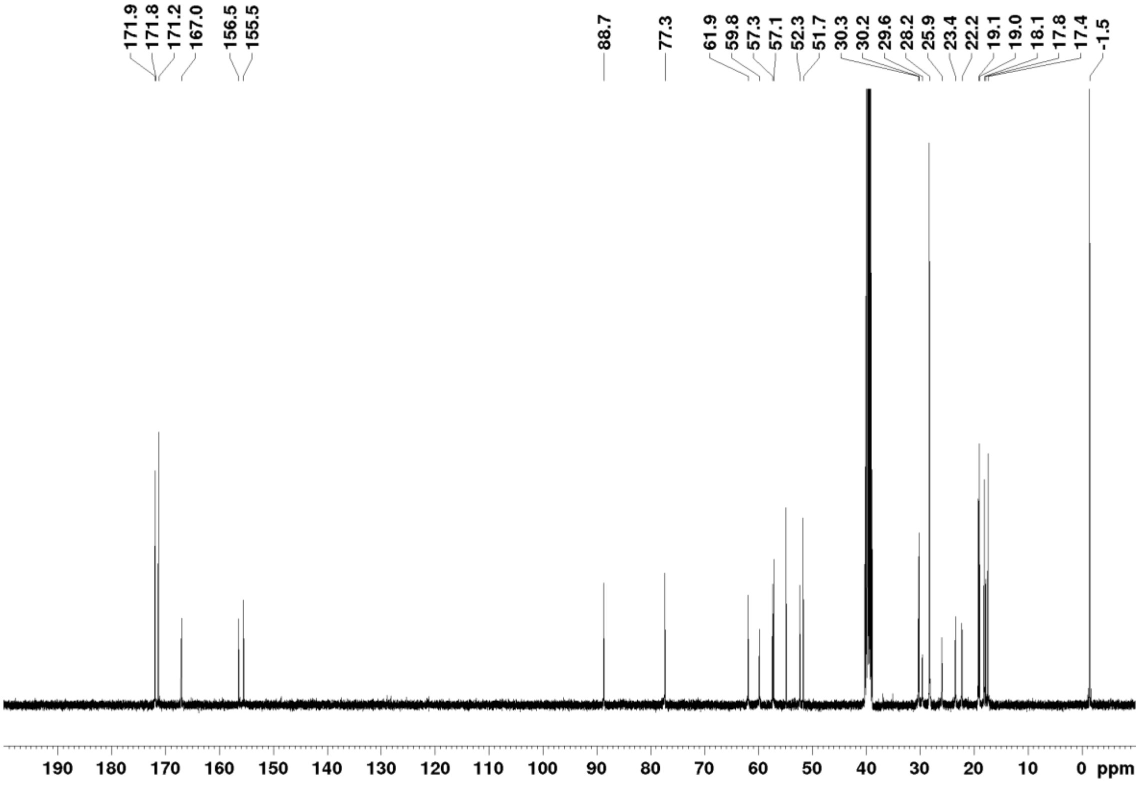

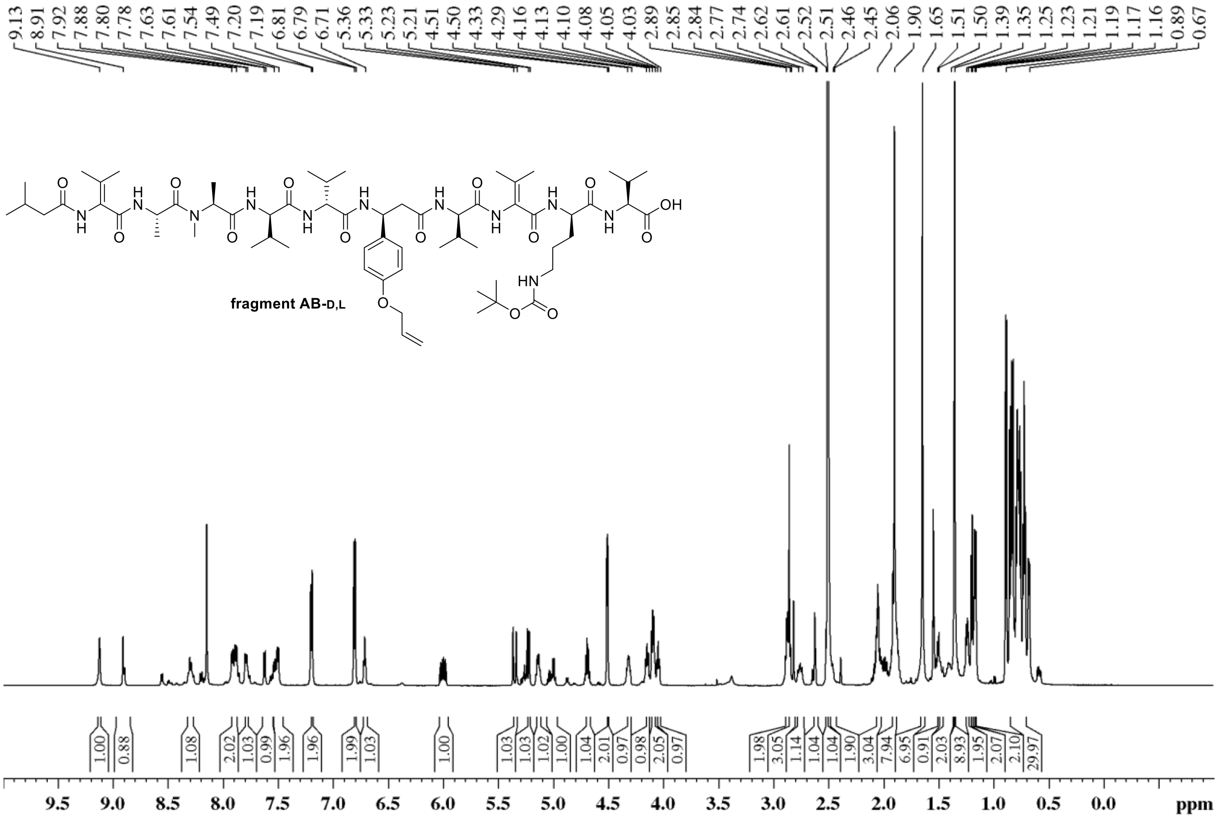
9.13 8.91 7.92 7.88 7.80 7.78 7.63 7.61 7.54 7.49 7.20 7.19 6.81 6.79 6.71 5.36 5.33 5.23 5.21 4.51 4.50 4.33 4.29 4.16 4.13 4.10 4.08 4.05 4.03 2.89 2.85 2.84 2.77 2.74 2.62 2.61 2.52 2.51 2.46 2.45 2.06 1.90 1.65 1.51 1.50 1.39 1.35 1.25 1.23 1.21 1.19 1.17 1.16 0.89 0.67
fragment AB-D,L
1.00 0.88 1.08 2.02 1.03 0.99 1.96 1.96 1.99 1.03 1.00 1.03 1.03 1.02 1.00 1.04 2.01 0.97 0.98 2.05 0.97 1.98 3.05 1.14 1.04 1.04 1.90 3.04 7.94 6.95 0.91 2.03 8.93 1.95 2.07 2.10 29.97
9.5 9.0 8.5 8.0 7.5 7.0 6.5 6.0 5.5 5.0 4.5 4.0 3.5 3.0 2.5 2.0 1.5 1.0 0.5 0.0 ppm

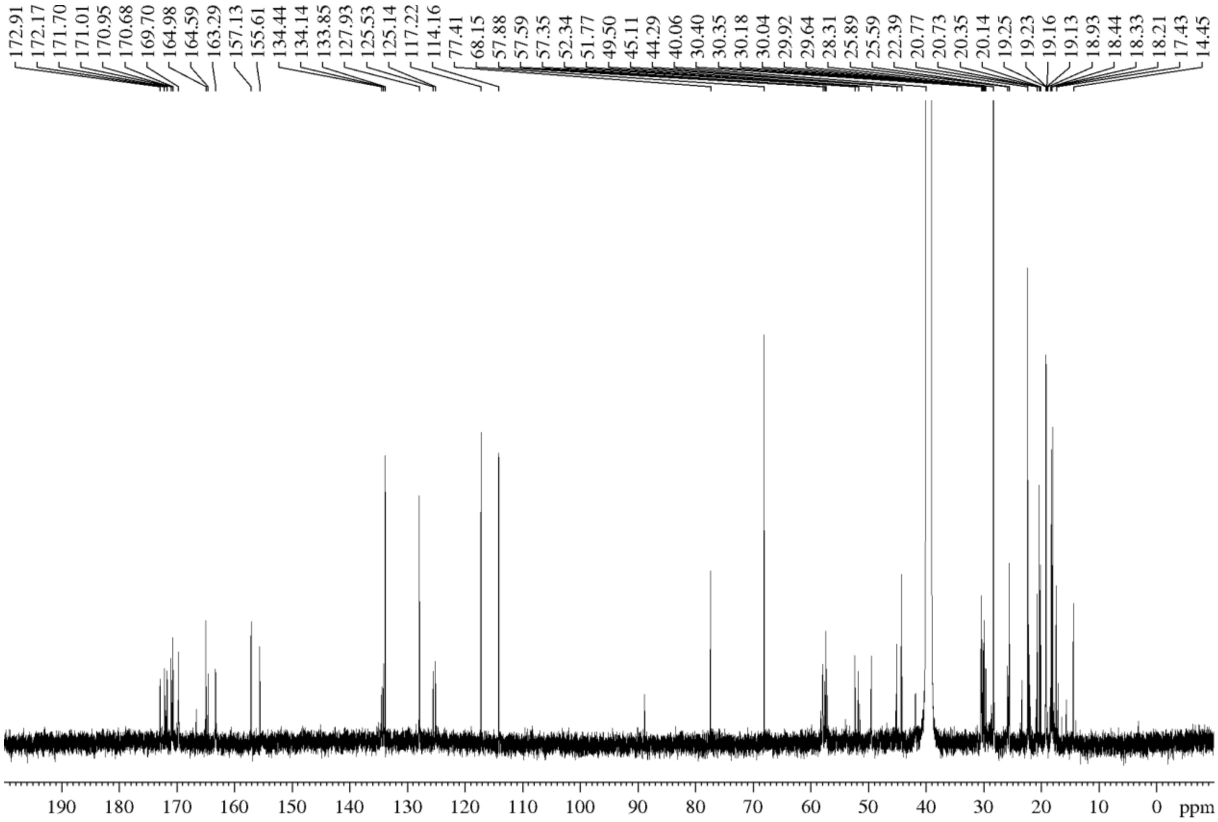
172.91 172.17 171.70 171.01 170.95 170.68 169.70 164.98 164.59 163.29 157.13 155.61 134.44 134.14 133.85 127.93 125.53 125.14 117.22 114.16 77.41 68.15 57.88 57.59 57.35 52.34 51.77 49.50 45.11 44.29 40.06 30.40 30.35 30.18 30.04 29.92 29.64 28.31 25.89 25.59 22.39 20.77 20.73 20.35 20.14 19.25 19.23 19.16 19.13 18.93 18.44 18.33 18.21 17.43 14.45
190 180 170 160 150 140 130 120 110 100 90 80 70 60 50 40 30 20 10 0 ppm

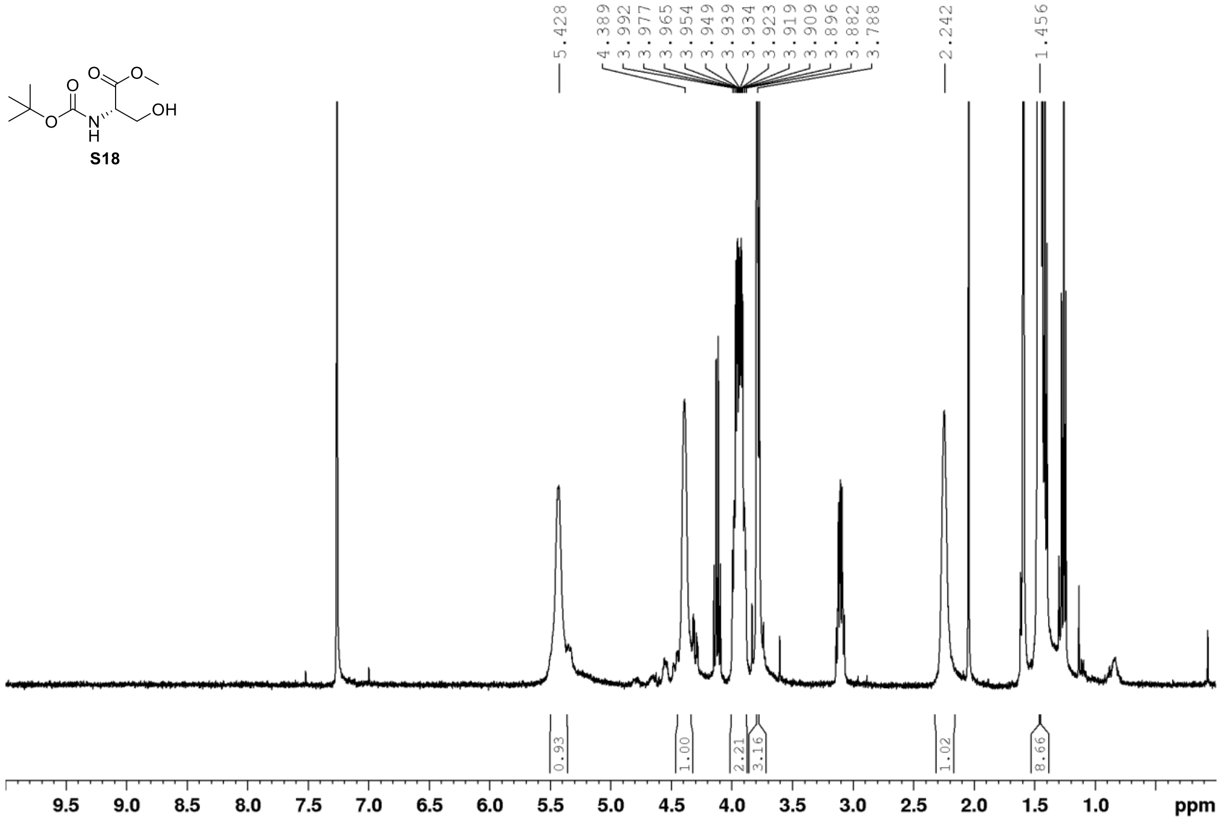
5.428
4.389
3.992
3.977
3.965
3.954
3.949
3.939
3.934
3.923
3.919
3.909
3.896
3.882
3.788
2.242
1.456
0.93
1.00
2.21
3.16
1.02
8.66
9.5 9.0 8.5 8.0 7.5 7.0 6.5 6.0 5.5 5.0 4.5 4.0 3.5 3.0 2.5 2.0 1.5 1.0 ppm
OH

S18

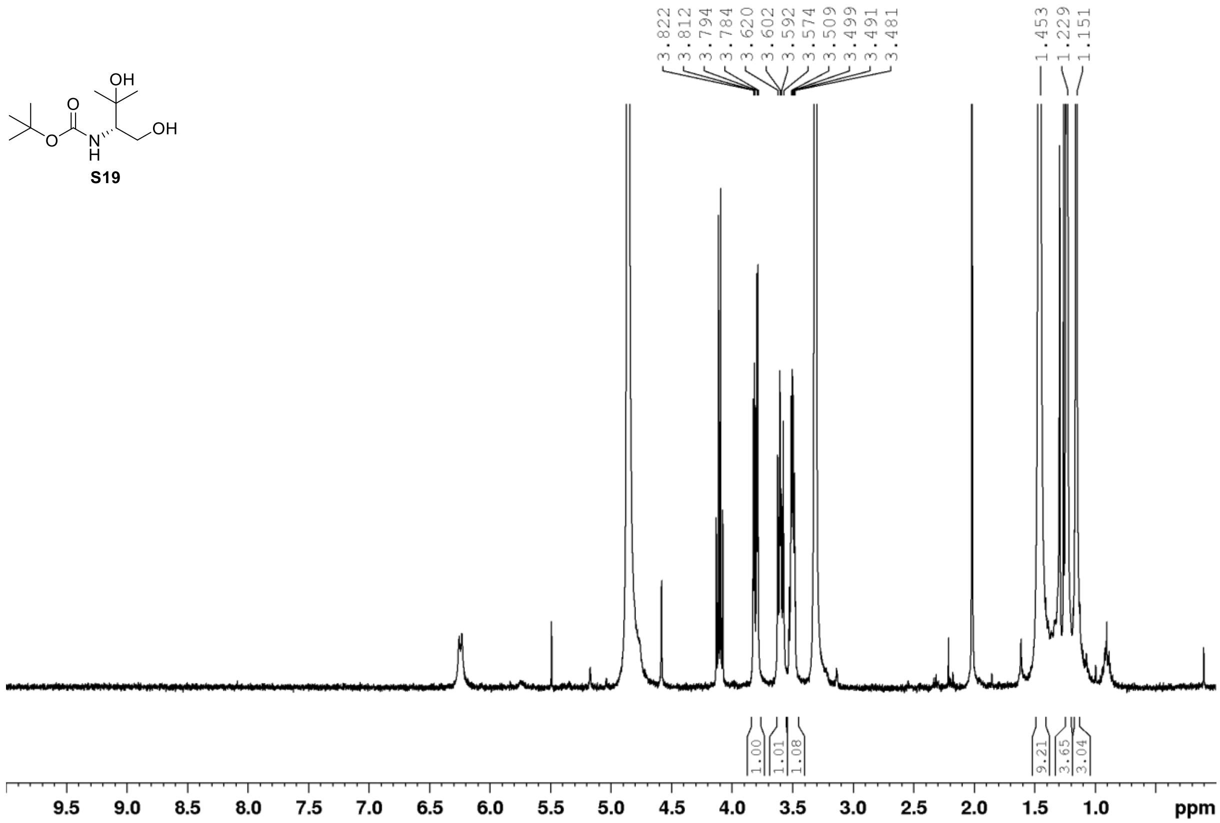
3.822
3.812
3.794
3.784
3.620
3.602
3.592
3.574
3.509
3.499
3.491
3.481
1.453
1.229
1.151
1.00
1.01
1.08
9.21
3.65
3.04
9.5 9.0 8.5 8.0 7.5 7.0 6.5 6.0 5.5 5.0 4.5 4.0 3.5 3.0 2.5 2.0 1.5 1.0 ppm
OH
OH

S19

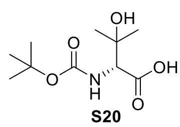

S20

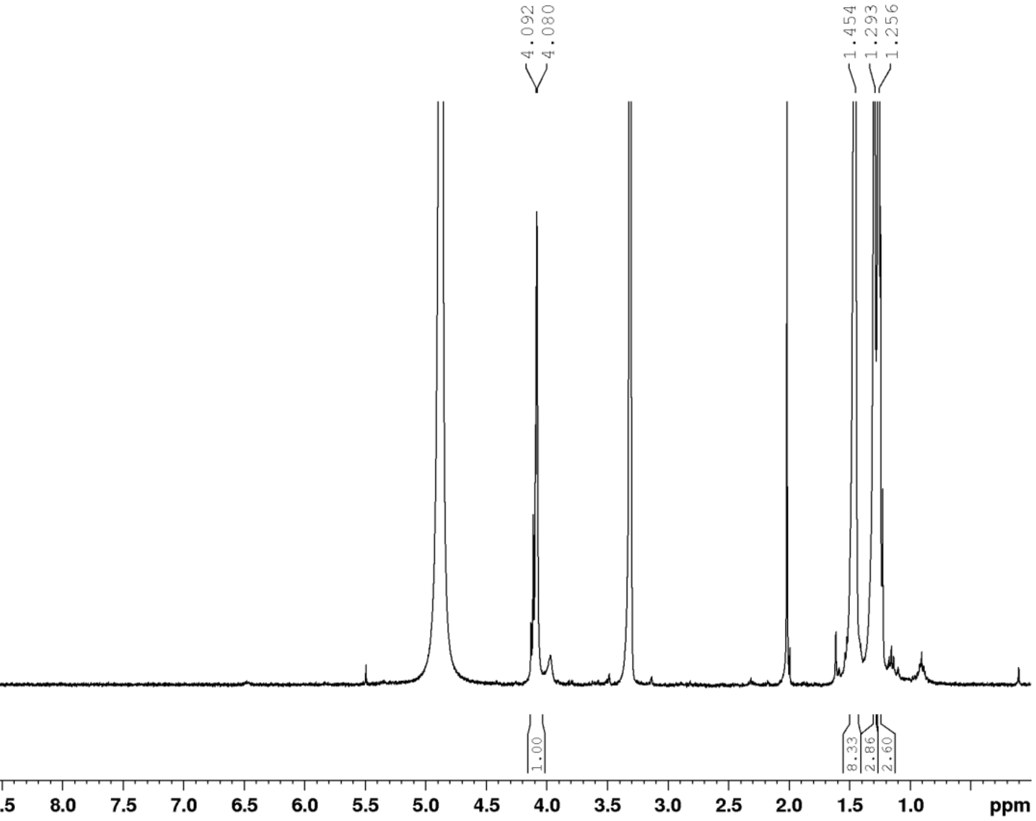

4.092
4.080
1.454
1.293
1.256
1.00
8.33
2.86
2.60
9.5
9.0
8.5
8.0
7.5
7.0
6.5
6.0
5.5
5.0
4.5
4.0
3.5
3.0
2.5
2.0
1.5
1.0
ppm

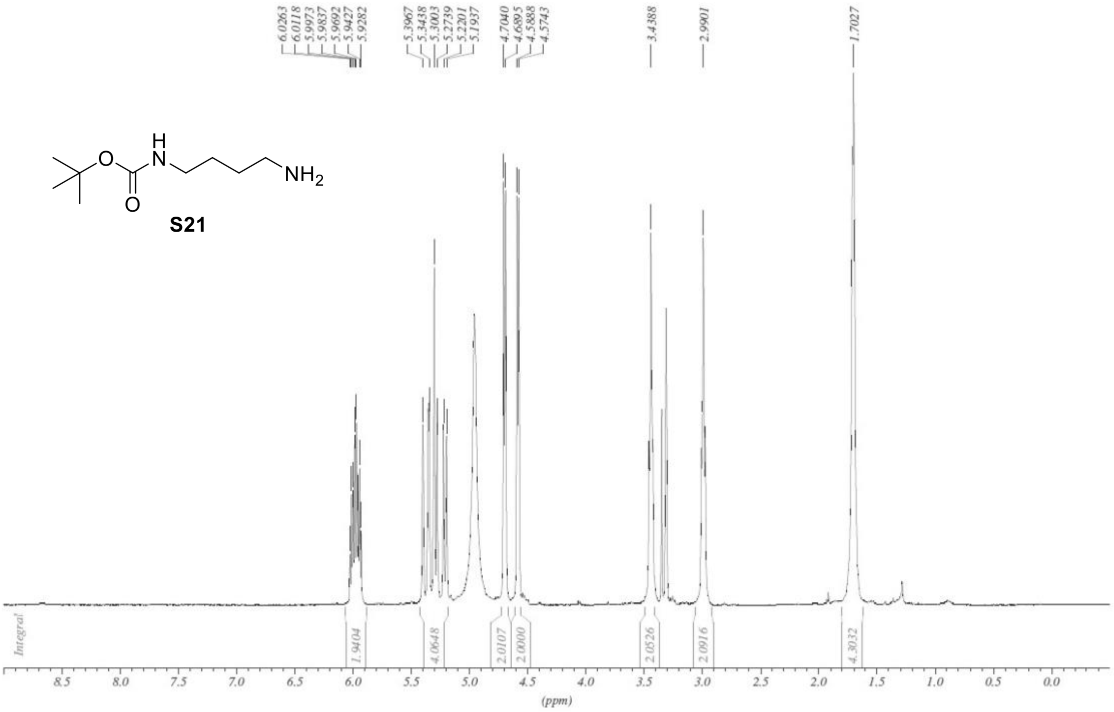
H
N
O
O
NH2
S21
6.0263
6.0118
5.9973
5.9837
5.9692
5.9427
5.9282
5.3967
5.3438
5.3003
5.2739
5.2201
5.1937
4.7040
4.6895
4.5888
4.5743
3.4388
2.9901
1.7027
Integral
1.9404
4.0648
2.0107
2.0000
2.0526
2.0916
4.3032
8.5
8.0
7.5
7.0
6.5
6.0
5.5
5.0
4.5
4.0
3.5
3.0
2.5
2.0
1.5
1.0
0.5
0.0
(ppm)

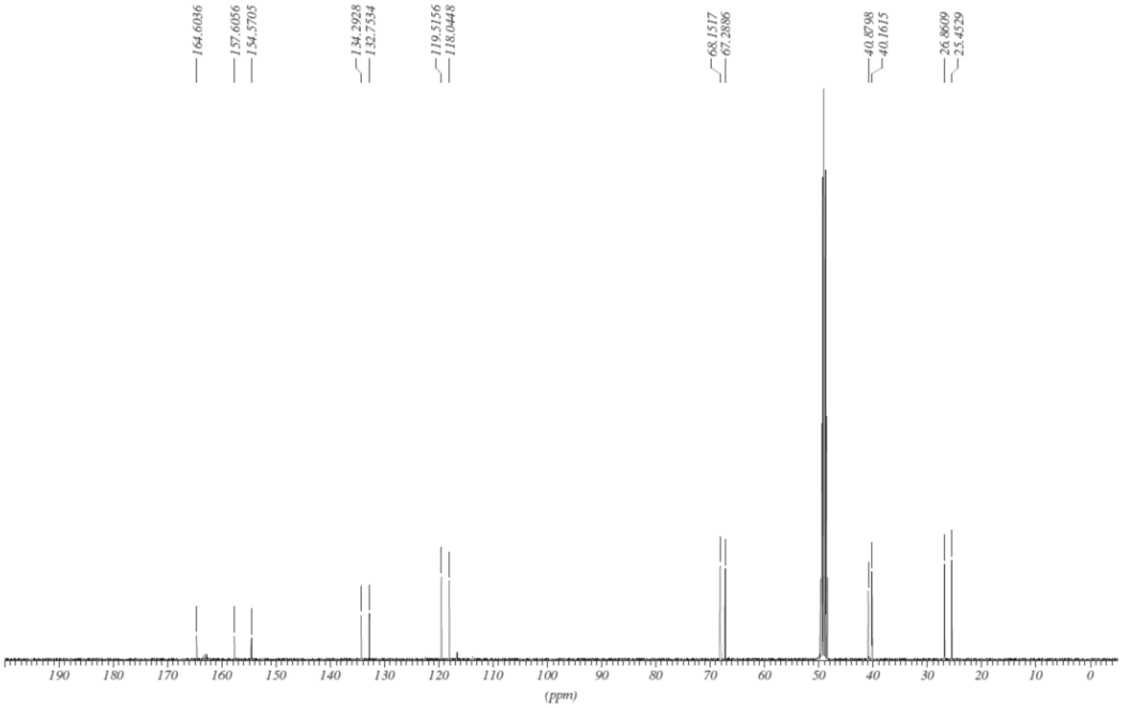
164.6036
157.6056
154.5705
134.2928
132.7534
119.5156
118.0448
68.1517
67.2886
40.8798
40.1615
26.8609
25.4529
190
180
170
160
150
140
130
120
110
100
90
80
70
60
50
40
30
20
10
0
(ppm)

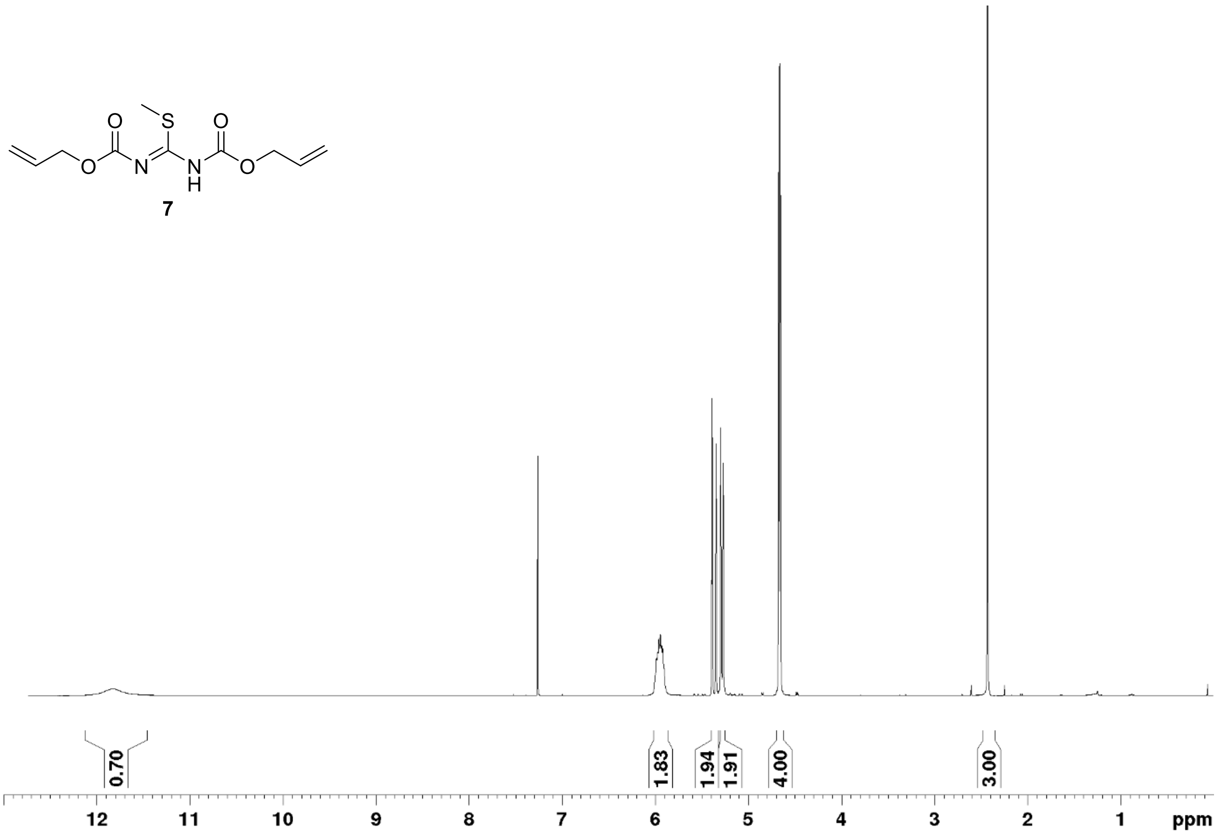
S
O
O
O
N
N
H
O
O
0.70
1.83
1.94
1.91
4.00
3.00
12
11
10
9
8
7
6
5
4
3
2
1
ppm

7

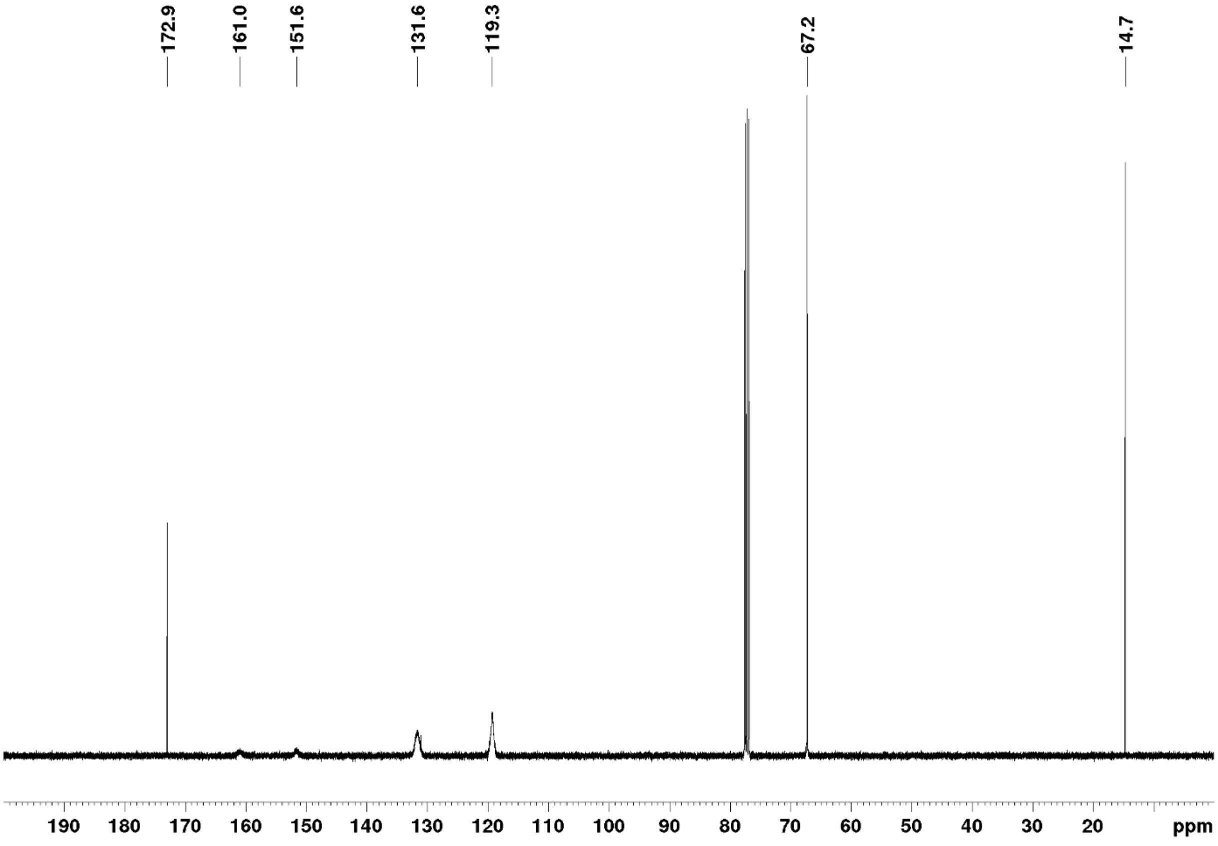
172.9
161.0
151.6
131.6
119.3
67.2
14.7
190
180
170
160
150
140
130
120
110
100
90
80
70
60
50
40
30
20
ppm

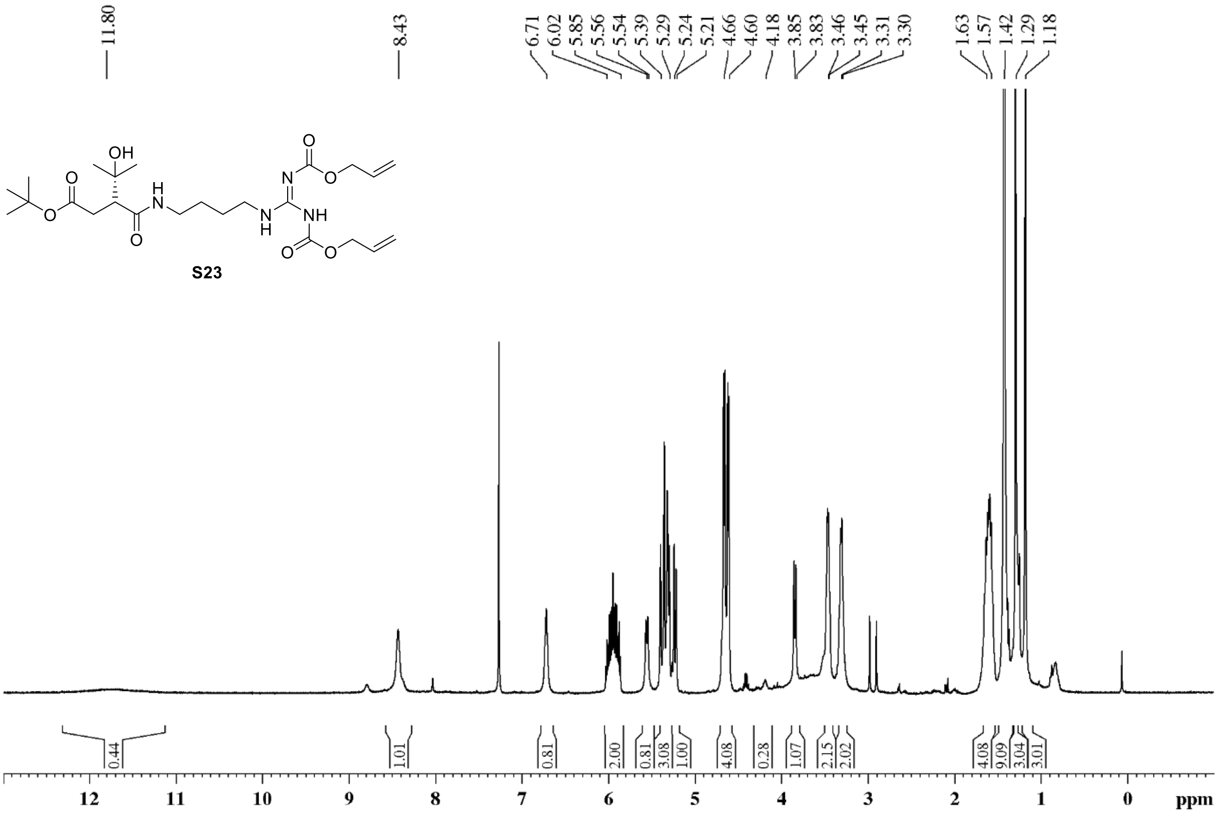
11.80
8.43
6.71 6.02 5.85 5.56 5.54 5.39 5.29 5.24 5.21 4.66 4.60 4.18 3.85 3.83 3.46 3.45 3.31 3.30
1.63 1.57 1.42 1.29 1.18
OH
O
N
H
NH
O
S23
0.44 1.01 0.81 2.00 0.81 3.08 1.00 4.08 0.28 1.07 2.15 2.02 4.08 9.09 3.04 3.01
12 11 10 9 8 7 6 5 4 3 2 1 0 ppm

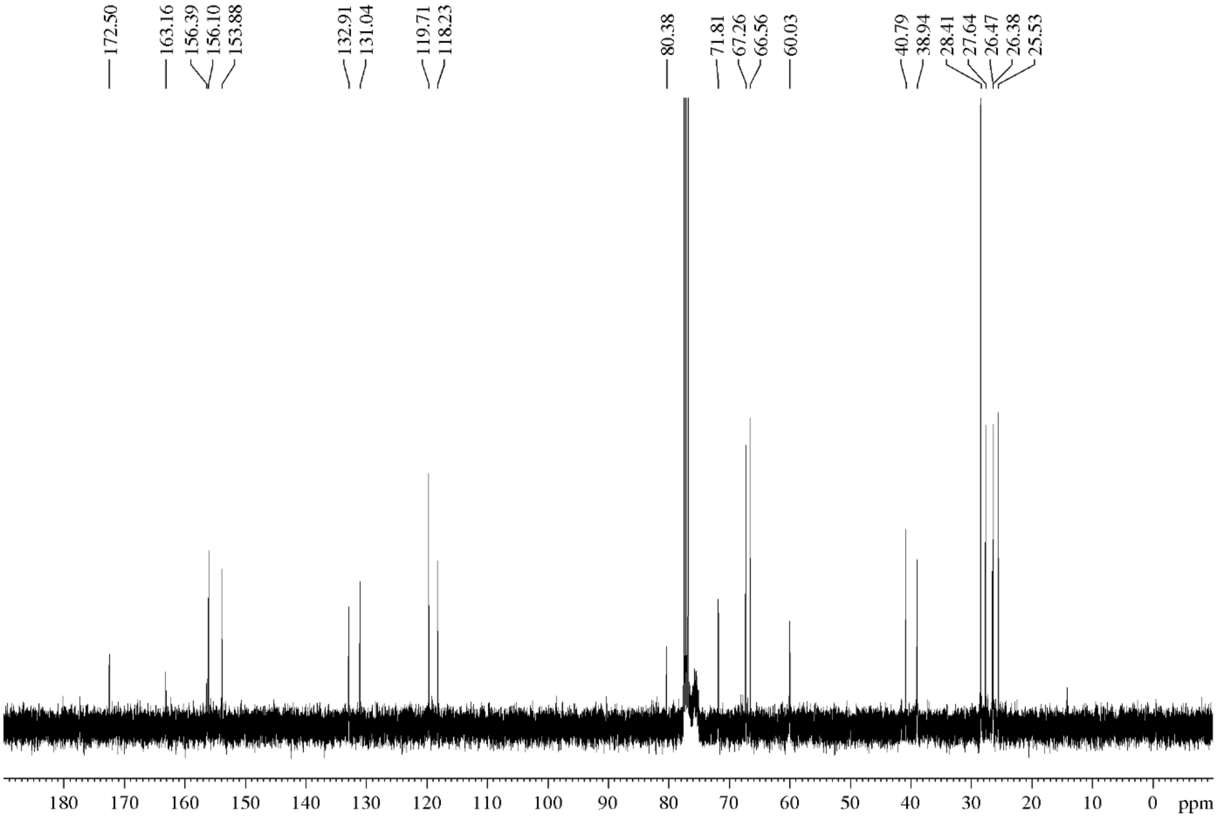
172.50
163.16 156.39 156.10 153.88
132.91 131.04
119.71 118.23
80.38
71.81 67.26 66.56
60.03
40.79 38.94 28.41 27.64 26.47 26.38 25.53
180 170 160 150 140 130 120 110 100 90 80 70 60 50 40 30 20 10 0 ppm

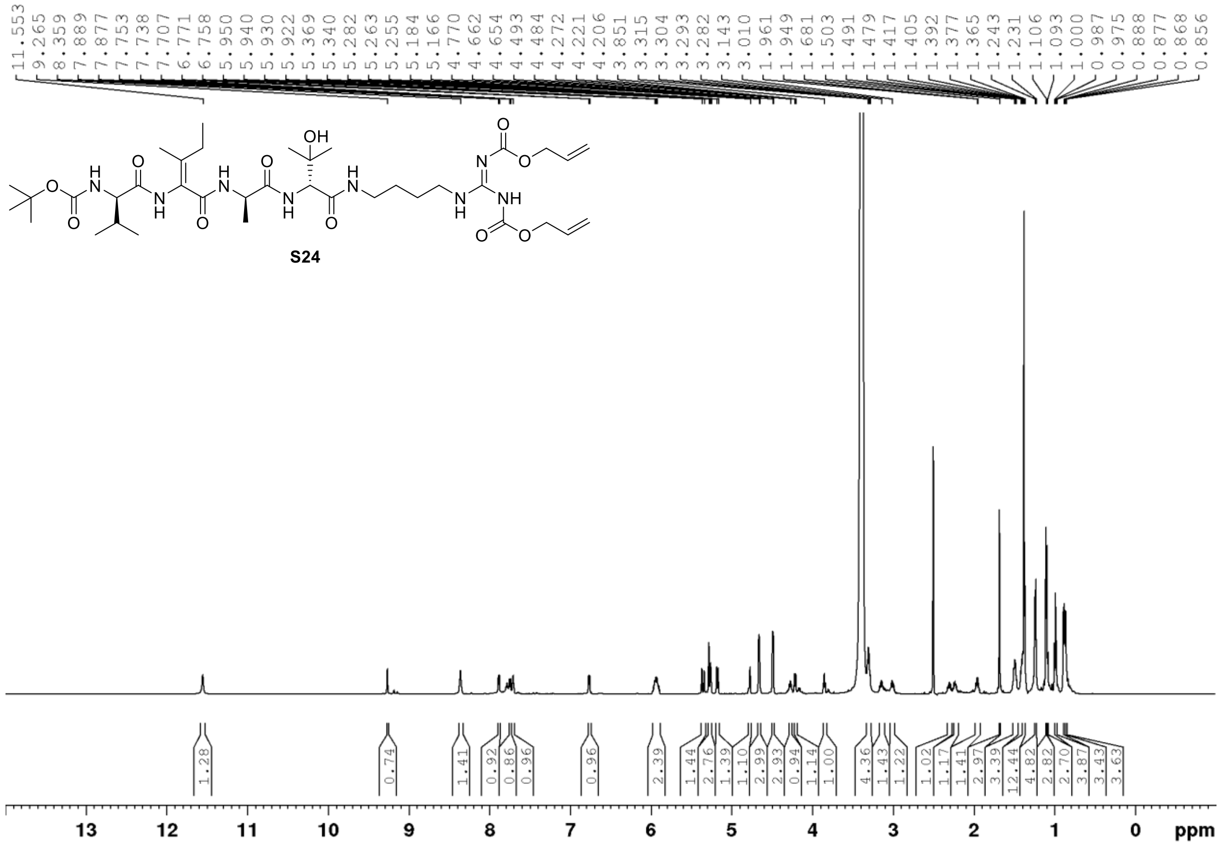
11.553
9.265
8.359
7.889
7.877
7.753
7.738
7.707
6.771
6.758
5.950
5.940
5.930
5.922
5.369
5.340
5.282
5.263
5.255
5.184
5.166
4.770
4.662
4.654
4.493
4.484
4.272
4.221
4.206
3.851
3.315
3.304
3.293
3.282
3.143
3.010
1.961
1.949
1.681
1.503
1.491
1.479
1.417
1.405
1.392
1.377
1.365
1.243
1.231
1.106
1.093
1.000
0.987
0.975
0.888
0.877
0.868
0.856
OH
O
N
O
H
N
H
N
O
N
H
N
H
N
H
N
H
NH
O
O
O
O
O
O
S24
1.28
0.74
1.41
0.92
0.86
0.96
0.96
2.39
1.44
2.76
1.39
1.10
2.99
2.93
0.94
1.14
1.00
4.36
1.43
1.22
1.02
1.17
1.41
2.97
3.39
12.44
4.82
2.82
2.70
3.87
3.43
3.63
13
12
11
10
9
8
7
6
5
4
3
2
1
0
ppm

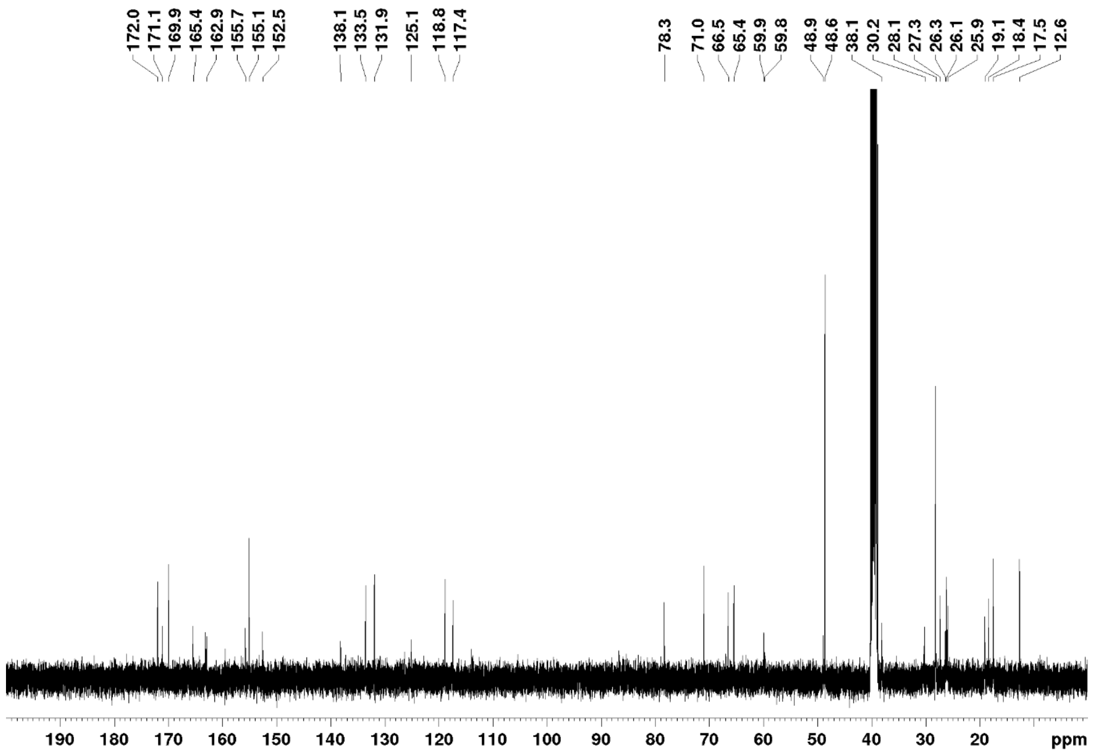
172.0
171.1
169.9
165.4
162.9
155.7
155.1
152.5
138.1
133.5
131.9
125.1
118.8
117.4
78.3
71.0
66.5
65.4
59.9
59.8
48.9
48.6
38.1
30.2
28.1
27.3
26.3
26.1
25.9
19.1
18.4
17.5
12.6
190
180
170
160
150
140
130
120
110
100
90
80
70
60
50
40
30
20
ppm

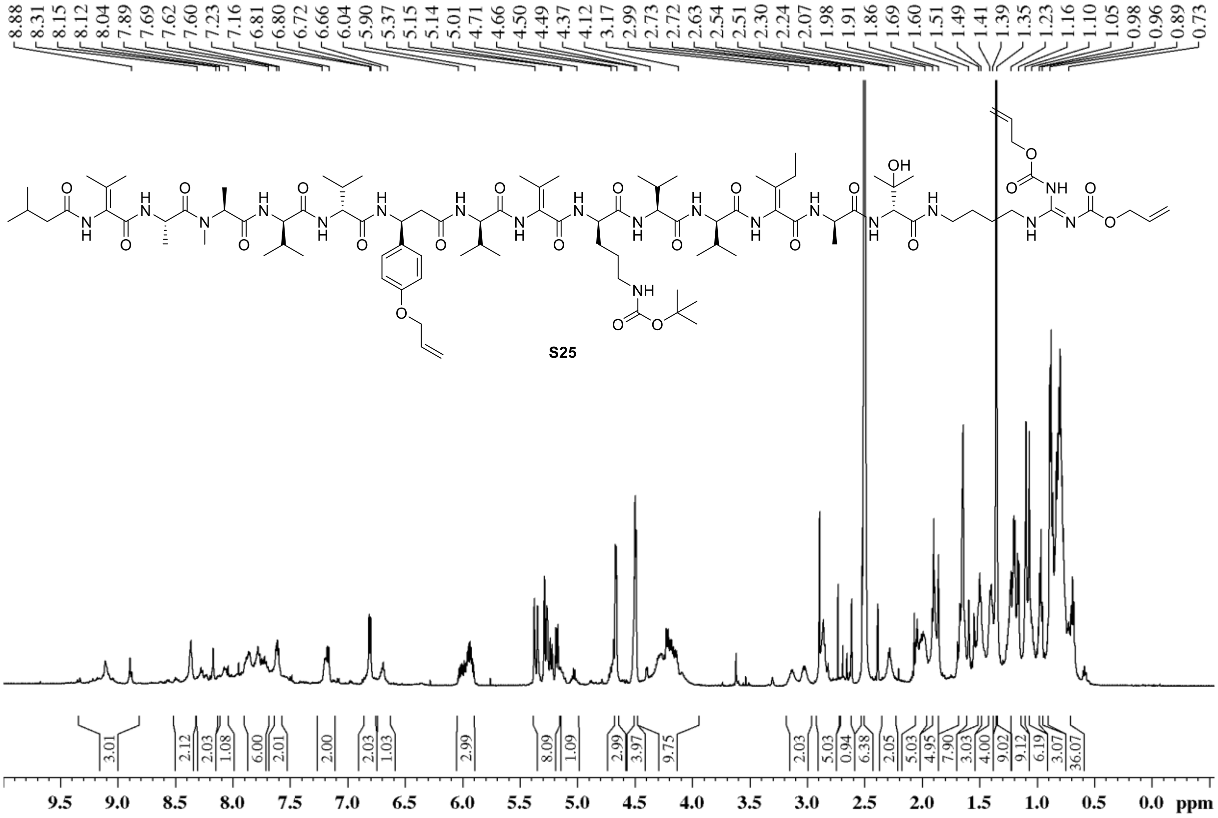
8.88 8.31 8.15 8.12 8.04 7.89 7.69 7.62 7.60 7.23 7.16 6.81 6.80 6.72 6.66 6.04 5.90 5.37 5.15 5.14 5.01 4.71 4.66 4.50 4.49 4.37 4.12 3.17 2.99 2.73 2.72 2.63 2.54 2.51 2.30 2.24 2.07 1.98 1.91 1.86 1.69 1.60 1.51 1.49 1.41 1.39 1.35 1.23 1.16 1.10 1.05 0.98 0.96 0.89 0.73
S25
3.01 2.12 2.03 1.08 6.00 2.01 2.00 2.03 1.03 2.99 8.09 1.09 2.99 3.97 9.75 2.03 5.03 0.94 6.38 2.05 5.03 4.95 7.90 3.03 4.00 9.02 9.12 6.19 3.07 36.07
9.5 9.0 8.5 8.0 7.5 7.0 6.5 6.0 5.5 5.0 4.5 4.0 3.5 3.0 2.5 2.0 1.5 1.0 0.5 0.0 ppm

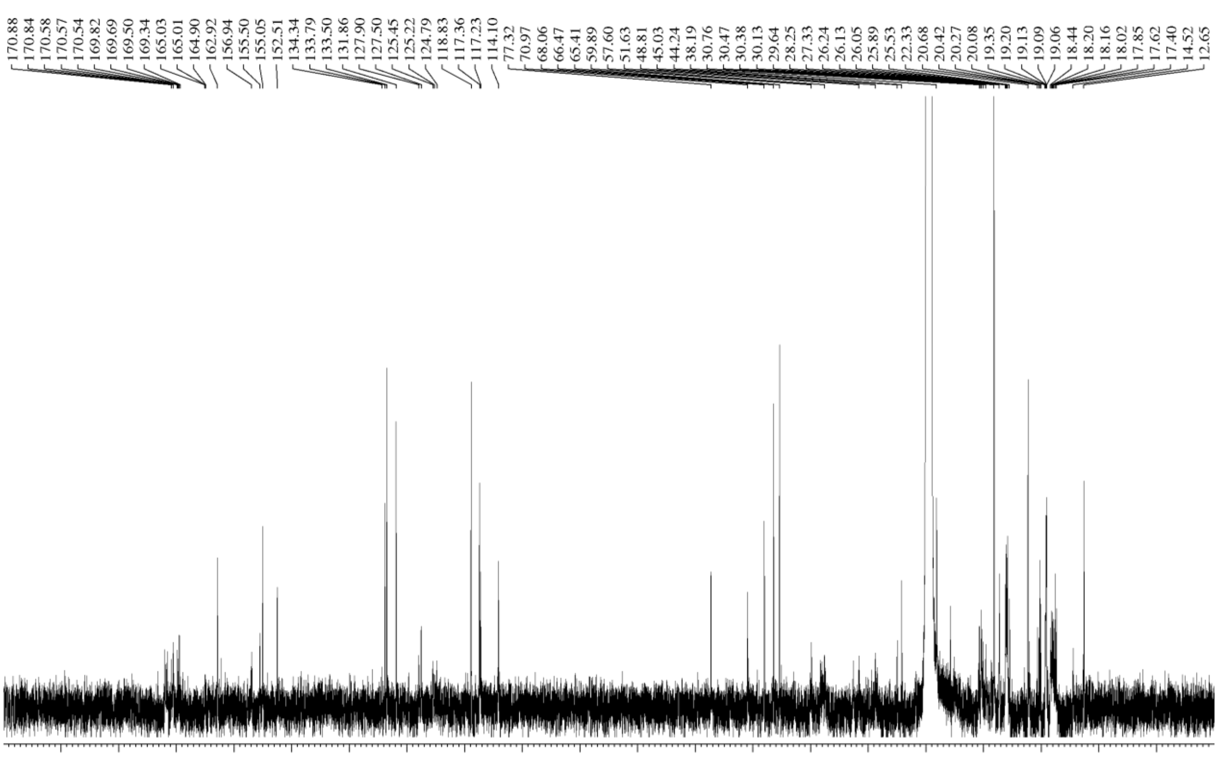
170.88 170.84 170.58 170.57 170.54 169.82 169.69 169.50 169.34 165.03 165.01 164.90 162.92 156.94 155.50 155.05 152.51 134.34 133.79 133.50 131.86 127.90 127.50 125.45 125.22 124.79 118.83 117.36 117.23 114.10 77.32 70.97 68.06 66.47 65.41 59.89 57.60 51.63 48.81 45.03 44.24 38.19 30.76 30.47 30.38 30.13 29.64 28.25 27.33 26.24 26.13 26.05 25.89 25.53 22.33 20.68 20.42 20.27 20.08 19.35 19.20 19.13 19.09 19.06 18.44 18.20 18.16 18.02 17.85 17.62 17.40 14.52 12.65
190 180 170 160 150 140 130 120 110 100 90 80 70 60 50 40 30 20 10 0 ppm

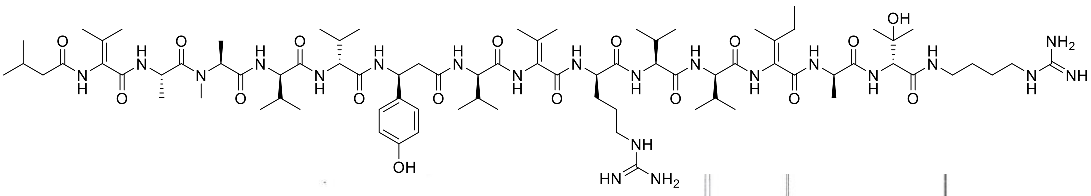

myxovalargin A / myxovalargin-D,L

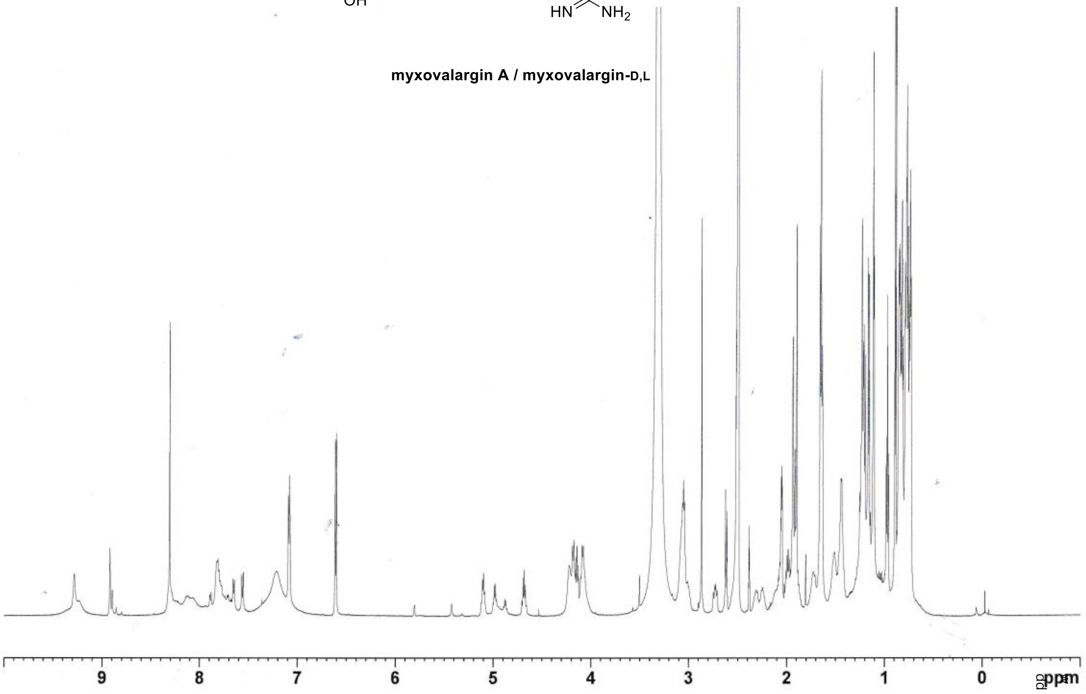

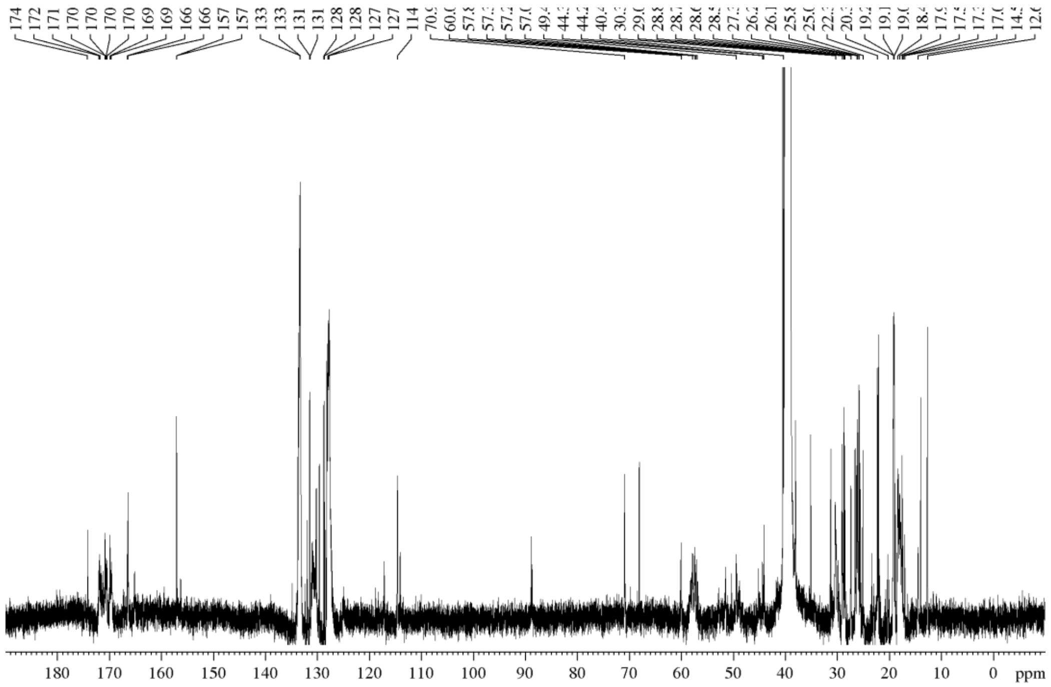

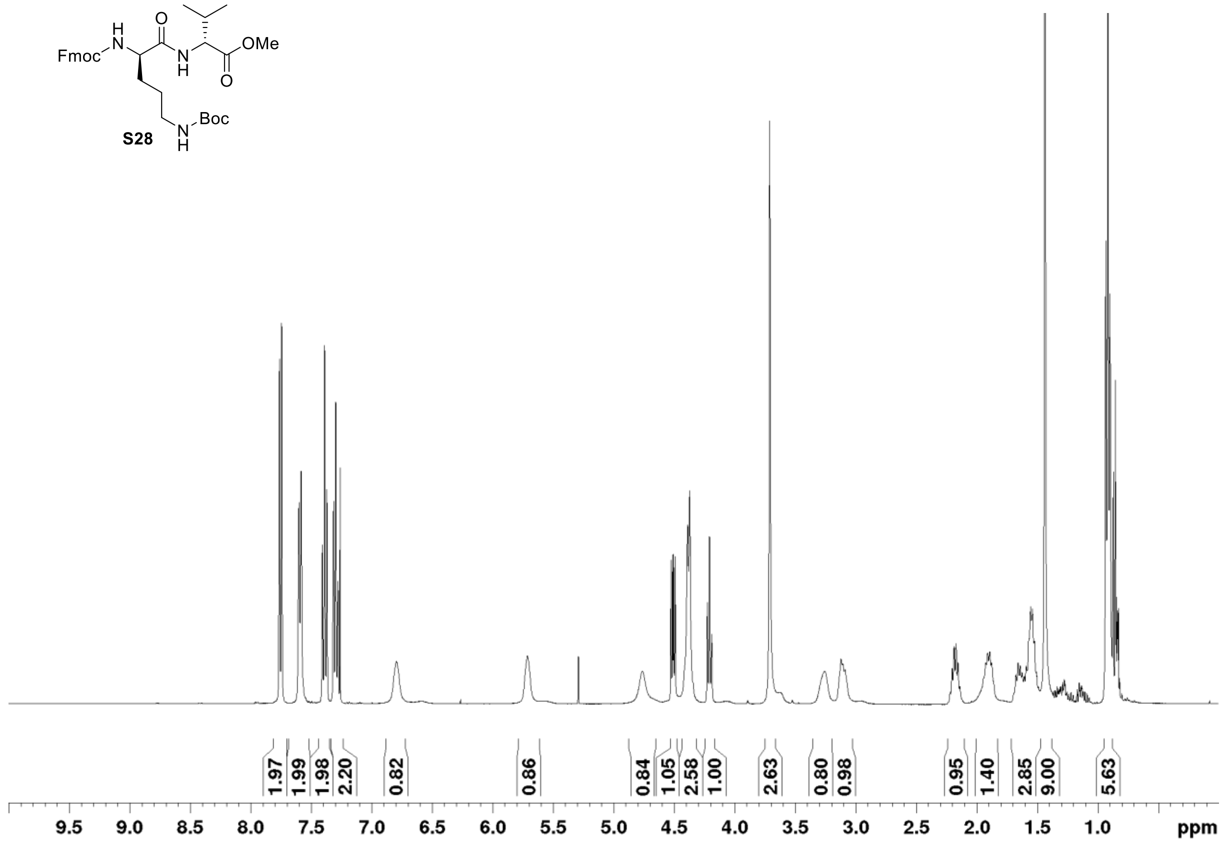

Fmoc
OMe
Boc
S28
1.97
1.99
1.98
2.20
0.82
0.86
0.84
1.05
2.58
1.00
2.63
0.80
0.98
0.95
1.40
2.85
9.00
5.63
9.5
9.0
8.5
8.0
7.5
7.0
6.5
6.0
5.5
5.0
4.5
4.0
3.5
3.0
2.5
2.0
1.5
1.0
ppm

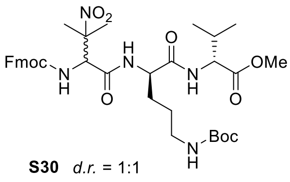
Fmoc
NO2
OMe
Boc

S30 *d.r.* = 1:1

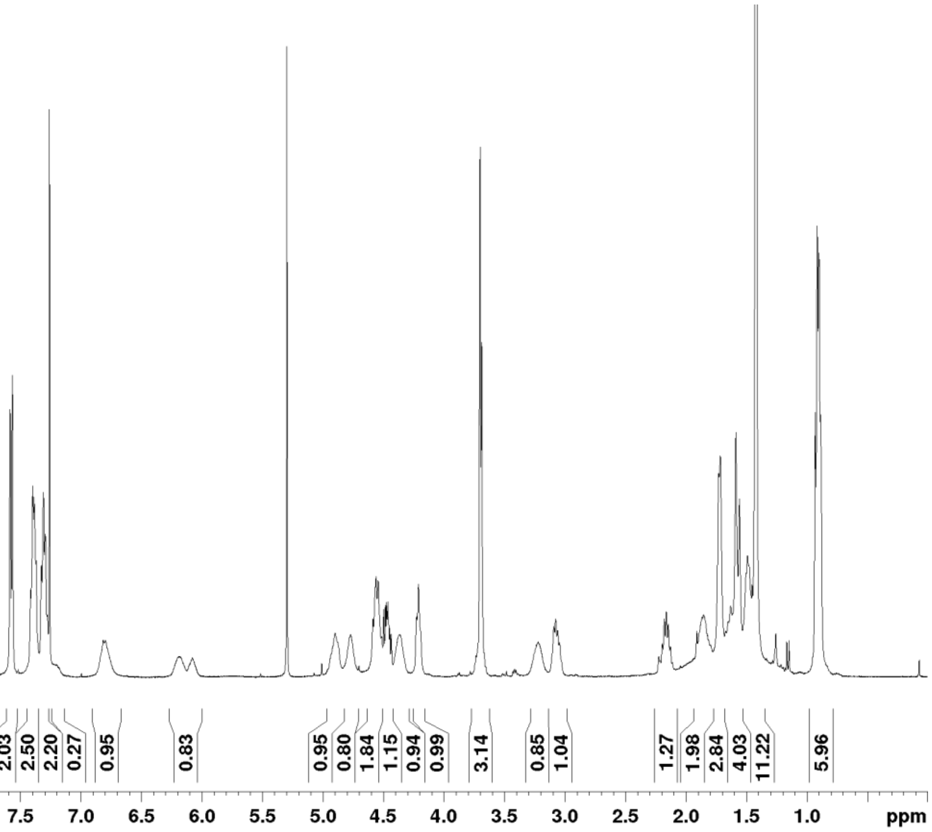
2.00
2.03
2.50
2.20
0.27
0.95
0.83
0.95
0.80
1.84
1.15
0.94
0.99
3.14
0.85
1.04
1.27
1.98
2.84
4.03
11.22
5.96
ppm

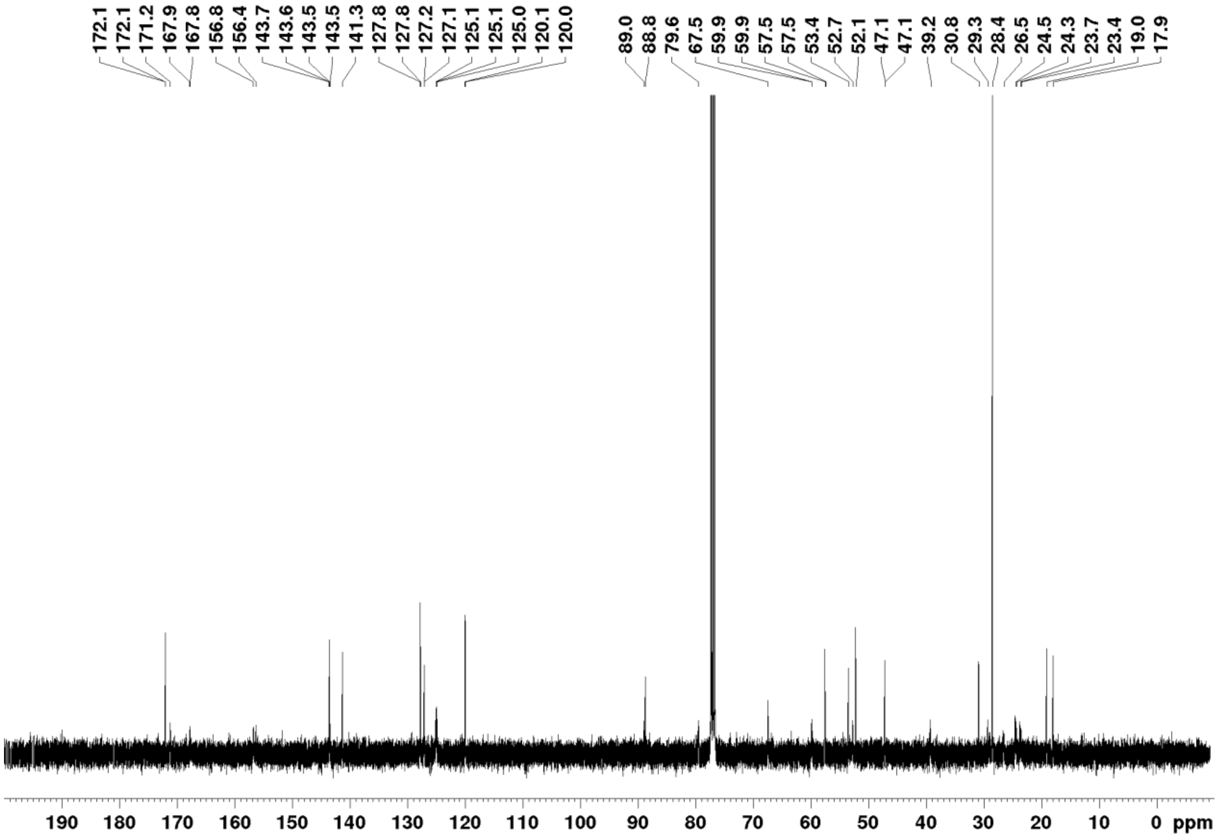
172.1
172.1
171.2
167.9
167.8
156.8
156.4
143.7
143.6
143.5
143.5
141.3
127.8
127.8
127.2
127.1
125.1
125.1
125.0
120.1
120.0
89.0
88.8
79.6
67.5
59.9
59.9
57.5
57.5
53.4
52.7
52.1
47.1
39.2
30.8
29.3
28.4
26.5
24.5
24.3
23.7
23.4
19.0
17.9
ppm

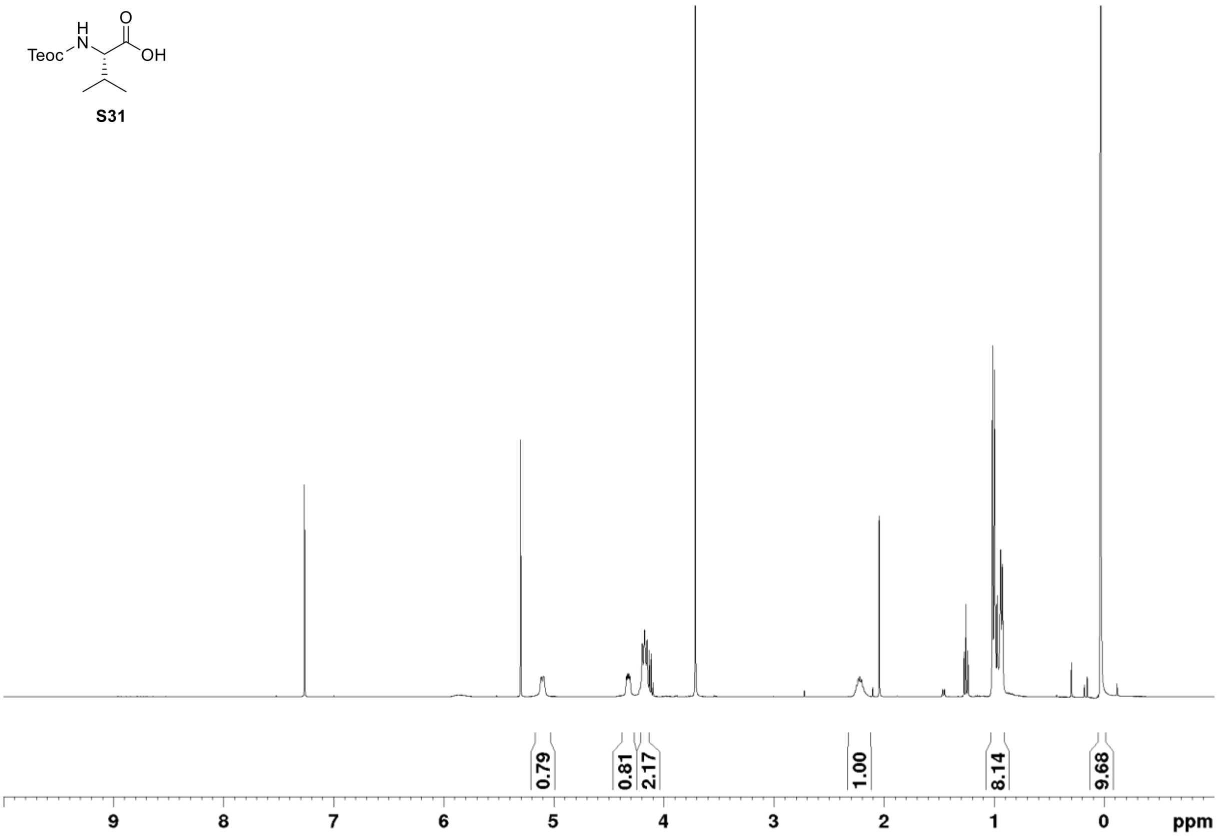
Teoc
H
N
O
OH
S31
0.79
0.81
2.17
1.00
8.14
9.68
9
8
7
6
5
4
3
2
1
0
ppm

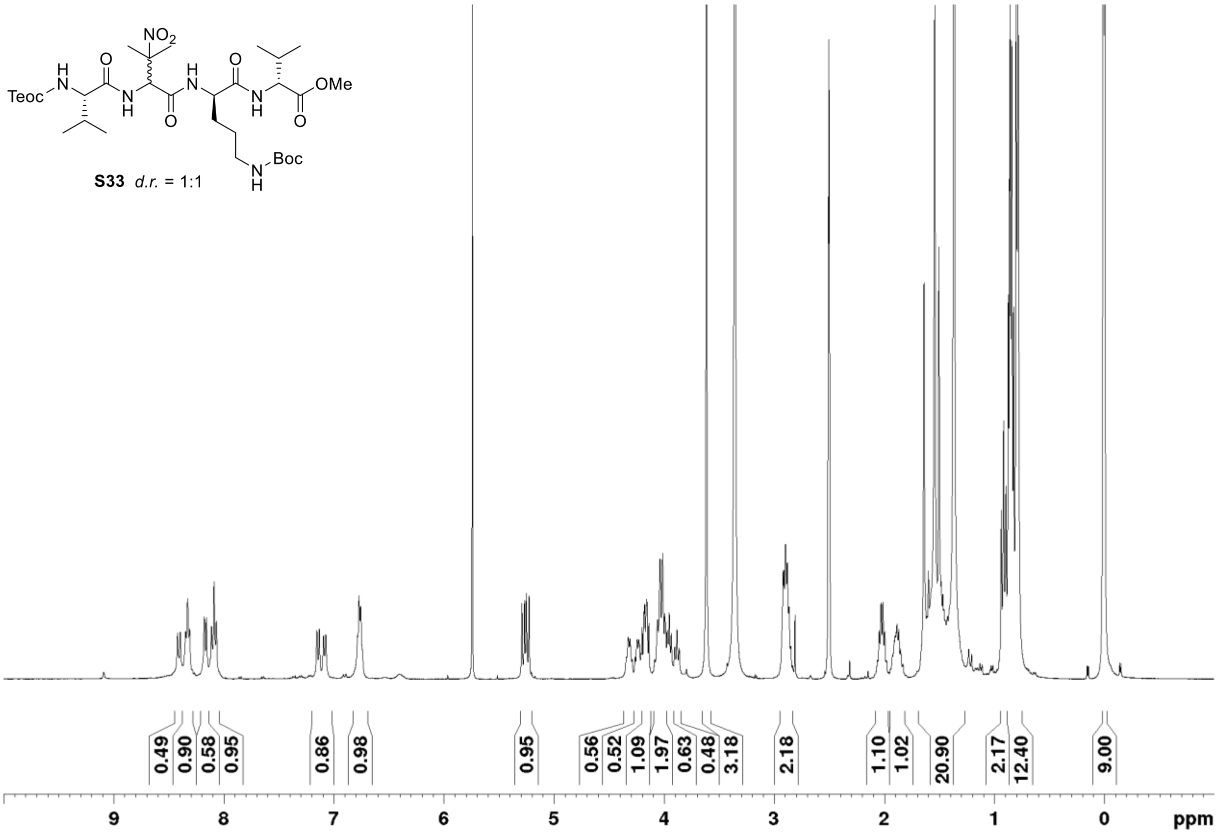
NO$_2$
Teoc
OMe
Boc
S33 *d.r.* = 1:1
0.49
0.90
0.58
0.95
0.86
0.98
0.95
0.56
0.52
1.09
1.97
0.63
0.48
3.18
2.18
1.10
1.02
20.90
2.17
12.40
9.00
9 8 7 6 5 4 3 2 1 0 ppm

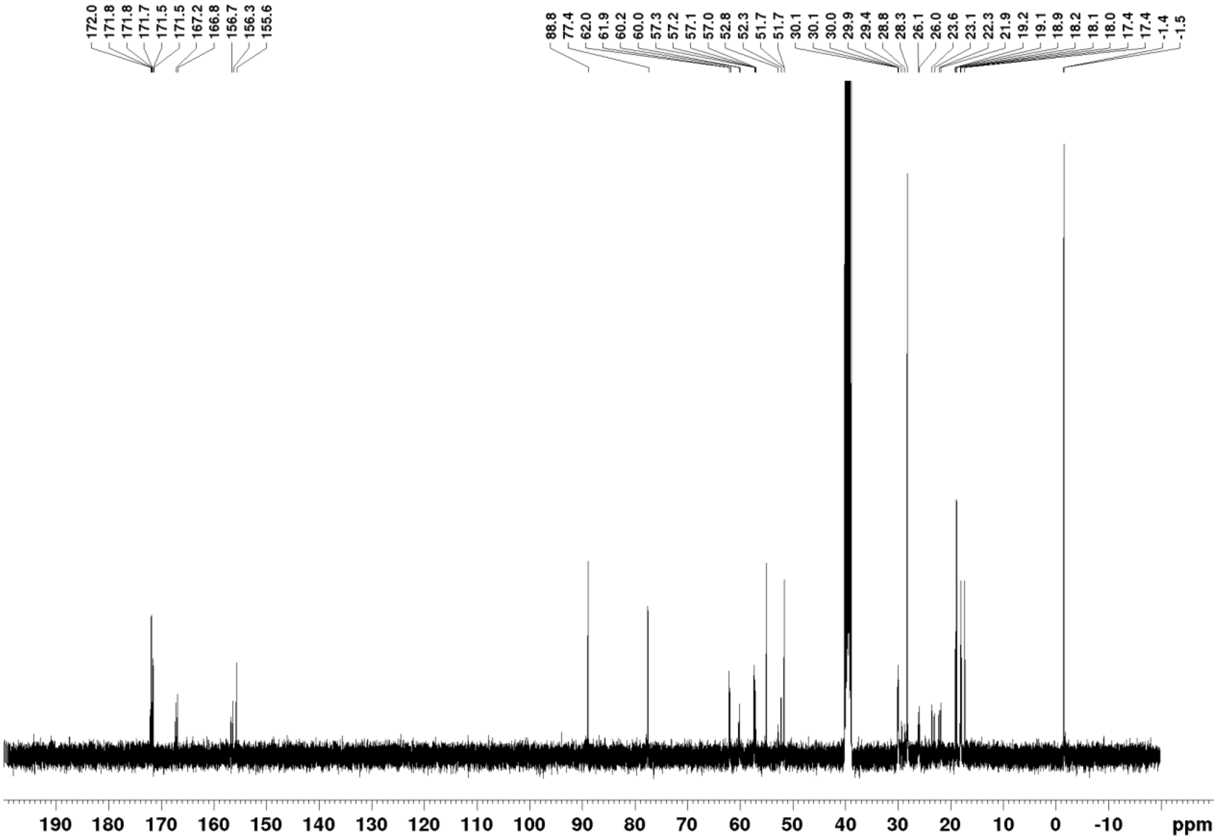
172.0
171.8
171.8
171.7
171.5
171.5
167.2
166.8
156.7
156.3
155.6
88.8
77.4
62.0
61.9
60.2
60.0
57.3
57.2
57.1
57.0
52.8
52.3
51.7
51.7
30.1
30.0
29.9
29.4
28.8
28.3
26.1
26.0
23.6
23.1
22.3
21.9
19.2
19.1
18.9
18.2
18.1
18.0
17.4
17.4
-1.4
-1.5
190 180 170 160 150 140 130 120 110 100 90 80 70 60 50 40 30 20 10 0 -10 ppm

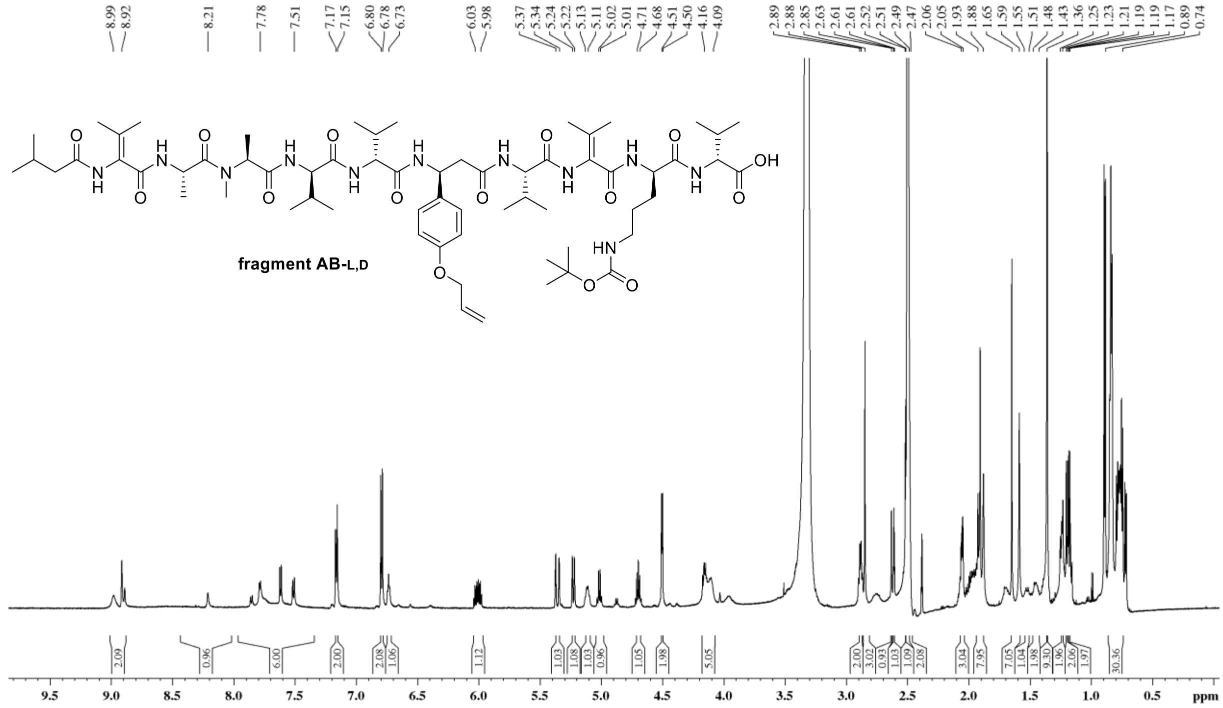
8.99 8.92 8.21 7.78 7.51 7.17 7.15 6.80 6.78 6.73 6.03 5.98 5.37 5.34 5.24 5.22 5.13 5.11 5.02 5.01 4.71 4.68 4.51 4.50 4.16 4.09 2.89 2.88 2.85 2.63 2.61 2.61 2.52 2.51 2.49 2.47 2.06 2.05 1.93 1.88 1.65 1.59 1.55 1.51 1.48 1.43 1.36 1.25 1.23 1.21 1.19 1.19 1.17 0.89 0.74
fragment AB-L,D
2.09 0.96 6.00 2.00 2.08 1.06 1.12 1.03 1.08 1.03 0.96 1.05 1.98 5.05 2.00 3.02 0.93 1.03 1.09 2.08 3.04 7.95 7.05 1.04 1.98 9.30 1.96 2.06 1.97 30.36
ppm

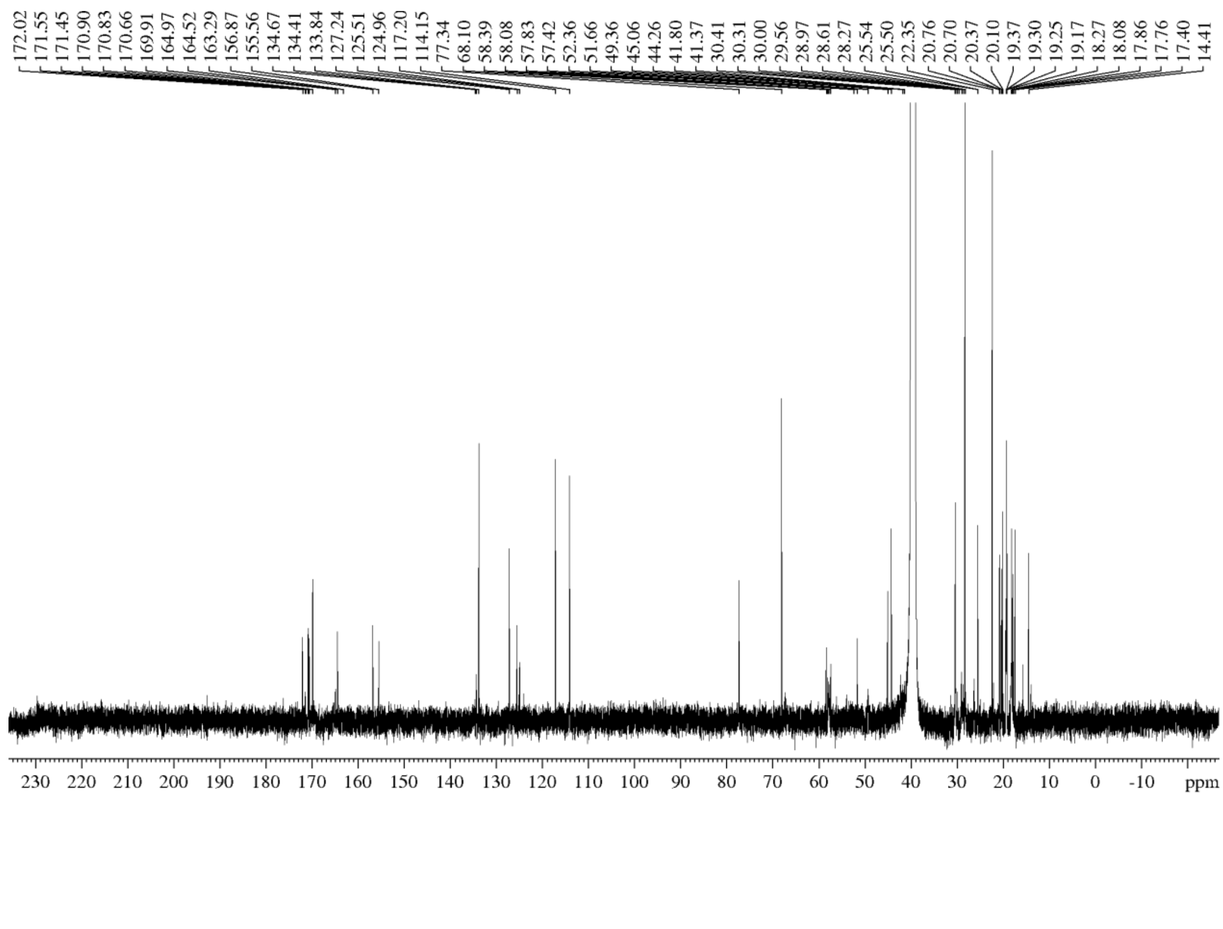
172.02 171.55 171.45 170.90 170.83 170.66 169.91 164.97 164.52 163.29 156.87 155.56 134.67 134.41 133.84 127.24 125.51 124.96 117.20 114.15 77.34 68.10 58.39 58.08 57.83 57.42 52.36 51.66 49.36 45.06 44.26 41.80 41.37 30.41 30.31 30.00 29.56 28.97 28.61 28.27 25.54 25.50 22.35 20.76 20.70 20.37 20.10 19.37 19.30 19.25 19.17 18.27 18.08 17.86 17.76 17.40 14.41
230 220 210 200 190 180 170 160 150 140 130 120 110 100 90 80 70 60 50 40 30 20 10 0 -10 ppm

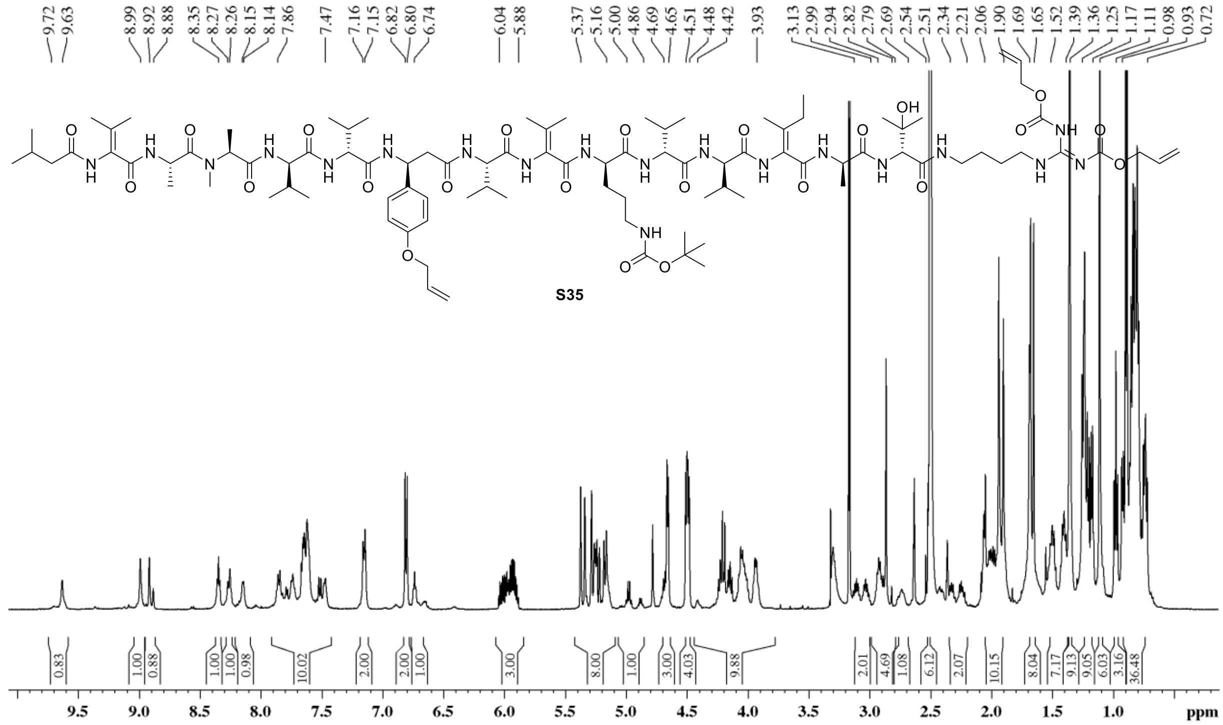
9.72 9.63 8.99 8.92 8.88 8.35 8.27 8.26 8.15 8.14 7.86 7.47 7.16 7.15 6.82 6.80 6.74 6.04 5.88 5.37 5.16 5.00 4.86 4.69 4.65 4.51 4.48 4.42 3.93 3.13 2.99 2.94 2.82 2.79 2.69 2.54 2.51 2.34 2.21 2.06 1.90 1.69 1.65 1.52 1.39 1.36 1.25 1.17 1.11 0.98 0.93 0.72
OH
S35
0.83 1.00 0.88 1.00 1.00 0.98 10.02 2.00 2.00 1.00 3.00 8.00 1.00 3.00 4.03 9.88 2.01 4.69 1.08 6.12 2.07 10.15 8.04 7.17 9.13 9.05 6.03 3.16 36.48
9.5 9.0 8.5 8.0 7.5 7.0 6.5 6.0 5.5 5.0 4.5 4.0 3.5 3.0 2.5 2.0 1.5 1.0 ppm

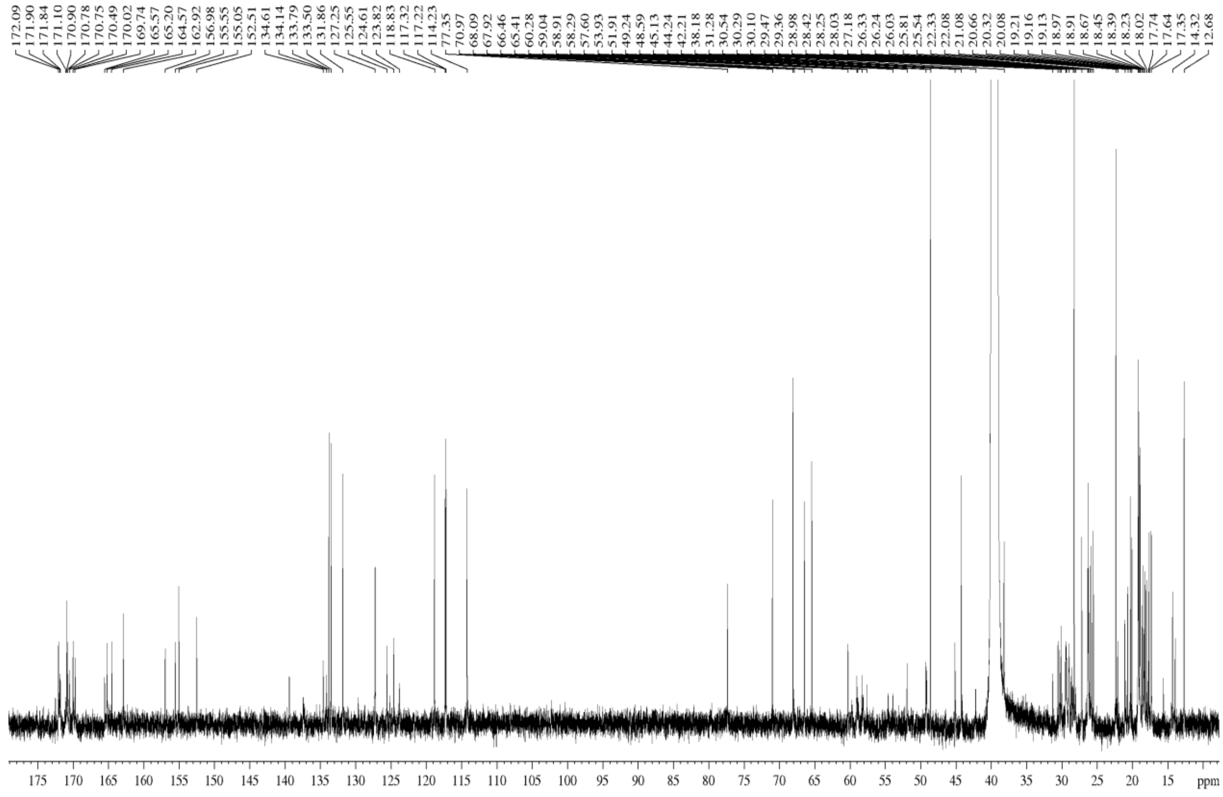
172.09 171.90 171.84 171.10 170.90 170.78 170.75 170.49 170.02 169.74 165.57 165.20 164.57 162.92 156.98 155.55 155.05 152.51 134.61 134.14 133.79 133.50 131.86 127.25 125.55 124.61 123.82 118.83 117.32 117.22 114.23 77.35 70.97 68.09 67.92 66.46 65.41 60.28 59.04 58.91 58.29 57.60 53.93 51.91 49.24 48.59 45.13 44.24 42.21 38.18 31.28 30.54 30.29 30.10 29.47 29.36 28.98 28.42 28.25 28.03 27.18 26.33 26.24 26.03 25.81 25.54 22.33 22.08 21.08 20.66 20.32 20.08 19.21 19.16 19.13 18.97 18.91 18.67 18.45 18.39 18.23 18.02 17.74 17.64 17.35 14.32 12.68
175 170 165 160 155 150 145 140 135 130 125 120 115 110 105 100 95 90 85 80 75 70 65 60 55 50 45 40 35 30 25 20 15 ppm

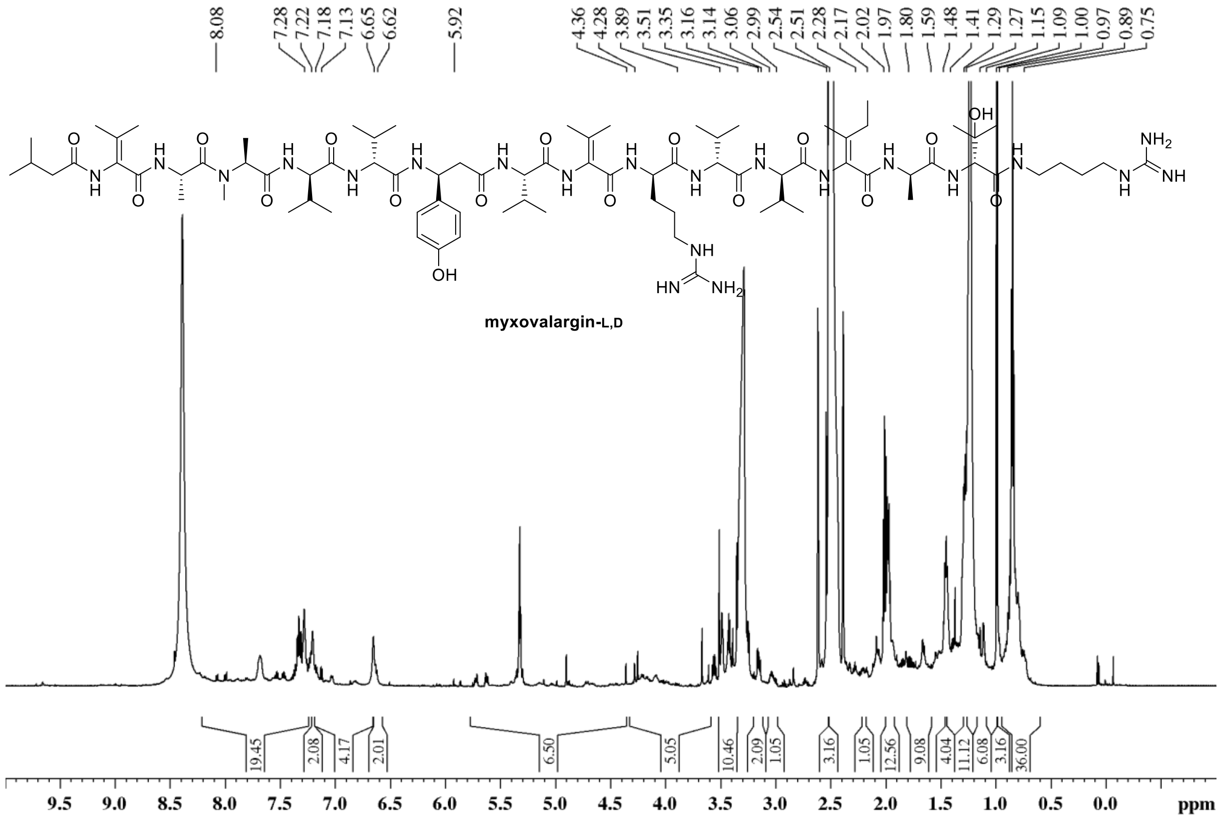
8.08
7.28
7.22
7.18
7.13
6.65
6.62
5.92
4.36
4.28
3.89
3.51
3.35
3.16
3.14
3.06
2.99
2.54
2.51
2.28
2.17
2.02
1.97
1.80
1.59
1.48
1.41
1.29
1.27
1.15
1.09
1.00
0.97
0.89
0.75
myxovalargin-L,D
19.45
2.08
4.17
2.01
6.50
5.05
10.46
2.09
1.05
3.16
1.05
12.56
9.08
4.04
11.12
6.08
3.16
36.00
9.5 9.0 8.5 8.0 7.5 7.0 6.5 6.0 5.5 5.0 4.5 4.0 3.5 3.0 2.5 2.0 1.5 1.0 0.5 0.0 ppm

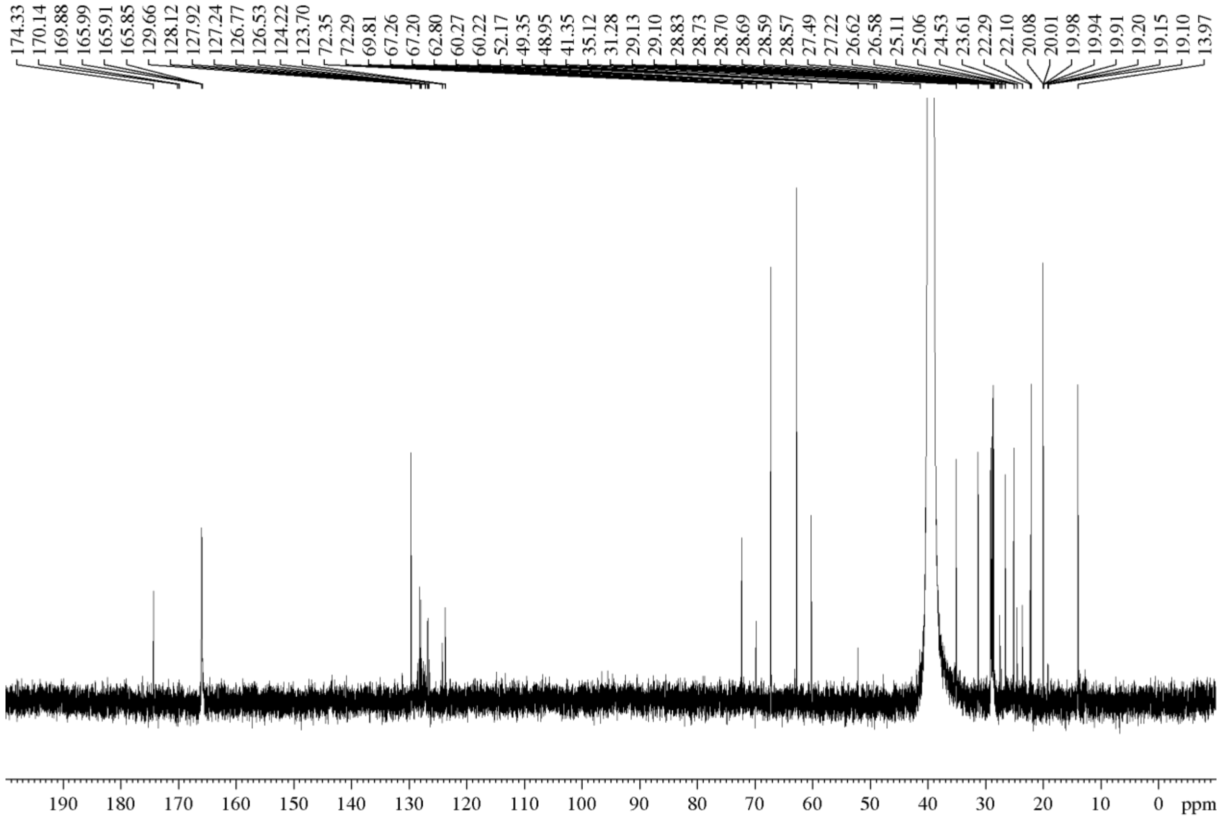
174.33
170.14
169.88
165.99
165.91
165.85
129.66
128.12
127.92
127.24
126.77
126.53
124.22
123.70
72.35
72.29
69.81
67.26
67.20
62.80
60.27
60.22
52.17
49.35
48.95
41.35
35.12
31.28
29.13
29.10
28.83
28.73
28.70
28.69
28.59
28.57
27.49
27.22
26.62
26.58
25.11
25.06
24.53
23.61
22.29
22.10
20.08
20.01
19.98
19.94
19.91
19.20
19.15
19.10
13.97
190 180 170 160 150 140 130 120 110 100 90 80 70 60 50 40 30 20 10 0 ppm

Copy of ^{1}H-NMR spectrum of natural myxovalargin A (solvent= DMSO-d_6).

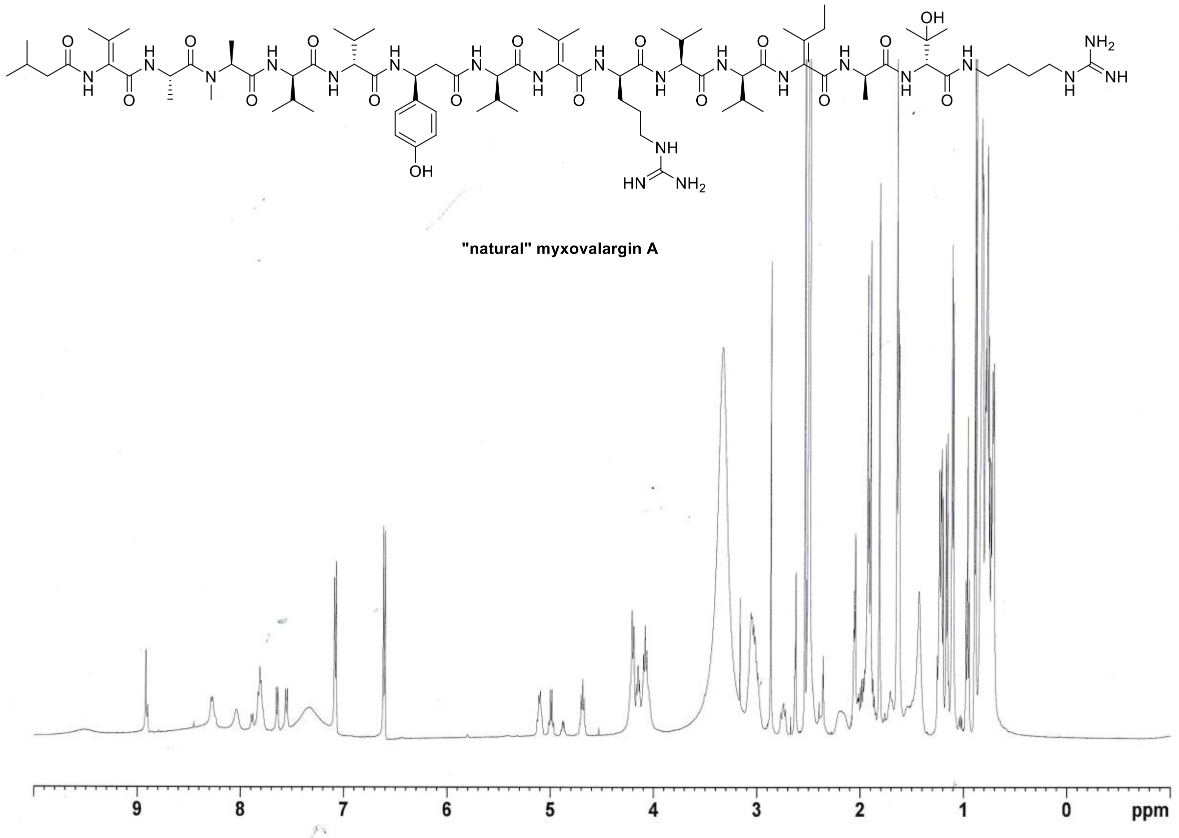

7. Cryo-EM structures

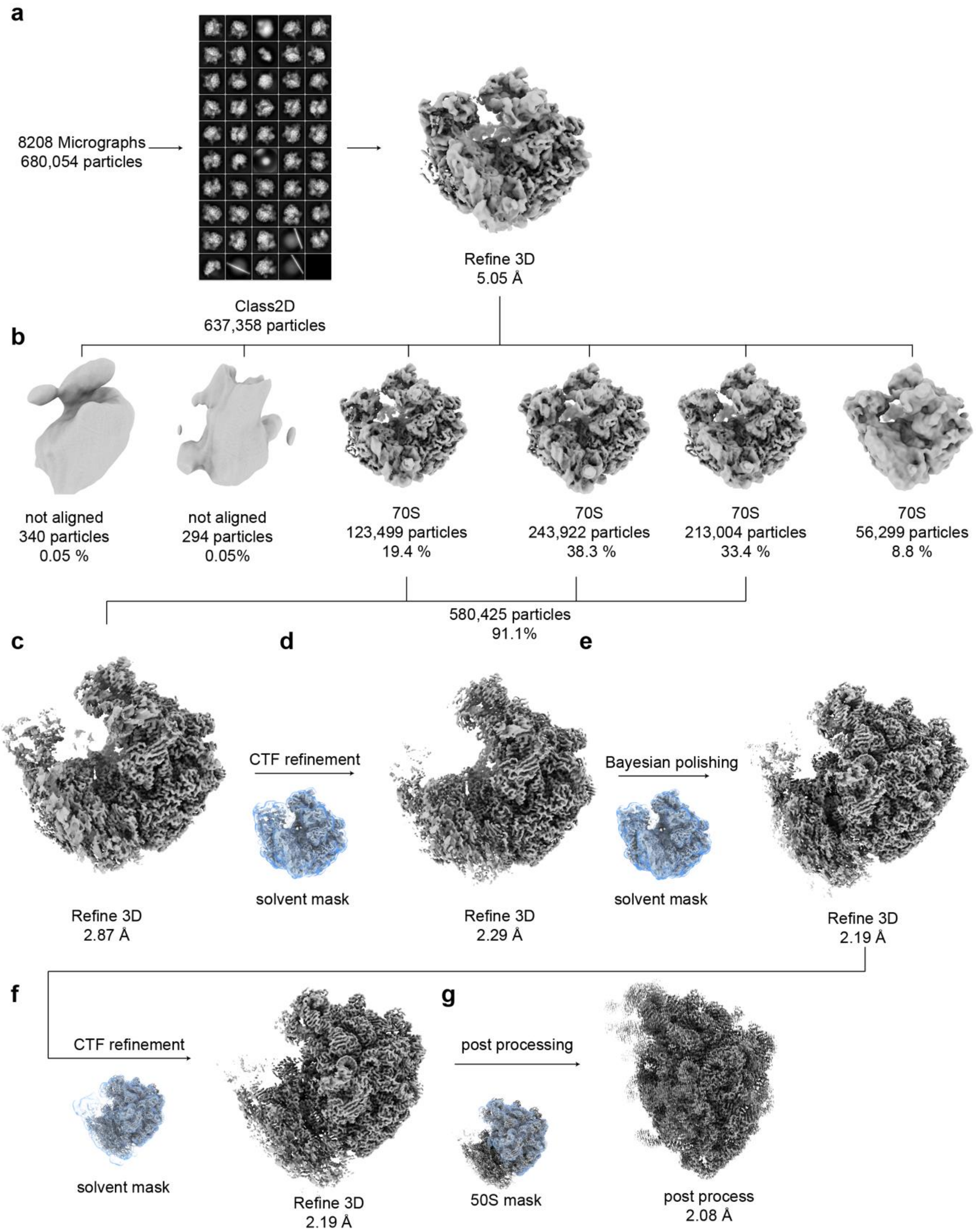

Fig. S7-1: *In silico* sorting of the cryo-EM dataset for the MyxA-70S complex. a, 680,054 particles were picked from 8,208 micrographs and subjected to 2D classification, resulting in 637,358 ribosome-like particles and an initial three times decimated 3D reconstruction of the 70S ribosome at 5.05 Å. **b**, After 100 iterations, 3D classification yielded four classes of 70S ribosomes and two classes of non-aligning species. **c-e**, Classes containing well-resolved 70S were combined (despite differences in the degree of 30S subunit rotation) to increase the number of particles to 580,425 (91.1% of the total) and subjected to **c**, 3D refinement, **d**, CTF refinement and **e**, Bayesian polishing, yielding a final reconstruction of the MyxA-50S map (50S masked) with a final average resolution of 2.10 Å. In **c-d**, the insets show the solvent or 50S masks that were applied during processing.

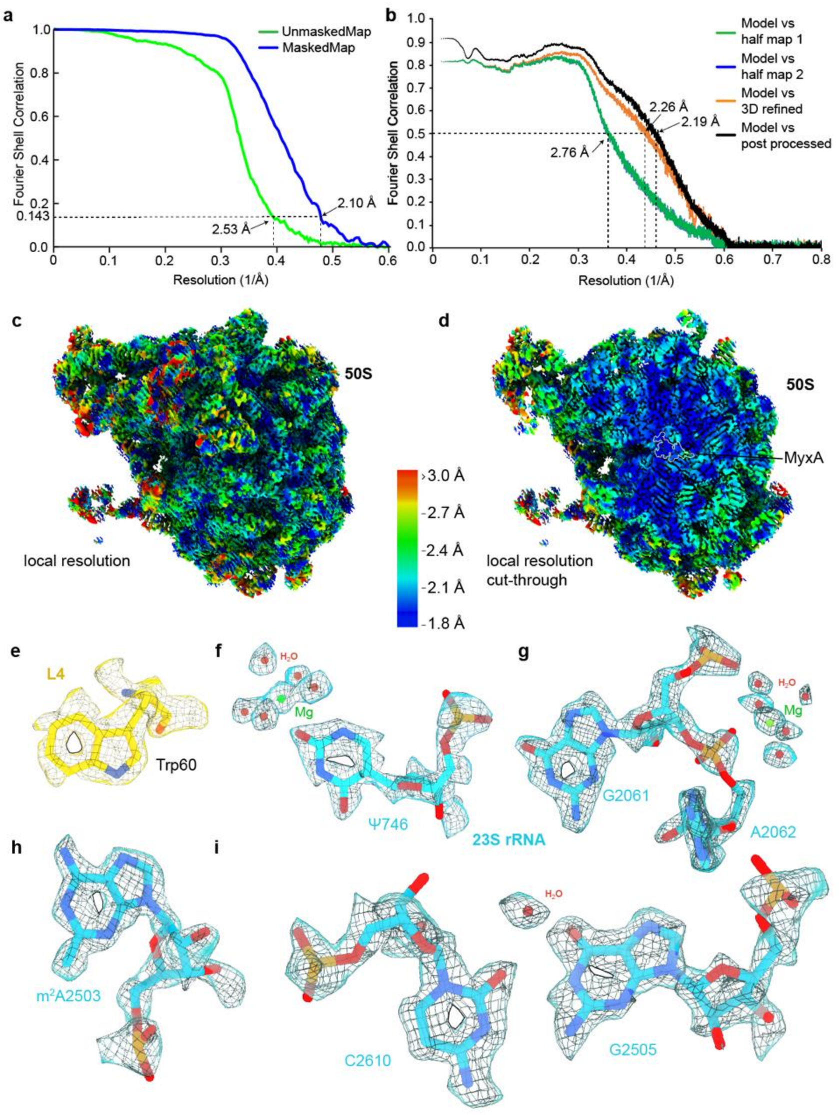

Fig. S7-2: Average and local resolutions of the MyxA-70S complex. a, Fourier Shell Correlation (FSC) of unmasked and masked reconstruction of the MyxA-70S complex, indicating average resolutions (at 0.143 FSC) of 2.53 Å and 2.10 Å, respectively. **b**, Map vs model cross correlation for the half maps 1 (green), 2 (blue), the unmasked 3D refined (orange) and the masked post processed (black) cryo-EM maps at 0.5 FSC. **c**, overview and **d**,

transverse section of the locally filtered masked post processed cryo-EM map of the MyxA-70S complex colored according to local resolution. The binding position of MyxA within the NPET of the 50S subunit is indicated. **e-j**, Examples of cryo-EM map density to illustrate the quality of the MyxA-50S map. Isolated densities of the post processed (masked) MyxA-50S map are shown for **e,** Trp60 of ribosomal protein L4, and **f-g,** 23S rRNA nucleotides **f**, Ψ746 and **g**, A2062 with hydrated magnesium ions, **h**, methylation on A2503 (m^2A2503) and **j**, coordinated water molecule between C2610 and G2505.

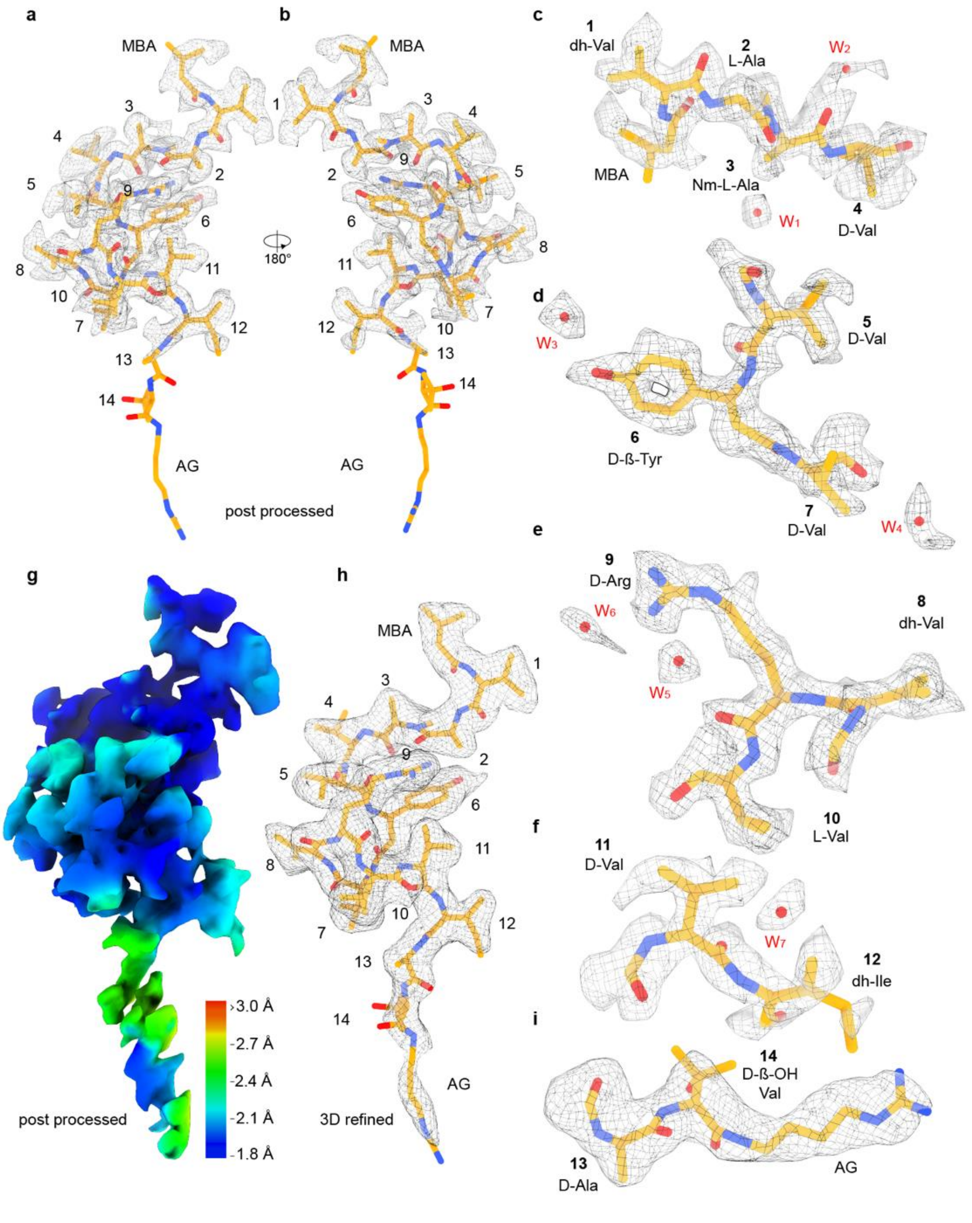

Fig. S7-3: Molecular model of the MyxA into the cryo-EM map density. a-b, Two views of the molecular model of MyxA (orange) modelled into the cryo-EM map density (mesh) shown at high threshold. Residues between 3-methylbutanoic acid (MBA) and agmatine (AG) are numbered from 1-14. **c-f**, Zoom into regions of **a-b** showing the correlation between the cryo-EM map density (mesh) and the molecular model for MyxA (orange) with surrounding waters

(red), namely, **c,** MBA, dh-Val1, L-Ala2, Nm-L-Ala3 and D-Val4 with waters W_1 and W_2 (red), **d,** D-Val5, D-β-Tyr6, D-Val7 with waters W_3, and W_4, **e,** dh-Val8, D-Arg9 and L-Val10 with waters W_5 and W_6, **f,** D-Val11 and dh-Ile12 with water W_7. **g-h**, same view as **a-b** but at lower threshold showing additional density for the agmatine (AG) moiety. In **g,** the map is colored according to local resolution, whereas in **h,** the map is shown as mesh with the molecular model of MyxA (orange). **i,** While density was observed for the AG moiety at lower threshold (**g, h**), only a tentative model for D-Ala13, D-β-OH-Val14 and AG could be generated.

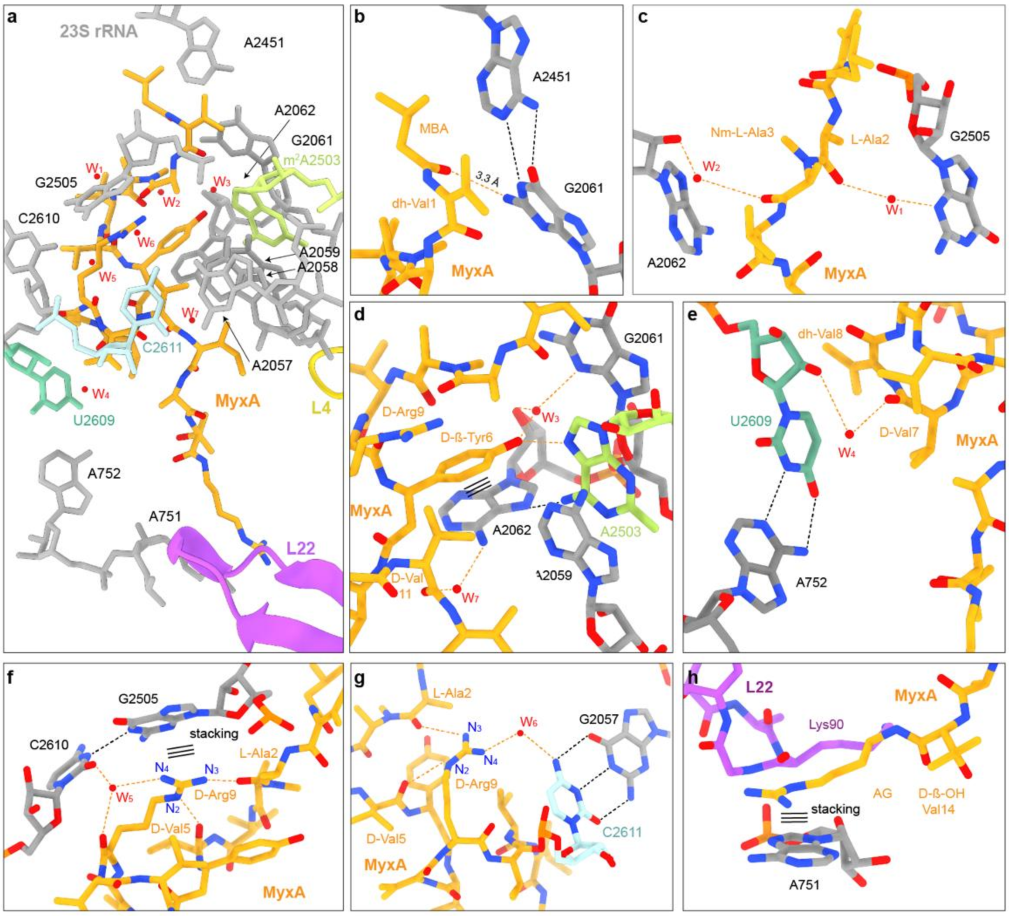

Fig. S7-4: Interactions of MyxA with the large 50S subunit. a, Overview of the MyxA binding site with MyxA (orange) surrounded by 23S rRNA nucleotides (grey), ribosomal proteins L4 (yellow) and L22 (purple) as well as putative water molecules W_1-W_7 (red). Identified *M. tuberculosis* $MyxA^R$ mutations equivalent to *E. coli* positions U2609 (turquoise), C2611 (cyan) and A2503 (lime) are highlighted. **b-h**, Interactions of MyxA (orange) with 23S rRNA nucleotides (grey) and waters (red), which are described in detail: **b**, the O1 carboxyl of the MBA moiety of MyxA comes within hydrogen bonding distance of G2061. **c**, the O1 of L-Ala2 and Nm-L-Ala3 of MyxA establish water-mediated interactions via W_1 with the N3 of G2505 and via W_2 with the 2'OH of A2062, respectively. **d**, The D-β-Tyr6 of MyxA stacks upon A2062 and its OH group of D-β-Tyr6 can hydrogen bond with the N7 of m^2A2503 (lime), as well as with water W_3, which is also coordinated by the N3 of G2061 and the 2'OH of A2062. This position of A2062 is further stabilized by water-mediated interaction between W_7 and the O1 of D-Val11 of MyxA, as well as a potential hydrogen bond with N6 of m^2A2503. **e**, the O1 of D-

Val 7 of MyxA is within hydrogen bond distance of the 2’OH of U2609 (turquoise), which itself forms a Watson-Crick base-pair with A752 (grey). **f-g**, The D-Arg9 sidechain of MyxA stacks upon G2505 and the N2 and N3 atoms form potential intramolecular hydrogen bonds with O1 of D-Val5 and L-Ala2, respectively. **f**, In addition, the N4 and O1 of D-Arg9 establish water-mediated interactions via W_5 with the O2 of C2610. **g**, The N4 of D-Arg9 can also form a water-mediated interaction via W_6 with the N4 of C2611 (cyan), which itself is Watson-Crick-base paired with G2057. **h**, Although less well-resolved, the arginine-like sidechain of the AG of MyxA appears to stack upon A751 and comes within close proximity to Lys90 of ribosomal protein L22 (purple).

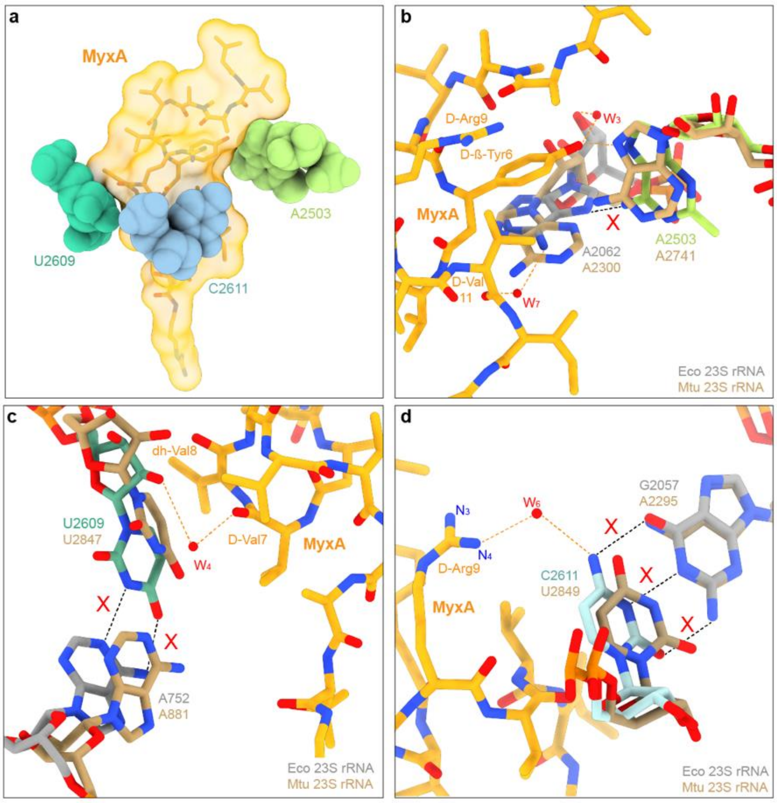

Fig. S7-5: MyxA^R resistance mutations impact on water-mediated interactions. a, Overview of the MyxA molecular model shown as transparent surface representation (orange) surrounded by *E.coli* 23S rRNA nucleotides A2503 (lime), U2609 (turquoise) and C2611 (cyan). **b**, Mutation at position A2503G (lime) (MtuA2741G, brown, PDB ID 5V93)[27] is predicted to break potential hydrogen bond of N6 of m^2A2503 with N3 of A2062 (MtuA2300, brown, PDB ID 5V93)[27] and destabilizing both stacking of D-β-Tyr6 onto A2062 (grey) and abolishing potential hydrogen bond interaction with W_3 (red). **c**, Mutation at position U2609C (turquoise, MtuU2847C, brown, PDB ID 5V93)[27] is predicted to break the Watson-Crick-base pair with A752 (grey, MtuA881, brown, PDB ID 5V93)[27] and abolishing potential hydrogen bond with W_4 (red). **d**, Mutation at position C2611G (cyan, MtuU2849G, brown, PDB ID 5V93)[27] is predicted to break the Watson-Crick-base pair with G2507 (MtuA2295, brown, PDB ID 5V93)[27] and abolishing the potential hydrogen bond interaction of N4 of D-Arg9 with W_6.

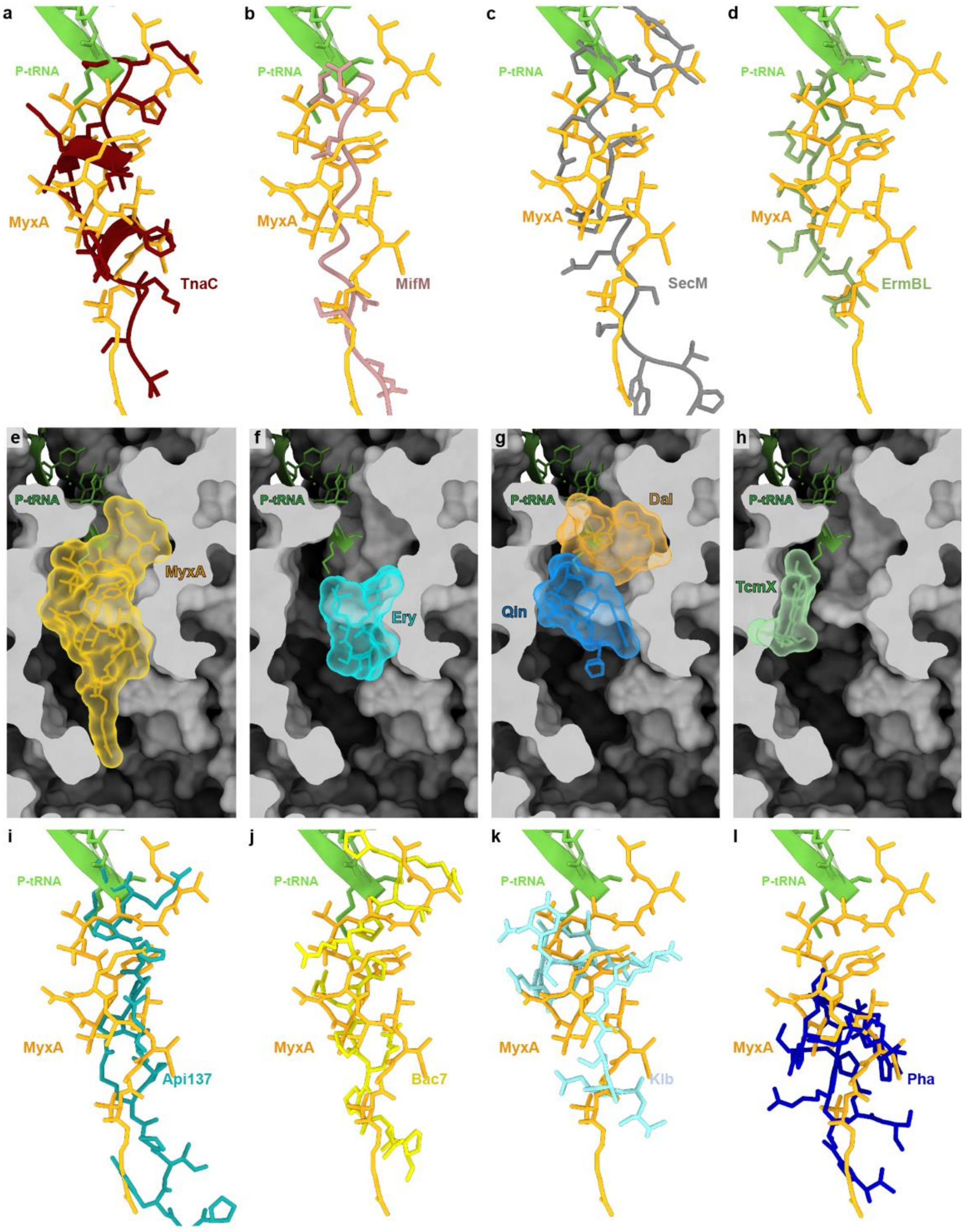

Fig. S7-6: Comparison of MyxA and other tunnel binding ligands. a-d, Comparison of the binding position of MyxA (orange) with nascent polypeptide chains stallers **a**, TnaC (red, PDB ID 7O19)[28], **b**, MifM (brown, PDB ID 3J9W)[29], **c**, SecM (grey, PDB ID 3JBV)[30] and **d**, ErmBL (green, PDB ID 5JU8)[31]. The position of the P-site-tRNA (P-tRNA, green, PDB ID 1VY4)[32] is shown for reference. **e-h**, Comparison of the binding position of **e**, MyxA (orange) with the tunnel-binding antibiotics **f**, erythromycin (Ery, cyan, PDB ID 4V7U)[33], **g**, dalfopristin (Dal, orange) and quinupristin (Qin, blue, PDB ID 4U26)[34] and **h**, tetracenomycin X (TcmX, green, PDB ID 6Y69)[35]. In **e-h**, the antibiotics are shown as transparent surface representations, the nascent peptide exit tunnel as a surface and P-site tRNA (P-tRNA, green, PDBID: 1VY4)[32] using the stick representation. **i-l**, Comparison of the binding position of MyxA (orange) with **i-j**, proline-rich antimicrobial peptides **i**, Api137 (turquoise, PDB ID 5O2R)[36] and **j**, Bac7 (yellow, PDB ID 5HAU)[37], as well as peptide antibiotics klebsazolicin (Klb, cyan, PDB ID 5W4K)[38] and

phazolicin (Pha, blue, PDB ID 6U48)[39]. The P-site tRNA (P-tRNA, green, PDB ID 1VY4)[32] is shown for reference.

Table S7-1. Statistics for the final model of the MyxA-50S complex

**Data collection**	**MyxA-50S complex**<br><br>PDB ID 7QQ3<br><br>EMDB EMD-14121
Microscope	Titan Krios
Detector	K2 DDC
Pixel size (Å)	0.828
Defocus range (µm)	-1.0 to -3.0
Voltage (keV)	300
Electron dose (e^-/Å^2)	1.1
FSC threshold	0.143
Initial particle images (no.)	680,054
Final particle images (no.)	580,425
**Model composition**	
Initial model used (PDB code)	4YBB
Protein residues	2896
RNA nucleotides	3016
Magnesium	145
Water	28

Ligand	1
**Refinement**	
Resolution (Å)	2.1
Map sharpening B factor(Å^2)	-10
**Validation: proteins**	
Poor rotamers (%)	4.22
Ramachandran outliers (%)	0.42
Ramachandran allowed (%)	3.55
Ramachandran favored (%)	96.03
Bad backbone bonds (%)	0
Bad backbone angles (%)	0.825
**Validation: RNA**	
Correct sugar puckers (%)	99.44
Good backbone conformations (%)	87.17
Bad bonds (%)	0.04
Bad angles (%)	0
**Scores**	
MolProbity	1.81
Clash score, all atoms	2.7

Methods

Ribosome purification for cryo-EM sample preparation. The purification of *E. coli* reassociated 70S ribosomes was performed according to Blaha et al.[40] Briefly, *E. coli* K12 MG1655 were grown in LB media at 37°C to an optical density (600 nm) of 0.7 and harvested by centrifugation at 5,000 x g using JLA-8.1000 fixed-angle rotor (Beckmann coulter). Cell pellet was then resuspended in lysis buffer (25 mM Hepes pH 7.5, 100 mM KOAc, 80 mM NH_4Cl, 7 mM $Mg(OAc)_2$, 1 mM dithiothreitol (DTT)) and lysed using a microfluidizer LM10 (Microfluidics) at 15,000 psi. After centrifugation for 10 min in SS-34 rotor (Beckmann coulter) at 15,000 x g, the cleared supernatant was collected and layered onto a 35% sucrose cushion (25 mM Hepes pH 7.5, 100 mM KOAc, 80 mM NH_4Cl, 7 mM $Mg(OAc)_2$, 1 mM DTT, 0.01% (w/v) N-Dodecyl-beta-Maltoside (DDM) and 35% (w/v) sucrose) and crude ribosome fraction was then isolated using Ti45 rotor (Beckmann coulter) by centrifugation for 18 h at 30,000 x g. The pellet was then resuspended using a Dounce homogenizer in buffer A (25 mM Hepes 100 mM KOAc, 80 mM NH_4Cl, 7 mM $Mg(OAc)_2$, 1 mM DTT, 0.01% (w/v) DDM) and tight-coupled 70S were isolated using buffer A 10-40% (w/v) sucrose gradient by centrifugation in SW32 rotor (Beckmann Coulter) at 30,000 x g for 16 h. The 70S peak was then collected using BioComp gradient collector, diluted in ribosome splitting buffer B (25 mM Hepes pH 7.5, 100 mM KOAc, 80 mM NH_4Cl, 1 mM $Mg(OAc)_2$, 1 mM DTT, 0.01% (w/v) DDM) and pelleted by centrifugation at 30,000 x g for 16 h using Ti70 rotor (Beckmann Coulter). 30S and 50S subunit were then isolated using 5-30% sucrose gradient in buffer B. After isolation, pelleting of isolated subunit, reassociation of 70S ribosomes was performed by mixing 1.5:1 30S:50S in 70S reassociation buffer C (25 mM Hepes pH 7.5, 100 mM KOAc, 80 mM NH_4Cl, 7 mM $Mg(OAc)_2$, 1 mM DTT, 0.01% (w/v) DDM) at 37°C for 10 min and isolated by 10-40% sucrose gradient in buffer C using a SW28 rotor (30,000 x g for 16 h). The reassociated 70S ribosomes were then pelleted using Ti70.1 rotor (Beckmann Coulter) at 30,000 x g for 16 h. The pelleted reassociated 70S ribosomes was then resuspended in buffer C, aliquoted and stored at -80°C.

Cryo-EM data collection and single-particle reconstruction of the MyxA-70S complex. Purified *E. coli* 70S ribosomes were mixed with 200 µM MyxA for 15 min at 37 °C in grid buffer (25 mM Hepes pH 7.5, 100mM KOAc, 80 mM NH_4Cl, 15 mM $Mg(OAc)_2$, 1 mM DTT, 0.01% (w/v) DDM). Four microliters of the reaction solution (A_{260} = 7 OD/ml) were applied to pre-coated Quantifoil holey carbon supported grids (R3/3, 3 nm C, Cu 300 mesh, Q44689, C3-C18nCu30-01) and vitrified using a Vitrobot Mark IV (FEI). Data collection was performed on Titan Krios 300V TEM equipped with a K2 direct detection camera (Gatan). Images of single ribosome particles were aligned using MotionCor[41,42] within RELION[43] and 680,054 particles were picked using crYOLO[44] with default settings and a general JANNI model (https://cryolo.readthedocs.io/en/stable/). Defocus values were determined using CTFFind 4[45,46]. Images were processed with RELION 3.1[43] and were subjected to 2D classification resulting in 637,358 ribosome-like particles (**Fig. S7-1a**). An initial 3D

refinement was done using a 70S *E. coli* ribosome as reference (PDB ID 7NSO)[47] lowpass filtered to 60 Å. The resulting reconstruction was 3D classified and yielded three classes of 70S ribosomes with different 30S subunit rotation containing a combined total of 91.1 % of the starting particles. The additional 8.9% were non-aligning or low-resolution particles (**Fig. S7-1b**). After 3D classification the combined 70S particles were 3D refined (**Fig. S7-1c-e**), resolution optimized by CTF refinement and Bayesian polishing, resulting in a final reconstruction of the MyxA-70S complex with an average resolution of 2.5 Å (unmasked) and 2.1 Å (50S masked) (**Fig. S7-1f** and **Fig. S7-2a**) determined using the "gold-standard" criterion ($FSC_{0.143}$).[43] The final reconstructions of each 3D refinement were corrected for the modulation transfer function and sharpened by applying a negative B factor automatically estimated by RELION 3.1.[43] Local resolution estimations were done on the half maps using Bsoft 2.1.0[48] blocres (-box 20 -sampling 0.828 -maxres 1.9 –edge 1 -verbose 1 –origin 0,0,0 -cutoff 0.143 –mask) and local filtering was calculated by blocfilt (-box 20 -sampling 0.828 -edge 1 -verbose 1 -origin 0,0,0 -resolution -mask). Local resolution calculations indicated that the majority of the core of the 50S subunit reached towards 1.8 Å (**Fig. S7-2c-d**), consistent with the well-resolved density for the ribosomal proteins and rRNAs. In particular, holes were visible for many aromatic r-protein sidechains and rRNA nucleobases, as well as highly defined density for rRNA modifications, and many hydrated magnesium ions and individual water molecules (**Fig. S7-2e-i**).

Cryo-EM model building and validation. A high-resolution *E. coli* ribosomal 50S subunit model (PDB ID 4YBB)[49] containing ribosomal proteins and rRNA was initially rigid body fitted to the cryo-EM map density using ChimeraX[50] and then manually adjusted in Coot.[51] The molecular model for MyxA was generated using ChemDraw (PerkinElmer Informatics) with the 3D model calculated with structural restraints from AceDRG (PMID: 28177306, PMID: 28177307) implemented in Coot Lidia[51]. The output model was then manually modelled into the corresponding density with Coot[51]. Model refinement was done in Phenix 1.19.2-4158 using metal and structural restraints calculated by Phenix eLBOW[52] and validated by Phenix comprehensive Cryo-EM validation and MolProbity server[53] (http://molprobity.biochem.duke.edu/) with map vs. model cross correlation at $FSC_{0.5}$ for all individual maps. The statistics of the final model are presented in **Table S7-1**.

Preparation of figures with Cryo-EM structures. ChimeraX[50] was used to isolate density and visualize density images, structural superpositions and alignments.

8. References

1. Ioerger, T. R. *et al.* Variation among genome sequences of H37Rv strains of Mycobacterium tuberculosis from multiple laboratories. *J. Bacteriol.* **192,** 3645–3653; 10.1128/JB.00166-10 (2010).

2. Evans, J. C. *et al.* Validation of CoaBC as a Bactericidal Target in the Coenzyme A Pathway of Mycobacterium tuberculosis. *ACS Infect. Dis.* **2,** 958–968; 10.1021/acsinfecdis.6b00150 (2016).

3. Chengalroyen, M. D. *et al.* DNA-Dependent Binding of Nargenicin to DnaE1 Inhibits Replication in Mycobacterium tuberculosis. *ACS Infect. Dis.* **8,** 612–625; 10.1021/acsinfecdis.1c00643 (2022).

4. Chengalroyen, M. D. *et al.* Biological Profiling Enables Rapid Mechanistic Classification of Phenotypic Screening Hits and Identification of KatG Activation-Dependent Pyridine Carboxamide Prodrugs With Activity Against Mycobacterium tuberculosis. *Front. Cell. Infect. Microbiol.* **10,** 582416; 10.3389/fcimb.2020.582416 (2020).

5. Singh, V. *et al.* The Inosine Monophosphate Dehydrogenase, GuaB2, Is a Vulnerable New Bactericidal Drug Target for Tuberculosis. *ACS Infect. Dis.* **3,** 5–17; 10.1021/acsinfecdis.6b00102 (2017).

6. Agarwal, P. *et al.* Foam Cells Control Mycobacterium tuberculosis Infection. *Front. Microbiol.* **11,** 1394; 10.3389/fmicb.2020.01394 (2020).

7. Singh, R., Barry, C. E. & Boshoff, H. I. M. The three RelE homologs of Mycobacterium tuberculosis have individual, drug-specific effects on bacterial antibiotic tolerance. *J. Bacteriol.* **192,** 1279–1291; 10.1128/JB.01285-09 (2010).

8. Scheid, U. Studies on the biosynthesis of myxobacterial natural products. Untersuchung der Biosynthese myxobakterieller Naturstoffe. Dissertation. Saarland University, Germany, 2021.

9. Garcia, R. & Müller, R. Family Myxococcaceae. In *The Prokaryotes*, edited by E. Rosenberg, E. DeLong, S. Lory, E. Stackebrandt & F. Thompson (Springer-Verlag, Berlin Heidelberg, 2014), Vol. 4th Edition, pp. 191–212.

10. Euzeby, J. List of new names and new combinations previously effectively, but not validly, published. *Int.J.Syst.Evol.Microbiol.* **57,** 893–897; 10.1099/ijs.0.65207-0 (2007).

11. Cortina, N. S., Krug, D., Plaza, A., Revermann, O. & Müller, R. Myxoprincomid: Entdeckung eines Naturstoffs mithilfe einer umfassenden Analyse des sekundären Metaboloms von Myxococcusxanthus. *Angew. Chem.* **124,** 836–841; 10.1002/ange.201106305 (2012).

12. Röttig, M. *et al.* NRPSpredictor2-a web server for predicting NRPS adenylation domain specificity. *Nucleic Acids Res.* **39,** W362-7; 10.1093/nar/gkr323 (2011).

13. Krug, D. & Müller, R. Discovery of additional members of the tyrosine aminomutase enzyme family and the mutational analysis of CmdF. *ChemBioChem* **10,** 741–750; 10.1002/cbic.200800748 (2009).

14. Cortina, N. S., Krug, D., Plaza, A., Revermann, O. & Müller, R. Myxoprincomid: Entdeckung eines Naturstoffs mithilfe einer umfassenden Analyse des sekundären Metaboloms von Myxococcusxanthus. *Angew. Chem.* **124,** 836–841; 10.1002/ange.201106305 (2012).

15. Rottig, M. *et al.* NRPSpredictor2--a web server for predicting NRPS adenylation domain specificity. *Nucleic Acids Res.* **39,** W362-7; 10.1093/nar/gkr323 (2011).

16. Kelley, L. A., Mezulis, S., Yates, C. M., Wass, M. N. & Sternberg, M. J. E. The Phyre2 web portal for protein modeling, prediction and analysis. *Nat. Protoc.* **10,** 845–858; 10.1038/nprot.2015.053 (2015).

17. Tesmar, A. von. Investigation of bacterial secondary metabolite pathways, Dissertation, Saarland University, Germany, 2017.

18. Ishida, K. *et al.* Biosynthesis and structure of aeruginoside 126A and 126B, cyanobacterial peptide glycosides bearing a 2-carboxy-6-hydroxyoctahydroindole moiety. *Chem. Biol.* **14,** 565–576; 10.1016/j.chembiol.2007.04.006 (2007).

19. Ishida, K. *et al.* Plasticity and evolution of aeruginosin biosynthesis in cyanobacteria. *Appl. Environ. Microbiol.* **75,** 2017–2026; 10.1128/AEM.02258-08 (2009).

20. Fujii, K. *et al.* Comparative study of toxic and non-toxic cyanobacterial products: Novel peptides from toxic Nodularia spumigena AV1. *Tetrahedron Lett.* **38,** 5525–5528; 10.1016/S0040-4039(97)01192-1 (1997).

21. Bitonti, A. J., Casara, P. J., McCann, P. P. & Bey, P. Catalytic irreversible inhibition of bacterial and plant arginine decarboxylase activities by novel substrate and product analogues. *Biochem. J.* **242,** 69–74 (1987).

22. Bock, T., Müller, R. & Blankenfeldt, W. Crystal structure of AibC, a reductase involved in alternative de novo isovaleryl coenzyme A biosynthesis in *Myxococcus xanthus*. *Acta Crystallogr. Sect. F* **72,** 652–658; 10.1107/S2053230X16011146 (2016).

23. Li, Y., Luxenburger, E. & Müller, R. Ein alternativer isovaleryl-CoA-biosyntheseweg: Beteiligung einer bisher unbekannten 3-methylglutaconyl-CoA-decarboxylase. *Angew. Chem.*; 10.1002/ange.201207984 (2012).

24. Li, Y., Luxenburger, E. & Müller, R. An alternative isovaleryl CoA biosynthetic pathway involving a previously unknown 3-methylglutaconyl CoA decarboxylase. *Angew. Chem. Int. Ed. Engl.* **52,** 1304–1308; 10.1002/anie.201207984 (2012).

25. Bode, H. B. *et al.* Determination of the absolute configuration of peptide natural products by using stable isotope labeling and mass spectrometry. *Chem. Eur. J.* **18,** 2342–2348; 10.1002/chem.201103479 (2012).

26. Bretscher, A. P. & Kaiser, D. Nutrition of *Myxococcus xanthus*, a fruiting myxobacterium. *J. Bacteriol.* **133,** 763–768 (1978).

27. Yang, K. *et al.* Structural insights into species-specific features of the ribosome from the human pathogen Mycobacterium tuberculosis. *Nucleic Acids Res* **45,** 10884–10894; 10.1093/nar/gkx785 (2017).

28. van der Stel, A.-X. *et al.* Structural basis for the tryptophan sensitivity of TnaC-mediated ribosome stalling. *Nat. Commun.* **12,** 5340; 10.1038/s41467-021-25663-8 (2021).

29. Sohmen, D. *et al.* Structure of the Bacillus subtilis 70S ribosome reveals the basis for species-specific stalling. *Nat. Commun.* **6,** 6941; 10.1038/ncomms7941 (2015).

30. Zhang, J. *et al.* Mechanisms of ribosome stalling by SecM at multiple elongation steps. *eLife* **4**; 10.7554/eLife.09684 (2015).

31. Arenz, S. *et al.* A combined cryo-EM and molecular dynamics approach reveals the mechanism of ErmBL-mediated translation arrest. *Nat. Commun.* **7,** 12026; 10.1038/ncomms12026 (2016).

32. Polikanov, Y. S., Steitz, T. A. & Innis, C. A. A proton wire to couple aminoacyl-tRNA accommodation and peptide-bond formation on the ribosome. *Nat. Struct. Mol. Biol.* **21,** 787–793; 10.1038/nsmb.2871 (2014).

33. Dunkle, J. A., Xiong, L., Mankin, A. S. & Cate, J. H. D. Structures of the Escherichia coli ribosome with antibiotics bound near the peptidyl transferase center explain spectra of drug action. *Proc. Natl. Acad. Sci. U.S.A.* **107,** 17152–17157; 10.1073/pnas.1007988107 (2010).

34. Noeske, J. *et al.* Synergy of streptogramin antibiotics occurs independently of their effects on translation. *Antimicrob. agents chemothera.* **58,** 5269–5279; 10.1128/AAC.03389-14 (2014).

35. Osterman, I. A. *et al.* Tetracenomycin X inhibits translation by binding within the ribosomal exit tunnel. *Nat. Chem. Biol.* **16,** 1071–1077; 10.1038/s41589-020-0578-x (2020).

36. Florin, T. *et al.* An antimicrobial peptide that inhibits translation by trapping release factors on the ribosome. *Nat. Struct. Mol. Biol.* **24,** 752–757; 10.1038/nsmb.3439 (2017).

37. Gagnon, M. G. *et al.* Structures of proline-rich peptides bound to the ribosome reveal a common mechanism of protein synthesis inhibition. *Nucleic Acids Res.* **44,** 2439–2450; 10.1093/nar/gkw018 (2016).

38. Metelev, M. *et al.* Klebsazolicin inhibits 70S ribosome by obstructing the peptide exit tunnel. *Nat. Chem. Biol.* **13,** 1129–1136; 10.1038/nchembio.2462 (2017).

39. Travin, D. Y. *et al.* Structure of ribosome-bound azole-modified peptide phazolicin rationalizes its species-specific mode of bacterial translation inhibition. *Nat. Commun.* **10,** 4563; 10.1038/s41467-019-12589-5 (2019).

40. Blaha, G. *et al.* Preparation of functional ribosomal complexes and effect of buffer conditions on tRNA positions observed by cryoelectron microscopy. *Complex Enzymes in Microbial Natural Product Biosynthesis, Part B: Polyketides, Aminocoumarins and Carbohydrates* **317,** 292–309; 10.1016/s0076-6879(00)17021-1 (2000).

41. Zheng, S. Q. *et al.* MotionCor2: anisotropic correction of beam-induced motion for improved cryo-electron microscopy. *Nat. Methods* **14,** 331–332; 10.1038/nmeth.4193 (2017).

42. Li, X. *et al.* Electron counting and beam-induced motion correction enable near-atomic-resolution single-particle cryo-EM. *Nat. Methods* **10,** 584–590; 10.1038/nmeth.2472 (2013).

43. Scheres, S. H. W. RELION: implementation of a Bayesian approach to cryo-EM structure determination. *J. Struct. Biol.* **180,** 519–530; 10.1016/j.jsb.2012.09.006 (2012).

44. Wagner, T. *et al.* SPHIRE-crYOLO is a fast and accurate fully automated particle picker for cryo-EM. *Communications Biol.* **2,** 218; 10.1038/s42003-019-0437-z (2019).

45. Mindell, J. A. & Grigorieff, N. Accurate determination of local defocus and specimen tilt in electron microscopy. *J. Struct. Biol.* **142,** 334–347; 10.1016/s1047-8477(03)00069-8 (2003).

46. Rohou, A. & Grigorieff, N. CTFFIND4: Fast and accurate defocus estimation from electron micrographs. *J. Struct. Biol.* **192,** 216–221; 10.1016/j.jsb.2015.08.008 (2015).

47. Beckert, B. *et al.* Structural and mechanistic basis for translation inhibition by macrolide and ketolide antibiotics. *Nat. Commun.* **12,** 4466; 10.1038/s41467-021-24674-9 (2021).

48. Heymann, J. B. Guidelines for using Bsoft for high resolution reconstruction and validation of biomolecular structures from electron micrographs. *Prot. Sci.* **27,** 159–171; 10.1002/pro.3293 (2018).

49. Noeske, J. *et al.* High-resolution structure of the Escherichia coli ribosome. *Nat. Struct. Mol. Biol.* **22,** 336–341; 10.1038/nsmb.2994 (2015).

50. Goddard, T. D. *et al.* UCSF ChimeraX: Meeting modern challenges in visualization and analysis. *Prot. Sci.* **27,** 14–25; 10.1002/pro.3235 (2018).

51. Emsley, P., Lohkamp, B., Scott, W. G. & Cowtan, K. Features and development of Coot. *Acta Crystallogr. D, Biol. Crystallogr.* **66,** 486–501; 10.1107/S0907444910007493 (2010).

52. Moriarty, N. W., Grosse-Kunstleve, R. W. & Adams, P. D. electronic Ligand Builder and Optimization Workbench (eLBOW): a tool for ligand coordinate and restraint generation. *Acta Crystallogr. D, Biol. Crystallogr.* **65,** 1074–1080; 10.1107/S0907444909029436 (2009).

53. Chen, V. B. *et al.* MolProbity: all-atom structure validation for macromolecular crystallography. *Acta Crystallogr. D, Biol. Crystallogr.* **66,** 12–21; 10.1107/S0907444909042073 (2010).
